# PREDICT-CP: study protocol of implementation of comprehensive surveillance to predict outcomes for school-aged children with cerebral palsy

**DOI:** 10.1136/bmjopen-2016-014950

**Published:** 2017-07-12

**Authors:** Roslyn N Boyd, Peter SW Davies, Jenny Ziviani, Stewart Trost, Lee Barber, Robert Ware, Stephen Rose, Koa Whittingham, Leanne Sakzewski, Kristie Bell, Christopher Carty, Steven Obst, Katherine Benfer, Sarah Reedman, Priya Edwards, Megan Kentish, Lisa Copeland, Kelly Weir, Camilla Davenport, Denise Brooks, Alan Coulthard, Rebecca Pelekanos, Andrea Guzzetta, Simona Fiori, Meredith Wynter, Christine Finn, Andrea Burgess, Kym Morris, John Walsh, Owen Lloyd, Jennifer A Whitty, Paul A Scuffham

**Affiliations:** 1 Queensland Cerebral Palsy and Rehabilitation Research Centre (QCPRRC), The University of Queensland, Brisbane, Queensland, Australia; 2 Queensland Paediatric Rehabilitation Service, Lady Cilento Children's Hospital, Brisbane, Queensland, Australia; 3 Children's Nutrition Research Centre, The University of Queensland, Brisbane, Queensland, Australia; 4 School of Health and Rehabilitation Sciences, The University of Queensland, Brisbane, Queensland, Australia; 5 Institute of Health and Biomedical Innovation, Queensland University of Technology, Brisbane, Queensland, Australia; 6 Menzies Health Institute Queensland, Griffith University, Gold Coast, Queensland, Australia; 7 CSIRO Australian e-Health Research Centre, Canberra, Australia; 8 Medical Imaging, Diagnostic and Interventional Neuroradiology, Royal Brisbane and Women’s Hospital, Brisbane, Queensland, Australia; 9 Queensland Children's Motion Analysis Service, Lady Cilento Children's Hospital, Brisbane, Queensland, Australia; 10 Clinical Governance, Education and Research, Gold Coast Health, Brisbane, Queensland, Australia; 11 Centre for Clinical Research, The University of Queensland, Brisbane, Queensland, Australia; 12 Department of Developmental Neuroscience, Instituto Di Ricovero E Cura A Carattere Scientifico (IRCCS), Pisa, Italy; 13 Department of Paediatric Orthopaedics, The Mater Health Services, Brisbane, Queensland, Australia; 14 Norwich Medical School, University of East Anglia, Norwich, UK; 15 School of Pharmacy, The University of Queensland, Brisbane, Queensland, Australia

**Keywords:** cerebral palsy, longitudinal cohort, motor development, brain structure and function, communication, gross motor function, manual ability

## Abstract

**Objectives:**

Cerebral palsy (CP) remains the world’s most common childhood physical disability with total annual costs of care and lost well-being of $A3.87b. The PREDICT-CP (NHMRC 1077257 Partnership Project: Comprehensive surveillance to PREDICT outcomes for school age children with CP) study will investigate the influence of brain structure, body composition, dietary intake, oropharyngeal function, habitual physical activity, musculoskeletal development (hip status, bone health) and muscle performance on motor attainment, cognition, executive function, communication, participation, quality of life and related health resource use costs. The PREDICT-CP cohort provides further follow-up at 8–12 years of two overlapping preschool-age cohorts examined from 1.5 to 5 years (NHMRC 465128 motor and brain development; NHMRC 569605 growth, nutrition and physical activity).

**Methods and analyses:**

This population-based cohort study undertakes state-wide surveillance of 245 children with CP born in Queensland (birth years 2006–2009). Children will be classified for Gross Motor Function Classification System; Manual Ability Classification System, Communication Function Classification System and Eating and Drinking Ability Classification System. Outcomes include gross motor function, musculoskeletal development (hip displacement, spasticity, muscle contracture), upper limb function, communication difficulties, oropharyngeal dysphagia, dietary intake and body composition, participation, parent-reported and child-reported quality of life and medical and allied health resource use. These detailed phenotypical data will be compared with brain macrostructure and microstructure using 3 Tesla MRI (3T MRI). Relationships between brain lesion severity and outcomes will be analysed using multilevel mixed-effects models.

**Ethics and dissemination:**

The PREDICT-CP protocol is a prospectively registered and ethically accepted study protocol. The study combines data at 1.5–5 then 8–12 years of direct clinical assessment to enable prediction of outcomes and healthcare needs essential for tailoring interventions (eg, rehabilitation, orthopaedic surgery and nutritional supplements) and the projected healthcare utilisation.

**Trial registration number:**

ACTRN: 12616001488493

**Strengths and limitations of this study:**

- The PREDICT-CP prospective cohort study provides comprehensive phenotypical data on a representative cohort of children with cerebral palsy.
- The longitudinal follow-up of this cohort (at 2–5 years and now cross-sectional at 8–12 years) will enable development of prediction models of outcome.
- Brain structure (macrostructure and microstructure at 3.0 T) will be compared with comprehensive motor, cognitive and communication outcomes at school age.
- A limitation is that only brain macrostructure at 1.5 T has been captured from early clinical brain MRI scans as part of clinical practice.

## Background

Cerebral palsy (CP) is a disorder of movement and posture secondary to an insult to the developing brain.^1^ The insult is static and permanent and may be the consequence of different factors, including both genetic and environmental causes. Although the insult is static, the consequent symptoms are variable and may change over time.^2^ The disability increases with age and ageing occurs earlier.^3^ Children may have a range of comorbidities,^4^ which are likely to impact outcomes and costs of care.^5^ Based on CP registers, a recent systematic review identified that in children diagnosed with CP at 5 years: 3 in 4 were in pain; 1 in 2 had an intellectual disability; 1 in 3 could not walk; 1 in 3 had hip displacement; 1 in 4 could not talk; 1 in 4 had epilepsy; 1 in 4 had a behaviour disorder; 1 in 4 had bladder control problems; 1 in 5 had a sleep disorder; 1 in 5 dribbled; 1 in 10 were blind; 1 in 15 were tube fed and 1 in 25 were deaf.^6^ It is known that peak motor attainment in CP is reached at 8–9 years and tends to plateau before a decline in adolescence.^3^ Secondary musculoskeletal disorders involving muscle, tendons, bones and joints are common as a result of spasticity, muscle weakness and immobility. CP has substantial lifelong effects on daily function, societal participation and quality of life (QoL) for children and their families. There is a paucity of data on the relationship between physical outcomes and school attainment.^7^ Better prediction of outcomes is important for families and healthcare providers.^8^

In Australia, CP remains the most common physical disability in children with ≈ 700 infants born each year that will be later diagnosed with CP.^9^ The overall costs to society of persons with CP was $A1.47b per year (0.14% of GDP), with an average annual cost of $A43 431 per individual.^10^ When taking into account the value of lost well-being (disability and premature death), the total costs were $A3.87b per year or $A115 000 per person. CP has a lifetime impact at a total cost of over $A2M per person.^10^ More recently, in a preschool-aged cohort (CP-Child, National Health and Medical Research Council NHMRC 465128), we have determined a strong relationship between severity of Gross Motor Function Classification System (GMFCS) levels I–V and a stepwise increase in incremental costs of care.^5^

The ability to better predict outcomes has the potential to guide intervention to reduce adverse outcomes (hip dislocation, poor growth, undernutrition or overnutrition, respiratory health complications from oropharyngeal dysphagia (OPD), pain, reduced participation in the community and under attainment at school). Development of prediction models based on early brain structure and function can inform health and social care provision (eg, via the National Disability Insurance Scheme (NDIS)) and provide best practice comprehensive surveillance to allow implementation of timely and effective interventions to achieve optimal outcomes.

Understanding the relationship between specific brain MRI appearance and outcome measures such as motor function is critically important.^11^ Such data may prove invaluable in providing accurate prognostic counselling at the time of diagnosis, as well as potentially guiding the most appropriate treatments tailored to each individual’s pattern of CP and type and severity of the brain lesion on imaging.^12^ A focus of the majority of epidemiological research is the prevention of CP, which requires clinical outcomes to be correlated with the presumed timing and aetiology of lesions in the developing brain.^11^ Pathological insults in the developing brain cause abnormalities or lesions, which may be detected by brain MRI, and the patterns of these lesions depend on the stage and/or presumed timing of the injury during brain development.^13^ Using this principle, a qualitative system of classification is established, whereby lesions can be identified as brain maldevelopments (occurring in the first and second trimesters),^11^ periventricular white matter lesions (occurring early in the third trimester and in preterm infants) or grey matter lesions (occurring late in the third trimester and at term).^11^ A systematic review found studies with enough MRI data for subjects to be classified into these presumed lesion timing groups, and in the majority of studies this lesion timing classification was able to be linked to at least one measure of motor outcome.^11^ There are limited data on brain lesion severity, brain microstructure and quantitative comprehensive outcomes.^11^

In the Australian CP-child study, entire birth years of Victorian and Queensland born children with CP across the full spectrum of gross motor abilities were prospectively followed to determine the relationship between the rate and limit of motor development (gross and fine motor function) as related to the nature of the brain lesion.^12 14^ Representative population-based data have been reported on i) early development and prediction of hip outcomes,^15^ ii) the relationship between brain structure and motor development^12^ and iii) social function^16^ and communication^17^ with cost and health resource use data across the spectrum of functional severity.^5^ The cross-sectional domains of school readiness (mobility, self-care, social function, communication) were reported at school entry.^16 18^

In CP, there is a likely relationship between the severity of the early brain injury on structural MRI (nature, extent, presumed timing), early motor status at 3 years and later outcomes at 8–12 years (motor attainment, musculoskeletal performance, hip displacement). In Sweden, Norway and Scotland, a population-wide surveillance programme (CP-UP) has been implemented for up to 10 years.^19^ Since implementation in Southern Sweden, no child with CP has had a dislocated hip,^19^ musculoskeletal contractures have been reduced^20^ and nutrition and bone health are monitored.^21 22^ National hip surveillance best practice guidelines have been developed and implemented in Australia,^23^ and in Queensland population-wide hip surveillance has been implemented.^24^

The PREDICT-CP study will undertake further comprehensive follow-up of four birth years of children with CP born in Queensland to capture longitudinal data on growth and physical outcomes (motor capacity, muscle and bone health, physical activity, feeding and oropharyngeal function, nutrition), cognition (executive function, educational attainment, communication) and participation, QoL, pain and these are related to costs of healthcare utilisation. The quantitative evaluation of early brain structure on MRI and functional status around 2 years will be compared with these comprehensive outcomes at 8–12 years to build prediction models of CP. Development and implementation of prediction models of outcomes are essential for tailoring interventions (rehabilitation, medical management, orthopaedic surgery, nutritional supplements) and in understanding the likely costs of healthcare.

Growth, nutrition and physical activity are important determinants of health outcomes in children with CP. Knowledge of levels and patterns of habitual physical activity (HPA) for children with CP are important as they have increased risk of inactivity (sedentary behaviour) related illness.^9 25^ In addition, poor nutrition and growth may have a secondary impact on body composition, bone health and brain maturation, as well as participation and health-related quality of life (HRQoL) in later childhood. In our overlapping CP-child study of growth, nutrition and physical activity (NHMRC 569605),^26^ we have determined at preschool-age the i) energy requirements, body composition, dietary intake,^27–29^ ii) validation of HPA cut-points,^30^ iii) validation of a modified 3-day weighed food record for the assessment of energy intake^29^ and iv) OPD across the spectrum of functional severity.^31 32^

Our early data on nutritional status^29^ used gold standard measures (doubly labelled water) to determine the energy requirements of preschool-aged children with CP compared with age-matched children with typical development (TD).^28^ Children who were GMFCS III–V had energy requirements 18% lower than ambulant children and 31% lower than children with TD,^28^ with no differences between ambulant children with CP and children with TD. In addition, energy intake was related to fat-free mass (FFM) index in both children with CP and children with TD.^29^ Associations were identified between OPD, energy intake and nutritional status after GMFCS level was taken into account. At preschool age, OPD was originally reported in 85% of our cohort, with a significantly greater proportion of OPD with each increase in GMFCS level.^31^ Following further testing of OPD psychometrics, with the inclusion of a typically developing reference sample, modified cut-points were developed resulting in a revised estimate of 56%.^33^ Children on full oral intakes that required modification (texture or additional energy and protein) were most at risk of poor growth and nutritional status.^31^

HPA accelerometer cut-points have been determined for sedentary and active behaviour in toddlers with CP,^34^ demonstrating that HPA levels are highly variable within GMFCS levels particularly GMFCS I–II.^35^ The musculoskeletal development of children with CP has focused on how spasticity interferes with normal muscle growth, and contributes to reduced joint range of motion, increased joint stiffness and muscle weakness.^36^ These factors lead to fixed contractures of the muscle-tendon unit and skeletal deformity that may require orthopaedic surgery.^37^ These secondary alterations progress with age^38^ and contribute to reduced gait speed, increased joint pain and falling, culminating in reduced HPA.^39^ Muscle adaptations begin early^37^ and compared with children with TD vary in the following ways: i) muscle volume is reduced^36 40 41^; ii) muscle fascicles are stiffer when passively stretched^40^; iii) muscle fascicles cannot stretch to lengths more favourable for force production^42^ and iv) the Achilles tendon is longer.^42 43^ This effectively means that the ability of muscle to generate force is reduced in children with CP. In ambulant children (GMFCS I–III), the calf muscle (gastrocnemius/soleus) has a major role in forward propulsion during walking/running^44^ and structural/functional adaptations are a cause of gait limitations.^45^ Characteristics of muscle structural/functional adaptations also vary according to unilateral/bilateral motor distribution.^46^ Lower limb treatments (casting, intramuscular Botulinum toxin A injections) aim to manage these adaptations in the preschool years; however, multilevel orthopaedic surgery is often required at functional attainment (8–11 years) according to a child’s gait profile.^47 48^ A gait profile of ambulant children (GMFCS I–III) combined with muscle properties would provide important information for surgical decision making and prediction of functional outcome. Examination and surveillance of the relationship between muscle structure/function and gait profile to functional capacity/performance, physical activity, bone health, nutritional status and healthcare costs would provide vital information for structuring management plans into later childhood.

The broad aim of the CP-child studies is to implement population-based comprehensive surveillance of children with CP from early diagnosis (at 1.5–3 years) based on brain structure and function (early gross and fine motor, growth, nutrition, HPA, musculoskeletal development) to predict comprehensive outcomes at school age (8–12 years), a time of definitive motor maturation, walking ability, need for orthopaedic intervention and educational attainment. In this extended follow-up of two previous overlapping prospective population-based CP cohorts (followed from 18 to 24 months corrected age to 5 years) across the full spectrum of functional severity (NHMRC 465128^14^; NHMRC 569605),^26^ we will re-examine the relationship to severity of brain structure at 8–12 years on diffusion MRI (dMRI in a 3.0 T MRI scanner). At 8–12 years, healthcare utilisation is likely to be different to preschool-age so that associations between health resource use and a beneficial health/social outcome can be re-evaluated. The PREDICT-CP child study (NHMRC 1077257) is prospectively registered at ACTRN: 12616001488493.

## Aims and hypotheses

The PREDICT-CP study will undertake comprehensive state-wide surveillance (in Queensland) of four birth years of a representative population-based cohort of children with CP. The relationship between brain structure on growth and physical outcomes (motor capacity, muscle and bone health, physical activity, oropharyngeal function, nutrition), cognition (executive function, educational attainment, communication) and participation (HPA, QoL, pain and sleep), will be related to educational attainment and health resource use costs.

### Hypotheses

1. The location, extent of the brain lesion(s) on semi-quantitative MRI (by 2 years) and early motor capacity and performance (1.5–3 years) will predict severity of motor capacity Gross Motor Function Measure (GMFM-66) and performance (6 min walk test (6MWT)), Paediatric Evaluation of Disability Inventory Computer Adaptive Test (PEDI-CAT) at 8–12 years.
2. The rate and limit of gross motor and fine motor development (GMFM-66, Assisting Hand Assessment (AHA), Both Hands Assessment (BoHA)), at 8–12 years will be influenced by the severity of musculoskeletal deformity (ie, slower development will correlate with increased spasticity/contracture, poor muscle function, marked hip displacement, pain, reduced sleep, reduced manual ability).
3. Cognition, executive function, communication and educational attainment will be related to brain lesion severity (location, extent of the brain lesions) on semi-quantitative MRI but not gross and fine motor capacity (GMFCS, Manual Ability Classification System (MACS)) at 8–12 years.
4. Nutritional status (under/overweight), OPD, body composition (FFM and fat mass (FM) via dual energy X-ray absorptiometry (DXA)), HPA, growth velocity and bone health will be related to the level of GMFCS attainment and will predict: i) higher healthcare utilisation and direct medical costs; ii) lower levels of participation in school, leisure and community and iii) poorer HRQoL.

### Study significance

For children with CP, this unique project will:

1. Quantify the impact of functional severity on medical resource use to inform service provision planning at school age (a period of intensive medical and orthopaedic treatments). From earlier sampling of these cohorts (NHMRC 465128/569605), we have detailed information on the content, dose and compliance, adverse events, medical, surgical and allied health resource use (interventions, medications, equipment) and consequences of outcome (from the age of 1.5 to 5 years). By study completion, we will have lifetime data on all interventions from age 1.5 to 5 years and 8–12 years, with regular assessments of their functional status/outcomes allowing predictive modelling of outcomes for children with CP.
2. Provide school-age follow-up of this comprehensively studied cohort enabling: i) prediction of outcome (brain structure and multiple outcomes); ii) prognostication on functional, cognitive, communication for school attainment; iii) risk factors for musculoskeletal problems (ie, hip, spine deformity and need for surgery) and iv) health outcomes due to sedentary behaviour, body composition, dietary intake and OPD.
3. Highlight the contribution of poor dietary intake, low levels of HPA and reduced bone health on growth, body composition and fracture risk, taking into account the severity of disability.
4. Define the relationship between HPA levels, motor capacity and muscle performance to predict eventual functional attainment and community performance.

As CP remains the most common childhood physical disability with high lifetime costs, models to predict outcomes and costs of care will inform health provision, social care and tailor data for national funding schemes such as the Australian NDIS.

## Methods

All children diagnosed with CP, born between 1 January 2006 and 31 December 2009 in Queensland will be invited to participate. These children had participated in two prospective longitudinal cohort studies between the ages of 1.5 and 5 years and will be invited to return at 8–12 years. The inclusion criteria included children with CP defined as a permanent (but not unchanging) disorder of movement and posture that resulted from an insult to the developing central nervous system. The characteristic signs were spasticity, movement disorders, muscle weakness, ataxia and rigidity.^14^ The exclusion criteria included i) children with a progressive or neurodegenerative lesion and ii) children born outside Queensland in the relevant birth years.

### Ethics approvals

There are no known health or safety risks associated with participation in any aspect of the described study. All radiological tests (including anterior-posterior (AP) pelvis, spine as required) and full body and lateral distal femur DXA for body composition and bone health have been reviewed for radiation safety. All families gave written informed consent to participate, and they were able to withdraw their child from the study at any time without explanation, without any penalty from staff at Children’s Health Queensland, or any effect on their child’s care. Data collected in this study have been stored in a coded re-identifiable form (by ID number).

### Ascertainment of the cohort

Prospective entry of birth years Queensland (born in 2006, 2007, 2008, 2009) who were entered from diagnosis commencing at 18 months and followed until school age (5 years) (n=245) in the Australian CP child study were invited to participate in the PREDICT-CP follow-up study. State-wide recruitment was established in collaboration with the Queensland Cerebral Palsy Register with data collection at tertiary referral hospitals. In cases where the diagnosis of CP was unclear, or where there was a suggestion of a progressive or degenerative course, further investigations (such as metabolic screening) were requested before a diagnosis of CP was confirmed. Children detected after 18 months of age were entered into the study at the time of diagnosis, offered brain MRI at entry and were followed up with serial motor assessments and other outcomes until 5 years.

**Figure 1:**
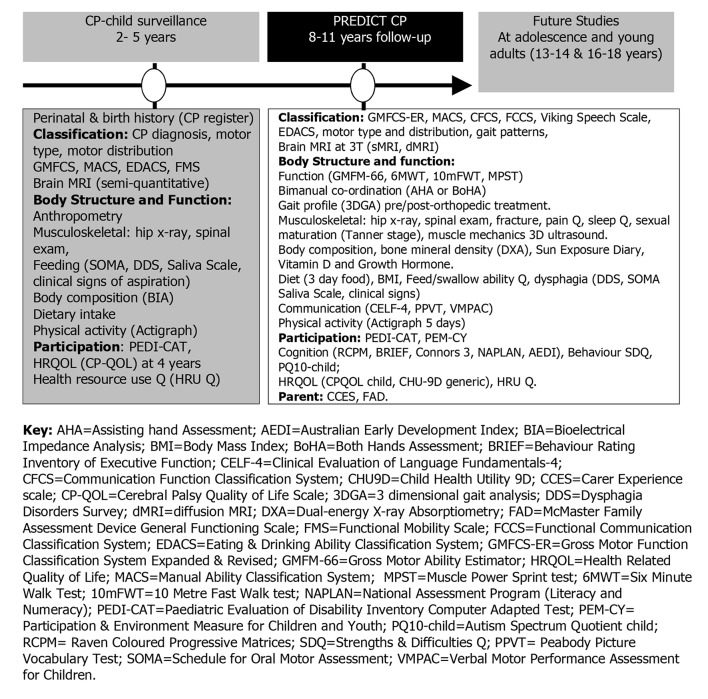
Summary of surveillance and outcome measures for the PREDICT study.

The recruited sample born in Queensland (n=245) in the birth years of 2006, 2007, 2008 and 2009 are representative of a population based sample.^49^ The sample is classified according to GMFCS for 2–18 years, a five-level classification system of children’s functional gross motor severity.^39^ It is based on self-initiated movements, antigravity postures and motor skills expected in a child aged 5 years.^50^ Children who are independently ambulant are classified as GMFCS I or II, those requiring an assistive mobility device to walk classified as GMFCS III and those in wheeled mobility as GMFCS IV and V. The recruited sample included children who were functioning at 5 years of age at GMFCS level I=96 (39.2%), II=38 (15.5%), III=38 (15.5%), IV=35 (14.3%) and V=38 (15.5%), of whom 146 were male (59.6%), of spastic motor type 208 (84.9%) and unilateral 78 (31.8%) or bilateral 165 (67.3%) motor distribution (figure 1). Children will be assessed during their 8–11 birth year at the Centre for Children’s Health Research in Brisbane. Comorbidities and need for medical management will be screened.

### Procedures

Children and families participated in previous research projects (NHMRC 569605 and NHMRC 465128) and were born in Queensland will be approached to participate in the current study. After providing informed consent, the child and their caregiver are invited to attend the Children’s Health Research Centre and the Lady Cilento Children’s Hospital, a tertiary referral centre for 1–2 days visit. All recent medical, surgical and neurological visits that had occurred since their last visit will be screened (from their medical records and by parent report) to confirm any changes in diagnosis of CP, differential diagnosis by neurological assessment (by a paediatrician, child neurologist or paediatric rehabilitation specialist). Experienced allied health researchers performed all motor, upper limb, language and cognitive assessments at the visit. Physiotherapists will check range of motion, clinical measures of spasticity, then rate the GMFCS classification, gait pattern and will measure the pelvic and spine radiographs where indicated according to standardised protocols.^23^

### Classification measures

All children with CP at all levels of ability (GMFCS I–V) at 8–12 years will be classified as mentioned below.

#### Functional severity

The GMFCS has internationally established validity, reliability and stability for the classification and prediction of motor function of children with CP aged 2–12 years.^50–52^ It has an acceptable inter-rater and intrarater (test–retest) reliability (generalisability coefficients 0.93 and 0.68, respectively).^51^ Two physiotherapists, trained in the use of the GMFCS, independently observed and classified children in one of five functional categories.^50^

Classifications of gross motor abilities change with age and therefore separate descriptions were used for different age bands. In the present study, the 6–12 years descriptions from the extended and revised GMFCS (GMFCS-ER) will be used.^53^ The GMFCS has been correlated with a number of motor scales, as well as CP motor type and distribution.^54^

#### Motor type and distribution

Motor type was classified as spastic, dystonic, ataxic, hypotonic, choreoathetosis, mixed CP or unclassifiable according to Surveillance of Cerebral Palsy in Europe guidelines.^55^ Distribution was classified by number of limbs impaired, unilateral and bilateral distribution (hemiplegia, diplegia, triplegia, quadriplegia) by at least two independent raters. The Dyskinesia Impairment Scale^56^ will be undertaken for those participants with a motor-type (primary or secondary) diagnosis of dystonia and/or choreoathetosis. This is an important assessment to measure the motor capacity and function of children with these particular motor types.^57^

#### Functional performance

The Functional Mobility Scale^58^ at 5 m (home), 50 m (school) and 500 m (community)^58^ will be used to evaluate functional performance. This is a valid and reliable measure of a child’s usual walking ability at three distances (5 m, 50 m and 500 m), representing their home, school and wider community, respectively.^59^

#### Gait pattern

Gait patterns will be classified according to the classification by Rodda and Graham,^60^ which demonstrated validity and reliability.^61^ Gait patterns for bilateral ambulant CP will be classified as either: i) true equinus, ii) jump knee, iii) apparent equinus or iv) crouch gait. For children with unilateral CP, gait patterns were classified according to the classification by Winters *et al*.^48^ This classification considered the sagittal plane joint movements: i) type I—foot drop during swing phase (apparent equinus); ii) type II—persistent ankle plantarflexion (true equinus); iii) type III—maintained plantar flexion through gait cycle plus limited knee flexion-extension and iv) type IV—similar to III, plus reduced hip flexion-extension. The classification by Winters *et al* had good inter-rater reliability using written reports (weighted kappa, wκ=0.76) and videos (wκ=0.63).^61–63^

#### Upper limb function

Upper limb function is classified using the MACS.^64^ The MACS is an international system to classify hand function based on the child’s typical performance when handling objects in daily activities. The MACS is a five-level classification of how well children with CP use their hands to handle objects in day-to-day activities.^64^ This classification system was developed for children aged 4–18 years, and has good reliability for use in children as young as 2 years.^64^ The MACS has reported construct validity, and excellent inter-rater reliability (intraclass correlation coefficient=0.97 between therapists and 0.96 between therapists and parents) for children with CP.^65^ Children will be classified using the MACS by an occupational therapist in discussion with the child’s carer.

#### Communication function

Communication function will be classified on three distinct but overlapping systems:

1. Communication Function Classification System classifies children’s performance in sending and receiving communicative messages using their typical communication means (considering all communication methods including Augmentative and Alternative Communication). It has been validated in children with CP aged 2–18 years. Reliability between professionals was moderate (κ=0.66), professional-parent fair (κ=0.49) and test–retest strong (κ=0.82).^66^
2. Functional Communication Classification System classifies children’s performance only in sending communicative messages, and also considers their typical communication (including all communication methods including Augmentative and Alternative Communication). It has excellent inter-rater reliability between professionals (κ=0.94) and parent-professional (κ=0.59).^67^
3. The Viking Speech Scale (VSS) was used to classify children’s speech production.^68^ The VSS is a four-level classification system, which can be used to classify speech intelligibility for strangers and unfamiliar conversation partners of children with CP aged 4 years and above. It has strong content validity, and moderate-to-substantial inter-rater reliability between pairings of speech pathologists, healthcare professionals and parents (κ=0.58–0.81).^68^

#### Eating and drinking function

The Eating and Drinking Ability Classification System (EDACS) classifies the eating and drinking abilities of children with CP aged 3 years and above. Classification is I–V and describes children’s safety and efficiency predominately focusing on food and fluid textures.^69^ The EDACS has strong inter-rater reliability between professionals (ICC=0.93), but fair reliability between parings of professionals and parents (ICC=0.45).^69^

### Primary outcomes

This prospective longitudinal study follow-up has two primary outcomes (hypothesis 1):

1. Gross motor function will be evaluated using the GMFM-66;
2. Brain lesion severity will be assessed using a structured scoring proforma (Fiori scale).

All additional measures are secondary outcome measures (hypotheses 2–4).

### Body structure and function measures

#### Brain structure on MRI

The American Academy of Neurology practice parameter has recommended that brain MRI should be part of the diagnosis of CP.^70^ Early MRI at 0–3 years was classified according to the nature and presumed timing of the lesion^11^ and analysed for brain lesion severity on the semi-quantitative scale of Fiori.^8^ Aetiology of CP was evaluated using MRI (location, nature and structure of the brain lesion).^11^ The brain lesion was classified by three main criteria:

1. The *anatomical features* of the lesion:
2. localisation by tissue (eg, cortical, white matter, deep grey matter, etc)
3. localisation by region (eg, lobes involved, laterality, etc)
4. extent of lesion (eg, generalised, hemispheric, lobar, etc)
5. The presumed *aetiology* of the lesion: i) genetic; ii) ischaemic; iii) infective and iv) other.
6. The presumed *timing* of the insult that caused the lesion:
7. Prenatal by trimester or by stage of brain development;
8. Perinatal;
9. Postnatal.

All MRIs were classified by a neurologist (SF) together with a neuroradiologist (AC) using a standardised method of image evaluation and classification. Following these evaluations, consensus was reached regarding the above three criteria. Based on preliminary data, it was estimated that >60% of children currently receiving a diagnosis of CP had early brain MRI as part of their clinical workup. All children (n=245) will be offered a repeat brain MRI at 8–12 years at 3 T. The majority will have their imaging performed and reported through the Herston Imaging Research Facility, on a Siemens 3.0 T MR scanner. The current minimum imaging protocol for patients with suspected CP consists of axial fast spin echo and coronal fast spin echo sequences and three-dimensional (3D) inversion prepared fast spoiled GRASS sequence. The 3D acquisitions will be reformatted in axial, coronal and sagittal planes, with additional oblique and curved reformatting. Age-specific protocols will be used to maximise the ability to detect cortical and white matter abnormalities at different stages of myelination. All neuroimagings will be reviewed by a neurologist (SF, AG) and a neuroradiologist (AC) familiar with the features of lesions that resulted in CP. This approach is consistent with a clinical practice guideline suggesting that all patients with the label of CP had high-quality MRI on at least one occasion.^70^ MRI scans will be performed predominantly awake, without anaesthesia and after informed consent. Preparation for the MRI will be offered to families in the form of a training DVD explaining the scanner experience and practice in a ‘mock scanner’ (0.0 T) where required.

Brain lesion severity will be assessed using a structured scoring proforma^8^ based on the CH2 template,^71^ a highly detailed single-subject T1 template in Montreal Neurological Institute MNI space, which is the international standard for brain mapping (International Consortium of Brain Mapping). Lesions will be transcribed onto the proforma and the following measures obtained: i) number of anatomical lobes involved, ii) number of slices on the template that were affected and iii) size and distribution of the lesion measured by a global lesion score and lesion subscores. The score (maximum of 40) is based on: i) anatomical lobes involved; ii) number of affected slices and iii) size and distribution of the lesion. The number of lobes and slices affected will be the average of summed right and left hemispheres. To calculate total lesion score, each frontal, parietal, temporal and occipital lobes will be first considered in three sections: periventricular, middle and subcortical matter. Each section scored 0.5 if <50% of area is involved; or 1, for >50% involvement, with a maximum lobar score of 3. Lobar scores for each hemisphere are summed, with a maximum hemispherical score of 12. The total lesion score is the sum of right and left hemispherical scores (maximum score of 24). A 1-point score (involved/not involved) wis also attributed to 16 anatomical structures including the corpus callosum, the cerebellum and the main subcortical structures. The final maximum score of the scale is, thus, a maximum of 40 (24+16).^72^ The Fiori scale method has strong inter-rater and intrarater reliability^72^ and strong construct validity based on dMRI and functional severity in children with unilateral CP.^73^

At 8–12 years, structural MRI (sMRI)-guided and functional MRI-guided dMRI scans suitable for connectivity analyses will be undertaken on the 3T scanner at Herston Imaging Research Facility (or The Lady Cilento Children’s Hospital at 3T for children requiring general anaesthesia ≈ 5%). dMRI for white matter fibre tracking and whole brain connectomes will be acquired using our published protocol.^74^ The sMRI images will be acquired using an Magnetisation Prepared Rapid Acquistion Gradient Echo(MPRAGE) sequence at an isotropic resolution of 1 mm. dMRI data will be preprocessed to reduce image artefacts,^75^ and the fibre orientation distribution was estimated using constrained spherical deconvolution.^76^ Probabilistic tractography will be conducted using MRtrix software and connectivity matrices generated using previously described methods.^74^ Quantitative diffusivity indices Fractional Anisotrophy (FA) and Mean Diffusivity (MD) will be encoded within the connectome to assess reorganisation.^74^ Network-based statistics^77^ will be performed between FA and MD connectomes to identify significant cortical networks associated with neural reorganisation. A second analysis will investigate brain maturation by comparing serial sMRI data acquired around 2 years with scans in the same children at 8–12 years to develop a predictive model of brain structure and functional outcome using spatiotemporal analysis of the longitudinal imaging data.^77^

### Clinical history and examination

Clinical history will be reviewed (see online supplementary Appendix 1: Queensland CP Child Physicians Checklist) to determine:

- Presence or absence of comorbidities including vision impairment, hearing difficulties, epilepsy;
- Feeding issues including presence or absence of gastrostomy tube and failure to thrive;
- Respiratory difficulties including episodes of pneumonia and aspiration.

10.1136/bmjopen-2016-014950.supp1 Supplementary Appendix 1

A comprehensive musculoskeletal examination will be performed by a physiotherapist to record data relating to joint range of movement, leg length difference, bony anomalies, motor type and lower limb muscle spasticity and contracture.^78–83^

### Anthropometry

Anthropometric measures will be collected as described in detail in our published growth, nutrition and physical activity protocol,^26^ including the following:

- Body mass to the nearest 100 g using chair scales (Seca).
- Height to the last completed millimetre with a stadiometer, or, length using a supine measuring board. Where a direct measure of height or length could not be obtained, height will be estimated from knee height or upper arm length using published validated techniques and formulas.^84^
- Body mass index will be calculated as mass (kg) divided by height (m) squared.
- Growth and growth velocity (Z*-*scores of measured or predicted height).
  Weight and body mass index Z-scores will be calculated for age and sex according to the Centres for Disease Control and Prevention 2000 growth data.^85^

#### Gross motor function

Gross motor function will be evaluated using the GMFM-66 and GMFM-88^86^ by experienced research physiotherapists. The GMFM-88 assessed a child’s motor abilities in lying to rolling, sitting, crawling to kneeling, standing, walking, running and jumping. The GMFM-66 comprised a subset of the 88 items identified (through Rasch analysis) as contributing to the measure of gross motor function in children with CP. The GMFM-66 will provide an overall measure of gross motor function and the GMFM-88 provides domain scores to explore specific motor skills.^86^

#### Upper limb performance

Children with unilateral CP whose manual ability is MACS I–III will be assessed on the school kids AHA, a Rasch measure of effectiveness of impaired hand in bimanual activities. Test–retest reliability was high (ICC 0.98) and there was predictive validity of future assisting hand use.^87^ BoHA will be used for children with bilateral CP who were manual ability MACS I–IV. The BoHA test content was developed by researchers in Norway and Sweden through modification of the AHA test items and by generation of new items.^88^ Associations between BoHA measures and MACS levels showed strong correlation (Spearman’s rho: 0.74). The person separation ratios (4.36 and 5.19) and the person reliability (0.95 and 0.96) for the subscales indicated that the children’s hand function could be separated into six and seven ability levels.^88^ BoHA is the first observation-based assessment of effective use of the hands in bimanual activities for children with bilateral CP.

Hand dominance will be assessed using the Edinburgh Handedness Inventory laterality quotient.^89^ The Edinburgh Handedness Inventory questionnaire consists of 10 items regarding hand preference (right or left) in performing a number of everyday tasks requiring one (eg, writing, drawing, throwing and using scissors) or two hands (eg, using a broom or opening a box). The laterality quotient is calculated using the following formula: laterality quotient = (right hand–left hand/ (right hand+left hand)×100). The Edinburgh Handedness Inventory is included to objectively determine upper limb dominance. This classification system consists of a table that requires the participants to indicate which hand they use to perform a selection of everyday tasks.

Stereognosis relates to a participant’s ability to perceive and recognise objects by using only tactile information^90 91^ and will be assessed on the impaired and unimpaired limbs, using the approach described by Sakzewski *et al*.^91^ Participants will be required to identify objects placed in their hand, without any visual cues. A total of nine objects will be placed in the hand one at a time. Three familiar objects (teaspoon, key, peg) and six similar matched objects (safety pin and paperclip; pen and pencil; coin and button) will be used. With vision occluded, participants are presented with each item. If a participant is unable to grasp, manipulate or release an object, the occupational therapist assisted the participant and will move the object for them within their hand. A corresponding set of items will be used to allow participants to identify the object in order to minimise any errors due to incorrect naming of the object. Scores ranged on a scale from 0 to 9, where participants scoring below 9 are considered to have impaired stereognosis.^90 91^

#### Radiological measures of hip displacement and spine

Hip surveillance, including AP pelvis X-ray, is recommended for all Australian children with CP to facilitate early detection and treatment of severe or progressive hip displacement.^19 92 93^ The migration percentage is widely accepted as the gold standard measure in hip surveillance,^78 94^ measuring femoral head displacement.^95^ Other measures included the Acetabular Index, assessing acetabular dysplasia,^96^ the Hilgenreiner's epiphyseal angle (HEA)^96^ and the femoral neck-shaft angle.^95 97^ The HEA^96^ is a radiographic measure describing the proximal femoral epiphysis and has been previously applied to assessment of coxa valga,^98 99^ but may offer prognostic information for hips at risk in CP. The HEA represents the acute angle between a line drawn parallel to and through the proximal femoral epiphysis and Hilgenreiner’s line.^81 82^ Physiotherapists will perform a clinical examination of spinal alignment and mobility to screen for evidence of a potential scoliosis or kyphosis. Where indicated, an AP spine radiograph (for scoliosis) or lateral (for kyphosis) will be performed. Spines where scoliosis was evident will be measured according to the Cobb angle.^100^

#### Body composition and bone health

Body composition measures and bone parameters will be acquired using a Lunar Prodigy DXA (GE Medical Systems, LUNAR, Madison, Wisconsin, USA). Body composition measures included FM (g) and FFM (g). Bone parameters include areal bone mineral density (g/cm^2^) and bone mineral content (g) for all total body, bilateral proximal and lateral distal femur sites. The lateral distal femur is a common site of fracture,^101 102^ with the technique previously described,^102^ and measurements are reproducible in children with CP.^103 104^ The analysis involves creating three regions of interest, each containing different proportions of trabecular and cortical bone with results for each region of interest (ROI), therefore, treated independently.^101 102^ Additionally, the proximal and distal femoral sites are used to calculate bone mineral apparent density (g/cm^3^), derived from the projected bone area (cm^2^) to provide an approximation of volumetric BMD.^105^ All scans in this research are a ‘one-off’ occurrence, with the total radiation dose for these five DXA scans being <15 μSv.^106^ This is equivalent to approximately 1–2 days natural background radiation exposure, and only equivalent to 3% of the dose constraint limit for children as research volunteers, up to the age of 18 years.^106^ The total estimated time for all DXA scans is 30 min, performed at the University of Queensland Children’s Nutrition Research Centre.

#### Fracture rate

Fractures will be diagnosed radiologically. Parents will report by telephone within 24 hours of fracture occurrence and will bring X-ray films and details of management to their study visit. Vertebral fracture will be diagnosed on lateral X-rays of the thoracic and lumbar spine when indicated. Children who were GMFCS III–V will undergo thoracic and lumbar spine (AP/lateral) at 8–12 years if there were clinical signs of fracture and/or scoliosis/kyphosis. Radiographs are minimised to reduce the radiation exposure.

#### Sexual maturation

Legal guardians of participants will be provided with standardised Tanner stage puberty diagrams, and parents will be asked to evaluate the child’s current pubertal stage.^107^ Parental pubertal assessment will be reviewed by a physician for precocious puberty. In cases of precocious puberty, a left hand/ wrist X-ray will be conducted to determine the bone age and relative skeletal maturity of children. The bone age will be used to determine if the CP condition is interfering with the proper growth and bone development of the child.

#### Pain

Children will complete the Paediatric Pain Questionnaire (PPQ) with adult help if required.^108^ The PPQ asks children to report their pain now (severity, type and location), as well as the severity of the worst pain they had in the previous week. The PPQ’s visual analogue scale for pain rating provides a valid and stable measure of pain intensity in children and adolescents with chronic musculoskeletal pain.^109^

#### Three-dimensional gait analysis and in vivo muscle mechanics

A full 3D gait analysis, including synchronised measurement of muscle activation using electromyography (EMG) and calf muscle mechanics using two-dimensional (2D) ultrasound, will be performed for all children functioning at GMFCS I–III. Participants will walk unaided and barefoot at a self-selected speed over a level walkway (10 m in length) with four force platforms embedded in the laboratory floor in the centre of the walkway. Reflective markers will be attached to the trunk, pelvis and lower limbs according to the modified ‘Plug in Gait’ marker set, with additional clusters of three markers on each thigh and shank segment, and a marker on the fifth metatarsal head.^110^ Marker trajectories will be recorded at 100 Hz using an 10-camera, 3D motion capture system (Vicon Motion Systems, Oxford, UK) and ground reaction force data will be acquired at 1 kHz using four 510 mm×465 mm force platforms (AMTI, Watertown, Massachusetts, USA) arranged in series. Lower limb muscle activations of the rectus femoris (RF), medial hamstrings (MH), medial gastrocnemius (MG), lateral gastrocnemius (LG), soleus (SOL) and tibialis anterior (TA) is recorded for both legs at 1 kHz using a wireless surface EMG system (Aurion ZeroWire, Milan, Italy). Raw EMG signals will be high-pass filtered (Butterworth, zero-lag, fourth order, 30 Hz) to remove movement artefact, full wave rectified and low passed filtered (Butterworth, zero-lag, fourth order, 6 Hz), and interpolated to 101 points per cycle. Non-negative matrix factorisation will be applied to extract muscle synergies,^111^ which represented neuromuscular control during gait. Whole body 3D gait kinematics, joint moments at ankle, knee and hip joints and musculotendinous lengths for MG, LG, SOL, RF and MH will be computed across at least five trials using OpenSim^112^ and normalised to length in standing. The Gait Profile Score^113^ will be calculated as an index of overall gait pathology. A digital output signal from the ultrasound system will be used to synchronise acquisition of all 3D marker, force plate and EMG data.

Two-dimensional B-mode ultrasound will be used to examine MG and SOL muscle function during walking by attaching a flat ultrasound transducer (LV7.5/65/64D, Telemed Echo Blaster 64 EXT-1T, Vilnius, Lithuania) to the surface of the skin above the MG muscle and recording muscle fascicle length and pennation angle changes, as described previously.^114^ Muscle fascicle behaviour during walking will be analysed using a semi-automatic process, which has been shown to be highly repeatable (coefficient of multiple correlation 0.88).^115^ The average of five complete strides will be used in the analysis for each participant to ensure the overall reliability of muscle fascicle length data.^116^ Freehand 3D ultrasound will be used to measure muscle size and structure of the lower leg muscles: MG, LG, SOL and TA.^117^ This method of 3D ultrasound is valid (within 1.3%) and reliable (ICC >0.99) for measuring gastrocnemius muscle volume and length in vivo.^117^ Calf muscle physiological cross-sectional area will be measured as the ratio of muscle volume muscle fascicle length, corrected for fascicle pennation angle.

### Activity limitations

The following measures of activity limitations for functional capacity will be performed for ambulant children (GMFCS I–III) at 8–11 years (≈ n=172).

#### 6 min walk test

This simple, submaximal test measures the distance walked over 6 min, providing information about endurance during functional activities.^118^ The 6MWT has excellent test–retest reliability (ICC=0.98) in CP.^119^ Percentile curves have been created on 1445 children with TD aged 7–16 years.^120^ The test will be performed according to guidelines of the American Thoracic Society on a 10 m course.^121^

#### Muscle power sprint test

The muscle power sprint test (MPST) provides an estimate of anaerobic power.^122^ The MPST requires participants to complete six 15 m runs as fast as possible with 10 s rest between each lap. Power output is calculated as the product of body mass and distance, divided by time.^122^ The MPST has been validated against the Wingate Anaerobic cycling test,^123^ and has excellent test–retest reliability (ICC 0.98) in children with CP.^122^

#### 10 m fast walk test

The 10 m fast walk test (10 mFWT) is a test of maximal walking speed over a distance considered the minimum for functional ambulation. The 10 mFWT has moderate test–retest reliability in children with CP (ICC 0.81).^124^

#### Lower limb functional strength

Thirty second repetition maximum (rep_max_) of functional strength exercises (including sit-to-stand, lateral step-ups and half-kneel to stand) will be tested according to published recommendations.^125^ Functional strength tests demonstrate acceptable inter-tester reliability (ICC≥0.91; coefficient of variation (CV) 12.1%–22.7%) in children with CP.^125^ For each lower limb functional strength exercise, participants will be given verbal and visual instructions as well as two practice repetitions prior to testing. The exercises were assessed in the following order: sit-to-stand, lateral step-up dominant leg, lateral step-up non-dominant leg, half-kneel to stand dominant and half-kneel to stand non-dominant. Participants will be given verbal encouragement throughout. Participants will be given 180 s rests between exercises. If a participant does not complete an exercise while performing the practice attempts, they will be assigned a score of 0 and will not proceed to testing.

#### Habitual physical activity

Triaxial accelerometers (ActiGraph GT3X+, Pensacola, Florida, USA) will be used to evaluate the frequency, intensity and duration of physical activity.^126^ ActiGraph accelerometers have evidence of validity and interinstrument reliability in children with TD compared with heart rate monitoring, direct observation, indirect calorimetry, whole-room calorimetry and doubly labelled water.^126^ The ActiGraph has been validated for measurement of physical activity intensity in adolescents with CP using oxygen uptake as the criterion measure.^127 128^ ActiGraphs will be fitted during assessment and worn during waking hours for 7 days.^126^ Participants’ caregivers completed a 7-day physical activity monitor log to record wear and non-wear times (see online supplementary Appendix 2). Stored data will be uploaded to an excel macro to determine daily wear time, average counts per min, daily time spent in sedentary, light, moderate and vigorous activity. Counts will be classified using established cut-points for children with CP.^128^

#### Blood samples for growth hormone and vitamin D

Blood will be collected and tested for hormones and other markers required for optimal growth, bone and metabolic health. Specifically, these tests are liver function, kidney function, full blood count, insulin-like growth factor-1, thyroid hormone, parathyroid hormone, vitamin D3, calcium, phosphate and iron studies. As described above, these parameters of growth, bone health and body composition are often altered in children with CP and are related to gross motor function classification, body composition, growth velocity and nutritional status. Blood tests are optional and consent will be obtained from the parent and assent from the child where possible. Blood samples will be collected by qualified phlebotomists, who are familiar with collecting blood from paediatric subjects using their standard procedures. Where preferred, samples will be collected under general anaesthesia, if a patient undergoes an unrelated and non-emergency surgical procedure (eg, orthopaedic surgery, Botulinum toxin A injections, MRI under anaesthesia). Parents of participants will be advised if these results fall outside the relevant reference ranges in relation to age, gender and pubertal status. Parents will provide informed consent for information to be provided to their treating clinician who will take responsibility for ongoing care and follow-up.

#### Dietary intake

Dietary energy intake will be recorded using a 3-day weighed food record as validated^29^ using our published methods.^26^ Food records will be analysed using FoodWorks. Mean energy intake will be expressed as megajoules per day and as a percentage of age-specific and gender-specific recommendations.^129^

#### Vitamin D intake

A food frequency questionnaire will be completed by parents/caregivers to determine the habitual intake of vitamin D-containing foods by the participants. The questionnaire consists of a table that requires parents to tick a frequency box and record the brand of a simple list of foods.^130^

#### Sun exposure

A 7 day sun exposure diary will measure daily sun exposure in the participants to measure ultraviolet radiation (UV) exposure for vitamin D adequacy. Each day, participants will record the amount of time spent in the sun during each 1 hour interval (0, <15, 15 to <30, 30 to <45 or 45–60 min) between 05:00 and 19:00 hours. Clothing cover (based on a clothing cover guide provided with the dairy) and use of sunscreen (frequency and application site) using established methodology will also recorded^131^ (see online supplementary Appendix 3). It is proposed that the sun exposure diary is done at the same time as the physical activity monitor record to lessen the burden on the participants. Sun exposure diaries will be done within 2 weeks of serum vitamin D levels being collected, to allow for meaningful interpretation of sun exposure and vitamin D levels.

#### Oropharyngeal dysphagia

OPD (feeding and swallowing difficulties) will be evaluated during a digital video-recorded snack of 20 min. Children will be presented with three standardised boluses of five textures; puree, semi-solid, chewable, tough chewable and fluid. The following measures will be used to rate the mealtime:

1. The Dysphagia Disorders Survey (DDS)—part 2 consists of a series of binary judgements on eight ingestion functions across the oral preparatory, oral, pharyngeal and gastro-oesophageal phases (maximum raw score of 22). The DDS has good reliability^132 133^ and convergent validity.^132–136^
2. The Schedule for Oral Motor Assessment (SOMA) consists of seven oral motor challenge categories corresponding to four food textures and three fluid utensils. The SOMA has been validated on 127 young infants; 58 comparison children with typical oral skills, 56 with non-organic failure to thrive (aged 8–24 months) and 13 children with CP and overt feeding difficulties (aged up to 42 months).^137^ It has strong inter-rater reliability (κ=1.0 in 68% of fluid category items and 58% of food category items) and test–retest reliability between boluses (κ=1.0 in 84% of items).^137^
3. Observations of 16 clinical signs suggestive of pharyngeal phase impairment (eg, cough, gurgly phonation, wet respiration) will be rated premealtime and postmealtime by a trained researcher, and rated according to each food/fluid texture from video by a speech pathologist.^138–140^
4. The Thomas-Stonell and Greenberg scale will be used premealtime and postmealtime to rate saliva loss.^141^ This consisted of two observational ordinal scales (1–5), based on severity and frequency of loss.
5. The Cerebral Palsy Child Feeding Questionnaire, used in the CP-Child Study^142^ gathered information on the child’s typical mealtime performance based on parent report, which supplemented the data obtained from the participant’s clinical feeding assessment.
6. The Feeding/Swallowing Impact Scale (FS-IS) addresses questions of carer QoL and how to incorporated into the economic evaluation. The FS-IS is a validated tool to measure the impact of caring for a child with dysphagia and concerns on caregiver QoL.^143^ It is an 18-item, parent questionnaire divided into three subsections: i) daily activities; ii) worry and iii) feeding difficulties. The tool was validated on the caregivers of 164 children (median age 14 months, mean: 32±44 months) with varying comorbidities including prematurity (born <37 weeks) in 66 (40%) children, 144 (88%) were medically complex with conditions in more than one diagnostic-based category and 77 (47%) of children had feeding tubes.^143^

#### Communication

All children will have language assessed using the core language subtests of the Clinical Evaluation of Language Fundamentals screener (CELF-4),^144^ in addition to the Peabody Picture Vocabulary Test (PPVT). The CELF-4 is a criterion referenced assessment of language skills in children aged 5–21 years, with Australian norms available. Children who were non-verbal only will complete the receptive subtests of the CELF-4 and the PPVT. Children unable to participate in standardised assessment (eg, due to significant cognitive, visual or motor limitations) will have language classified using the Triple C, a parent-reported observational assessment. Communication performance will be indicated by parents on a communication questionnaire developed for this study (see online supplementary Appendix 4), which includes information on augmentative and alternative communication system type, use, frequency and access.^144^

Speech production will be assessed using the verbal motor production assessment for children (VMPAC). The VMPAC^145^ is a diagnostic tool for the systematic assessment of neuromotor integrity of the motor speech system validated in 1434 children aged 3–12 years^146^. The following subtests of the VMPAC will be administered:

1. Oromotor production in word sequences and sentences (six items): items consisted of three-word and four-word sequences and five-word sentences. These items are designed to evaluate the child’s ability to sequence oromotor movements across different plane within a linguistic context.
2. Oromotor production in connected speech and language (five items): this subtest assesses the child’s motor control (eg, motor precision) as it varied in the context of higher level language formulation.
3. Oromotor production in automatic verbal sequences (one item): this subtest allows evaluation of speech characteristics including pitch, resonance, vocal quality, loudness, prosody, intonation and rate during production of an automatic speech task (eg, counting to 10, saying the alphabet).
4. Oromotor production in word sequences and sentences (six items): items consisted of three-word and four-word sequences and five-word sentences. These items are designed to evaluate the child’s ability to sequence oromotor movements across different plane within a linguistic context. Oromotor production in connected speech and language (five items): this subtest assesses the child’s motor control (eg, motor precision) as it varied in the context of higher level language formulation.
5. Oromotor production in automatic verbal sequences (one item): this subtest allows evaluation of speech characteristics including pitch, resonance, vocal quality, loudness, prosody, intonation and rate during production of an automatic speech task (eg, counting to 10, saying the alphabet).

#### Paediatric evaluation of disability inventory computer adapted test

Performance of self-care will be evaluated using the parent-reported PEDI-CAT for the domains of self-care, mobility and social functioning using scaled scores (Rasch), which have good validity and reliability.^147^ The PEDI-CAT was developed on the basis of the original PEDI.^148^ Scaled scores (possible range 20–80) for each domain provide an indication of the child’s performance along a continuum of item difficulty and are most suitable for research.^147^ Scaled scores give more precise results in the extreme ranges than normative standard scores.^147^ Scaled scores are recommended to track functional progress in children who are substantially delayed.^147^

### Participation and environmental measures

#### Participation and environment measure for children and youth

The participation and environment measure for children and youth (PEM-CY) is a parent-reported instrument that examines participation and environment across three settings: i) home; ii) school and iii) community.^149^ There are 10 items in the home section, 5 in the school section and 10 in the community setting. For each item, the parent is asked to identify how frequently (over the past 4 months) the child has participated (eight options: daily to never); how involved the child typically is while participating (five-point scale: very involved to minimally involved) and whether the parent would like to see the child’s participation in this type of activity change (no or yes, with five options for the type of change desired). For each setting, the parent is then asked to report on whether certain features of the environment make it easier or harder for the child to participate. The PEM-CY has reported moderate-to-good internal consistency (0.59 and above) and test–retest reliability (0.58 and above) in a population of children (aged 5–17 years) with and without disabilities residing in the USA and Canada (n=576).^149^ The PEM-CY will be collected using either a paper or online questionnaire format to gain an understanding of the participation of children and adolescents and the impact of environmental barriers and facilitators.

#### Strengths and Difficulties Questionnaire

The Strengths and Difficulties Questionnaire^150 151^ is a 33-item parent-rated questionnaire that is used to assess parents’ perceptions of prosocial and difficult behaviours in their child or child adjustment. Parents responded to 25 questions about their child’s behaviour in the last 6 months using a three-point Likert scale (ie, ‘0’=not true to ‘2’=certainly true). These 25 questions are combined to create five subscales of: frequency of emotional symptoms; conduct problems; inattention/hyperactivity; peer problems and prosocial behaviour (eg, ‘considerate of other people’s feelings’). A total score for each scale (0–10) and overall total difficulties score (0–40) is calculated, with higher scores indicating more distress on all scales except prosocial behaviour. Scores of 17 or above for the total difficulties scale are used as a clinical cut-off point. Scores from the five subscales and the overall difficulties scale will be used as a measure of the child’s psychological functioning. The overall total difficulties score has been demonstrated to have moderate-to-high internal consistency (Cronbach's α=0.73–0.82) and test–retest reliability (r=0.77–0.85).^152^

#### School attainment

The Australian Early Development Index is a population measure of development at school entry, assessing school readiness.^153^ The National Assessment Program—Literacy and Numeracy (NAPLAN) measures literacy/numeracy achievement and individual reports will be obtained from families as a measure of early school achievement.^154^ Where applicable, consent will be obtained from parents to the release of NAPLAN result by the Queensland Curriculum and Assessment Authority.

### Cognition

#### Raven’s coloured progressive matrices

The Raven’s coloured progressive matrices (RCPM) is an assessment of non-verbal intelligence for children aged 5–11 years with intellectual delay or physical disability. It consists of 36 items (15–30 min) of increasing difficulty in which the child has to complete a pattern. RCPM has validity with CP and Australian norms.^155 156^ In 618 Australian children (aged 6–11 years), the RCPM had good internal consistency (0.76–0.88) and split-half reliability (0.81–0.90).^156^

#### Behaviour rating inventory of executive function

The behaviour rating inventory of executive function is a parent completed 86-item measure of executive function in their child’s everyday life, yielding two scores: i) the behavioural regulation index and ii) the metacognition index to form a global executive composite score. Both scores can be converted into T scores with ≥65 indicative of dysfunction.^157^ It has good convergent and divergent validity with Child Behaviour Checklist and the Behaviour Assessment System for Children,^158^ high internal consistency, (Cronbach’s α=0.80–0.98) for parent form.^158 159^

### Attention and executive functioning

#### Conners three parent short form (Conners 3)

The Conners three parent short form (Conners 3)^160^ is a thorough assessment of attention deficit hyperactivity disorder (ADHD) and its most common comorbid problems and disorders in children and adolescents aged 6–18 years. The Conners 3 will be completed by the participant’s parents or guardian and consisted of 110 statements and takes approximately 20 mins to complete. Parents or guardians rate each statement using a four-point scale ranging from ‘0, not true at all (never, seldom)’ to ‘3, very much true (very often, very frequently)’. The Conners 3 measures the seven key areas of inattention, learning problems, aggression, family relations, hyperactivity/impulsivity, executive functioning and peer relations. Raw scores are converted into T scores based on a large representative normative sample based on the US consensus data. In addition, the Conners 3 calculates T scores for symptom scales including ADHD hyperactive/Impulsive, ADHD combined, oppositional defiant disorder, ADHD inattentive and conduct disorder. Both internal consistency coefficients (α=0.83–0.94) and test–retest reliability (r=0.52–0.94) were good for the Conners 3 parent version total sample age range.^160^

#### The autism spectrum quotient-child

The autism spectrum quotient-child (AQ10-child) is a 10-item parent-reported autism screening measure for children aged 4–11 years.^161^ It was developed from the Quantitative Checklist for Autism in Toddlers. Parents respond to the 10 items on a four-point Likert scale from definitely agree to definitely disagree. Items will be summed and the scale had a cut-off of 6. It had high internal consistency (Cronbach’s α=0.90), sensitivity (0.95) and specificity (0.89).^161^

#### Sleep Disturbance Scale for Children

Sleep disorders are up to four times more common in children with CP compared with the general population and are linked to both physical (total body involvement, severe visual impairment) and environmental factors (single-parent household, bed-sharing). The most commonly reported disorders include difficulty initiating and maintaining sleep, sleep-wake transitions and sleep breathing disorders.^162^ The Sleep Disturbance Scale for Children is a 27-item parent-reported questionnaire that assesses sleep disturbance in children within the past 6 months.^163^ Each item is responded to on a five-item Likert scale with higher values representing greater clinical severity. Items are summed to produce a total score and six subscores representing different facets of sleep disturbance: disorders of initiating and maintaining sleep, sleep breathing disorders, disorders of arousal, sleep-wake transition disorders, disorders of excessive somnolence and sleep hyperhidrosis. It has good internal consistency (Cronbach’s α=0.71–0.79), test–retest reliability (r=0.71) and discriminative validity in distinguishing between clinical and community samples.^163^

### QoL measures

#### Condition-specific QoL measures

The cerebral palsy quality of life-child assesses well-being across multiple domains using parent-report (aged 4–12 years) and child self-report from 9 years.^164–166^ Psychometrics are excellent with Cronbach’s α 0.74–0.92 parent-proxy report and 0.80–0.90 child self-report. Test–retest was adequate (ICC 0.76–0.89) and moderately correlated with health (r=0.30–0.51).^164–166^

#### Generic QOL

The Child Health Utility-9D is a generic instrument for children aged 7–11 years for which there is an algorithm to give a single preference-based utility index for health states (giving a single generic preference-based indicator of each individual’s health state), making the data amenable for economic evaluations for interventions.^167–170^ The EuroQoL five-dimensional descriptive system (EQ-5D-5L) is a generic instrument designed to describe and value health based on five dimensions: mobility, self-care, usual activities, pain/discomfort and anxiety/depression.^171^ The EQ-5D-5L will be completed by the parent/caregiver, about themselves and scored using the Australian algorithm.^172 173^ This is relevant (alongside the CES) to address questions such as carers QoL and how to incorporate it into economic evaluation.

#### Demographic questionnaire

Parents will be required to complete questionnaires related to various aspects of their child's development/progress including participation, feeding, HRQoL, sleep, fracture history, pain, executive functioning in everyday life, clinical comorbidities including epilepsy, psychological functioning, treatments and interventions the child has received since the completion of the CP-child projects (see online supplementary Appendix 5). The socioeconomic status of Australian families will be classified into tertiles using scores on the Socioeconomic Indexes for Areas Index of Relative Disadvantage.^174^

### Parent questionnaires

#### Carer Experience Scale

The Carer Experience Scale (CES) will be completed by primary caregivers at their study visit. This validated measure of care-related QoL has six domains (activities, support, assistance, fulfilment, control and relationship with the care recipient) and takes approximately 3 min to complete.^175^ The CES is scored from an algorithm derived from preferences of the general population and can be used to value carer outcomes in economic evaluation using index values.^176^

#### The McMaster family assessment device general functioning scale

Caring for a child with a disability can have an impact on the health and functioning of the caregiver and family unit.^177^ The family assessment device is a 12-item measure of general family functioning.^178^ Each item is rated on a four-point Likert scale from strongly agree to strongly disagree and the items are summed to create the total score. It has good internal consistency (Cronbach’s α=0.92), construct validity and reliability.^178–180^

#### Monitoring of resource use and direct costs of treatments

To determine the relationship between motor prognosis and healthcare costs, resource use and direct costs of treatment will be monitored using the Health Resource Use questionnaire^14^ (see online supplementary Appendix 6). Associations between costs (dependent variable) and all other outcome variables (independent variables) including those related to growth, body composition and HPA will be assessed, with adjustment for confounders such as brain lesion severity. Health-related resource use data will be collected including therapy frequency and duration (traditional/alternate), hospital admissions, GP and medical specialist visits, medications (eg, Botulinum toxin A) and equipment (eg, orthoses). Data will be collected via questionnaire,^14^ supplemented by consented access to individual hospital, Medical Benefits Scheme (MBS) and Pharmaceutical Benefits Scheme (PBS) records. Standard cost sources (MBS, PBS) will be used to apply unit costs to resources. Statistical approaches, which consider the likely skewed distribution of cost data (such as generalised linear models, with extensions to allow for correlations in the data across observations from multiple time points from each individual as described below), and diagnostic-related group costs for admissions to hospital) will be employed. As this cohort is embedded in a state-wide clinical service, we have consistency of interventions based on best practice guidelines for Botulinum toxin A, bone health and hip surveillance generalisable across Australia. All children are offered best practice treatment across the state-wide service.

### Data analysis plan

A comprehensive database has been established for all data collection, including clinical measures, MRI scoring and questionnaires so that it is entered prospectively at the time of each assessment. Summary reports are automatically generated from the database to report back to families and treating clinicians after each visit. Biostatistics methods proposed in this study include analysis of binary outcomes in longitudinal studies using weighted estimating equations (eg, presence of comorbidities); multilevel mixed-effects models of longitudinal binary outcomes (eg, GMFCS levels) and generalised estimating equations for ordinal data.

### Sample size

We assume 95% of the total sample of 245 children consent to the PREDICT-CP programme (we have 98% retention in NHMRC 465128, 569605). For Hypothesis 1,^1^ assuming between-GMFCS group and within-child variability in GMFM-66 were similar to findings by Rosenbaum *et al*,^181^ we were able to detect significant between-group differences in GMFM-66 scores at 8–12 years according to the primary predictor variable of initial GMFCS group (aged 2–5 years) with >80% power and alpha=0.05 for each pairwise comparison. For comparisons between MRI classification at 2 years and GMFM-66 at 8–12 years, then based on GMFCS and MRI data from NHMRC 465128 (where GMFCS I=30%, II–V=16% each and white matter injury=43%), assuming that GMFM-66 at 8–12 years has a SD of 7 units within each GMFCS group,^181^ we are able to detect a difference between children with white-matter damage and other MRI pattern types of ≥4.6 GMFM-66 points in GMFCS I and ≥6.7 points within each GMFCS level II–V, with 80% power, alpha=0.05. For secondary outcomes, we have differing power depending on sample characteristics, for example, two groups, evenly distributed, we will detect differences of ≥0.36 SD with α=0.05, 80% power.

### Statistical considerations

Summary statistics will be described using either mean (SD) or median (25th–75th percentile) for continuous variables, according to distribution, or as frequency (percentage) for categorical variables. Cross-sectional associations will be assessed using linear regression for interval outcome data, with effect estimates presented as mean difference and 95% CI, using logistic regression for binary outcome data, with effect estimates presented as ORs and 95% CI, and using Poisson regression for count outcome data, with effect estimates presented as incidence rate ratios and 95% CI. Longitudinal associations will be investigated using analyses that account for the multiple observations per participant. The particular analysis will be determined by data structure, for example, hierarchical mixed-effects models or generalised estimating equations. If hierarchical mixed-effects models are used then ‘participant’ will be included in the model as a random effect, to account for the possible non-independence of observations from the same participant. When building multivariable models, first univariable models including all potential variables of interest will be constructed. Variables will be selected for potential inclusion in multivariable models based on univariable significance at the p<0.2 level. Multivariable models will be built in a stepwise manner with redundant variables eliminated using Akaike’s and Schwarz’s Bayesian criteria. In principle, multivariable models will be first constructed for the whole sample, then after stratification by GMFCS level. Interactions are investigated as appropriate.

Missing data were treated on a case-by-case basis depending on the observed pattern of missingness. For example, if data were ‘missing at random’, we will use multiple imputation methods, and if data are ‘not missing at random’, we will use pattern-mixture models. There was no global rule to account for multiple comparisons, instead adjustment for multiple comparisons will be made for each separate suite of analyses as appropriate, bearing in mind the type I and II error rates for each suite. Cost data will be standardised to current values. Cost data are typically skewed and therefore will be tested for normality and transformed using a log, gamma or another appropriate link function for the multivariable analysis.

Complex multivariable analyses accounting for attrition, if required, will be conducted by a biostatistician. The precise analyses will be determined by data structure. In principle, hierarchical mixed-effects models will be performed for the whole population then by GMFCS level. First, univariable then multivariable analyses will be undertaken. Variables included as fixed effects in the final multivariate model will be selected based on univariate results if p<0.2. Redundant variables are eliminated from multivariable models using Akaike’s and Schwarz’s Bayesian criteria. All models include a random intercept and slope (time effect) for each participant, accounting for the non-independence in repeated measures from the same participant and allowing for heterogeneity between participants. For Hypothesis 1,^1^ we will first undertake univariable analyses of the association between quantitative MRI (primary), motor capacity (primary), performance and potential confounding variables (eg, gender) at 2–5 years with motor capacity at 8 -12 years using a mixed-effects model with data grouped by individual participants to account for (up to 3) repeated measures between 2 and 5 years. Then all variables significant at p<0.2 will be included in the prediction model, before being investigated for elimination. Interactions will be investigated as appropriate. Missing data will be treated on a case-by-case basis using MAR (multiple imputation algorithms) or NMAR (using pattern-mixture models). Adjustment for multiple comparisons will be made for each separate analyses mindful of type I and II error rates, as is standard practice.

## Supplementary Material

Reviewer comments

Author's manuscript
