# Supplementary material for: PREDICT-CP: study protocol of implementation of comprehensive surveillance to predict outcomes for school-aged children with cerebral palsy
Source: BMJ Open. 2017 Jul 12;7(7):e014950. doi: 10.1136/bmjopen-2016-014950 (PMC5734266; doi:10.1136/bmjopen-2016-014950)
Supplement: Supplementary Appendix 1 [file bmjopen-2016-014950supp001.pdf]

Appendix 1: Qld Predict CP – Physician's checklist

**Diagnosis**		COMMENTS
Cerebral palsy	yes/ no/ unsure	Definition (Rosenbaum et al., 2005): "Cerebral Palsy (CP) describes a group of disorders of the development of movement and posture, causing activity limitation that are attributed to non-progressive disturbances that occurred in the developing foetal or infant brain. The motor disorders of cerebral palsy are often accompanied by disturbances of sensation, cognition, communication, perception, and/or behaviour, and/or by a seizure disorder."<br><br>What constitutes Cerebral Palsy? (Badawi et al., 1998): Cerebral palsy (CP) is a term of convenience applied to a group of motor disorders of central origin defined by clinical description. It is not a diagnosis in that its application infers nothing about pathology, aetiology or prognosis. It is an umbrella term covering a wide range of disorders which result in childhood motor impairment. There must be motor impairment, and this impairment must stem from a malfunction of the brain (rather than spinal cord or muscles). Furthermore, the brain malfunction must be non-progressive and it must manifest early in life.

Patterns of motor impairment

			COMMENTS	
Motor type	Spastic		See attachment for further information (SCPE definitions, 2000<br><br>For mixed motor type state 1 for dominant and 2 for secondary etc.	
	Ataxic			
	dystonic			
	choreoathetotic			
	Hypotonic			
	Hyperkinetic			
Distribution	Bilateral/ unilateral			
	No of limbs (based on activity or function not passive testing of muscle tone)		1/ 2/ 3/ 4	
Head circumference –				

current (cm)			

Functional level		COMMENTS
GMFCS level	I/ II/ III/ IV/ V	
Age at classification	years months	Age of child when this assessment is being performed (and GMFCS is classified)
Upper limb/ Handedness	Right predominant/ Left predominant/ Bilateral	

Co-morbidities		COMMENTS
Epilepsy	No<br><br>Yes (defined by 2 unprovoked seizures excluding febrile or neonatal seizures)<br><br>If yes, still on medication, Yes/ No	
Seizure type		Ask the family to describe exactly what they observed.<br><br>Generalised or partial<br><br>Generalised – sudden onset of seizures that compromises responsiveness and affects the whole body.<br><br>Partial – seizures have focality therefore symptoms reflect onset in 1 part of the brain<br><br>Date of commencement of seizures
Seizure syndrome		
Controlled/ not controlled		
Medications	Yes/ no/ not applicable	Medications for seizures or any other medications

Visual impairment<br><br>(after correction, on the better eye)	Normal<br><br>Impaired<br><br>Severely impaired (blind or no useful vision)	
Hearing impairment (before correction, on the better ear)	Normal<br><br>Impaired<br><br>Severely impaired (hearing loss > 70 dB	
Speech	Normal<br><br>Delayed	Expressive<br><br>Receptive<br><br>Both Unclassified
Communication	Oral<br><br>Signs<br><br>Device	
Intellectual impairment	Mild<br><br>Moderate<br><br>Severe<br><br>Probably impaired, severity unknown<br><br>Probably no impairment<br><br>No impairment<br><br>Unknown	SCPE classification:<br><br>Normal- IQ>=85, attendance of regular school without support<br><br>Borderline- IQ 70 to 84<br><br>Mild impairment- IQ 50 to 69, some basic literacy and numeracy achieved<br><br>Moderate impairment- IQ 20-49<br><br>Severe impairment- IQ < 20
Nutrition		
Body weight	kg / percentile	
Body height	cm / percentile	

Method of nutrition	Oral<br><br>Tube- nasogastric<br><br>PEG	PEG – percutaneous endoscopic gastrostomy
	Partially<br><br>Entirely	

Respiratory Health		COMMENTS
No of hospitalisations for chest infections in the past 6 months (since last visit)		
No of episodes of pneumonia		
Asthma	yes/ no	
No of episodes of asthmatic attacks in the past 6 months (since last visit)		
Others	Constipation<br><br>Diabetes<br><br>Cardiac conditions	
Continence	Urinary<br><br>Faecal	
Past surgical history		
Examination findings		Clinical signs and symptoms
MRI previous (yes/no)<br><br>date<br><br>location		

Appendix 2: Predict CP study - 7 day physical activity monitor log

Thank you for helping with our research! This study aims to identify when your child is active and when they are inactive, so that we can work out how much movement and physical activity children with Cerebral Palsy do as part of their daily life.

To do this we are using an activity monitor together with a logbook of wear time. To help make sure our measures are as accurate as possible, please read the instructions below.

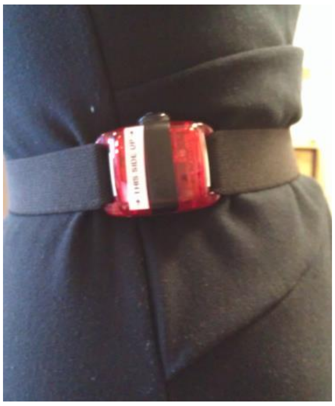

The activity monitor

Where do I wear it? The belt should be worn around your child's waist with the monitor on the side of the hip, on the dominant side. That is, wear the monitor on the (circle) RIGHT / LEFT side.

Position the Actigraph with the arrows pointing up and the open/close button on top. Remember that the monitor is already on and recording and don't worry if it flashes.

How many days should I wear this? Seven days. This should be made up of five weekdays and two weekend days.

How long do I wear it? The monitor should be worn as much as possible during waking hours. It can be hidden under clothing as long as it is on the belt and firm against the waist on the side of the hip, on the unaffected side.

Can I take the monitor off? Take the monitor off only:
When going to sleep; or for water activities such as having a shower, or swimming.

Important points:

Wear the monitor on the belt around the waist on the side of the hip, on the dominant side!
Wear the monitor all hours your child is awake, taking it off only to sleep or if having a swim or shower.

The log book

The log book allows us to compare the movement of the activity monitor to what you record doing at the time – this allows us to check it is accurate.

What do I need to do? At the top of the form are some questions about when the monitor was worn and when your child was asleep. Underneath this we then need you to we need you to record for each

half hour if the monitor was on or off. Under this, each block represents 15minutes of activity. Simply tick the box that corresponds to the activity – eg. Swimming, sleeping, sitting or walking.

How many days should I do this? The same seven days that your child is wearing the activity monitor.

Monitor on: _____am/pm ______________ Monitor off: _____am/pm ______________

Once you have completed the seven days, put both the activity monitor and the log book into the return mail bag provided, peel off the registered post number, and put it into a Yellow Express Post Box to return it to us.

Thank you for your help and participation in this study.

Appendix 3: PREDICT CP- 7 day sun Exposure Diary

PREDICT CP 7- Day Sun Exposure Diary

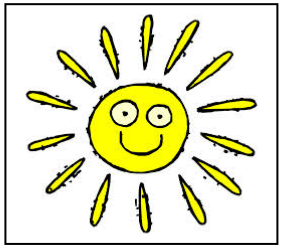

Name of Child: …………………………………………. Date of Birth: …..……………. Dates of Diary: ……………………………………………

Instructions for completing diary
Please read the following instructions on how to complete your sun exposure diary.

• **Please answer all the questions on the first page**

• **For each day:** record the date

• **Time Outside in the Sun**: For each hour interval, put a cross in the box under the amount of time you spent in the sun during that interval. Please **do not leave** any hour interval(s) not ticked; i.e. tick '0 minutes' if you have not been exposed to the sun for that particular hour interval.

• **Sunscreen:** Put a cross in the 'YES" or 'NO' box on the top right of the page to indicate if you have applied sunscreen that day. If sunscreen was applied, shade in the area(s) on the diagram to reflect where you have applied sunscreen on your body that day. Please also put a cross in a box under the "**Did You Apply Sunscreen"** column, against the hour(s) of the day at which you applied sunscreen. If you did not apply sunscreen that day, do not put a cross in any of the boxes under this column

• **Clothing Worn**: Use the '**Sun Diary Clothing Guide**' to fill in the type of clothing you wore for each hour interval that day. Insert the relevant number that matches the picture for upper and lower body, headwear, and footwear. Please make sure you have specified all clothing worn at each hour interval, and that you have **written a number in every box in this section.** Please put a cross against the hour(s) that gloves have been worn that day. If no gloves have been worn, do not put a cross in any of the boxes in the gloves column.

To return the diary
A courier will collect the completed sun exposure diary from your home at a time that is convenient to you

Any questions please contact **Camilla:** 30697355, camilla.davenport@uq.edu.au

**Office Use Only**
RA signature:
RA name:
Date:
Study ID:

Questions about usual sun exposure

Please answer **all** the following questions. Please **circle** or write your answers in the space provided.

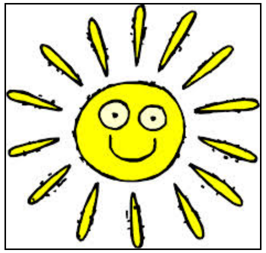

1. Have you been on a holiday in the sun in last month?   YES / NO

2. Does your child usually wear sunscreen? YES / NO

   If **YES** what brand? ____________________

   What SPF factor? ______________________

3. What is the colour of your child's **untanned** skin?

   Very fair

   Fair

   Olive

   Light brown

   Dark Brown

Please refer to the following information as a guide to filling out the Sun Exposure Diary.

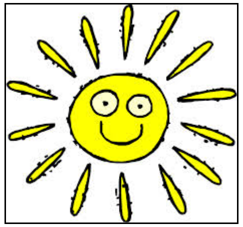

Day Example: Monday 01/07/2015

Name: Elsa Snow

Sunscreen: applied at 6.00am on face, neck, upper and lower limbs (excluding palms of hands and feet), again at 12 noon.

Time Outside in the Sun:

- 6-7 am: 15 minutes, playing **outside** at home
- 8-9 am: 20 minutes, driving to school in car
- 12-1 pm: 10 minutes, playing **outside** at school
- 1-2 pm: 10 minutes, playing **outside** at school
- 5-6 pm: 10 minutes, walking to swimming lessons **outside**
- 6-7 pm: 30 minutes, swimming lessons indoors

Type of Clothing Worn:

- 6 am to 6 pm: short-sleeved top with shorts, no headwear, enclosed shoes. These items worn until 6.00pm.
- 12-1pm & 1-2pm: “as above” with legionnaire’s cap

- 6-7 pm: swimsuit, no headwear, no foot or hand wear.

SUN DIARY CLOTHING GUIDE							
**UPPER BODY**	**NO CLOTHING ON UPPER BODY**<br>**0**<br>**No upper clothing**	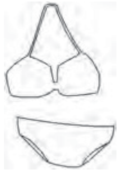<br>**1**<br>**Bikini**	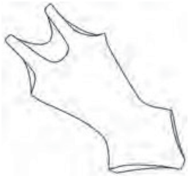<br>**2**<br>**Swimsuit**	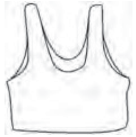<br>**3**<br>**Crop top**	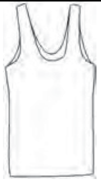<br>**4**<br>**Singlet top**	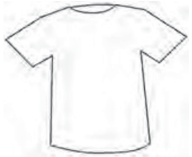<br>**5**<br>**Short-sleeved top**	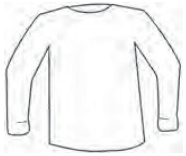<br>**6**<br>**Long-sleeved top**
**LOWER BODY**	**NO CLOTHING ON LOWER BODY**<br>**0**<br>**No lower clothing**	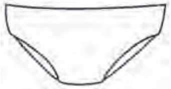<br>**1**<br>**Speedos/briefs**	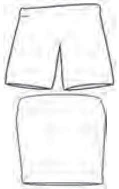<br>**2**<br>**Shorts or short skirt**	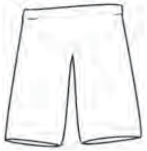<br>**3**<br>**Medium shorts or 3/4 pants**	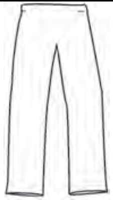<br>**4**<br>**Long trousers/ jeans**	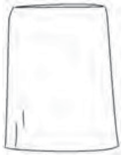<br>**5**<br>**Medium skirt**	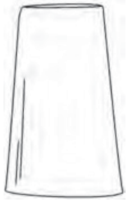<br>**6**<br>**Long skirt**
**HEADWEAR**	**NO HEADWEAR**<br>**0**<br>**No headwear**	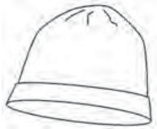<br>**1**<br>**Beanie**	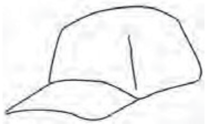<br>**2**<br>**Cap**	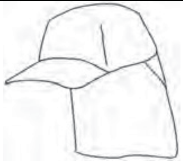<br>**3**<br>**Legionnaire's cap**	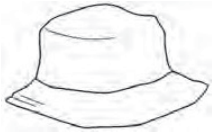<br>**4**<br>**Bucket hat**	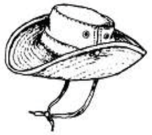<br>**5**<br>**Wide-brimmed hat**	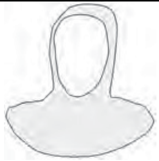<br>**6**<br>**Veil/burkha**
**FOOTWEAR**	**NO FOOTWEAR/ HANDWEAR**<br>**0**<br>**No footwear**	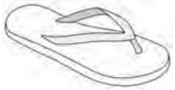<br>**1**<br>**Thong/open sandals**	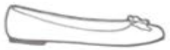<br>**2**<br>**Semi-enclosed shoes**	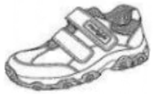<br>**3**<br>**Enclosed shoes**	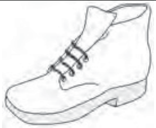<br>**4**<br>**Boots**	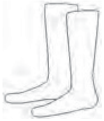<br>**5**<br>**Long socks**	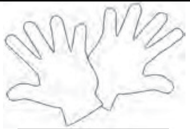<br>**HANDWEAR**<br>**Gloves**

DAY EXAMPLE
DATE 01/07/15

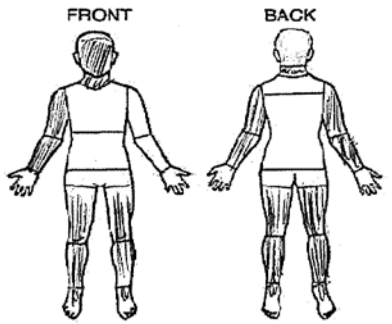

Did you apply sunscreen today?

☑ Yes ☐ No

If yes, shade the diagram on the left to show the parts of the body that you applied sunscreen to today

	TIME OUTDOORS (Cross the box which best represents the amount of time you spent outdoors during each one hour interval shown below)					DID YOU APPLY SUNSCREEN	INSERT NUMBER DENOTING TYPE OF CLOTHING WORN (You must write one number in each box)				
	0 minutes	Below 15 minutes	15-30 minutes	30-45 minutes	45-60 minutes	Cross as many as applicable	Upper body	Lower body	Head wear	Footwear	Gloves (cross all that apply)
**Morning**											
5 - 6 am	☑	☐	☐	☐	☐	☐	0	0	0	0	☐
6 - 7 am	☐	☐	☑	☐	☐	☑	5	2	0	3	☐
7 - 8 am	☑	☐	☐	☐	☐	☐	5	2	0	3	☐
8 - 9 am	☑	☐	☐	☐	☐	☐	5	2	0	3	☐
9 - 10 am	☑	☐	☐	☐	☐	☐	5	2	0	3	☐
10 - 11 am	☑	☐	☐	☐	☐	☐	5	2	0	3	☐
11 - 12 am	☑	☐	☐	☐	☐	☐	5	2	0	3	☐
**Afternoon**											
12 - 1 pm	☐	☑	☐	☐	☐	☑	5	2	3	3	☐
1 - 2 pm	☐	☑	☐	☐	☐	☐	5	2	3	3	☐
2 - 3 pm	☑	☐	☐	☐	☐	☐	5	2	0	3	☐
3 - 4 pm	☑	☐	☐	☐	☐	☐	5	2	0	3	☐
4 - 5 pm	☑	☐	☐	☐	☐	☐	5	2	0	3	☐
5 - 6 pm	☐	☑	☐	☐	☐	☐	5	2	0	3	☐
6 - 7 pm	☑	☐	☐	☐	☐	☐	2	0	0	0	☐

PREDICT_Sun Diary page 5

DAY 1

DATE _ _ / _ _ / _ _

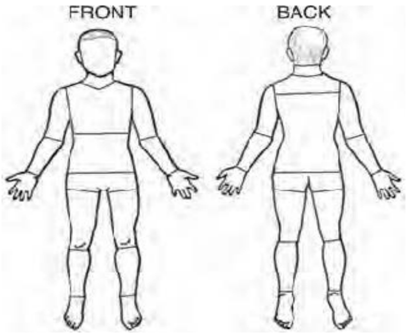

Did you apply sunscreen today?

☐ Yes ☐ No

If yes, shade the diagram on the left to show the parts of the body that you applied sunscreen to today

	**TIME OUTDOORS** (Cross the box which best represents the amount of time you spent outdoors during each one hour interval shown below)					**DID YOU APPLY SUNSCREEN**	**INSERT NUMBER DENOTING TYPE OF CLOTHING WORN** (You must write one number in each box)				
	0 minutes	Below 15 minutes	15-30 minutes	30-45 minutes	45-60 minutes	Cross as many as applicable	Upper body	Lower body	Head wear	Footwear	Gloves (cross all that apply)
**Morning**											
5 - 6 am	☐	☐	☐	☐	☐	☐					☐
6 - 7 am	☐	☐	☐	☐	☐	☐					☐
7 - 8 am	☐	☐	☐	☐	☐	☐					☐
8 - 9 am	☐	☐	☐	☐	☐	☐					☐
9 - 10 am	☐	☐	☐	☐	☐	☐					☐
10 - 11 am	☐	☐	☐	☐	☐	☐					☐
11 - 12 am	☐	☐	☐	☐	☐	☐					☐
**Afternoon**											
12 - 1 pm	☐	☐	☐	☐	☐	☐					☐
1 - 2 pm	☐	☐	☐	☐	☐	☐					☐
2 - 3 pm	☐	☐	☐	☐	☐	☐					☐
3 - 4 pm	☐	☐	☐	☐	☐	☐					☐
4 - 5 pm	☐	☐	☐	☐	☐	☐					☐
5 - 6 pm	☐	☐	☐	☐	☐	☐					☐
6 - 7 pm	☐	☐	☐	☐	☐	☐					☐

DAY 2

DATE _ _ / _ _ / _ _

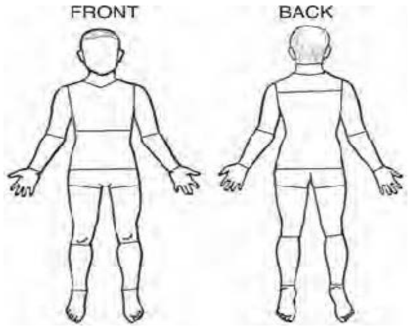

Did you apply sunscreen today?

☐ Yes ☐ No

If yes, shade the diagram on the left to show the parts of the body that you applied sunscreen to today

	**TIME OUTDOORS** (Cross the box which best represents the amount of time you spent outdoors during each one hour interval shown below)					**DID YOU APPLY SUNSCREEN**	**INSERT NUMBER DENOTING TYPE OF CLOTHING WORN** (You must write one number in each box)				
	0 minutes	Below 15 minutes	15-30 minutes	30-45 minutes	45-60 minutes	Cross as many as applicable	Upper body	Lower body	Head wear	Footwear	Gloves (cross all that apply)
**Morning**											
5 - 6 am	☐	☐	☐	☐	☐	☐					☐
6 - 7 am	☐	☐	☐	☐	☐	☐					☐
7 - 8 am	☐	☐	☐	☐	☐	☐					☐
8 - 9 am	☐	☐	☐	☐	☐	☐					☐
9 - 10 am	☐	☐	☐	☐	☐	☐					☐
10 - 11 am	☐	☐	☐	☐	☐	☐					☐
11 - 12 am	☐	☐	☐	☐	☐	☐					☐
**Afternoon**											
12 - 1 pm	☐	☐	☐	☐	☐	☐					☐
1 - 2 pm	☐	☐	☐	☐	☐	☐					☐
2 - 3 pm	☐	☐	☐	☐	☐	☐					☐
3 - 4 pm	☐	☐	☐	☐	☐	☐					☐
4 - 5 pm	☐	☐	☐	☐	☐	☐					☐
5 - 6 pm	☐	☐	☐	☐	☐	☐					☐
6 - 7 pm	☐	☐	☐	☐	☐	☐					☐

**DAY 3**
DATE _ _ / _ _ / _ _

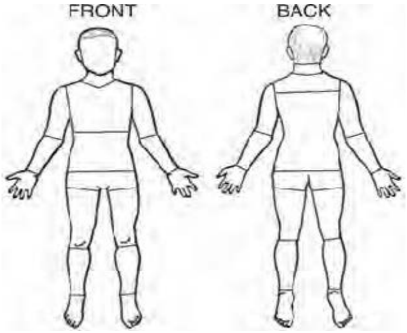

Did you apply sunscreen today?

☐ Yes ☐ No

If yes, shade the diagram on the left to show the parts of the body that you applied sunscreen to today

	**TIME OUTDOORS** (Cross the box which best represents the amount of time you spent outdoors during each one hour interval shown below)					**DID YOU APPLY SUNSCREEN**	**INSERT NUMBER DENOTING TYPE OF CLOTHING WORN** (You must write one number in each box)				
	0 minutes	Below 15 minutes	15-30 minutes	30-45 minutes	45-60 minutes	Cross as many as applicable	Upper body	Lower body	Head wear	Footwear	Gloves (cross all that apply)
**Morning**											
5 - 6 am	☐	☐	☐	☐	☐	☐	☐	☐	☐	☐	☐
6 - 7 am	☐	☐	☐	☐	☐	☐	☐	☐	☐	☐	☐
7 - 8 am	☐	☐	☐	☐	☐	☐	☐	☐	☐	☐	☐
8 - 9 am	☐	☐	☐	☐	☐	☐	☐	☐	☐	☐	☐
9 - 10 am	☐	☐	☐	☐	☐	☐	☐	☐	☐	☐	☐
10 - 11 am	☐	☐	☐	☐	☐	☐	☐	☐	☐	☐	☐
11 - 12 am	☐	☐	☐	☐	☐	☐	☐	☐	☐	☐	☐
**Afternoon**											
12 - 1 pm	☐	☐	☐	☐	☐	☐	☐	☐	☐	☐	☐
1 - 2 pm	☐	☐	☐	☐	☐	☐	☐	☐	☐	☐	☐
2 - 3 pm	☐	☐	☐	☐	☐	☐	☐	☐	☐	☐	☐
3 - 4 pm	☐	☐	☐	☐	☐	☐	☐	☐	☐	☐	☐
4 - 5 pm	☐	☐	☐	☐	☐	☐	☐	☐	☐	☐	☐
5 - 6 pm	☐	☐	☐	☐	☐	☐	☐	☐	☐	☐	☐
6 - 7 pm	☐	☐	☐	☐	☐	☐	☐	☐	☐	☐	☐

DAY 4

DATE _ _ / _ _ / _ _

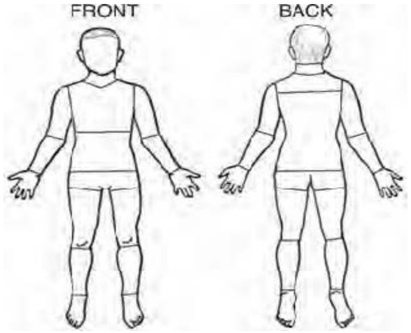

Did you apply sunscreen today?

☐ Yes ☐ No

If yes, shade the diagram on the left to show the parts of the body that you applied sunscreen to today

	TIME OUTDOORS (Cross the box which best represents the amount of time you spent outdoors during each one hour interval shown below)					DID YOU APPLY SUNSCREEN	INSERT NUMBER DENOTING TYPE OF CLOTHING WORN (You must write one number in each box)				
	0 minutes	Below 15 minutes	15-30 minutes	30-45 minutes	45-60 minutes	Cross as many as applicable	Upper body	Lower body	Head wear	Footwear	Gloves (cross all that apply)
**Morning**											
5 - 6 am	☐	☐	☐	☐	☐	☐	☐	☐	☐	☐	☐
6 - 7 am	☐	☐	☐	☐	☐	☐	☐	☐	☐	☐	☐
7 - 8 am	☐	☐	☐	☐	☐	☐	☐	☐	☐	☐	☐
8 - 9 am	☐	☐	☐	☐	☐	☐	☐	☐	☐	☐	☐
9 - 10 am	☐	☐	☐	☐	☐	☐	☐	☐	☐	☐	☐
10 - 11 am	☐	☐	☐	☐	☐	☐	☐	☐	☐	☐	☐
11 - 12 am	☐	☐	☐	☐	☐	☐	☐	☐	☐	☐	☐
**Afternoon**											
12 - 1 pm	☐	☐	☐	☐	☐	☐	☐	☐	☐	☐	☐
1 - 2 pm	☐	☐	☐	☐	☐	☐	☐	☐	☐	☐	☐
2 - 3 pm	☐	☐	☐	☐	☐	☐	☐	☐	☐	☐	☐
3 - 4 pm	☐	☐	☐	☐	☐	☐	☐	☐	☐	☐	☐
4 - 5 pm	☐	☐	☐	☐	☐	☐	☐	☐	☐	☐	☐
5 - 6 pm	☐	☐	☐	☐	☐	☐	☐	☐	☐	☐	☐
6 - 7 pm	☐	☐	☐	☐	☐	☐	☐	☐	☐	☐	☐

DAY 5
DATE _ _ / _ _ / _ _

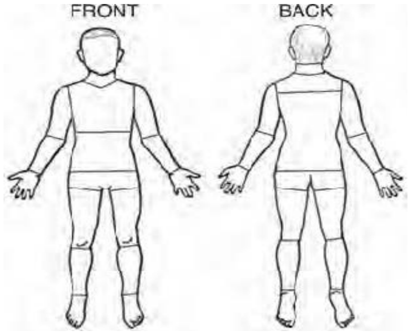

Did you apply sunscreen today?

☐ Yes ☐ No

If yes, shade the diagram on the left to show the parts of the body that you applied sunscreen to today

	**TIME OUTDOORS** (Cross the box which best represents the amount of time you spent outdoors during each one hour interval shown below)					**DID YOU APPLY SUNSCREEN**	**INSERT NUMBER DENOTING TYPE OF CLOTHING WORN** (You must write one number in each box)				
	0 minutes	Below 15 minutes	15-30 minutes	30-45 minutes	45-60 minutes	Cross as many as applicable	Upper body	Lower body	Head wear	Footwear	Gloves (cross all that apply)
**Morning**											
5 - 6 am	☐	☐	☐	☐	☐	☐					☐
6 - 7 am	☐	☐	☐	☐	☐	☐					☐
7 - 8 am	☐	☐	☐	☐	☐	☐					☐
8 - 9 am	☐	☐	☐	☐	☐	☐					☐
9 - 10 am	☐	☐	☐	☐	☐	☐					☐
10 - 11 am	☐	☐	☐	☐	☐	☐					☐
11 - 12 am	☐	☐	☐	☐	☐	☐					☐
**Afternoon**											
12 - 1 pm	☐	☐	☐	☐	☐	☐					☐
1 - 2 pm	☐	☐	☐	☐	☐	☐					☐
2 - 3 pm	☐	☐	☐	☐	☐	☐					☐
3 - 4 pm	☐	☐	☐	☐	☐	☐					☐
4 - 5 pm	☐	☐	☐	☐	☐	☐					☐
5 - 6 pm	☐	☐	☐	☐	☐	☐					☐
6 - 7 pm	☐	☐	☐	☐	☐	☐					☐

DAY 6

DATE _ _ / _ _ / _ _

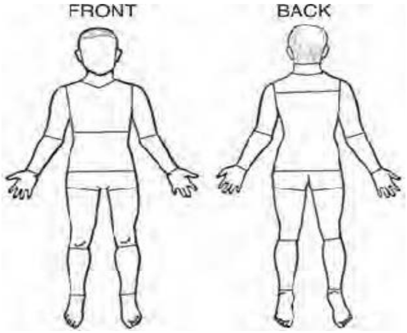

Did you apply sunscreen today?

☐ Yes ☐ No

If yes, shade the diagram on the left to show the parts of the body that you applied sunscreen to today

	**TIME OUTDOORS** (Cross the box which best represents the amount of time you spent outdoors during each one hour interval shown below)					**DID YOU APPLY SUNSCREEN**	**INSERT NUMBER DENOTING TYPE OF CLOTHING WORN** (You must write one number in each box)				
	0 minutes	Below 15 minutes	15-30 minutes	30-45 minutes	45-60 minutes	Cross as many as applicable	Upper body	Lower body	Head wear	Footwear	Gloves (cross all that apply)
**Morning**											
5 - 6 am	☐	☐	☐	☐	☐	☐	☐	☐	☐	☐	☐
6 - 7 am	☐	☐	☐	☐	☐	☐	☐	☐	☐	☐	☐
7 - 8 am	☐	☐	☐	☐	☐	☐	☐	☐	☐	☐	☐
8 - 9 am	☐	☐	☐	☐	☐	☐	☐	☐	☐	☐	☐
9 - 10 am	☐	☐	☐	☐	☐	☐	☐	☐	☐	☐	☐
10 - 11 am	☐	☐	☐	☐	☐	☐	☐	☐	☐	☐	☐
11 - 12 am	☐	☐	☐	☐	☐	☐	☐	☐	☐	☐	☐
**Afternoon**											
12 - 1 pm	☐	☐	☐	☐	☐	☐	☐	☐	☐	☐	☐
1 - 2 pm	☐	☐	☐	☐	☐	☐	☐	☐	☐	☐	☐
2 - 3 pm	☐	☐	☐	☐	☐	☐	☐	☐	☐	☐	☐
3 - 4 pm	☐	☐	☐	☐	☐	☐	☐	☐	☐	☐	☐
4 - 5 pm	☐	☐	☐	☐	☐	☐	☐	☐	☐	☐	☐
5 - 6 pm	☐	☐	☐	☐	☐	☐	☐	☐	☐	☐	☐
6 - 7 pm	☐	☐	☐	☐	☐	☐	☐	☐	☐	☐	☐

DAY 7

DATE _ _ / _ _ / _ _

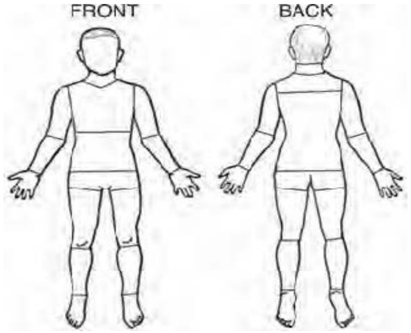

Did you apply sunscreen today?

☐ Yes ☐ No

If yes, shade the diagram on the left to show the parts of the body that you applied sunscreen to today

	TIME OUTDOORS (Cross the box which best represents the amount of time you spent outdoors during each one hour interval shown below)					DID YOU APPLY SUNSCREEN	INSERT NUMBER DENOTING TYPE OF CLOTHING WORN (You must write one number in each box)				
	0 minutes	Below 15 minutes	15-30 minutes	30-45 minutes	45-60 minutes	Cross as many as applicable	Upper body	Lower body	Head wear	Footwear	Gloves (cross all that apply)
**Morning**											
5 - 6 am	☐	☐	☐	☐	☐	☐	☐	☐	☐	☐	☐
6 - 7 am	☐	☐	☐	☐	☐	☐	☐	☐	☐	☐	☐
7 - 8 am	☐	☐	☐	☐	☐	☐	☐	☐	☐	☐	☐
8 - 9 am	☐	☐	☐	☐	☐	☐	☐	☐	☐	☐	☐
9 - 10 am	☐	☐	☐	☐	☐	☐	☐	☐	☐	☐	☐
10 - 11 am	☐	☐	☐	☐	☐	☐	☐	☐	☐	☐	☐
11 - 12 am	☐	☐	☐	☐	☐	☐	☐	☐	☐	☐	☐
**Afternoon**											
12 - 1 pm	☐	☐	☐	☐	☐	☐	☐	☐	☐	☐	☐
1 - 2 pm	☐	☐	☐	☐	☐	☐	☐	☐	☐	☐	☐
2 - 3 pm	☐	☐	☐	☐	☐	☐	☐	☐	☐	☐	☐
3 - 4 pm	☐	☐	☐	☐	☐	☐	☐	☐	☐	☐	☐
4 - 5 pm	☐	☐	☐	☐	☐	☐	☐	☐	☐	☐	☐
5 - 6 pm	☐	☐	☐	☐	☐	☐	☐	☐	☐	☐	☐
6 - 7 pm	☐	☐	☐	☐	☐	☐	☐	☐	☐	☐	☐

Thank you very much for answering the questions and completing the diary. Please be assured that this information will be kept confidential.

Appendix 4: Augmentative and Alternative Communication (AAC) Questionnaire

Communication System ○ Yes ○ No

Is it:

Makaton/ formal signs: number of signs: ______

Symbols or pictures for choices: number presented: ______

Communication board: number of words/ symbols: ______

Communication book: number of words/ symbols: ______

Single message device (eg Big Mack/ Step-by-Step)

Voice output communication device: name of device ____________________________

Access:

Eye pointing

Laser/ infra-red pointer

Visual scanning

Auditory scanning

Direct access (pointing)

Frequency of use hours per day: □□

days per week: □

Appendix 5 PREDICT: Demographic questionnaire

What is your relationship to this child?

( ) Mother (biological or adoptive)

( ) Father (biological or adoptive)

( ) Step mother

( ) Step Father

( ) Legal Guardian (please explain)

What is your date of birth?

Does your child have any other difficulties, in addition to Cerebral Palsy, which would affect their participation in home, school or community activities (please tick any that apply)?

( ) Epilepsy

( ) Autism Spectrum Disorder (including Asperger Syndrome)

( ) ADHD

( ) Hearing Impairment

( ) Intellectual Impairment

( ) Learning Disability

( ) Visual Impairment

( ) Other (please explain)

Which best describes the household in which your child is currently living?

( ) Original, two parent family (both biological or adoptive parents are living in the same household as the child)

( ) Step, two parent family (two parents are living in the same household as the child and one is a step-parent)

( ) Shared custody (child lives in two separate households and divides his/her time between them)

( ) Sole parent family (Child lives with one parent only, may have contact with other parent)

( )	Other (please explain)

What is your current marital status?

( )	Married

( )	Defacto

( )	Separated

( )	Divorced

( )	Never married/defacto

( )	Widow/er

Does your child have any siblings living in the same household?

( )	Yes

( )	No

For each sibling living in the same household with your children please indicate the following:

	Sibling 1	Sibling 2	Sibling 3	Sibling 4
Age:				
Gender:				
Any difficulties that would affect their participation in home, school or community activities (please tick any that apply)?	( ) Nil<br>( ) Epilepsy<br>( ) Autism Spectrum Disorder (including Asperger Syndrome<br>( ) ADH<br>( ) Hearing Impairment<br>( ) Intellectual Impairment<br>( ) Learning	( ) Nil<br>( ) Epilepsy<br>( ) Autism Spectrum Disorder (including Asperger Syndrome<br>( ) ADH<br>( ) Hearing Impairment<br>( ) Intellectual Impairment<br>( ) Learning	( ) Nil<br>( ) Epilepsy<br>( ) Autism Spectrum Disorder (including Asperger Syndrome<br>( ) ADH<br>( ) Hearing Impairment<br>( ) Intellectual Impairment<br>( ) Learning	( ) Nil<br>( ) Epilepsy<br>( ) Autism Spectrum Disorder (including Asperger Syndrome<br>( ) ADH<br>( ) Hearing Impairment<br>( ) Intellectual Impairment<br>( ) Learning

	Disability	Disability	Disability	Disability
	( ) Speech Language Difficulty	( ) Speech Language Difficulty	( ) Speech Language Difficulty	( ) Speech Language Difficulty
	( ) Visual Impairment	( ) Visual Impairment	( ) Visual Impairment	( ) Visual Impairment
	( ) Other (please explain)	( ) Other (please explain)	( ) Other (please explain)	( ) Other (please explain)

Age:

Gender:

Any difficulties that would affect their participation in home, school or community activities (please tick any that apply)?

( ) Epilepsy

( ) Autism Spectrum Disorder (including Asperger Syndrome)

( ) ADHD

( ) Hearing Impairment

( ) Intellectual Impairment

( ) Learning Disability

( ) Speech/Language Difficulty

( ) Visual Impairment

( ) Other (please explain)

Is English the main language spoken at home?

( ) Yes

( ) No, please specific the main language:

What is your postcode?

What is *your* highest level of education?

( ) Less than year 10

( ) Year 10/11

( ) Year 12

( ) Trade/apprenticeship

( ) TAFE/college certificate

( ) University degree

( ) University postgraduate degree

What is *your partner's* highest level of education?

( ) Less than year 10

( ) Year 10/11

( ) Year 12

( ) Trade/apprenticeship

( ) TAFE/college certificate

( ) University degree

( ) University postgraduate degree

Which best describes your current employment?

( ) Full time

( ) Part time

( ) Casual

( ) Full time parent/ home duties

( ) Unemployed (seeking work)

Which best describes your partner's current employment?

( ) Full time

( ) Part time

( ) Full time parent/ home duties

( ) Unemployed (seeking work)

Which best describes your family's combined annual income?

( ) <25,000

( ) 25,000-50,000

( ) 50,000-75,000

( ) 75,000-100,000

( ) 100,000-150,000

( ) 150,000+

What type of school does your child attend?

( ) State School

( ) Catholic School

( ) Independent (Private) School

( ) Special School

( ) Home schooled

( ) Other (please explain)

If your child does not attend a Special School, are they in a special class?

( ) Yes, my child is in a separate class for children with special needs

( ) No, my child is taught with the rest of his/her grade

Which grade is your child currently in?

( )   Prep

( )   1

( )   2

( )   3

( )   4

What support does your child receive at school? (Please tick all that apply)

( )   Nil

( )   Teacher Aide

( )   Learning support teacher/Specialist teacher

( )   School Nurse

( )   Occupational Therapy

( )   Physiotherapy

( )   Speech Pathology

( )   Academic/ Guidance Officer

( )   Other (please explain)

Does your child have an individual education plan (IEP – an individualised plan for your child's education)?

( ) Yes

( ) No

Does your child receive any other assistance with schooling (e.g. private tuition)?

( ) Yes, please describe

( ) No

Which of the following extra-curricular activities (outside of school) does your child participate in? (please tick all that apply). Please indicate for each activity, the number of times your child would participate in the activity (either formal lessons and/or practice:

Extra-curricular Activities	Frequency
( ) Nil	
( ) Music Classes (e.g. singing, instrument)	( ) Monthly ( ) Weekly ( ) 2-3 times/wk
( ) Creative Arts Classes (e.g. drama, drawing)	( ) Monthly ( ) Weekly ( ) 2-3 times/wk
( ) Team Sport (e.g. soccer, cricket)	( ) Monthly ( ) Weekly ( ) 2-3 times/wk
( ) Individual Sport (e.g. swimming, martial arts)	( ) Monthly ( ) Weekly ( ) 2-3 times/wk
( ) Dance (e.g. ballet, hip hop)	( ) Monthly ( ) Weekly ( ) 2-3 times/wk
( ) Social Clubs (e.g. scouts, girl guides)	( ) Monthly ( ) Weekly ( ) 2-3 times/wk
( ) Religious classes or clubs (e.g. Sunday school)	( ) Monthly ( ) Weekly ( ) 2-3 times/wk
( ) Other (please explain)	( ) Monthly ( ) Weekly ( ) 2-3 times/wk

Appendix 6: Qld PREDICT CP Study Health Resource Use Form

Allied Health

During the last 6 months, have you received….

1. Physiotherapy Yes No

Does it emphasise on Motor learning Functional therapy NDT therapy Postural management

How often weekly fortnightly monthly others (________)

Duration of session 30 minutes 45 minutes 60 minutes others (________)

Format individual group others (________)

Compliance very poor poor average good very good

2. Occupational therapy Yes No

Does it emphasise on CIMT therapy Goal directed training Postural management

How often weekly fortnightly monthly others (________)

Duration of session 30 minutes 45 minutes 60 minutes others (________)

Format individual group others (________)

Compliance very poor poor average good very good

3. Speech therapy Yes No

How often weekly fortnightly monthly others (________)

Duration of session 30 minutes 45 minutes 60 minutes others (________)

Format individual group others (________)

Compliance very poor poor average good very good

4. Conductive education Yes No

How often weekly fortnightly monthly others (________)

Duration of session 30 minutes 45 minutes 60 minutes others (________)

Format ☐ individual ☐ group ☐ others (________)

Compliance ☐ very poor ☐ poor ☐ average ☐ good ☐ very good

5. Point percussion ☐ Yes ☐ No

How often ☐ weekly ☐ fortnightly ☐ monthly ☐ others (________)

Duration of session ☐ 30 minutes ☐ 45 minutes ☐ 60 minutes ☐ others (________)

Format ☐ individual ☐ group ☐ others (________)

Compliance ☐ very poor ☐ poor ☐ average ☐ good ☐ very good

6. Acupuncture ☐ Yes ☐ No

How often ☐ weekly ☐ fortnightly ☐ monthly ☐ others (________)

Duration of session ☐ 30 minutes ☐ 45 minutes ☐ 60 minutes ☐ others (________)

Format ☐ individual ☐ group ☐ others (________)

Compliance ☐ very poor ☐ poor ☐ average ☐ good ☐ very good

7. Other therapy (________________) ☐ Yes ☐ No

How often ☐ weekly ☐ fortnightly ☐ monthly ☐ others (________)

Duration of session ☐ 30 minutes ☐ 45 minutes ☐ 60 minutes ☐ others (________)

Format ☐ individual ☐ group ☐ others (________)

Compliance ☐ very poor ☐ poor ☐ average ☐ good ☐ very good

8. Other therapy (________________) ☐ Yes ☐ No

How often ☐ weekly ☐ fortnightly ☐ monthly ☐ others (________)

Duration of session ☐ 30 minutes ☐ 45 minutes ☐ 60 minutes ☐ others (________)

Format ☐ individual ☐ group ☐ others (________)

Compliance ☐ very poor ☐ poor ☐ average ☐ good ☐ very good

9. Other therapy (________________) ☐ Yes ☐ No

How often ☐ weekly ☐ fortnightly ☐ monthly ☐ others (________)

Duration of session ☐ 30 minutes ☐ 45 minutes ☐ 60 minutes ☐ others (________)

Format ☐ individual ☐ group ☐ others (________)

Compliance ☐ very poor ☐ poor ☐ average ☐ good ☐ very good

10. Therapy Packages ☐ Yes ☐ No

Post BTX-A therapy package ☐ Yes ☐ No (see attached table)

Post surgery package ☐ Yes ☐ No (see attached table)

Compliance ☐ very poor ☐ poor ☐ average ☐ good ☐ very good

Medical

During the last 6 months, has your child had….

1. Hospital admission ☐ Yes ☐ No ☐ Number

Visit 1 Reason: ____

Length of stay: ____

Investigations performed and treatment received: __________________________

____

Visit 2 Reason: ____

Length of stay: ____

Investigations performed and treatment received: __________________________

____

Visit 3 Reason: ____

Length of stay: ____

Investigations performed and treatment received: __________________________

____

Visit 4 Reason: ____

Length of stay: ____

Investigations performed and treatment received: ____________________________

____

2. Specialist medical appointments Yes No

Reason: ____

Length of stay: ____

Investigations performed and treatment received: _____________________________

____

3. Other out-patient appointments Yes No

Reason: ____

Length of stay: ____

Investigations performed and treatment received: _____________________________

____

Medication

During the last 6 months, has your child had….

1. Medication Yes No

For asthma Yes No

Which medication: ____

Frequency (number of times per day): ____

Dosage (per day): ____

Duration (length of treatment): ____

Any adverse events ☐ Yes ☐ No

Required admissions to hospital (see ‘Medical’) ☐ Yes ☐ No

2. For epilepsy ☐ Yes ☐ No

Frequency (number of times per day): ____

Dosage (per day): _________________________

Duration: ____

Any adverse events ☐ Yes ☐ No

Required admissions to hospital (see ‘Medical’) ☐ Yes ☐ No

3. For saliva control ☐ Yes ☐ No

Frequency (number of times per day): ____________________

Dosage (per day): _________________________

Duration: ____

Any adverse events ☐ Yes ☐ No

Required admissions to hospital (see ‘Medical’) ☐ Yes ☐ No

4. Others: ____

Frequency (number of times per day): ____________________

Dosage (per day): __________________________

Duration: ____

Any adverse events ☐ Yes ☐ No

Required admissions to hospital (see ‘Medical’) ☐ Yes ☐ No

Spasticity management/ muscle contracture management

During the last 6 months, has your child received….

1.

BTX-A ☐ Yes ☐ No

No of limbs treated ☐ 1 ☐ 2 ☐ 3 ☐ 4 ☐ others (____)

No. of muscles per limb ☐ calf ☐ hamstrings ☐ adductors ☐ UL ☐ others (____)

Total dose (units BOTOX or Dysport): ____

Body weight at Rx date: ____

Units/ kilogram/ body weight: ____

Any adverse events ☐ Yes ☐ No

Required admissions to hospital (see 'Medical') ☐ Yes ☐ No

2.

Phenol ☐ Yes ☐ No

No of limbs treated ☐ 1 ☐ 2 ☐ 3 ☐ 4 ☐ others (____)

Obturator Nerve ☐ No

☐ Yes ☐ direct ☐ indirect ☐ other site (________)

Total dose: ____

Any adverse events ☐ Yes ☐ No

Required admissions to hospital (see 'Medical') ☐ Yes ☐ No

3.

Oral anti-spastic medication ☐ Yes ☐ No

What medications: ____

Dosage (per day): ____

Duration: ____

Any adverse events ☐ Yes ☐ No

Required admissions to hospital (see 'Medical') ☐ Yes ☐ No

4.

Intrathecal Balcofen	☐ Yes	☐ No			

Dosage (per day): ____

Pump refill	☐ Yes	☐ No			
Any adverse events	☐ Yes	☐ No			

Required admissions to hospital (see ‘Medical’) ☐ Yes ☐ No

5.

Soft tissue release	☐ Yes	☐ No			
What level	☐ Hip	☐ knee	☐ ankle	☐ foot	☐ spine
Which side	☐ unilateral	☐ bilateral			

6.

Bony reconstruction-derotational osteotomy	☐ Yes	☐ No			
What level	☐ Hip	☐ knee	☐ ankle	☐ foot	☐ spine
Which side	☐ unilateral	☐ bilateral			

7.

Salvage procedure	☐ Yes	☐ No			
What level	☐ Hip	☐ knee	☐ ankle	☐ foot	☐ spine
Which side	☐ unilateral	☐ bilateral			

8.

SEMLs	☐ Yes	☐ No			
What level	☐ Hip	☐ knee	☐ ankle	☐ foot	☐ spine
Which side	☐ unilateral	☐ bilateral			

Any adverse events ☐ Yes ☐ No

Required admissions to hospital (see 'Medical') ☐ Yes ☐ No

9.

Rhizotomy ☐ Yes ☐ No

Date and number of rootlets cut: ____

Which side ☐ unilateral ☐ bilateral

Any adverse events ☐ Yes ☐ No

Required admissions to hospital (see 'Medical') ☐ Yes ☐ No

10.

Serial casting ☐ Yes ☐ No

No of episodes ☐ 1 ☐ 2 ☐ 3 ☐ others (_______)

No. of limbs treated ☐ 1 ☐ 2

Total no of weeks done ☐ 1 ☐ 2 ☐ 3 ☐ 4 ☐ >4

Any adverse events ☐ Yes ☐ No

Required admissions to hospital (see 'Medical') ☐ Yes ☐ No

Equipment

During the last 6 months, have you received….

1.

Wheelchair ☐ Yes ☐ No

Is it ☐ New ☐ Continuing

Frequency of use hours per day: ______________________________

days per week: ______________________________

Compliance ☐ very poor ☐ poor ☐average ☐ good ☐ v. good

2.

Sticks	☐ Yes	☐ No			
Is it	☐ New	☐ Continuing			
Frequency of use	hours per day: ________________________________				
	days per week: ________________________________				
Compliance	☐ very poor	☐ poor	☐average	☐ good	☐ v. good

3.

Crutches	☐ Yes	☐ No			
Is it	☐ New	☐ Continuing			
Frequency of use	hours per day: ________________________________				
	days per week: ________________________________				
Compliance	☐ very poor	☐ poor	☐average	☐ good	☐ v. good

4.

Kaye Walker	☐ Yes	☐ No			
Is it	☐ New	☐ Continuing			
Frequency of use	hours per day: ________________________________				
	days per week: ________________________________				
Compliance	☐ very poor	☐ poor	☐average	☐ good	☐ v. good

5.

Pony Walker	☐ Yes	☐ No			
Is it	☐ New	☐ Continuing			
Frequency of use	hours per day: ________________________________				
	days per week: ________________________________				

Compliance ☐ very poor ☐ poor ☐average ☐ good ☐ v. good

6.

David Hart Walker ☐ Yes ☐ No

Is it ☐ New ☐ Continuing

Frequency of use hours per day: ___________________________________

days per week: ___________________________________

Compliance ☐ very poor ☐ poor ☐average ☐ good ☐ v. good

7.

Special seating ☐ Yes ☐ No

What brand: ____

Is it ☐ New ☐ Continuing

Frequency of use hours per day: ___________________________________

days per week: ___________________________________

Compliance ☐ very poor ☐ poor ☐average ☐ good ☐ v. good

8.

Standing frame ☐ Yes ☐ No

What brand: ____

Is it ☐ New ☐ Continuing

Frequency of use hours per day: ___________________________________

days per week: ___________________________________

Compliance ☐ very poor ☐ poor ☐average ☐ good ☐ v. good

9.

Orthoses ☐ Yes ☐ No

Is it	□ New	□ Continuing			
Is it	□ fixed AFO	□ hinged AFO		□ KAFO	□ HKFO
	□ unilateral		□ bilateral		
	□ hip abduction brace (static or SWASH)				
	□ Soft garment/ sleeves/ UPsuit				
	□ Night time positioning equipment (list brand_____________)				
Which side	□ unilateral	□ bilateral			
Frequency of use	hours per day: ________________________________				
	days per week: ________________________________				
Compliance	□ very poor	□ poor	□average	□ good	□ v. good
