# Supplementary material for: PREDICT-CP: study protocol of implementation of comprehensive surveillance to predict outcomes for school-aged children with cerebral palsy
Source: BMJ Open. 2017 Jul 12;7(7):e014950. doi: 10.1136/bmjopen-2016-014950 (PMC5734266; doi:10.1136/bmjopen-2016-014950)
Supplement: Author's manuscript [file bmjopen-2016-014950.draft_revisions.pdf]

PREDICT CP: Study Protocol of implementation of comprehensive surveillance to Predict outcomes for school aged children with Cerebral Palsy

Journal:	*BMJ Open*
Manuscript ID	bmjopen-2016-014950
Article Type:	Protocol
Date Submitted by the Author:	17-Feb-2017

Complete List of Authors:	Boyd, Roslyn; The University of Queensland, Queensland Cerebral Palsy and Rehabilitation Research Centre; The University of Queensland, Queensland Children's Medical Research Institute<br>Davies, Peter; The University of Queensland, Children's Nutrition Research Centre<br>Ziviani, Jenny; Children's Health Queensland Hospital and Health Service, Allied Health Research; The University of Queensland, School of Health and Rehabilitation Science<br>Trost, Stewart; Queensland University of Technology, Institute of Health and Biomedical Innovation, Centre for Children's Health Research<br>Barber, Lee; The University of Queensland, Queensland Cerebral Palsy and Rehabilitation Research Centre, School of Medicine, Faculty of Medicine and Biomedical Sciences<br>Ware, Robert; The University of Queensland, School of Population Health<br>Rose, Stephen; CSIRO Australian e-Health Research Centre, Neuroimaging<br>Whittingham, Koa; The University of Queensland, Queensland Cerebral Palsy and Rehabilitation Research Centre<br>Sakzewski, Leanne; The University of Queensland, Queensland Cerebral Palsy and Rehabilitation Research Centre ; Children's Health Queensland, Children's Allied Health Research<br>Bell, Kristie; The University of Queensland, Queensland Cerebral Palsy and Rehabilitation Research Centre; The University of Queensland, Queensland Children's Medical Research Institute<br>Carty, Chris; Griffith Univ, Biomechanics<br>Obst, Steven ; The University of Queensland, Queensland Cerebral Palsy and Rehabilitation Research Centre<br>Benfer, Katherine; The University of Queensland, Queensland Cerebral Palsy and Rehabilitation Research Centre<br>Reedman, Sarah; The University of Queensland, School of Medicine, Queensland Cerebral Palsy and Rehabilitation Research Centre<br>Edwards, Priya; The University of Queensland, Queensland Cerebral Palsy and Rehabilitation Research Centre; Children's Health Queensland Hospital and Health Service, Queensland Paediatric Rehabilitation Service<br>Kentish, Megan; Children's Health Queensland Hospital and Health Service, Queensland Paediatric Rehabilitation Service<br>Copeland, Lisa; Children's Health Queensland Hospital and Health Service, Queensland Paediatric Rehabilitation Service<br>Weir, Kelly ; Gold Coast University Hospital, Speech Pathology<br>Davenport, Camilla; The University of Queensland, Queensland Cerebral Palsy and Rehabilitation Research Centre

	Brooks, Denise ; The University of Queensland, Queensland Cerebral Palsy and Rehabilitation Research Centre<br>Coulthard, Alan; The University of Queensland, University of Queensland, Faculty of Medicine<br>Pelekanos, Rebecca; The University of Queensland, Queensland Cerebral Palsy and Rehabilitation Research Centre<br>Guzzetta, Andrea; Stella Maris Institute, Neurology<br>Fiori, Simona; Stella Maris Institute, Neurology<br>Wynter, Meredith; Children's Health Queensland Hospital and Health Service, Queensland Paediatric Rehabilitation Service<br>Finn, Christine; Children's Health Queensland Hospital and Health Service, Queensland Paediatric Rehabilitation Service; The University of Queensland, Queensland Cerebral Palsy and Rehabilitation Research Centre<br>Burgess, Andrea; The University of Queensland, Queensland Cerebral Palsy and Rehabilitation Research Centre<br>Morris, Kym; The University of Queensland, Queensland Cerebral Palsy and Rehabilitation Research Centre<br>Walsh, John; Mater Medical Research Institute, Orthoapedics; Children's Health Queensland Hospital and Health Service, Department of Orthopaedics<br>Lloyd, Owen; Children's Health Queensland Hospital and Health Service, Queensland Paediatric Rehabilitation Service<br>Whitty, Jennifer; University of East Anglia Norwich Medical School, School of Pharmacy<br>Scuffham, Paul; Griffith University, Griffith Health Institute and School of Medicine

**Primary Subject Heading**:	Paediatrics
Secondary Subject Heading:	Neurology, Paediatrics, Rehabilitation medicine
Keywords:	Cerebral Palsy, Longitudinal cohort, brain structure and function, NUTRITION & DIETETICS, manual ability, communication

SCHOLARONE™
Manuscripts

Article type: Study Protocol

Title: PREDICT CP: Study Protocol of implementation of comprehensive surveillance to Predict outcomes for school aged children with Cerebral Palsy

Author List:

Roslyn Boyd,[1,4] Peter SW Davies,[2] Jenny Ziviani,[3, 4] Stewart Trost,[5] Lee Barber,[1] Robert Ware,[6] Stephen Rose,[14] Koa Whittingham,[1] Leanne Sakzewski, [1,4] Kristie Bell,[1,4], Christopher Carty,[9] Steven Obst,[1] Katherine Benfer,[1] Sarah Reedman,[1] Priya Edwards,[4] Megan Kentish,[4] Lisa Copeland,[4] Kelly Weir,[1, 10,16] Camilla Davenport,[1,2] Denise Brookes,[2] Alan Coulthard,[11] Rebecca Pelekanos,[12] Andrea Guzzetta,[15] Simona Fiori,[15]Meredith Wynter,[4] Christine Finn,[1] Andrea Burgess,[1] Kym Morris,[1] Owen Lloyd,[4] John Walsh,[9,13] Jennifer A. Whitty,[8] Paul A Scuffham, [16]

Author Affiliations:

[1]Queensland Cerebral Palsy and Rehabilitation Research Centre (QCPRRC), Child Health Research Centre, The University of Queensland, Centre for Children's Health Research, Faculty Medicine, The University of Queensland, Brisbane.

[2]Children's Nutrition Research Centre (CNRC), Child Health Research Centre, Centre for Children's Health Research, Faculty of Medicine, The University of Queensland, Brisbane.

[3]School of Health and Rehabilitation Sciences, The University of Queensland.

[4]Queensland Paediatric Rehabilitation Service, Children's Health Queensland, Lady Cilento Children's Hospital, Brisbane, QLD.

[5]Institute of Health and Biomedical Innovation, Queensland University of Technology

[6]UQ Child Health Research Centre, The University of Queensland

[7]CSIRO Mathematical and Information Sciences Biomedical Imaging Group, Australian e-Health Research Centre.

[8] Norwich Medical School, University of East Anglia, Norwich UK and School of Pharmacy, The University of Queensland

[9]Queensland Children's Motion Analysis Service, , Children's Health Queensland

[10]Clinical Governance, Education and Research, Gold Coast Health Service.

For peer review only

For peer review only - http://bmjopen.bmj.com/site/about/guidelines.xhtml

[11]Medical Imaging, Royal Brisbane and Women's Hospital, Faculty of Medicine, The University of Queensland

[12]University of Queensland Centre for Clinical Research

[13]Department of Paediatric Orthopaedics, the Mater Health Services, Brisbane.

[14]Medical Imaging, Diagnostic and Interventional Neuroradiology Department at the Royal Brisbane and Women's Hospital, Queensland Health, Brisbane.

[15] Department of Developmental Neuroscience, Instituto Di Ricovero E Cura A Carattere Scientifico (IRCCS), Stella Maris, University of Pisa, Italy;

[16] Menzies Health Institute Queensland, Griffith University;

[17] Queensland Centre for Intellectual and Developmental Disability, The University of Queensland;

Corresponding Author:

Professor Roslyn Boyd, PhD, Scientific Director,

Queensland Cerebral Palsy and Rehabilitation Research Centre,
Level 6, Children's Health Research Centre, The University of Queensland,
62 Graham Street, South Brisbane. QLD 4101, Australia.
E-Mail: r.boyd@uq.edu.au; Phone: +61 (07) 3069 7372 Fax: +61 (07) 3069 7109

Total Word Count: 11,480

Key words (5 max): Cerebral Palsy, Longitudinal cohort, Motor development, Brain Structure and Function, Communication, Gross Motor Function, Manual Ability,

For peer review only

ABSTRACT

Objectives

Cerebral Palsy (CP) remains the world's most common childhood physical disability with total annual costs of care and lost wellbeing of AU$3.87b. The Predict-CP study will investigate the influence of brain structure, body composition, dietary intake, oropharyngeal function, habitual physical activity, musculoskeletal development (hip status, bone health), and muscle performance on motor attainment, cognition, executive function, communication, participation, quality of life and related health resource use costs. The Predict-CP cohort provides further follow-up at 8-11 years of two overlapping preschool-age cohorts examined from 2-5 years (NHMRC465128 motor and brain development; NHMRC569605 growth, nutrition and physical activity).

Methods and Analyses

This population based cohort study undertakes state-wide surveillance of 245 children with CP born in Queensland (birth years 2006-2009). Children will be classified for Gross Motor Function Classification System (GMFCS); Manual Ability Classification System (MACS), Communication Function Classification System (CFCS) and Eating and Drinking Ability Classification System (EDACS). Outcomes include gross motor function, musculoskeletal development (hip displacement, spasticity, muscle contracture), upper limb function, communication difficulties, oropharyngeal dysphagia, dietary intake and body composition, participation, parent and child reported quality of life and medical and allied health resource use. These detailed phenotypical data will be compared to brain macro and micro structure using 3 Tesla Magnetic Resonance Imaging (3T MRI). Relationships between brain lesion severity and outcomes will be analysed using multilevel mixed-effects models.

Ethics and Dissemination

The PREDICT CP protocol is a prospectively registered and ethically accepted study protocol. The study combines data at 2-5 then 8-11 years of direct clinical assessment to enable prediction of outcomes and health care needs essential for tailoring interventions (e.g. rehabilitation, orthopaedic surgery and nutritional supplements) and the projected health care utilisation.

ANZTR Trial Registration Number: ACTRN 12616001488493

Strengths and limitations of this study:

- The Predict prospective cohort study provides comprehensive phenotypical data on a representative cohort of children with CP.
- The longitudinal follow-up of this cohort (at 2-5 years and now cross sectional at 8-11 years) will enable development of prediction models of outcome.

For peer review only

- Brain structure (macro and micro structure at 3.0 tesla) will be compared comprehensive motor, cognitive and communication outcomes at school age.
- A limitation is that only brain macrostructure at 1.5T has been captured from early clinical brain MRI scans as part of clinical practice.

For peer review only

BACKGROUND

Cerebral Palsy (CP) is a disorder of movement and posture secondary to an insult to the developing brain [1]. The insult is static and permanent and may be the consequence of different factors, including both genetic and environmental causes. Although the insult is static, the consequent symptoms are variable and may change over time [2]. The disability increases with age and ageing occurs earlier [3]. Children may have a range of comorbidities [4], which are likely to impact outcomes and costs of care [5]. Based on CP registers, a recent systematic review identified that in children diagnosed with CP at 5 years: 3 in 4 were in pain; 1 in 2 had an intellectual disability; 1 in 3 could not walk; 1 in 3 had hip displacement; 1 in 4 could not talk; 1 in 4 had epilepsy; 1 in 4 had a behaviour disorder; 1 in 4 had bladder control problems; 1 in 5 had a sleep disorder; 1 in 5 dribbled; 1 in 10 were blind; 1 in 15 were tube fed; and 1 in 25 were deaf [6]. It is known that peak motor attainment in CP is reached at 8-9 years and tends to plateau before a decline in adolescence [3]. Secondary musculoskeletal disorders involving muscle, tendons, bones and joints are common as a result of spasticity, muscle weakness and immobility. Cerebral Palsy has substantial lifelong effects on daily function, societal participation and quality of life (QOL) for children and their families. There is a paucity of data on the relationship between physical outcomes and school attainment [7]. Better prediction of outcomes is important for families and health care providers [8].

In Australia, CP remains the most common physical disability in children with ≈ 700 infants born each year that will be later diagnosed with CP [9]. The overall costs to society of persons with CP was AU$1.47b per year (0.14% of GDP), with an average annual cost of AU$43,431 per individual [10]. When taking into account the value of lost wellbeing (disability and premature death) the total costs were AU$3.87b per year or $115,000 per person. Cerebral Palsy has a lifetime impact at a total cost of over AU$2M per person [10]. More recently, in a preschool-aged cohort (CP-Child, National Health and Medical Research Council NHMRC 465128) we have determined a strong relationship between severity of Gross Motor Function Classification System (GMFCS) levels I-V and a stepwise increase in incremental costs of care [5].

The ability to better predict outcomes has the potential to guide intervention to reduce adverse outcomes (hip dislocation, poor growth, under or over nutrition, respiratory health complications from oropharyngeal dysphagia, pain, reduced participation in the community and under attainment at school). Development of prediction models based on early brain structure and function can inform health and social care provision (for example, via the National Disability Insurance Scheme, N.D.I.S.) and provide best practice comprehensive surveillance to allow implementation of timely and effective interventions to achieve optimal outcomes.

Understanding the relationship between specific brain Magnetic Resonance Imaging (MRI) appearance and outcome measures such as motor function is critically important [11]. Such data may prove invaluable in providing accurate prognostic counselling at the time of diagnosis, as well as potentially guiding the most appropriate treatments tailored to each individual's pattern of CP and type and severity of the brain lesion on imaging [12]. A focus of the majority of epidemiological research is the prevention of CP, which requires

For peer review only - http://bmjopen.bmj.com/site/about/guidelines.xhtml

clinical outcomes to be correlated with the presumed timing and aetiology of lesions in the developing brain [11]. Pathological insults in the developing brain cause abnormalities or lesions, which may be detected by brain MRI, and the patterns of these lesions depend on the stage and/or presumed timing of the injury during brain development [13]. Using this principle, a qualitative system of classification is established whereby lesions can be identified as brain maldevelopments (occurring in the 1st and 2nd trimesters) [11], periventricular white matter lesions (occurring early in the 3rd trimester and in preterm infants), or grey matter lesions (occurring late in the 3rd trimester and at term) [11]. A systematic review found studies with enough MRI data for subjects to be classified into these presumed lesion timing groups, and in the majority of studies this lesion timing classification was able to be linked to at least one measure of motor outcome [11.] There were however limited data on brain lesion severity, brain microstructure and quantitative comprehensive outcomes [11].

In the Australian CP-child study entire birth years of Victorian and Queensland born children with CP across the full spectrum of gross motor abilities were prospectively followed to determine the relationship between the rate and limit of motor development (gross and fine motor function) as related to the nature of the brain lesion [12 14]. Representative population-based data has been reported on i) early development and prediction of hip outcomes [15], ii) the relationship between brain structure and motor development[12], and iii) social function [16] and communication [17] with cost and health resource use data across the spectrum of functional severity [5]. The cross sectional domains of school readiness (mobility, self-care, social function, communication) were reported at school entry [16 18].

In CP there is a likely relationship between the severity of the early brain injury on structural MRI (nature, extent, presumed timing), early motor status at 3 years, and later outcomes at 8-11 years (motor attainment, musculoskeletal performance, hip displacement). In Sweden, Norway, and Scotland a population wide surveillance program (CP-UP) has been implemented for up to 10 years [19]. Since implementation in Southern Sweden no child with CP has had a dislocated hip [19], musculoskeletal contractures have been reduced [20] and nutrition and bone health are monitored [21 22]. National hip surveillance best practice guidelines have been developed and implemented in Australia [23], and in Queensland population wide hip surveillance has been implemented [24].

The Predict CP study will undertake further comprehensive follow-up of four birth years of children with CP born in Queensland to capture longitudinal data on growth and physical outcomes (motor capacity, muscle and bone health, physical activity, feeding and oropharyngeal function, nutrition), cognition (executive function, educational attainment, communication), and participation, quality of life, pain and relate these to costs of health care utilisation. The quantitative evaluation of early brain structure on MRI and functional status at 2 years will be compared to these comprehensive outcomes at 8-11 years to build prediction models of CP. Development and implementation of prediction models of outcomes are essential for tailoring interventions (rehabilitation, medical management, orthopaedic surgery, nutritional supplements) and in understanding the likely costs of health care.

For peer review only - http://bmjopen.bmj.com/site/about/guidelines.xhtml

For peer review only

Growth, nutrition and physical activity are important determinants of health outcomes in children with CP. Knowledge of levels and patterns of Habitual Physical Activity (HPA) for children with CP are important as they have increased risk of inactivity (sedentary behaviour) related illness [9 25]. In addition, poor nutrition and growth may have a secondary impact on body composition, bone health and brain maturation, as well as participation and Health Related Quality of Life (HRQOL) in later childhood. In our overlapping CP-child study of growth, nutrition and physical activity (NHMRC 569605) [26] we have determined at preschool-age the i) energy requirements, body composition, dietary intake [27-29], ii) validation of HPA cut-points [30], iii) validation of a modified 3-day weighed food record for the assessment of energy intake [29] and determined iv) oropharyngeal dysphagia (OPD) across the spectrum of functional severity [31 32].

Our early data on nutritional status [29] used gold standard measures (doubly labelled water) to determine the energy requirements of preschool-aged children with CP compared to age matched children with typical development (TD) [28]. Children who were GMFCS III-V had energy requirements 18% lower than ambulant children and 31% lower than children with TD [28], with no differences between ambulant children with CP and children with TD. In addition, energy intake was related to fat free mass index in both children with CP and children with TD [29]. Associations were identified between OPD, energy intake and nutritional status after GMFCS level has been taken into account. At preschool age, OPD was originally reported in 85% of our cohort, with a significantly greater proportion of OPD with each increase in GMFCS level [31]. Following further testing of OPD psychometrics, with the inclusion of a typically developing reference sample, modified cut points were developed resulting in a revised estimate of 56% [33]. Children on full oral intakes that required modification (texture or additional energy and protein) were most at risk of poor growth and nutritional status [31].

Habitual Physical Activity accelerometer cut-points have been determined for sedentary and active behaviour in toddlers with CP [34], demonstrating that HPA levels are highly variable within GMFCS levels particularly GMFCS I-II [35].The musculoskeletal development of children with CP has focused on how spasticity interferes with normal muscle growth, and contributes to reduced joint range of motion, increased joint stiffness, and muscle weakness [36]. These factors lead to fixed contractures of the muscle-tendon unit and skeletal deformity that may require orthopaedic surgery [37]. These secondary alterations progress with age [38] and contribute to reduced gait speed, increased joint pain and falling, culminating in reduced HPA [39]. Muscle adaptations begin early[37] and compared to children with TD vary in the following ways: i) muscle volume is reduced [36 40 41]; ii) muscle fascicles are stiffer when passively stretched [40]; iii) muscle fascicles cannot stretch to lengths more favourable for force production [42]; and iv) the Achilles tendon is longer [42 43]. This effectively means the ability of muscle to generate force is reduced in children with CP. In ambulant children (GMFCS I-III) the calf muscle (gastrocnemius/soleus) has a major role in forward propulsion during walking/running [44] and structural/functional adaptations are a cause of gait limitations [45]. Characteristics of muscle structural/functional adaptations also vary according to uni/bilateral motor distribution [46]. Lower limb treatments (casting, intramuscular Botulinum toxin A injections) aim to manage these adaptations in the preschool years however multi-level orthopaedic surgery is often required at functional attainment (8-11

For peer review only - http://bmjopen.bmj.com/site/about/guidelines.xhtml

years) according to a child's gait profile [47 48]. A gait profile of ambulant children (GMFCS I-III) combined with muscle properties would provide important information for surgical decision making and prediction of functional outcome. Examination and surveillance of the relationship between muscle structure/function and gait profile to functional capacity/performance, physical activity, bone health, nutritional status and health care costs would provide vital information for structuring management plans into later childhood.

The broad aim of the CP-Child studies is to implement population based comprehensive surveillance of children with CP from early diagnosis (at 2-3 years) based on brain structure and function (early gross and fine motor, growth, nutrition, HPA, musculoskeletal development) to predict comprehensive outcomes at school age (8-11 years), a time of definitive motor maturation, walking ability, need for orthopaedic intervention and educational attainment. In this extended follow-up of two previous overlapping prospective population based CP cohorts (followed from 18-24 months corrected age (c.a.) to 5 years) across the full spectrum of functional severity (NHMRC 465128 [14]; NHMRC 569605 [26]) we will re-examine the relationship to severity of brain structure at 8-11 years on diffusion MRI (dMRI in a 3.0T MRI scanner). At 8-11 years health care utilisation is likely to be different to preschool-age so that associations between health resource use and a beneficial health/social outcome will be re-evaluated.

Aims and hypotheses

The Predict-CP study will undertake comprehensive state-wide surveillance (in Queensland) of four birth years of a representative population based cohort of children with CP. The relationship between brain structure on growth and physical outcomes (motor capacity, muscle and bone health, physical activity, oropharyngeal function, nutrition), cognition (executive function, educational attainment, communication), and participation (Habitual Physical Activity, Quality of Life, pain and sleep), will be related to educational attainment and health resource use costs.

Hypotheses

1. The location, extent of the brain lesion(s) on semi-quantitative MRI (at 2 years) and early motor capacity and performance (2-3 years) will predict severity of motor capacity Gross Motor Function Measure (GMFM-66) and performance (6 minute walk test, 6MWT), Pediatric Evaluation of Disability Inventory Computer Adaptive Test (PEDI-CAT) at 8-11 years.
2. The rate and limit of gross motor and fine motor development (GMFM-66, Assisting Hand Assessment (AHA), Both Hands Assessment (BoHA)), at 8-11 years will be influenced by the severity of musculoskeletal deformity (i.e. slower development will correlate with increased spasticity/contracture, poor muscle function, marked hip displacement, pain, reduced sleep, reduced manual ability).
3. Cognition, executive function, communication and educational attainment will be related to brain lesion severity (location, extent of the brain lesions) on semi-quantitative MRI but not gross and fine motor capacity (GMFCS, MACS) at 8-11 years.
4. Nutritional status (under/overweight), OPD, body composition (fat free mass and fat mass via Dual energy X-ray Absorptiometry (DXA)), Habitual Physical Activity (HPA), growth velocity and bone

For peer review only - http://bmjopen.bmj.com/site/about/guidelines.xhtml

health will be related to the level of GMFCS attainment and will predict: i) higher health care utilisation and direct medical costs; ii) lower levels of participation in school, leisure and community, and iii) poorer HRQOL.

Study Significance

For children with CP this unique project will:

1. Quantify the impact of functional severity on medical resource use to inform service provision planning at school age (a period of intensive medical and orthopaedic treatments). From earlier sampling of these cohorts (NHMRC 465128/569605) we have detailed information on the content, dose and compliance, adverse events, medical, surgical and allied health resource use (interventions, medications, equipment) and consequences of outcome (from the age of 1.5 till 5 years). By study completion we will have life-time data on all interventions from age 1.5 'till 8-11 years, with regular assessments of their functional status/ outcomes allowing predictive modelling of outcomes for children with CP.
2. This project will provide school-age follow-up of this comprehensively studied cohort enabling: i) prediction of outcome (brain structure and multiple outcomes); ii) prognostication on functional, cognitive, communication for school attainment; iii) risk factors for musculoskeletal problems (i.e. hip, spine deformity and need for surgery); and iv) health outcomes due to sedentary behaviour, body composition, dietary intake and OPD.
3. Highlight the contribution of poor dietary intake, low levels of HPA, and reduced bone health on growth, body composition and fracture risk, taking into account the severity of disability.
4. Define the relationship between HPA levels, motor capacity and muscle performance to predict eventual functional attainment and community performance.

As CP remains the most common childhood physical disability with high lifetime costs, models to predict outcomes and costs of care will inform health provision, social care and tailor data for national funding schemes such as the Australian National Disability Insurance Scheme (NDIS).

METHODS

All children diagnosed with CP, born between 1st January 2006 and 31st December, 2009 in Queensland will be invited to participate. These children have participated in two prospective longitudinal cohort studies between the ages of 1.5 to 5 years and will now be invited to return at 8-11 years. For inclusion criteria Cerebral Palsy is defined as a permanent (but not unchanging) disorder of movement and posture that results from an insult to the developing central nervous system. The characteristic signs are spasticity, movement disorders, muscle weakness, ataxia and rigidity[14].

Exclusion criteria

1. Children with a progressive or neurodegenerative lesion.
2. Children born outside Queensland in the relevant birth years.

Ethics Approvals

Ethics committee approvals were obtained for the Australian CP child study through The Royal Children's Hospital Melbourne Ethics Committee, (HREC/25010 F), Southern Health Human Research Ethics

For peer review only - http://bmjopen.bmj.com/site/about/guidelines.xhtml

Committee C (05077C), University of Queensland Medical Research Ethics Committee (2007001784), the Children's Health Services District Ethics Committee (HREC/07/QRCH/107), the Mater Health Services Human Research Ethics Committee (1186C), the Queensland Cerebral Palsy Register at the Cerebral Palsy League of Queensland (CPLQ 2008/ 09-1010), Gold Coast Health Service District Human Research Ethics Committee (HREC/08/QGC/45), Central Queensland Health Services District Human Research Ethics Committee (HREC/08/QCQ/19), Cairns and Hinterland Health Service District Human research Ethics Committee (HREC/08/QCHHS/521) and the Townsville Health Service District Human Research Ethics Committee (HREC/08/QTHS/33). Subsequent ethics approvals have been obtained for the PREDICT study from the University of Queensland Medical Research Ethics Committee (2007001784) and the Children's Health Services District Ethics Committee (HREC/07/QRCH/107).

There are no known health or safety risks associated with participation in any aspect of the described study. All radiological tests (including AP pelvis, spine as required) and full body and lateral distal femur DXA for body composition and bone health have been reviewed for radiation safety. All families will give written informed consent to participate, and they are able to withdraw their child from the study at any time without explanation, without any penalty from staff at Children's Health Queensland, or any effect on their child's care. Data collected in this study will be stored in a coded re-identifiable form (by ID number).

Ascertainment of the cohort

Prospective entry of birth years Queensland (born in 2006, 2007, 2008, 2009) whom were entered at 18 months and followed until school age (5 years) (n=245) in the Australian CP child study will be invited to participate in the Predict-CP follow-up study. State-wide recruitment was established in collaboration with the Queensland Cerebral Palsy Register with data collection at tertiary referral hospitals. In cases where the diagnosis of CP was unclear, or where there is a suggestion of a progressive or degenerative course, further investigations (such as metabolic screening) were requested before a diagnosis of CP was confirmed. Children detected after 18 months of age were entered into the study at the time of diagnosis, offered brain MRI at entry and were followed up with serial motor assessments and other outcomes until confirmation of the diagnosis of CP at 5 years.

The recruited sample born in Queensland (n=245) in the birth years of 2006, 2007, 2008 and 2009 are representative of a population based sample [49]. The sample is classified according to the Gross Motor Function Classification System for 2-18 years (GMFCS), a five level classification system of children's functional gross motor severity [39]. It is based on self-initiated movements, anti-gravity postures and motor skills expected in a typical five year old [50]. Children who are independently ambulant are classified as GMFCS I or II, those requiring an assistive mobility device to walk classified as GMFCS III and those in wheeled mobility as GMFCS IV and V. The recruited sample includes children who are functioning at 5 years of age at Gross Motor Function Classification, GMFCS level I=96 (39%), II=38 (15.6%), III=38 (15.6%), IV=35 (13.6%), V=38 (15.6%); of whom 146 are male (62%), of spastic motor type 208 (86.6%), and unilateral 78 (31.8%) or bilateral 165 (68%) motor distribution (Figure 1). Children will be assessed

For peer review only - http://bmjopen.bmj.com/site/about/guidelines.xhtml

during their eighth to eleventh birth year at the Centre for Children's Health Research in Brisbane. Co-morbidities and need for medical management will be screened.

Procedures

Children and families who have participated in previous research projects (NHMRC 569605 and NHMRC 465128) and were born in Queensland will be approached to participate in the current study. After providing informed consent the child and their caregiver will be invited to attend the Children's Health Research Centre and the Lady Cilento Children's Hospital, a tertiary referral centre for a 1-2 day visit. All recent medical, surgical and neurological visits that have occurred since their last visit will be screened (from their medical records and by parent report) to confirm any changes in diagnosis of CP, differential diagnosis by neurological assessment (by a Paediatrician, Child Neurologist or Paediatric Rehabilitation Specialist). Experienced Allied Health researchers will perform all motor, upper limb, language and cognitive assessments at the visit. Physiotherapists will check range of motion, clinical measures of spasticity, then rate GMFCS, gait pattern, MACS and measure pelvic and spine radiographs where indicated according to standardized protocols [23].

Classification Measures

All children with CP at all levels of ability (GMFCS I-V) at 8-11 years will be classified for:

Functional severity

The GMFCS has internationally established validity, reliability and stability for the classification and prediction of motor function of children with CP aged 2-12 years [50-52]. It has an acceptable inter-rater and intra-rater (test-retest) reliability (generalisability coefficients 0.93 and 0.68, respectively) [51]. Two physiotherapists, trained in the use of the GMFCS, will independently observe and classify children in one of five functional categories [50].

Classifications of gross motor abilities change with age and therefore separate descriptions are used for different age bands. In the current study, the 6-12 year descriptions from the extended and revised GMFCS (GMFCS-ER) will be used [53]. The GMFCS has been correlated with a number of motor scales, as well as CP motor type and distribution [54].

Motor type and distribution

Motor type will be classified as spastic, dystonic, ataxic, hypotonic, choreoathetosis, mixed CP or unclassifiable according to Surveillance of Cerebral Palsy in Europe (SPCE) guidelines [55]. Distribution will be classified by number of limbs impaired, uni- and bi-lateral distribution (hemiplegia, diplegia, triplegia, quadriplegia) by at least two independent raters. The Dyskinesia Impairment Scale [56] will be undertaken for those participants with a motor-type (primary or secondary) diagnosis of dystonia and/or choreoathotosis. This is an important assessment to measure the motor capacity and function of children with these particular motor-types [57].

For peer review only - http://bmjopen.bmj.com/site/about/guidelines.xhtml

Functional performance

The Functional Mobility Scale (FMS) [58] at 5m (home), 50m (school) and 500m (community) [58] will be used to evaluate functional performance. This is a valid and reliable measure of a child's usual walking ability at three distances (5m, 50m and 500m), representing their home, school and wider community [59].

Gait pattern

Gait patterns will be classified according to the Rodda & Graham's classifications [60], which has demonstrated validity and reliability[61]. Gait patterns for bilateral ambulant CP will be classified as either: i) True Equinus, ii) Jump Knee, iii) Apparent Equinus; or iv) Crouch Gait. For children with unilateral CP, gait patterns will be classified according to Winters, Gage and Hicks [48]. This classification considers the sagittal plane joint movements: i) type I - foot drop during swing phase (Apparent Equinus); ii) type II - persistent ankle plantarflexion (True Equinus); iii) type III - maintained plantar flexion through gait cycle plus limited knee flexion-extension; and iv) type IV - similar to III, plus reduced hip flexion-extension. Winter's classification has good inter-rater reliability using written reports (weighted kappa, wκ=0.76) and videos (wκ=0.63) [61-63].

Upper Limb Function

Upper limb function is classified using the Manual Ability Classification System (MACS) [64]. The MACS is an international system to classify hand function based on the child's typical performance when handling objects in daily activities. The MACS is a five level classification of how well children with CP use their hands to handle objects in day-to-day activities [64]. This classification system was developed for children aged from 4-18 years, and has good reliability for use in children as young as two years[64]. The MACS has reported construct validity, and excellent inter-rater reliability (ICC=0.97 between therapists and 0.96 between therapists and parents) for children with CP [65]. Children will be classified on the MACS by an occupational therapist in discussion with the child's carer.

Communication Function

Communication function will be classified on three distinct but overlapping systems:

i. Communication Function Classification System (CFCS) classifies children's performance in sending and receiving communicative messages using their typical communication means (considering all communication methods including Augmentative and Alternative Communication). It has been validated in children with CP aged 2-18 years. Reliability between professionals was moderate (κ=0.66), professional-parent fair (κ=0.49), and test-retest strong (κ=0.82) [66].
ii. Functional Communication Classification System (FCCS) classifies children's performance only in sending communicative messages, and also considers their typical communication (including all communication methods including Augmentative and Alternative Communication). It has excellent inter-rater reliability between professionals (kappa=0.94) and parent-professional (κ=0.59) [67].

For peer review only - http://bmjopen.bmj.com/site/about/guidelines.xhtml

iii. The Viking Speech Scale (VSS) will be used to classify children's speech production [68]. The VSS is a four level classification system, which can be used to classify speech intelligibility for strangers and unfamiliar conversation partners of children with CP aged 4 years and above. It has strong content validity, and moderate-substantial inter-rater reliability between pairings of speech pathologists, health care professionals and parents (kappa=0.58-0.81) [68].

Eating and drinking function

The Eating and Drinking Ability Classification System (EDACS) classifies the eating and drinking abilities of children with CP aged 3 years and above. Classification is I-V and describes children's safety and efficiency predominately focusing on food and fluid textures [69]. The EDACS has strong inter-rater reliability between professionals (ICC=0.93), but fair reliability between parings of professionals and parents (ICC=0.45) [69].

Body Structure and Function Measures

Brain structure on magnetic resonance imaging (MRI)

The American Academy of Neurology practice parameter has concluded that brain MRI should be part of the diagnosis of CP [70]. Early MRI at 0-3 years will be classified according to the nature and presumed timing of the lesion [11] and analysed for brain lesion severity on the semi-quantitative scale of Fiori[8]. Aetiology of CP will be evaluated using MRI (location, nature and structure of the brain lesion)[11]. The brain lesion will be classified by 3 main criteria:

A. the *anatomical features* of the lesion:
   a. localisation by tissue (e.g. cortical, white matter, deep grey matter etc.)
   b. localisation by region (e.g. lobes involved, laterality etc.)
   c. extent of lesion (e.g. generalised, hemispheric, lobar etc.)
B. the presumed *aetiology* of the lesion: i) genetic; ii) ischemic; iii) infective and iv) other.
C. the presumed *timing* of the insult that caused the lesion:
   a. Prenatal by trimester or by stage of brain development;
   b. Perinatal;
   c. Postnatal.

All MRIs will be classified by a neurologist (SF) together with a neuroradiologist (AC) using a standardised method of image evaluation and classification. Following these evaluations, consensus will be reached regarding the above three criteria. Based on preliminary data it is estimated that >60 percent of children currently receiving a diagnosis of CP will have had early brain MRI as part of their clinical work-up. All children (n=245) will be offered a repeat brain MRI at 8-11 years at 3T. The majority will have their imaging performed and reported through the Herston Imaging Research Facility, on a Siemens 3.0T MR scanner. The current minimum imaging protocol for patients with suspected CP consists of axial fast spin echo and coronal fast spin echo sequences and 3D inversion prepared fast spoiled GRASS sequence. 3D acquisitions

For peer review only - http://bmjopen.bmj.com/site/about/guidelines.xhtml

are reformatted in axial, coronal and sagittal planes, with additional oblique and curved reformatting. Age specific protocols are used to maximize the ability to detect cortical and white matter abnormalities at different stages of myelination. All neuroimaging will be reviewed by a neurologist (SF, AG) and a neuroradiologist (AC) familiar with the features of lesions that result in CP. This approach is consistent with a Clinical Practice guideline suggesting that all patients with the label of CP have high quality MR imaging on at least one occasion[70]. MRI scans will be performed predominantly awake, without anaesthesia and after informed consent. Preparation for the MRI will be offered to families in the form of a training DVD explaining the scanner experience and practice in a "mock scanner" (0.0Tesla) will be offered, where required.

Brain lesion severity will be assessed using a structured scoring proforma [8] based on the CH2 template [71], a highly detailed single-subject T1 template in Montreal Neurological Institute MNI space, which is the international standard for brain mapping (International Consortium of Brain Mapping - ICBM). Lesions will be transcribed onto the proforma and the following measures obtained: number of i) anatomical lobes involved, ii) number of slices on the template that were affected and iii) size and distribution of the lesion measured by a global lesion score and lesion sub-scores. The score (maximum of 40) is based on: i) anatomical lobes involved; ii) number of affected slices; and iii) size and distribution of the lesion. The number of lobes and slices affected will be the average of summed right and left hemispheres. To calculate total lesion score, each frontal, parietal, temporal and occipital lobe will be first considered in three sections: periventricular, middle and subcortical matter. Each section will be scored as 0.5 if less than 50% of area was involved; or 1, for greater than 50% involvement, with a maximum lobar score of 3. Lobar scores for each hemisphere will be summed, with a maximum hemispherical score of 12 possible. The total lesion score will be the sum of right and left hemispherical scores (maximum score of 24). A 1-point score (involved/not involved) will also be attributed to 16 anatomical structures including the corpus callosum, the cerebellum and the main subcortical structures. The final maximum score of the scale will be, thus, a maximum of 40 (24+16) [72] . The Fiori scale method has strong inter rater and intra-rater reliability [72] and strong construct validity based on dMRI and functional severity in children with unilateral CP [73].

At 8-11 years structural MRI (sMRI) and functional MRI (fMRI) guided diffusion-weighted MRI (dMRI) scans suitable for connectivity analyses will be undertaken on the 3T scanner at Herston Imaging Research Facility (or Lady Cilento Children's Hospital at 3T for children requiring general anaesthesia ≈ 5%). Diffusion-weighted MRI (dMRI) for white matter fibre tracking and whole brain connectomes will be acquired using our published protocol [74]. Structural MRI (sMRI) images will be acquired using an MPRAGE sequence at an isotropic resolution of 1 mm. dMRI data will be pre-processed to reduce image artefacts [75], and the fibre orientation distribution estimated using constrained spherical deconvolution[76]. Probabilistic tractography will be conducted using MRtrix software and connectivity matrices generated using previously described methods [74]. Quantitative diffusivity indices Fractional Anisotrophy (FA) and Mean Diffusivity (MD) will be encoded within the connectome to assess reorganisation[74]. Network based statistics (NBS) [77] will be performed between FA and MD connectomes to identify significant cortical networks associated with

For peer review only - http://bmjopen.bmj.com/site/about/guidelines.xhtml

neural reorganization. A second analysis will investigate brain maturation by comparing serial sMRI data acquired around 2 years with scans in the same children at 8-11 years to develop a predictive model of brain structure and functional outcome using spatiotemporal analysis of the longitudinal imaging data [77].

Clinical history and examination

Clinical history will be reviewed (Appendix 1: QLD CP Child Physicians Checklist) to determine:

a. Presence or absence of comorbidities including vision impairment, hearing difficulties, epilepsy;
b. Feeding issues including presence or absence of gastrostomy tube and failure to thrive;
c. Respiratory difficulties including episodes of pneumonia and aspiration.

A comprehensive musculoskeletal examination will be performed by a physiotherapist to record data relating to joint range of movement, leg length difference, bony anomalies, motor type and lower limb muscle spasticity and contracture [78-83].

Anthropometry

Anthropometric measures will be collected as described in detail in our published growth, nutrition and physical activity protocol [26], including the following:

a. Body mass to the nearest 100 grams using chair scales (Seca Ltd).
b. Height to the last completed millimetre with a stadiometer, or, length using a supine measuring board. Where a direct measure of height or length cannot be obtained, height will be estimated from knee height or upper arm length using published validated techniques and formulas [84].
c. Body mass index will be calculated as mass (kg) divided by height (m) squared.
d. Growth and growth velocity (*Z*-scores of measured or predicted height).

Weight and body mass index Z-scores will be calculated for age and sex according to Centres for Disease Control and Prevention (U.S. CDC) 2000 growth data [85] .

Gross motor function

Gross motor function will be evaluated using the GMFM-66 & GMFM-88 [86] by experienced research physiotherapists. The GMFM-88 assesses a child's motor abilities in lying to rolling, sitting, crawling to kneeling, standing, walking, running and jumping. The GMFM-66 is comprised of a subset of the 88 items identified (through Rasch analysis) as contributing to the measure of gross motor function in children with cerebral palsy. The GMFM-66 will be used to provide an overall measure of gross motor function and the GMFM-88 provides domain scores to explore specific motor skills [86].

Upper limb performance

Children with unilateral CP whom are manual ability MACS I-III will be assessed on the School kids Assisting Hand Assessment (AHA), a Rasch measure of effectiveness of impaired hand in bimanual

For peer review only - http://bmjopen.bmj.com/site/about/guidelines.xhtml

activities. Test-retest reliability is high (ICC 0.98) and there is predictive validity of future assisting hand use[87]. The Both Hands Assessment (BoHA) will be utilised for children with bilateral CP whom are manual ability MACS I-IV. The BoHA test content was developed by researchers in Norway and Sweden through modification of the Assisting Hand Assessment (AHA) test items and by generation of new items [88]. Associations between BoHA measures and MACS levels show strong correlation (Spearman's rho: 0.74). The person separation ratios (4.36 and 5.19) and the person reliability (0.95 and 0.96) for the subscales indicate that the children's hand function can be separated into 6 and 7 ability levels [88]. The BoHA is the first observation based assessment of effective use of the hands in bimanual activities for children with bilateral CP.

Hand dominance will be assessed using the Edinburgh Handedness Inventory laterality quotient [89]. The Edinburgh Handedness Inventory questionnaire consists of 10 items regarding hand preference (right or left) in performing a number of everyday tasks requiring one (writing, drawing, throwing and using scissors) or two hands (e.g. using a broom or opening a box). The laterality quotient is calculated using the following formula: laterality quotient = (right hand – left hand/ (right hand + left hand)*100). The Edinburgh Handedness Inventory has been included to objectively determine upper limb dominance. This classification system consists of a table that requires the participant to indicate which hand they use to perform a selection of everyday tasks.

Stereognosis relates to a participant's ability to perceive and recognise objects by using only tactile information [90 91] and will be assessed on the impaired and unimpaired limbs, using the approach described by Sakzewski et al [91]. Participants will be required to identify objects placed in their hand, without any visual cues. A total of nine objects are placed in the hand one at a time. Three familiar objects (teaspoon, key, peg) and six similar matched objects (safety pin and paperclip; pen and pencil; coin and button) will be used. With vision occluded, participants will be presented with each item. If a participant is unable to grasp, manipulate or release an object the occupational therapist will assist the participant and move the object for them within their hand. A corresponding set of items will be used to allow participants to identify the object in order to minimise any errors due to incorrect naming of the object. Scores range on a scale from 0-9, where participants scoring below 9, will be considered to have impaired stereognosis [90 91].

Radiological measures of hip displacement and spine

Hip surveillance, including anterior-posterior (AP) pelvis x-ray, is recommended for all Australian children with CP to facilitate early detection and treatment of severe or progressive hip displacement [19 92 93]. The migration percentage (MP) is widely accepted as the gold standard measure in hip surveillance [78 94], measuring femoral head displacement [95]. Other measures include the acetabular index (AI), assessing acetabular dysplasia [96], the Hilgreneiner's Epiphysseal Angle (HEA) [96] and the femoral neck-shaft angle (NSA) [95 97]. The HEA [96] is a radiographic measure describing the proximal femoral epiphysis and has been previously applied to assessment of coxa valga [98 99], but may offer prognostic information for hips at risk in

For peer review only - http://bmjopen.bmj.com/site/about/guidelines.xhtml

cerebral palsy. The HEA represents the acute angle between a line drawn parallel to and through the proximal femoral epiphysis and Hilgenreiner's line [81 82]. Physiotherapists will perform a clinical examination of spinal alignment and mobility to screen for evidence of a potential scoliosis or kyphosis. Where indicated an AP spine radiograph (for scoliosis) or lateral (for kyphosis) will be performed. Spines where scoliosis is evident will be measured according to the Cobb angle [100].

Body composition and bone health:

Body composition measures and bone parameters will be acquired using a Lunar Prodigy DXA (GE Medical Systems, LUNAR, Madison, WI, USA). Body composition measures include: fat mass (FM, g) and fat free mass (FFM, g). Bone parameters include: areal bone mineral density (aBMD, g/cm^2) and bone mineral content (BMC, g) for all total body, bilateral proximal and lateral distal femur sites. The lateral distal femur, is a common site of fracture[101 102], with the technique previously described [102], and measurements are reproducible in children with CP [103 104]. The analysis involves creating three regions of interest, each containing different proportions of trabecular and cortical bone with results for each ROI, therefore, treated independently [101 102]. Additionally, the proximal and distal femoral sites will be used to calculate bone mineral apparent density (BMAD, g/cm^3), derived from the projected bone area (cm^2) to provide an approximation of volumetric BMD [105]. All scans in this research are a 'one off" occurrence, with the total radiation dose for these five DXA scans being <15 μSv[106]. This is equivalent to approximately 1-2 days natural background radiation exposure, and only equivalent to 3% of the dose constraint limit for children as research volunteers, up to the age of 18 years[106]. The total estimated time for all DXA scans is 30 minutes, performed at UQ Children's Nutrition Research Centre.

Fracture rate

Fractures will be diagnosed radiologically. Parents will report by telephone within 24 hours of fracture occurrence and will bring X-ray films and details of management to their study visit. Vertebral fracture will be diagnosed on lateral X-rays of the thoracic and lumbar spine when indicated. Children whom are GMFCS III-V will undergo thoracic and lumbar spine (AP/lateral) at 8-11 years if there are clinical signs of fracture and/or scoliosis/kyphosis. Radiographs will be minimised to reduce the radiation exposure.

Sexual Maturation

Legal guardians of participants will be provided with standardised Tanner stage puberty diagrams, and parents will be asked to evaluate the child's current pubertal stage [107]. Parental pubertal assessment will be reviewed by a physician for precocious puberty. In cases of precocious puberty, a left hand/ wrist X-ray will be conducted to determine the bone age and relative skeletal maturity of children. The bone age will be used to determine if the CP condition is interfering with the proper growth and bone development of the child.

For peer review only - http://bmjopen.bmj.com/site/about/guidelines.xhtml

Pain

Children will complete the Pediatric Pain Questionnaire (PPQ) with adult help if required[108]. The PPQ asks children to report their pain now (severity, type, and location), as well as the severity of the worst pain they had in the previous week. The PPQ's visual analogue scale (VAS) for pain rating provides a valid and stable measure of pain intensity in children and adolescents with chronic musculoskletal pain [109].

Three-dimensional gait analysis and in vivo muscle mechanics

A full 3D gait analysis, including synchronised measurement of muscle activation using electromyography (EMG) and calf muscle mechanics using 2D ultrasound, will be performed for all children functioning at GMFCS I-III. Participants will walk unaided and barefoot at a self-selected speed over a level walkway (10m in length) with four force platforms embedded in the laboratory floor in the centre of the walkway. Reflective markers will be attached to the trunk, pelvis, and lower limbs according to the modified 'Plug in Gait' marker set, with additional clusters of three markers on each thigh and shank segment, and a marker on the 5th metatarsal head [110]. Marker trajectories will be recorded at 100 Hz using an 8-camera, 3D motion capture system (Vicon Motion Systems, Oxford, UK) and ground reaction force data will acquired at 1 kHz using four 510mm × 465mm force platforms (AMTI, Watertown, MA, USA) arranged in series. Lower limb muscle activations of the rectus femoris (RF), medial hamstrings (MH), medial gastrocnemius (MG), lateral gastrocnemius (LG), soleus (SOL) and tibialis anterior (TA) will be recorded for both legs at 1 KHz using a wireless surface EMG system (Aurion ZeroWire, Milan, Italy). Raw EMG signals will be high-pass filtered (Butterworth, zero-lag, 4th order, 30 Hz) to remove movement artefact, full wave rectified and low passed filtered (Butterworth, zero-lag, 4th order, 6Hz), and interpolated to 101 points per cycle. Non-negative matrix factorisation will be applied to extract muscle synergies [111], which represent neuromuscular control during gait. Whole body 3D gait kinematics, joint moments at ankle, knee and hip joints and musculotendinous lengths for MG, LG, SOL, RF and MH will be computed across at least five trials using OpenSim [112] and normalised to length in standing. The Gait Profile Score [113] will be calculated as an index of overall gait pathology. A digital output signal from the ultrasound system was used to synchronize acquisition of all 3D marker, force plate and EMG data.

Two-dimensional B-mode ultrasound will be used to examine MG and SOL muscle function during walking by attaching a flat ultrasound transducer (LV7.5/65/64D, Telemed Echo Blaster 64 EXT-1T, Vilnius, Lithuania) to the surface of the skin above the MG muscle and recording muscle fascicle length and pennation angle changes, as described previously [114]. Muscle fascicle behaviour during walking will be analysed using a semi-automatic process which has been shown to be highly repeatable (Coefficient of multiple correlation 0.88) [115]. The average of five complete strides will be used in the analysis for each participant to ensure the overall reliability of muscle fascicle length data [116]. Freehand three dimensional (3D) ultrasound will be used to measure muscle size and structure of the lower leg muscles: MG, LG, SOL and TA [117]. This method of 3D ultrasound is valid (within 1.3%) and reliable (ICC>0.99) for measuring

For peer review only - http://bmjopen.bmj.com/site/about/guidelines.xhtml

gastrocnemius muscle volume and length in vivo [117] . Calf muscle physiological cross sectional area will be measured as the ratio of muscle volume muscle fascicle length, corrected for fascicle pennation angle.

Activity Limitations

The following measures of activity limitations for functional capacity will be performed for ambulant children (GMFCS I-III) at 8-11 years (≈ n=172).

Six Minute Walk Test (6MWT)

This simple, sub-maximal test measures the distance walked over six minutes, providing information about endurance during functional activities [118]. The 6MWT has excellent test-retest reliability (ICC=0.98) in CP [119]. Percentile curves have been created on 1,445 children with TD aged 7-16 years [120]. The test will be performed according to guidelines of the American Thoracic Society on a 10m course [121].

Muscle power sprint test (MPST)

The MPST provides an estimate of anaerobic power [122]. The MPST requires participants to complete six 15m runs as fast as possible with 10s rest between each lap. Power output is calculated as the product of body mass and distance, divided by time [122]. The MPST has been validated against the Wingate Anaerobic cycling test [123], and has excellent test-retest reliability (ICC 0.98) in children with CP [122].

10m fast walk test (10m FWT)

The 10mFWT is a test of maximal walking speed over a distance considered the minimum for functional ambulation. The 10mFWT has moderate test-retest reliability for in children with CP (ICC 0.81) [124].

Lower limb functional strength

Thirty second repetition maximum (rep_{max}) of functional strength exercises (including sit-to-stand, lateral step-ups and half-kneel to stand) will be tested according to published recommendations [125]. Functional strength tests demonstrate acceptable inter-tester reliability (ICC= >0.91; coefficient of variation (CV) 12.1-22.7%) in children with CP [125]. For each lower limb functional strength exercise participants will be given verbal and visual instructions as well as two practice repetitions prior to testing. The exercises were assessed in the following order: sit-to-stand, lateral step-up dominant leg, lateral step-up non-dominant leg, half-kneel to stand dominant and half-kneel to stand non-dominant. Participants will be given verbal encouragement throughout. Participants will be given 180 seconds rests between exercises. If a participant cannot complete an exercise whilst performing the practice attempts, they will be assigned a score of 0 and will not proceed to testing.

Habitual Physical Activity (HPA)

Triaxial accelerometers (ActiGraph GT3X+, Pensacola, FL, USA) will be used to evaluate the frequency, intensity, and duration of physical activity [126]. ActiGraph accelerometers have evidence of validity and inter-

For peer review only - http://bmjopen.bmj.com/site/about/guidelines.xhtml

instrument reliability in children with TD compared to heart rate monitoring, direct observation, indirect calorimetry, whole-room calorimetry and doubly labelled water [126]. The ActiGraph has been validated for measurement of physical activity intensity in adolescents with CP using oxygen uptake as the criterion measure[127 128]. ActiGraphs will be fitted during assessment and worn during waking hours for 7 days[126]. Stored data will be uploaded to an excel macro to determine daily wear time, average counts per min, daily time spent in sedentary, light, moderate, and vigorous activity. Counts will be classified using established cut-points for children with CP[128].

Blood samples for Growth Hormone and Vitamin D:

Blood will be collected and tested for hormones and other markers of required for optimal growth, bone and metabolic health. Specifically, these tests are liver function, kidney function, full blood count (FBC), Insulin like Growth Factor-1 (IGF1), thyroid hormone, parathyroid hormone, vitamin D3, calcium, phosphate, and iron studies. As described above, these parameters of growth, bone health and body composition are often altered in children with CP and are related to gross motor function classification, body composition, growth velocity and nutritional status. Blood tests will be optional and consent will be obtained from the parent and assent from the child where possible. Blood samples will be collected by qualified phlebotomists, who are familiar with collecting blood from paediatric subjects using their standard procedures. Where preferred, samples will be collected under general anaesthesia, if a patient is undergoing an unrelated and non-emergency surgical procedure (e.g. orthopaedic surgery, Botulinum Toxin A injections, MRI under anaesthesia). Parents of participants will be advised if these results fall outside the relevant reference ranges in relation to age, gender and pubertal status. Parents will provide informed consent for information to be provided to their treating clinician who will take responsibility for ongoing care and follow up.

Dietary Intake

Dietary energy intake will be recorded using a 3-day weighed food record as validated[29] using our published methods[26]. Food records will be analysed using FoodWorks™. Mean energy intake will be expressed as megajoules per day and as a percentage of age and gender specific recommendations [129].

Vitamin D intake

A vitamin D in a food frequency questionnaire will be completed by parents to determine the habitual intake of Vitamin D containing foods of the participants. The questionnaire consists of a table that requires parent's to tick a frequency box and record the brand of a simple list of foods[130] (Appendix 2).

Sun Exposure

A Sun Exposure Diary will measure daily sun exposure in the participants to measure ultraviolet radiation (UV) exposure for vitamin D adequacy. Each day, participants will record the amount of time spent in the sun during each 1-hour interval (0, <15, 15–<30, 30–<45, or 45–60 minutes) between 5:00 AM and 7:00

For peer review only - http://bmjopen.bmj.com/site/about/guidelines.xhtml

PM. Clothing cover (based on a clothing cover guide provided with the dairy) and use of sunscreen (frequency and application site) using established methodology will also be recorded [131] (Appendix 3). It is proposed that the sun exposure diary will be done at the same time as the Physical activity monitor record to lessen the burden on the participants. Sun exposure diaries will be done within two weeks of serum Vitamin D levels being collected, to allow for meaningful interruption of sun exposure and Vitamin D levels.

Oropharyngeal dysphagia

Oropharyngeal dysphagia (feeding and swallowing difficulties) will be evaluated during a digital video recorded snack of 20 minutes. Children will be presented with three standardised boluses of five textures; puree, semi-solid, chewable, tough chewable and fluid. The following measures will be used to rate the mealtime:

1. The Dysphagia Disorders Survey (DDS) – Part 2 consists of a series of binary judgments on eight ingestion functions across the oral preparatory, oral, pharyngeal and gastro-oesophageal phases (maximum raw score of 22). The DDS has good reliability [132 133] and convergent validity [132-136].

2. The Schedule for Oral Motor Assessment (SOMA) consists of seven oral motor challenge categories corresponding to four food textures and three fluid utensils. The SOMA has been validated on 127 young infants; 58 comparison children with typical oral skills, 56 with non-organic failure to thrive (aged 8-24 months), and 13 children with CP and overt feeding difficulties (aged up to 42 months) [137]. It has strong inter-rater reliability (κ=1.0 in 68% of fluid category items and 58% of food category items) and test-retest reliability between boluses (κ=1.0 in 84% of items) [137].

3. Observations of 16 clinical signs suggestive of pharyngeal phase impairment (e.g. cough, gurgly phonation, wet respiration) will be rated pre- and post-mealtime by a trained researcher, and rated according to each food/ fluid texture from video by a speech pathologist [138-140].

4. The Thomas-Stonell and Greenberg scale will be used pre and post mealtime to rate saliva loss [141]. This consists of two observational ordinal scales (1-5), based on severity and frequency of loss.

5. The Cerebral Palsy Child Feeding Questionnaire (CPFQ), used in the CP Child Study [142] will gather information on the child's typical mealtime performance based on parent report, which will supplement the data obtained from the participant's clinical feeding assessment.

6. The Feeding/Swallowing Impact Scale (FS-IS) will address questions of carer Quality of Life and how to incorporate it into economic evaluation. The Feeding/Swallowing Impact Scale (FS-IS) is a validated tool to measure the impact of caring for a child with dysphagia and concerns on caregiver Quality of Life[143]. It is an 18 item, parent questionnaire divided into three subsections: i) daily activities; ii) worry; and iii) feeding difficulties. The tool was validated on the caregivers of 164 children (median age 14 months, mean: 32 ± 44 months) with varying co-morbidities including prematurity (<37 weeks) in 66 (40%) children, 144 (88%)

For peer review only - http://bmjopen.bmj.com/site/about/guidelines.xhtml

were medically complex with conditions in more than one diagnostic based category; and 77 (47%) of children had feeding tubes[143].

Communication

All children will have language assessed using the core language subtests of the Clinical Evaluation of Language Fundamentals screener (CELF-4) [144], in addition to the Peabody Picture Vocabulary Test (PPVT). The CELF-4 is a criterion referenced assessment of language skills in children aged 5-21 years, with Australian norms available. Children who are non-verbal will only complete the receptive subtests of the CELF-4 and the PPVT. Children unable to participate in standardised assessment (e.g. due to significant cognitive, visual or motor limitations) will have language classified using the Triple C, a parent reported observational assessment. Communication performance will be indicated by parents on a communication questionnaire developed for this study (Appendix 5), which includes information on Augmentative and Alternative Communication system type, use, frequency and access [144].

Speech production will be assessed using the Verbal Motor Production assessment for Children (VMPAC). The VMPAC [145] is a diagnostic tool for the systematic assessment of neuromotor integrity of the motor speech system validated in 1,434 children aged 3-12 years[146]. The following subtests of the VMPAC will be administered:

1. Oromotor Production in Word Sequences and Sentences (6 items): Items consists of 3- and 4-word sequences and 5-word sentences. These items are designed to evaluate the child's ability to sequence oromotor movements across different plane within a linguistic context.

2. Oromotor Production in Connected Speech and Language (5 items): This subtest will assess the child's motor control (e.g. motor precision) as it varies in the context of higher level language formulation.

3. Oromotor Production in Automatic Verbal Sequences (1 item): This subtest allows evaluation of speech characteristics including pitch, resonance, vocal quality, loudness, prosody, intonation, and rate during production of an automatic speech task (e.g. counting to ten, saying the alphabet).

4. Oromotor Production in Word Sequences and Sentences (6 items): Items consists of 3- and 4-word sequences and 5-word sentences. These items are designed to evaluate the child's ability to sequence oromotor movements across different plane within a linguistic context. Oromotor Production in Connected Speech and Language (5 items): This subtest will assess the child's motor control (e.g. motor precision) as it varies in the context of higher level language formulation.

5. Oromotor Production in Automatic Verbal Sequences (1 item): This subtest allows evaluation of speech characteristics including pitch, resonance, vocal quality, loudness, prosody, intonation, and rate during production of an automatic speech task (e.g. counting to ten, saying the alphabet).

For peer review only - http://bmjopen.bmj.com/site/about/guidelines.xhtml

Pediatric Evaluation of Disability Inventory Computer Adapted Test (PEDI-CAT)

Performance of self-care will be evaluated using the parent-reported Pediatric Evaluation of Disability Inventory Computer Adapted Test (PEDI-CAT) for the domains of self-care, mobility and social functioning using scaled scores (Rasch), which have good validity and reliability[147]. The PEDI-CAT was developed on the basis of the original PEDI [148]. Scaled scores (possible range 20-80) for each domain provide an indication of the child's performance along a continuum of item difficulty and are most suitable for research [147]. Scaled scores give more precise results in the extreme ranges than normative standard scores [147]. Scaled scores are recommended to track functional progress in children who are substantially delayed [147].

Participation and Environmental Measures

Participation and Environment Measure for Children and Youth (PEM-CY)

The PEM-CY is a parent-reported instrument that examines participation and environment across three settings: i) home; ii) school; and iii) community [149]. There are 10 items in the home section, five in the school section and 10 in the community setting. For each item, the parent is asked to identify how frequently (over the past four months) the child has participated (eight options: daily to never); how involved the child typically is while participating (five point scale: very involved to minimally involved); and whether the parent would like to see the child's participation in this type of activity change (no or yes, with five options for the type of change desired). For each setting, the parent is then asked to report on whether certain features of the environment make it easier or harder for the child to participate. The PEM-CY has reported moderate to good internal consistency (0.59 and above) and test-retest reliability (0.58 and above) in a population of children (aged 5 to 17 years) with and without disabilities residing in the United States of America and Canada (*n*=576) [149]. The PEM-CY will be collected using either a paper or online questionnaire format to gain an understanding of the participation of children and adolescents and the impact of environmental barriers and facilitators.

Strengths and Difficulties Questionnaire (SDQ)

The Strengths and Difficulties Questionnaire (SDQ) [150 151] is a 33 item parent-rated questionnaire that is used to assess parents' perceptions of pro-social and difficult behaviours in their child or child adjustment. Parents respond to 25 questions about their child's behaviour in the last six-months using a three-point Likert scale (i.e., "0" = not true to "2" = certainly true). These 25 questions combine to create five sub-scales of: frequency of emotional symptoms; conduct problems; inattention/hyperactivity; peer problems; and prosocial behaviour (e.g. "considerate of other people's feelings"). A total score for each scale (0-10) and overall total difficulties score (0-40) will be calculated, with higher scores indicating more distress on all scales except prosocial behaviour. Scores of 17 or above for the total difficulties scale will be used a clinical cut-off point. Scores from the five sub-scales and the overall difficulties scale will be used as a measure of the child's psychological functioning. The overall total difficulties score has been demonstrated to have

For peer review only - http://bmjopen.bmj.com/site/about/guidelines.xhtml

moderate to high internal consistency (Cronbachs α = 0.73-0.82) and test-retest reliability (r = 0.77 – 0.85) [152].

School Attainment

The Australian Early Development Index (AEDI) is a population measure of development at school entry, assessing school readiness [153]. The National Assessment Program - Literacy and Numeracy (NAPLAN), measures literacy/numeracy achievement and individual reports will be obtained from families as a measure of early school achievement [154]. Where applicable, consent will be obtained from parents to the release of NAPLAN result by the Queensland Curriculum and Assessment Authority (QCAA).

Cognition

Raven's Coloured Progressive Matrices (RCPM)

The RCPM is an assessment of nonverbal intelligence for children aged 5-11 years with intellectual delay or physical disability. It consists of 36 items (15-30 minutes) of increasing difficulty in which the child has to complete a pattern. RCPM has validity with CP and Australian norms [155 156]. In 618 Australian children (6-11years) the RCPM had good internal consistency (0.76-0.88) and split-half reliability (0.81-0.90) [156].

Behaviour Rating Inventory of Executive Function (BRIEF)

The BRIEF is a parent completed 86 item measure of executive function in their child's everyday life, yielding two scores: i) the behavioural regulation index; and ii) the metacognition index to form a global executive composite score. Both scores can be converted into T scores with ≥65 indicative of dysfunction [157]. It has good convergent and divergent validity with Child Behaviour Checklist and the Behaviour Assessment System for Children [158], high internal consistency, (Cronbach's α 0.80-0.98) for parent form [158 159].

Attention and Executive Functioning

Connors 3 parent short form (Conners 3™)

The Conners 3™ [160] is a thorough assessment of ADHD and its most common co-morbid problems and disorders in children and adolescents ages 6-18 years old. The Conners 3™ will be completed by the participant's parents or guardian and consists of 110 statements and takes approximately 20 minutes to complete. Parents or guardians must rate each statement using a four-point scale ranging from '0 – Not true at all (never, seldom)' to '3 – Very much true (very often, very frequently)'. The Conners 3™ measures the seven key areas of inattention, learning problems, aggression, family relations, hyperactivity/impulsivity, executive functioning, and peer relations. Raw scores are converted into T scores based on a large representative normative sample based on United States of America consensus data. In addition, the Conners 3™ calculates T scores for symptom scales including ADHD Hyperactive/Impulsive, ADHD Combined, Oppositional Defiant Disorder, ADHD Inattentive, and Conduct Disorder. Both internal consistency

For peer review only - http://bmjopen.bmj.com/site/about/guidelines.xhtml

coefficients (α = 0.83-0.94) and test-retest reliability (r = 0.52-0.94) are good for the Conners 3™ Parent version total sample age range [160].

The Autism Spectrum Quotient-Child (AQ10-Child)

The AQ10-child is a 10 item parent-report autism screening measure for children aged 4-11 years [161]. It was developed from the Quantitative Checklist for Autism in Toddlers (Q-CHAT). Parents respond to the 10 items on a 4 point Likert Scale from Definitely Agree to Definitely Disagree. Items are summed and the scale has a cut off of 6. It has high internal consistency (Cronbach's α =0.90), sensitivity (0.95) and specificity (0.89) [161].

Sleep Disturbances Scale for Children (SDSC)

Sleep disorders are up to four times more common in children with cerebral palsy compared to the general population and are linked to both physical (total body involvement, severe visual impairment) and environmental factors (single-parent household, bed-sharing). The most commonly reported disorders include difficulty initiating and maintaining sleep, sleep-wake transitions and sleep breathing disorders [162]. The SDSC is a 27 item parent-report questionnaire that assesses sleep disturbance in children within the past six months [163]. Each item is responded to on a 5 item Likert scale with higher values representing greater clinical severity. Items are summed to produce a total score and six sub-scores representing different facets of sleep disturbance: disorders of initiating and maintaining sleep, sleep breathing disorders, disorders of arousal, sleep-wake transition disorders, disorders of excessive somnolence and sleep hyperhidrosis. It has good internal consistency (Cronbach's α = 0.71-0.79), test-retest reliability (r=0.71) and discriminative validity in distinguishing between clinical and community samples [163].

Quality of Life Measures

Condition-specific Quality of Life measures

The Cerebral Palsy Quality of Life-child (CPQOL-child) assesses wellbeing across multiple domains using parent-report (4-12 years) and child self-report from 9 years [164-166]. Psychometrics are excellent with Cronbach's α 0.74-0.92 parent-proxy report and 0.80-0.90 child self-report. Test re-test is adequate (ICC 0.76-0.89) and moderately correlated with health (r=0.30-0.51)[164-166].

Generic Quality of life

The Child Health Utility-9D (CHU-9D) is a generic instrument for children aged 7-11 years for which there is an algorithm to give a single preference-based utility index for health states (giving a single generic preference-based indicator of each individual's health state), making the data amenable for economic evaluations for interventions [167-170]. The EuroQOL five dimensional descriptive system (EQ-5D-5L) is a generic instrument designed to describe and value health based on 5 dimensions: mobility, self-care, usual

For peer review only - http://bmjopen.bmj.com/site/about/guidelines.xhtml

activities, pain/discomfort and anxiety/depression [171]. The EQ-5D-5L will be completed by the parent/care-giver, about themselves and scored using the Australian algorithm [172 173]. This is relevant (alongside the CES) to address questions such as carers QOL and how to incorporate it into economic evaluation.

Demographic Questionnaire

Parents will be required to complete questionnaires related to various aspects of their child's development/progress including participation, feeding, health related quality of life, sleep, fracture history, pain, executive functioning in everyday life, clinical co morbidities including epilepsy, psychological functioning, treatments and interventions the child has received since the completion of the CP Child projects (Appendix 1).

Parent Questionnaires

Carer Experience Scale (CES)

The CES will be completed by primary care givers at their study visit. This validated measure of care-related QoL has six domains (activities, support, assistance, fulfilment, control and relationship with the care recipient) and takes approximately 3 minutes to complete [174]. The CES is scored from an algorithm derived from preferences of the general population and can be used to value carer outcomes in economic evaluation using index values [175].

The McMaster Family Assessment Device General Functioning Scale (FAD)

Caring for a child with a disability can have an impact on the health and functioning of the care-giver and family unit [176]. The FAD is a 12-item measure of general family functioning [177]. Each item is rated on a four point Likert scale from strongly agree to strongly disagree and the items are summed to create the total score. It has good internal consistency (Cronbach's $\alpha = 0.92$), construct validity and reliability [177-179].

Monitoring of resource use and direct costs of treatments

To determine the relationship between motor prognosis and healthcare costs, resource use and direct costs of treatment will be monitored using the Health Resource use (HRU) questionnaire [14] (Appendix 4). Associations between costs (dependent variable) and all other outcome variables (independent variables) including those related to growth, body composition and HPA will be assessed, with adjustment for confounders such as brain lesion severity. Health-related resource use data collected includes therapy frequency and duration (traditional/alternate), hospital admissions, GP and medical specialist visits, medications (e.g. Botulinum Toxin-A), and equipment (e.g. orthoses). Data will be collected via questionnaire [14], supplemented by consented access to individual hospital, Medical Benefits Scheme (MBS) and Pharmaceutical Benefits Scheme (PBS) records. Standard cost sources (MBS, PBS) will be used to apply unit costs to resources. Statistical approaches, which consider the likely skewed distribution of cost data

For peer review only - http://bmjopen.bmj.com/site/about/guidelines.xhtml

(such as generalised linear models, with extensions to allow for correlations in the data across observations from multiple time points from each individual as described below), and diagnostic related group costs for admissions to hospital)will be employed. As this cohort is embedded in a state-wide clinical service, we have consistency of interventions based on best practice guidelines for Botulinum toxin A (BoNT-A), bone health and hip surveillance generalisable across Australia. All children are offered best practice treatment across the state-wide service.

Data Analysis Plan

A comprehensive database has been established for all data collection, including clinical measures, MRI scoring and questionnaires so that it is entered prospectively at the time of each assessment. Summary reports are automatically generated from the database to report back to families and treating clinicians after each visit. Bio statistics methods proposed in this study include analysis of binary outcomes in longitudinal studies using weighted estimating equations (e.g. presence of comorbidities); multilevel mixed-effects models of longitudinal binary outcomes (e.g. GMFCS levels), and generalised estimating equations for ordinal data.

Sample size

We assume 95% of the total sample of 245 children will consent to Predict-CP program (we have 98% retention in NHMRC 465128, 569605). For H^1, assuming between-GMFCS group and within-child variability in GMFM-66 are similar to Rosenbaum[180], we will be able to detect significant between-group differences in GMFM-66 scores at 8-11 years according to the primary predictor variable of initial GMFCS group (2-5 years) with >80% power and alpha=0.05 for each pairwise comparison. For comparisons between MRI classification at 2 years and GMFM-66 at 8-11 years, then based on GMFCS and MRI data from NHMRC 465128 (where GMFCS I=30%, II-V=16% each and WMI=43%), assuming that GMFM-66 at 8-11 years has a SD of 7 units within each GMFCS group[180], we will be able to detect a difference between children with white-matter damage and other MRI pattern types of ≥ 4.6 GMFM-66 points in GMFCS I and ≥ 6.7 points within each GMFCS level II–V, with 80% power, alpha=0.05. For secondary outcomes we have differing power depending on sample characteristics e.g. two groups, evenly distributed, we will detect differences of ≥0.36 SD with α=0.05, 80% power.

Statistical considerations

Summary statistics will be described using either mean (standard deviation) or median (25th-75th percentile) for continuous variables, according to distribution, or as frequency(percentage) for categorical variables. Cross-sectional associations will be assessed using linear regression for interval outcome data, with effect estimates presented as mean difference and 95% confidence interval (95%CI), using logistic regression for binary outcome data, with effect estimates presented as odds ratios and 95%CI, and using Poisson regression for count outcome data, with effect estimates presented as incidence rate ratios and 95%CI. Longitudinal associations will be investigated using analyses that account for the multiple observations per participant. The particular analysis will be determined by data structure, for example hierarchical mixed-effects models or generalised estimating equations. If hierarchical mixed-effects models are used then 'participant' will be

For peer review only - http://bmjopen.bmj.com/site/about/guidelines.xhtml

included in the model as a random effect, to account for the possible non-independence of observations from the same participant. When building multivariable models, first univariable models including all potential variables of interest will be constructed. Variables will be selected for potential inclusion in multivariable models based on univariable significance at the p<0.2 level. Multivariable models will be built in a step-wise manner with redundant variables eliminated using Akaike's and Schwarz's Bayesian criteria. In principal, multivariable models will first be constructed for the whole sample, then after stratification by GMFCS level. Interactions will be investigated as appropriate. For H^1 we will first undertake univariable analyses of the association between quantitative MRI, motor capacity, performance, and potential confounding variables (e.g. gender) at 2-5 years with motor capacity at 8 years using a mixed-effects model with data grouped by individual participants to account for (up to 3) repeated measures between 2-5 years.

Missing data will be treated on a case-by-case basis depending on the observed pattern of missingness. For example if data is 'missing at random' we will use multiple imputation methods, and if data is 'not missing at random' we will use pattern-mixture models. There will be no global rule to account for multiple comparisons, instead adjustment for multiple comparisons will be made for each separate suite of analyses as appropriate, bearing in mind the type I & II error rates for each suite. Cost data will be standardised to current values. Cost data are typically skewed and therefore will be tested for normality and transformed using a log, gamma or another appropriate link function for the multivariable analysis

Complex multivariable analyses accounting for attrition, if required, will be conducted by a biostatistician. The precise analyses will be determined by data structure. In principal, hierarchical mixed-effects models will be performed for the whole population then by GMFCS level. First, univariable then multivariable analyses will be undertaken. Variables included as fixed effects in the final multivariate model will be selected based on univariate results if p<0.2. Redundant variables will be eliminated from multivariable models using Akaike's and Schwarz's Bayesian criteria. All models will include a random intercept and slope (time effect) for each participant, accounting for the non-independence in repeated measures from the same participant and allowing for heterogeneity between participants. For H^1 we will first undertake univariable analyses of the association between quantitative MRI, motor capacity, performance, and potential confounding variables (e.g. gender) at 2-5 years with motor capacity at 8 years using a mixed-effects model with data grouped by individual participants to account for (up to 3) repeated measures between 2-5 years. Then all variables significant at P<0.2 will be included in the prediction model, before being investigated for elimination. Interactions will be investigated as appropriate. Missing data will be treated on a case-by-case basis using MAR (multiple imputation) or NMAR (using pattern-mixture models). Adjustment for multiple comparisons will be made for each separate analyses mindful of type I & II error rates, as is standard practice.

DISCUSSION

This study protocol describes the rationale, aims, hypotheses and methods for a large, prospective, longitudinal, population-based study of motor development and brain structure in a representative sample of

For peer review only - http://bmjopen.bmj.com/site/about/guidelines.xhtml

preschool aged children with Cerebral Palsy, using direct clinical assessment. The results of this study will be published in peer reviewed journals and presented at relevant international conferences. Outcomes of this prospective cohort study will inform prediction of patient outcomes and likely needs, which will impact clinical practice, the management of children with CP, their carers and the health care system more widely.

AUTHORS' CONTRIBUTIONS

RB is the chief investigator and together with chief investigators PSWD, JZ, ST, LB, RW, SR, KW, JAW, KB and Associate Investigators KB, CC, JW, PE, MK, LC, LS, KW, AC, AG conceptualized, designed and established this research study and successfully obtained study funding for a National Health and Medical Research Council partnership grant. RP, KB, CD, SO, SR, DB, AB, SF and AG were responsible for the selection of particular assessments. JAW conceptualised the inclusion of QoL instruments for children and carers able to support economic evaluation. RB, SR, SF and AG were responsible for conceptualising the brain MRI protocol and analysis. RB is responsible for ethics applications and reporting. RB, KB, LB, CC, PE, LS, LC, KW, DB, KB, CD, SO, SER, CF, AB, KM, OL will be responsible for recruitment and data collection. RB drafted the manuscript with input from all the co-authors. All authors have agreed the final version of the manuscript and were involved in the decision to submit the manuscript. There is no financial support for the authors regarding this manuscript. The external funding agencies (NHMRC) have provided funds for the conduct of the study but will not be involved manuscript preparation, decisions to publish or the interpretation of results arising from the study.

COMPETING INTERESTS

The authors declare they have no competing interests.

FINANCIAL INTERESTS

The authors declare that they have no financial or non-financial competing interests.

FUNDING STATEMENT

This work was supported by the National Health and Medical Research Council of Australia (NHMRC Partnership Project 1077257) with in kind support from Children's Health Queensland, CSIRO and Griffith University. The National Health and Medical Research Council has provided the following people support: Research Fellowship (R Boyd); Early Career Fellowship (L Barber, L Sakzewksi, K Whittingham).

List of abbreviations

ADL: Activities of daily living

AHA: Assisting hand assessment

BGBS: Basal ganglia and brainstem score

BM: Brain Malformation

BMC: bone mineral content

For peer review only - http://bmjopen.bmj.com/site/about/guidelines.xhtml

aBMD: areal bone mineral density

BMI: Body mass index

BRIEF: Brief rating inventory of executive functioning

CDGM: Cortical and deep grey matter

CFCS: Communication Function Classification System

CHU-9D: Child health utility 9D

CP: Cerebral palsy

CPFQ: Cerebral Palsy Feeding Questionnaire

CSD: Constrained spherical deconvolution

DDS: Dysphagia Disorders Survey

DXA: Dual-energy X-ray absorpitometry

EDACS: Eating and Drinking Ability Classification System

EPI: Echo-planar imaging

ET: Echo time

FC: Functional connectivity

FCCS: Functional Communication Classification System

FOV: Field of view

FSIQ: Full scale intelligence quotient

GEEs: Generalised estimating equations

GMFCS: Gross motor functional classification system

GMFM: Gross motor function measure

GRE: Gradient-recalled-echo

GS: Global score

HPA: Habitual physical activity

HS: Hemispheric score

ICC: Intraclass correlation coefficients

ICF: International classification of functioning, disability and health

IQ: Intelligence quotient

KM: Krägeloh-Mann

LI: Laterality index

MACS: Manual ability classification system

METS: Metabolic equivalents

MDC: Minimum detectable change

MRI: Magnetic resonance imaging

OPD: Oropharyngeal Dysphagia

PEDI: Pediatric evaluation of disability inventory

PEM-CY: Participation and environment measure for children and youth

PPVT: Peabody Picture Vocabulary Test

For peer review only - http://bmjopen.bmj.com/site/about/guidelines.xhtml

For peer review only

PWM: Periventricular white matter

QCPRRC: Queensland cerebral palsy and rehabilitation research centre

QOL: Quality of life

Rep_{max}: Repetition maximum

ROM: Range of motion

RT: Repetition time

SDQ: Strengths and difficulties questionnaire

SEM: Standard error of measurement

SOMA: Schedule for Oral Motor Assessment

sMRI: Structural magnetic resonance imaging

TD: Typical development

UL: Upper limb

VMPAC: Verbal Motor Performance Assessment for Children

WHO: World health organisation

3T: 3 tesla

6MWT: Six minute walk test

For peer review only

REFERENCES

1. Rosenbaum P, Paneth N, Leviton A, et al. A report: the definition and classification of cerebral palsy April 2006. Dev Med Child Neurol 2007;**109**:8.
2. Graham HK. Absence of reference to progressive musculoskeletal pathology in definition of cerebral palsy. Dev Med Child Neurol 2006;**48**(1):78-79.
3. Hanna SE, Rosenbaum PL, Bartlett DJ, et al. Stability and decline in gross motor function among children and youth with cerebral palsy aged 2 to 21 years. Dev Med Child Neurol 2009;**51**(4):295-302.
4. Australian Cerebral Palsy Register Group. Report of the Australian Cerebral Palsy Register, Birth Years 1993-2006, 2013.
5. Jordan R, David M, Pareezer L, et al. Relationship between health resource cost and performance outcomes in preschool age children with cerebral palsy; economic analysis. Dev Med Child Neurol 2013;**55**(s3):6.
6. Novak I, Hines M, Goldsmith S, et al. Clinical prognostic messages from a systematic review on cerebral palsy. Pediatrics 2012;**130**(5):e1285-312.
7. Bodimeade HL, Whittingham K, Lloyd O, et al. Executive function in children and adolescents with unilateral cerebral palsy. Dev Med Child Neurol 2013;**55**(10):926-33.
8. Guzzetta A, Sinclair K, Clarke D, et al. A novel semi-quantitative scale for classification of brain MRI for children with cerebral palsy. Dev Med Child Neurol 2010;**52**:74.
9. Australian Bureau of Statistics. *Sport and Recreation: A Statistical Overview, Australia*. Canberra: Australian Government, 2006.
10. Access Economics. The Economic Impact of Cerebral Palsy in Australia in 2007, 2008.
11. Krägeloh-Mann I, Horber V. The role of magnetic resonance imaging in elucidating the pathogenesis of cerebral palsy: a systematic review. Dev Med Child Neurol 2007;**49**(2):144-51.
12. Arnfield E, Guzzetta A, Boyd R. Relationship between brain structure on magnetic resonance imaging and motor outcomes in children with cerebral palsy: a systematic review. Res Dev Disabil 2013;**34**(7):2234-50.
13. Krägeloh-Mann I. Imaging of early brain injury and cortical plasticity. Exp Neurol 2004;**190**(Suppl 1):84-90.
14. Boyd RN, Jordan R, Pareezer L, et al. Australian Cerebral Palsy Child Study: protocol of a prospective population based study of motor and brain development of preschool aged children with cerebral palsy. BMC Neurol 2013;**13**(1):57.
15. Pym A, Ware R, Jordan R, et al. Early predictors of hip displacement by school age in young children with cerebral palsy. Dev Med Child Neurol 2012;**54**(s6):61.
16. Whittingham K, Fahey M, Rawicki B, et al. The relationship between motor abilities and early social development in a preschool cohort of children with cerebral palsy. Res Dev Disabil 2010;**31**(6):1346-51.
17. Coleman A, Weir KA, Ware RS, et al. Relationship Between Communication Skills and Gross Motor Function in Preschool-Aged Children With Cerebral Palsy. Arch Phys Med Rehabil 2013;**94**(11):2210-17.
18. Stephenson J, Bauman A, Armstrong T, et al. The costs of illness attributable to physical inactivity in Australia. Canberra, Australia: Commonwealth Department of Health and Aged Care and the Australian Sports Commission, 2000.
19. Hagglund G, Andersson S, Duppe H, et al. Prevention of dislocation of the hip in children with cerebral palsy. The first ten years of a population-based prevention programme. J Bone Joint Surg 2005;**87**(1):95-101.
20. Hagglund G, Wagner P. Spasticity of the gastrosoleus muscle is related to the development of reduced passive dorsiflexion of the ankle in children with cerebral palsy. Acta Orthop 2011;**82**(6):744-48.
21. Uddenfeldt Wort U, Nordmark E, Wagner P, et al. Fractures in children with cerebral palsy: a total population study. Dev Med Child Neurol 2013;**55**(9):821-6.
22. Westbom L, Lundkvist Josenby A, Wagner P, et al. Growth in children with cerebral palsy during five years after selective dorsal rhizotomy: a practice-based study. BMC Neurol 2010;**10**:57.
23. Wynter M, Gibson N, Kentish M, et al. The Consensus Statement on Hip Surveillance for Children with Cerebral Palsy: Australian Standards of Care. J Pediatr Rehabil Med 2011;**4**(3):183-95.

For peer review only - http://bmjopen.bmj.com/site/about/guidelines.xhtml

24. Kentish M, Wynter M, Snape N, et al. Five-year outcome of state-wide hip surveillance of children and adolescents with cerebral palsy. Journal of Pediatric Rehabilation Medicine 2011;**4**(3):205-17.
25. Weil E, Wachterman M, McCarthy EP, et al. Obesity among adults with disabling conditions. J Am Med Assoc 2002;**288**(10):1265-8.
26. Bell KL, Boyd RN, Tweedy SM, et al. A prospective, longitudinal study of growth, nutrition and sedentary behaviour in young children with cerebral palsy. BMC Public Health 2010;**10**:179.
27. Walker JL, Bell KL, Boyd RN, et al. Energy requirements in preschool-age children with cerebral palsy. Am J Clin Nutr 2012;**96**(6):1309-15.
28. Walker JL, Bell KL, Boyd RN, et al. Validation of a modified three-day weighed food record for measuring energy intake in preschool-aged children with cerebral palsy. Clin Nutr 2013;**32**(3):426-31.
29. Walker JL, Bell KL, Stevenson RD, et al. Relationships between Dietary Intake and Body Composition according to Gross Motor Functional Ability in Preschool-Aged Children with Cerebral Palsy. Ann Nutr Metab 2012;**61**(4):349-57.
30. Oftedal S, Bell K, Mitchell L, et al. A Systematic Review of the Clinimetric Properties of Habitual Physical Activity Measures in Young Children with a Motor Disability. Int J Pediatr 2012;**2012**:12.
31. Benfer KA, Weir KA, Bell KL, et al. Oropharyngeal dysphagia and gross motor skills in children with cerebral palsy. Pediatrics 2013;**131**(5):e1553-62.
32. Weir K, Bell K, Ware R, et al. Reported eating ability of young children with cerebral palsy: Is there an association with gross motor function? . Arch Phys Med Rehabil 2012;**94**(3):495-502.
33. Benfer KA, Weir KA, Bell KL, et al. Oropharyngeal dysphagia in preschool children with cerebral palsy: oral phase impairments. Res Dev Disabil 2014;**35**(12):3469-81.
34. Oftedal S, Bell KL, Davies PS, et al. Relationship between daily physical activity performance and functional mobility capacity and performance in toddlers with cerebral palsy. Dev Med Child Neurol 2013;**55**(S3):5.
35. Keawutan P, Bell K, Davies PS, et al. Systematic review of the relationship between habitual physical activity and motor capacity in children with cerebral palsy. Res Dev Disabil 2014;**35**(6):1301-09.
36. Barrett R, Lichtwark G. Gross muscle morphology and structure in spastic cerebral palsy: a systematic review. Dev Med Child Neurol 2010;**52**(9):794-804.
37. Graham HK. Botulinum toxin type A management of spasticity in the context of orthopaedic surgery for children with spastic cerebral palsy. Eur J Neurol 2001;**8** (Suppl 5):30-9.
38. Bache CE, Selber P, Graham HK. (ii) The management of spastic diplegia. Curr Orthop 2003;**17**(2):88-104.
39. Opheim A, Jahnsen R, Olsson E, et al. Balance in relation to walking deterioration in adults with spastic bilateral cerebral palsy. Phys Ther 2012;**92**(2):279-88.
40. Barber L, Barrett R, Lichtwark G. Passive muscle mechanical properties of the medial gastrocnemius in young adults with spastic cerebral palsy. J Biomech 2011;**44**(13):2496-500.
41. Barber L, Hastings-Ison T, Baker R, et al. Medial gastrocnemius muscle volume and fascicle length in children aged 2 to 5 years with cerebral palsy. Dev Med Child Neurol 2011;**53**(6):543-8.
42. Barber L, Barrett R, Lichtwark G. Medial gastrocnemius muscle fascicle active torque-length and Achilles tendon properties in young adults with spastic cerebral palsy. J Biomech 2012;**45**(15):2526-30.
43. Wren TA, Cheatwood AP, Rethlefsen SA, et al. Achilles tendon length and medial gastrocnemius architecture in children with cerebral palsy and equinus gait. J Pediatr Orthop 2010;**30**(5):479-84.
44. Perry J, Hoffer MM, Giovan P, et al. Gait analysis of the triceps surae in cerebral palsy. A preoperative and postoperative clinical and electromyographic study. J Bone Joint Surg 1974;**56**(3):511-20.
45. Eek MN, Beckung E. Walking ability is related to muscle strength in children with cerebral palsy. Gait Posture 2008;**28**(3):366-71.
46. Barber LA, Read F, Lovatt Stern J, et al. Medial gastrocnemius muscle volume in ambulant children with unilateral and bilateral cerebral palsy aged 2 to 9 years. Dev Med Child Neurol 2016.
47. Gage JR, Schwartz MH, Koop SE, et al., editors. *The Identification and Treatment of Gait Problems in Cerebral Palsy*. 2nd ed. London: MacKeith Press, 2009.
48. Winters TF, Gage JR, Hicks R. Gait patterns in spastic hemiplegia in children and young adults. J Bone Joint Surg 1987;**69**(3):437-41.

For peer review only

49. Howard J, Soo B, Graham HK, et al. Cerebral palsy in Victoria: motor types, topography and gross motor function. J Paediatr Child Health 2005;**41**(9-10):479-83.
50. Palisano R, Rosenbaum P, Walter RS, et al. Development and reliability of a system to classify gross motor function in children with cerebral palsy. Dev Med Child Neurol 1997;**39**(4):214-23.
51. Wood EC, Rosenbaum P. The Gross Motor Function Classification System for Cerebral Palsy: A study of reliability and stability over time. Dev Med Child Neurol 2000;**42**:292-96.
52. Oeffinger D, Tylkowski C, Rayens M, et al. Gross Motor Function Classification System and outcome tools for assessing ambulatory cerebral palsy: A multicenter study. Dev Med Child Neurol 2004;**46**:311-19.
53. Palisano RJ, Rosenbaum P, Bartlett D, et al. Content validity of the expanded and revised Gross Motor Function Classification System. Dev Med Child Neurol 2008;**50**(10):744-50.
54. Gorter J, Rosenbaum P, Palisano R, et al. Limb distribution, motor impairment and functional classification of cerebral palsy. Dev Med Child Neurol 2004;**46**:461-67.
55. Surveillance of Cerebral Palsy in E. Surveillance of cerebral palsy in Europe: a collaboration of cerebral palsy surveys and registers. Surveillance of Cerebral Palsy in Europe (SCPE). Dev Med Child Neurol 2000;**42**(12):816.
56. Monbaliu E, Ortibus E, De Cat J, et al. The Dyskinesia Impairment Scale: a new instrument to measure dystonia and choreoathetosis in dyskinetic cerebral palsy. Dev Med Child Neurol 2012;**54**(3):278-83.
57. Monbaliu E, de Cock P, Ortibus E, et al. Clinical patterns of dystonia and choreoathetosis in participants with dyskinetic cerebral palsy. Dev Med Child Neurol 2016;**58**(2):138-44.
58. Harvey A, Graham H, Morris M, et al. The Functional Mobility Scale: Ability to detect change following single event multilevel surgery. Dev Med Child Neurol 2007;**49**:603-7.
59. Harvey AR, Morris ME, Graham HK, et al. Reliability of the functional mobility scale for children with cerebral palsy. Phys Occup Ther Pediatr 2010;**30**(2):139-49.
60. Rodda J, Graham HK. Classification of gait patterns in spastic hemiplegia and spastic diplegia: a basis for a management algorithm. Eur J Neurol 2001;**8** (Suppl 5):98-108.
61. Dobson F, Morris ME, Baker R, et al. Gait classification in children with cerebral palsy: A systematic review. Gait Posture 2007;**25**(1):140-52.
62. Dobson F, Morris ME, Baker R, et al. Clinician agreement on gait pattern ratings in children with spastic hemiplegia. Dev Med Child Neurol 2006;**48**(6):429-35.
63. Stott NS, Atherton WG, Mackey AH, et al. The reliability and validity of assessment of sagittal plane deviations in children who have spastic diplegia. Arch Phys Med Rehabil 2005;**86**(12):2337-41.
64. Eliasson AC, Krumlinde-Sundholm L, Rosblad B, et al. The Manual Ability Classification System (MACS) for children with cerebral palsy: scale development and evidence of validity and reliability. Dev Med Child Neurol 2006;**48**(7):549-54.
65. Morris C, Kurinczuk JJ, Fitzpatrick R, et al. Reliability of the manual ability classification system for children with cerebral palsy. Dev Med Child Neurol 2006;**48**(12):950-53.
66. Hidecker MJC, Paneth N, Rosenbaum PL, et al. Developing and validating the Communication Function Classification System for individuals with cerebral palsy. Dev Med Child Neurol 2011;**53**(8):704-10.
67. Barty E, Caynes K, Johnston LM. Development and reliability of the Functional Communication Classification System for children with cerebral palsy. Dev Med Child Neurol 2016;**58**(10):1036-41.
68. Pennington L, Virella D, Mjøen T, et al. Development of The Viking Speech Scale to classify the speech of children with cerebral palsy. Res Dev Disabil 2013;**34**(10):3202-10.
69. Sellers D, Mandy A, Pennington L, et al. Development and reliability of a system to classify the eating and drinking ability of people with cerebral palsy. Dev Med Child Neurol 2014;**56**(3):245-51.
70. Ashwal S, Russman BS, Blasco PA, et al. Practice parameter: diagnostic assessment of the child with cerebral palsy. Neurology 2004;**62**(6):851-63.
71. Holmes CJ, Hoge R, Collins L, et al. Enhancement of MR images using registration for signal averaging. J Comput Assist Tomogr 1998;**22**(2):324-33.
72. Fiori S, Cioni G, Klingels K, et al. Reliability of a novel, semi-quantitative scale for classification of structural brain magnetic resonance imaging in children with cerebral palsy. Dev Med Child Neurol 2014;**56**(9):839-45.

For peer review only - http://bmjopen.bmj.com/site/about/guidelines.xhtml

73. Fiori S, Guzzetta A, Pannek K, et al. Validity of semi-quantitative scale for brain MRI in unilateral cerebral palsy due to periventricular white matter lesions: Relationship with hand sensorimotor function and structural connectivity. NeuroImage Clinical 2015;**8**:104-9.
74. Pannek K, Raffelt D, Bell C, et al. HOMOR: higher order model outlier rejection for high b-value MR diffusion data. Neuroimage 2012;**63**(2):835-42.
75. Tournier JD, Calamante F, Connelly A. Robust determination of the fibre orientation distribution in diffusion MRI: non-negativity constrained super-resolved spherical deconvolution. Neuroimage 2007;**35**(4):1459-72.
76. Pannek K, Mathias JL, Bigler ED, et al. An automated strategy for the delineation and parcellation of commissural pathways suitable for clinical populations utilising high angular resolution diffusion imaging tractography. Neuroimage 2010;**50**(3):1044-53.
77. Rose S, Rowland T, Pannek K, et al. Structural hemispheric asymmetries in the human precentral gyrus hand representation. Neuroscience 2012;**210**:211-21.
78. Cornell MS, Hatrick NC, Boyd R, et al. The hip in children with cerebral palsy. Predicting the outcome of soft tissue surgery. Clin Orthop Relat Res 1997;**340**(340):165-71.
79. Faraj S, Atherton WG, Stott NS. Inter- and intra-measurer error in the measurement of Reimers' hip migration percentage. J Bone Joint Surg Br 2004;**86**(3):434-37.
80. Parrott J, Boyd RN, Dobson F, et al. Hip displacement in spastic cerebral palsy: repeatability of radiologic measurement. J Pediatr Orthop 2002;**22**(5):660.
81. Scrutton D, Baird G. Surveillance measures of the hips of children with bilateral cerebral palsy. Arch Dis Child 1997;**76**(4):381-84.
82. Scrutton D, Baird G, Smeeton N. Hip dysplasia in bilateral cerebral palsy: incidence and natural history in children aged 18 months to 5 years. Dev Med Child Neurol 2001;**43**(9):586-600.
83. Boyd RN, Nattrass G, Graham HK, et al. The effect of botulinum toxin type A and a variable hip abduction orthosis on gross motor function: a randomized controlled trial. Eur J Neurol 2001;**8** (Suppl 5):109-19.
84. Stevenson RD. Use of Segmental Measures to Estimate Stature in Children with Cerebral Palsy. Arch Pediatr Adolesc Med 1995;**149**:658-62.
85. Ogden CL, Kuczmarski RJ, Flegal KM, et al. Centers for Disease Control and Prevention 2000 growth charts for the United States: improvements to the 1977 National Center for Health Statistics version. Pediatrics 2002;**109**(1):45-60.
86. Russell D, Rosenbaum P, Avery L, et al. *Gross motor function measure (GMFM-66 and GMFM-88) user's manual*: MacKeith press, 2002.
87. Holmefur M, Krumlinde-Sundholm L, Bergstrom J, et al. Longitudinal development of hand function in children with unilateral cerebral palsy. Dev Med Child Neurol 2010;**52**(4):352-7.
88. Elvrum A-KG. Assessment of hand function in children with bilateral cerebral palsy: Development and measurement properties of outcome measures and classifications [Degree of Philosophiae Doctor]. Norwegian University of Science and Technology, 2016.
89. Oldfield RC. The assessment and analysis of handedness: the Edinburgh inventory. Neuropsychologia 1971;**9**(1):97-113.
90. Auld ML, Boyd RN, Moseley GL, et al. Impact of tactile dysfunction on upper-limb motor performance in children with unilateral cerebral palsy. Arch Phys Med Rehabil 2012;**93**(4):696-702.
91. Sakzewski L, Ziviani J, Boyd R. The relationship between unimanual capacity and bimanual performance in children with congenital hemiplegia. Dev Med Child Neurol 2010;**52**(9):811-16.
92. Hägglund G, Lauge-Pedersen H, Wagner P. Characteristics of children with hip displacement in cerebral palsy. BMC Musculoskelet Disord 2007;**8**(1):101-01.
93. Wynter M, Gibson N, Kentish M, et al. The development of Australian Standards of Care for Hip Surveillance in Children with Cerebral Palsy: how did we reach consensus? J Pediatr Rehabil Med 2011;**4**(3):171-82.
94. Reimers J, Bialik V. Influence of femoral rotation on the radiological coverage of the femoral head in children. Pediatr Radiol 1981;**10**(4):215-18.
95. Foroohar A, McCarthy JJ, Yucha D, et al. Head-shaft angle measurement in children with cerebral palsy. J Pediatr Orthop 2009;**29**(3):248-50.

For peer review only - http://bmjopen.bmj.com/site/about/guidelines.xhtml

96. Hilgenreiner H. Early diagnosis and early treatment of congenital dislocation of the hip. Med Klin 1925;**21**:1385-425.
97. Robin J, Graham HK, Selber P, et al. Proximal femoral geometry in cerebral palsy: a population-based cross-sectional study. J Bone Joint Surg Br 2008;**90**(10):1372-79.
98. Birkenmaier C, Jorysz G, Volkmar J, et al. Normal development of the hip: a geometric analysis based on planimetric radiography. J Pediatr Orthop B 2010;**19**(1):1-8.
99. Haike H, Breuckmann G, Schulze H. [Surgical treatment of so-called congenital hip dislocation]. Arch Orthop Unfallchir 1969;**66**(4):277-85.
100. Morrissy RT, Goldsmith G, Hall E, et al. Measurement of the Cobb angle on radiographs of patients who have scoliosis. Evaluation of intrinsic error. J Bone Joint Surg 1990;**72**(3):320-27.
101. Henderson RC, Henderson BA, Kecskemethy HH, et al. Adaptation of the lateral distal femur DXA scan technique to adults with disabilities. J Clin Densitom 2015;**18**(1):102-8.
102. Henderson RC, Lark RK, Newman JE, et al. Pediatric reference data for dual X-ray absorptiometric measures of normal bone density in the distal femur. AJR Am J Roentgenol 2002;**178**(2):439-43.
103. Mueske NM, Chan LS, Wren TA. Reliability of lateral distal femur dual-energy X-ray absorptiometry measures. J Clin Densitom 2014;**17**(4):522-7.
104. Zemel BS, Stallings VA, Leonard MB, et al. Revised pediatric reference data for the lateral distal femur measured by Hologic Discovery/Delphi dual-energy X-ray absorptiometry. J Clin Densitom 2009;**12**(2):207-18.
105. Carter DR, Bouxsein ML, Marcus R. New approaches for interpreting projected bone densitometry data. J Bone Miner Res 1992;**7**(2):137-45.
106. Australian Radiation Protection and Nuclear Safety Agency. Code of practice: Exposure of humans to ionizing radiation for research purposes. Radiation Protection Series No 8. Canberra, Australia, 2005.
107. Duke PM, Litt IF, Gross RT. Adolescents' Self-Assessment of Sexual Maturation. Pediatrics 1980;**66**(6):918.
108. Varni JW, Thompson KL, Hanson V. The Varni/Thompson Pediatrie Pain Questionnaire. I. Chronic musculoskeletal pain in juvenile rheumatoid arthritis. Pain 1987;**28**(1):27-38.
109. Gragg RA, Rapoff MA, Danovsky MB, et al. Assessing Chronic Musculoskeletal Pain Associated with Rheumatic Disease: Further Validation of the Pediatric Pain Questionnaire. J Pediatr Psychol 1996;**21**(2):237-50.
110. Kainz H, Modenese L, Lloyd DG, et al. Joint kinematic calculation based on clinical direct kinematic versus inverse kinematic gait models. J Biomech 2016;**49**(9):1658-69.
111. Steele KM, Rozumalski A, Schwartz MH. Muscle synergies and complexity of neuromuscular control during gait in cerebral palsy. Dev Med Child Neurol 2015;**57**(12):1176-82.
112. Delp SL, Anderson FC, Arnold AS, et al. OpenSim: open-source software to create and analyze dynamic simulations of movement. IEEE Trans Biomed Eng 2007;**54**(11):1940-50.
113. Baker R, McGinley JL, Schwartz MH, et al. The gait profile score and movement analysis profile. Gait Posture 2009;**30**(3):265-9.
114. Lichtwark GA, Wilson AM. Interactions between the human gastrocnemius muscle and the Achilles tendon during incline, level and decline locomotion. J Exp Biol 2006;**209**(21):4379-88.
115. Cronin NJ, Carty CP, Barrett RS, et al. Automatic tracking of medial gastrocnemius fascicle length during human locomotion. J Appl Physiol 2011;**111**(5):1491-96.
116. Gillett JG, Barrett RS, Lichtwark GA. Reliability and accuracy of an automated tracking algorithm to measure controlled passive and active muscle fascicle length changes from ultrasound. Comput Methods Biomech Biomed Engin 2013;**16**(6):678-87.
117. Barber L, Barrett R, Lichtwark G. Validation of a freehand 3D ultrasound system for morphological measures of the medial gastrocnemius muscle. J Biomech 2009;**42**(9):1313-19.
118. Verschuren O, Ketelaar M, Keefer D, et al. Identification of a core set of exercise tests for children and adolescents with cerebral palsy: a Delphi survey of researchers and clinicians. Dev Med Child Neurol 2011;**53**(5):449-56.
119. Maher CA, Williams MT, Olds TS. The six-minute walk test for children with cerebral palsy. Int J Rehabil Res 2008;**31**(2):185-8.

For peer review only - http://bmjopen.bmj.com/site/about/guidelines.xhtml

120. Li AM, Yin J, Au JT, et al. Standard reference for the six-minute-walk test in healthy children aged 7 to 16 years. Am J Respir Crit Care Med 2007;**176**(2):174-80.
121. ATS statement. Am J Respir Crit Care Med 2002;**166**(1):111-7.
122. Verschuren O, Takken T, Ketelaar M, et al. Reliability for running tests for measuring agility and anaerobic muscle power in children and adolescents with cerebral palsy. Pediatr Phys Ther 2007;**19**(2):108-15.
123. Verschuren O, Bongers BC, Obeid J, et al. Validity of the muscle power sprint test in ambulatory youth with cerebral palsy. Pediatr Phys Ther 2013;**25**(1):25-8.
124. Thompson P, Beath T, Bell J, et al. Test-retest reliability of the 10-metre fast walk test and 6-minute walk test in ambulatory school-aged children with cerebral palsy. Dev Med Child Neurol 2008;**50**(5):370-6.
125. Verschuren O, Ketelaar M, Takken T, et al. Reliability of hand-held dynamometry and functional strength tests for the lower extremity in children with Cerebral Palsy. Disabil Rehabil 2008;**30**(18):1358-66.
126. Trost SG, McIver KL, Pate RR. Conducting accelerometer-based activity assessments in field-based research. Med Sci Sports Exerc 2005;**37**(11 Suppl):S531-43.
127. Clanchy K, Tweedy S, Boyd R. Measurement of Habitual Physical Activity Performance in Adolescents with Cerebral Palsy. Dev Med Child Neurol 2011;**53**(6):499-505.
128. Clanchy KM, Tweedy SM, Boyd RN, et al. Validity of accelerometry in ambulatory children and adolescents with cerebral palsy. Eur J Appl Physiol 2011;**111**(12):2951-9.
129. National Health and Medical Research Council. Nutrient Reference Values for Australia and New Zealand. Canberra, Australia: Department of Health and Ageing, 2005.
130. Taylor C, Lamparello B, Kruczek K, et al. Validation of a food frequency questionnaire for determining calcium and vitamin D intake by adolescent girls with anorexia nervosa. J Am Diet Assoc 2009;**109**(3):479-85, 85 e1-3.
131. Harrison SL, Buettner PG, Maclennan R. The North Queensland "Sun-Safe Clothing" study: design and baseline results of a randomized trial to determine the effectiveness of sun-protective clothing in preventing melanocytic nevi. Am J Epidemiol 2005;**161**(6):536-45.
132. Sheppard J. *Dysphagia Disorders Survey and Dysphagia Management Staging Scale (Adult and Pediatric Applications): User's Manual: Australian Edition.* Ryde, NSW: The Centre for Developmental Disability 2003.
133. Sheppard JJ, Hochman R, Baer C. The Dysphagia Disorder Survey: Validation of an assessment for swallowing and feeding function in developmental disability. Res Dev Disabil 2014;**35**(5):929-42.
134. Benfer K, Weir K, Bell K, et al. Longitudinal cohort protocol study of oropharyngeal dysphagia: relationships to gross motor attainment, growth and nutritional status in preschool children with cerebral palsy. BMJ Open 2012;**2**(4):e001460.
135. Calis EA, Veugelers R, Sheppard JJ, et al. Dysphagia in children with severe generalized cerebral palsy and intellectual disability. Dev Med Child Neurol 2008;**50**(8):625-30.
136. Sheppard J. Nutritional correlates of dysphagia in individuals institutionalized with mental retardation. Dysphagia 1998;**3**:85-9.
137. Skuse D, Stevenson J, Reilly S, et al. Schedule for oral-motor assessment (SOMA): Methods of validation. Dysphagia 1995;**10**:192-202.
138. Weir K, McMahon S, Barry L, et al. Clinical signs and symptoms of oropharyngeal aspiration and dysphagia in children. Eur Respir J 2009;**33**(3):604-11.
139. DeMatteo C, Matovich D, Hjartson A. Comparison of clinical and videofluoroscopic evaluation of children with feeding and swallowing difficulties. Dev Med Child Neurol 2005;**47**(3):149-57.
140. Lefton-Greif M, McGrath-Morrow S. Deglutition and respiration: development, coordination, and practical implications. . Semin Speech Lang 2007;**28**:166-79.
141. Thomas-Stonell N, Greenberg J. Three treatment approaches and clinical factors in the reduction of drooling. Dysphagia 1998;**3**:73-8.
142. Benfer KA, Weir KA, Bell KL, et al. Longitudinal Study of Oropharyngeal Dysphagia in Preschool Children With Cerebral Palsy. Arch Phys Med Rehabil 2016;**97**(4):552-60 e9.

For peer review only - http://bmjopen.bmj.com/site/about/guidelines.xhtml

143. Lefton-Greif MA, Okelo SO, Wright JM, et al. Impact of children's feeding/swallowing problems: validation of a new caregiver instrument. Dysphagia 2014;**29**(6):671-7.
144. Semel E, Wiig E, Secord W. *Clinical Evaluation of Language Fundamentals*. 4th edition ed. Marrickville Australia: Harcourt Assessment, Inc, 2006.
145. Fenson L, Marchmann V, Thal D, et al. *McArthur Bates Communicative Development Inventory*. 2nd edition ed. Baltimore, Maryland.: Paul H Brookes Publishing Co, 2007.
146. Hayden D, Square P. *Verbal Motor Performance Assessment for Children: Examiner's Manual.* USA.: Psychological Corporation, 1999.
147. Haley SM, Coster WJ, Dumas HM, et al. *Pediatric Evaluation of Disability Inventory Computer Adaptive Test, Development, Standardization and Administration Manual Version 1.3.6*. Boston, MA: CRECare LLC, 2012.
148. Haley SM, Andrellos PJ, Coster W, et al. *Pediatric Evaluation of Disability Inventory*: New England Medical Center Hospitals, 1992.
149. Coster W, Bedell G, Law M, et al. Psychometric evaluation of the Participation and Environment Measure for Children and Youth. Dev Med Child Neurol 2011;**53**(11):1030-7.
150. Goodman R. The Strengths and Difficulties Questionnaire: A research note. Journal of Child Psychology and Psychiatry 1997;**38**(5):581-86.
151. Goodman R. The extended version of the Strengths and Difficulties Questionnaire as a guide to psychiatric caseness and consequent burden. Journal of Child Psychology and Psychiatry 1999;**40**(5):791-99.
152. Goodman R. Psychometric propeties of the Strengths and Difficulties Questionnaire. J Am Acad Child Adolesc Psychiatry 2001;**40**:1337-45.
153. Australian Government. A Snapshot of Early Childhood Development in Australia 2012 - AEDI National Report. Canberra: The Australian Government, 2009.
154. Australian Curriculum Assessment and Reporting Authority. Measurement Framework for Schooling in Australia. Sydney, Australia: Education Council, 2013.
155. Cotton SM, Kiely PM, Crewther DP, et al. A normative and reliability study for the Raven's Coloured Progressive Matrices for primary school aged children from Victoria Australia. Pers Individ Dif 2005;**39**(3):647-59.
156. Pueyo R, Junqué C, Vendrell P, et al. Raven's coloured progressive matrices as a measure of cognitive functioning in cerebral palsy. J Intellect Disabil Res 2008;**52**(5):437-45.
157. Gioia G, Isquith P, Guy S, et al. *Behavior Rating Inventory of Executive Function*. Lutz , Florida: Parnic, 2000.
158. Reynolds C, Kamphaus R. *Behavior Assessment System for children.*: Circle Pines, MN: American Guidance Service, 1992.
159. Achenbach T. *Integrative Guide to the 1991 CBCL/4-18, YSR, and TRF Profiles.* Burlington, VT: University of Vermont,: Department of Psychology 1991.
160. Conners CK. *Conner's Rating Scales-Revised Technical Manual*. Toronto: Multi-Health Systems 1997.
161. Allison C, Auyeung B, Baron-Cohen S. Toward brief "red flags" for autism screening: the short autism spectrum quotient and the short quantitative checklist in 1,000 cases and 3,000 controls. J Am Acad Child Adolesc Psychiatry 2012;**51**(2):202-12. e7.
162. Newman CJ, O'Regan M, Hensey O. Sleep disorders in children with cerebral palsy. Dev Med Child Neurol 2006;**48**(07):564-68.
163. Bruni O, Ottaviano S, Guidetti V, et al. The Sleep Disturbance Scale for Children (SDSC). Construction and validation of an instrument to evaluate sleep disturbances in childhood and adolescence. J Sleep Res 1996;**5**(4):251-61.
164. Davis E, Shelly A, Waters E, et al. Quality of life for adolescents with cerebral palsy: Perspectives of adolescents and parents. Dev Med Child Neurol 2009;**51**:193.
165. Shelly A, Davis E, Waters E, et al. The relationship between quality of life and functioning for children with cerebral palsy. Dev Med Child Neurol 2008;**50**(3):199-203.
166. Waters E, Davis E, Mackinnon A, et al. Psychometric properties of the quality of life questionnaire for children with CP. Dev Med Child Neurol 2007;**49**(1):49-55.

For peer review only - http://bmjopen.bmj.com/site/about/guidelines.xhtml

167. Olree KS, Engsberg JR, Ross SA, et al. Changes in synergistic movement patterns after selective dorsal rhizotomy. Dev Med Child Neurol 2000;**42**(5):297-303.
168. Stevens K. Developing a descriptive system for a new preference- based measure of health related quality of life forchildren. Qual Life Res 2009;**18**:1105-13.
169. Stevens K. Working with children to develop dimensions for a preference-based generic paediatric health related quality of life measure. Qual Life Res 2010;**20**:340-51.
170. Stevens K. Assessing the performance of a new generic measure of health-related quality of life for children and refining it for use in health state valuation Applied Health Economics and Health Policy 2011;**9**(3):157-69.
171. Brooks R. EuroQol: the current state of play. Health Policy 1996;**37**(1):53-72.
172. Herdman M, Gudex C, Lloyd A, et al. Development and preliminary testing of the new five-level version of EQ-5D (EQ-5D-5L). Qual Life Res 2011;**20**(10):1727-36.
173. Norman R, Cronin P, Viney R. A Pilot Discrete Choice Experiment to Explore Preferences for EQ-5D-5L Health States. Applied Health Economics and Health Policy 2013;**11**(3):287-98.
174. Al-Janabi H, Coast J, Flynn TN. What do people value when they provide unpaid care for an older person? A meta-ethnography with interview follow-up. Soc Sci Med 2008;**67**(1):111-21.
175. Al-Janabi H, Flynn TN, Coast J. Estimation of a preference-based carer experience scale. Med Decis Making 2011;**31**(3):458-68.
176. Brehaut JC, Kohen DE, Raina P, et al. The Health of Primary Caregivers of Children With Cerebral Palsy: How Does It Compare With That of Other Canadian Caregivers? Pediatrics 2004;**114**(2):e182-e91.
177. Epstein NB, Baldwin LM, Bishop DS. The McMaster family assessment device. J Marital Fam Ther 1983;**9**(2):171-80.
178. Miller IW, Epstein NB, Bishop DS, et al. The McMaster family assessment device: reliability and validity. J Marital Fam Ther 1985;**11**(4):345-56.
179. Byles J, Byrne C, Boyle MH, et al. Ontario Child Health Study: Reliability and Validity of the General Functioning Subscale of the McMaster Family Assessment Device. Fam Process 1988;**27**(1):97-104.
180. Rosenbaum PL, Walter SD, Hanna SE, et al. Prognosis for gross motor function in cerebral palsy: creation of motor development curves. J Am Med Assoc 2002;**288**(11):1357-63.

For peer review only

For peer review only - http://bmjopen.bmj.com/site/about/guidelines.xhtml

Figure 1: Summary of surveillance and outcome measures for PREDICT study

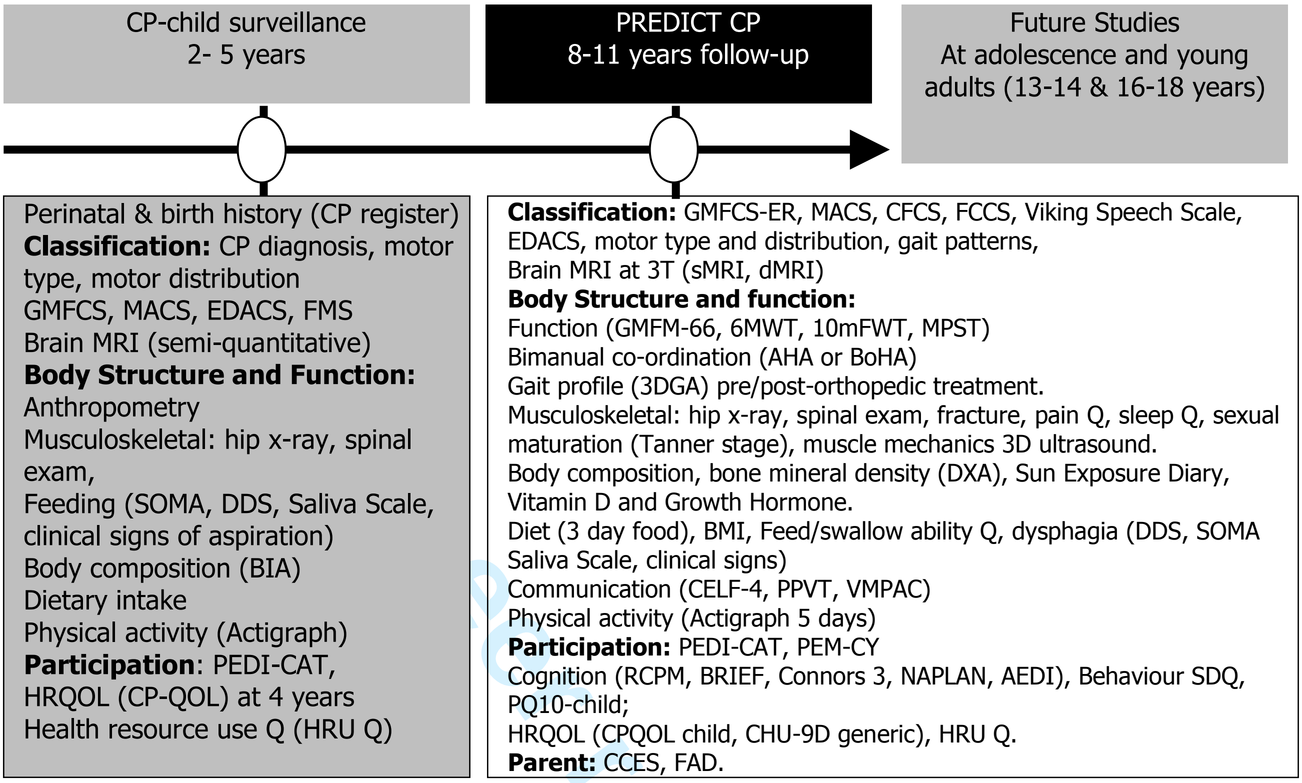

Key: AHA=Assisting hand Assessment; AEDI=Australian Early Development Index; BIA=Bioelectrical Impedance Analysis; BMI=Body Mass Index; BoHA=Both Hands Assessment; BRIEF=Behaviour Rating Inventory of Executive Function; CELF-4=Clinical Evaluation of Language Fundamentals-4; CFCS=Communication Function Classification System; CHU9D=Child Health Utility 9D; CCES=Carer Experience scale; CP-QOL=Cerebral Palsy Quality of Life Scale; 3DGA=3 dimensional gait analysis; DDS=Dysphagia Disorders Survey; dMRI=diffusion MRI; DXA=Dual-energy X-ray Absorptiometry; FAD=McMaster Family Assessment Device General Functioning Scale; FMS=Functional Mobility Scale; FCCS=Functional Communication Classification System; EDACS=Eating & Drinking Ability Classification System; GMFCS-ER=Gross Motor Function Classification System Expanded & Revised; GMFM-66=Gross Motor Ability Estimator; HRQOL=Health Related Quality of Life; MACS=Manual Ability Classification System; MPST=Muscle Power Sprint test; 6MWT=Six Minute Walk Test; 10mFWT=10 Metre Fast Walk test; NAPLAN=National Assessment Program (Literacy and Numeracy); PEDI-CAT=Paediatric Evaluation of Disability Inventory Computer Adapted Test; PEM-CY= Participation & Environment Measure for Children and Youth; PQ10-child=Autism Spectrum Quotient child; RCPM= Raven Coloured Progressive Matrices; SDQ=Strengths & Difficulties Q; PPVT= Peabody Picture Vocabulary Test; SOMA=Schedule for Oral Motor Assessment; VMPAC=Verbal Motor Performance Assessment for Children.

For peer review only - http://bmjopen.bmj.com/site/about/guidelines.xhtml

Appendix 1 PREDICT: Demographic questionnaire

What is your relationship to this child?

( ) Mother (biological or adoptive)

( ) Father (biological or adoptive)

( ) Step mother

( ) Step Father

( ) Legal Guardian (please explain)

What is your date of birth?

Does your child have any other difficulties, in addition to Cerebral Palsy, which would affect their participation in home, school or community activities (please tick any that apply)?

( ) Epilepsy

( ) Autism Spectrum Disorder (including Asperger Syndrome)

( ) ADHD

( ) Hearing Impairment

( ) Intellectual Impairment

( ) Learning Disability

( ) Visual Impairment

( ) Other (please explain)

Which best describes the household in which your child is currently living?

( ) Original, two parent family (both biological or adoptive parents are living in the same household as the child)

( ) Step, two parent family (two parents are living in the same household as the child and one is a step-parent)

( ) Shared custody (child lives in two separate households and divides his/her time between them)

( ) Sole parent family (Child lives with one parent only, may have contact with other parent)

For peer review only

For peer review only - http://bmjopen.bmj.com/site/about/guidelines.xhtml

( ) Other (please explain)

What is your current marital status?

( ) Married

( ) Defacto

( ) Separated

( ) Divorced

( ) Never married/defacto

( ) Widow/er

Does your child have any siblings living in the same household?

( ) Yes

( ) No

For each sibling living in the same household with your children please indicate the following:

	Sibling 1	Sibling 2	Sibling 3	Sibling 4
Age:				
Gender:				
Any difficulties that would affect their participation in home, school or community activities (please tick any that apply)?	( ) Nil<br>( ) Epilepsy<br>( ) Autism Spectrum Disorder (including Asperger Syndrome<br>( ) ADH<br>( ) Hearing Impairment<br>( ) Intellectual Impairment<br>( ) Learning	( ) Nil<br>( ) Epilepsy<br>( ) Autism Spectrum Disorder (including Asperger Syndrome<br>( ) ADH<br>( ) Hearing Impairment<br>( ) Intellectual Impairment<br>( ) Learning	( ) Nil<br>( ) Epilepsy<br>( ) Autism Spectrum Disorder (including Asperger Syndrome<br>( ) ADH<br>( ) Hearing Impairment<br>( ) Intellectual Impairment<br>( ) Learning	( ) Nil<br>( ) Epilepsy<br>( ) Autism Spectrum Disorder (including Asperger Syndrome<br>( ) ADH<br>( ) Hearing Impairment<br>( ) Intellectual Impairment<br>( ) Learning

For peer review only - http://bmjopen.bmj.com/site/about/guidelines.xhtml

	Disability	Disability	Disability	Disability
	( ) Speech Language Difficulty	( ) Speech Language Difficulty	( ) Speech Language Difficulty	( ) Speech Language Difficulty
	( ) Visual Impairment	( ) Visual Impairment	( ) Visual Impairment	( ) Visual Impairment
	( ) Other (please explain)	( ) Other (please explain)	( ) Other (please explain)	( ) Other (please explain)

For peer review only

Age:

Gender:

Any difficulties that would affect their participation in home, school or community activities (please tick any that apply)?

( ) Epilepsy

( ) Autism Spectrum Disorder (including Asperger Syndrome)

( ) ADHD

( ) Hearing Impairment

( ) Intellectual Impairment

( ) Learning Disability

( ) Speech/Language Difficulty

( ) Visual Impairment

( ) Other (please explain)

Is English the main language spoken at home?

( ) Yes

( ) No, please specific the main language:

For peer review only - http://bmjopen.bmj.com/site/about/guidelines.xhtml

What is your postcode?

What is *your* highest level of education?

( ) Less than year 10

( ) Year 10/11

( ) Year 12

( ) Trade/apprenticeship

( ) TAFE/college certificate

( ) University degree

( ) University postgraduate degree

What is *your partner's* highest level of education?

( ) Less than year 10

( ) Year 10/11

( ) Year 12

( ) Trade/apprenticeship

( ) TAFE/college certificate

( ) University degree

( ) University postgraduate degree

Which best describes your current employment?

( ) Full time

( ) Part time

( ) Casual

( ) Full time parent/ home duties

( ) Unemployed (seeking work)

For peer review only - http://bmjopen.bmj.com/site/about/guidelines.xhtml

Which best describes your partner's current employment?

( ) Full time

( ) Part time

( ) Full time parent/ home duties

( ) Unemployed (seeking work)

Which best describes your family's combined annual income?

( ) <25,000

( ) 25,000-50,000

( ) 50,000-75,000

( ) 75,000-100,000

( ) 100,000-150,000

( ) 150,000+

What type of school does your child attend?

( ) State School

( ) Catholic School

( ) Independent (Private) School

( ) Special School

( ) Home schooled

( ) Other (please explain)

If your child does not attend a Special School, are they in a special class?

( ) Yes, my child is in a separate class for children with special needs

( ) No, my child is taught with the rest of his/her grade

Which grade is your child currently in?

For peer review only - http://bmjopen.bmj.com/site/about/guidelines.xhtml

( ) Prep

( ) 1

( ) 2

( ) 3

( ) 4

What support does your child receive at school? (Please tick all that apply)

( ) Nil

( ) Teacher Aide

( ) Learning support teacher/Specialist teacher

( ) School Nurse

( ) Occupational Therapy

( ) Physiotherapy

( ) Speech Pathology

( ) Academic/ Guidance Officer

( ) Other (please explain)

Does your child have an individual education plan (IEP – an individualised plan for your child's education)?

( ) Yes

( ) No

Does your child receive any other assistance with schooling (e.g. private tuition)?

( ) Yes, please describe

( ) No

Which of the following extra-curricular activities (outside of school) does your child participate in? (please tick all that apply). Please indicate for each activity, the number of times your child would participate in the activity (either formal lessons and/or practice:

For peer review only - http://bmjopen.bmj.com/site/about/guidelines.xhtml

Extra-curricular Activities	Frequency
( ) Nil	
( ) Music Classes (e.g. singing, instrument)	( ) Monthly ( ) Weekly ( ) 2-3 times/wk
( ) Creative Arts Classes (e.g. drama, drawing)	( ) Monthly ( ) Weekly ( ) 2-3 times/wk
( ) Team Sport (e.g. soccer, cricket)	( ) Monthly ( ) Weekly ( ) 2-3 times/wk
( ) Individual Sport (e.g. swimming, martial arts)	( ) Monthly ( ) Weekly ( ) 2-3 times/wk
( ) Dance (e.g. ballet, hip hop)	( ) Monthly ( ) Weekly ( ) 2-3 times/wk
( ) Social Clubs (e.g. scouts, girl guides)	( ) Monthly ( ) Weekly ( ) 2-3 times/wk
( ) Religious classes or clubs (e.g. Sunday school)	( ) Monthly ( ) Weekly ( ) 2-3 times/wk
( ) Other (please explain)	( ) Monthly ( ) Weekly ( ) 2-3 times/wk

For peer review only

For peer review only - http://bmjopen.bmj.com/site/about/guidelines.xhtml

Appendix 2: Qld Predict CP – Physician's checklist

**Diagnosis**		COMMENTS
Cerebral palsy	yes/ no/ unsure	Definition (Rosenbaum et al., 2005): "Cerebral Palsy (CP) describes a group of disorders of the development of movement and posture, causing activity limitation that are attributed to non-progressive disturbances that occurred in the developing foetal or infant brain. The motor disorders of cerebral palsy are often accompanied by disturbances of sensation, cognition, communication, perception, and/or behaviour, and/or by a seizure disorder."<br><br>What constitutes Cerebral Palsy? (Badawi et al., 1998): Cerebral palsy (CP) is a term of convenience applied to a group of motor disorders of central origin defined by clinical description. It is not a diagnosis in that its application infers nothing about pathology, aetiology or prognosis. It is an umbrella term covering a wide range of disorders which result in childhood motor impairment. There must be motor impairment, and this impairment must stem from a malfunction of the brain (rather than spinal cord or muscles). Furthermore, the brain malfunction must be non-progressive and it must manifest early in life.

Patterns of motor impairment

			COMMENTS	
Motor type	Spastic		See attachment for further information (SCPE definitions, 2000<br><br>For mixed motor type state 1 for dominant and 2 for secondary etc.	
	Ataxic			
	dystonic			
	choreoathetotic			
	Hypotonic			
	Hyperkinetic			
Distribution	Bilateral/ unilateral			
	No of limbs (based on activity or function not passive testing of muscle tone)		1/ 2/ 3/ 4	
Head circumference –				

For peer review only - http://bmjopen.bmj.com/site/about/guidelines.xhtml

For peer review only

current (cm)			

Functional level		COMMENTS
GMFCS level	I/ II/ III/ IV/ V	
Age at classification	years months	Age of child when this assessment is being performed (and GMFCS is classified)
Upper limb/ Handedness	Right predominant/ Left predominant/ Bilateral	

Co-morbidities		COMMENTS
Epilepsy	No<br><br>Yes (defined by 2 unprovoked seizures excluding febrile or neonatal seizures)<br><br>If yes, still on medication, Yes/ No	
Seizure type		Ask the family to describe exactly what they observed.<br><br>Generalised or partial<br><br>Generalised – sudden onset of seizures that compromises responsiveness and affects the whole body.<br><br>Partial – seizures have focality therefore symptoms reflect onset in 1 part of the brain<br><br>Date of commencement of seizures
Seizure syndrome		
Controlled/ not controlled		
Medications	Yes/ no/ not applicable	Medications for seizures or any other medications

For peer review only - http://bmjopen.bmj.com/site/about/guidelines.xhtml

Visual impairment<br><br>(after correction, on the better eye)	Normal<br><br>Impaired<br><br>Severely impaired (blind or no useful vision)	
Hearing impairment (before correction, on the better ear)	Normal<br><br>Impaired<br><br>Severely impaired (hearing loss > 70 dB	
Speech	Normal<br><br>Delayed	Expressive<br><br>Receptive<br><br>Both Unclassified
Communication	Oral<br><br>Signs<br><br>Device	
Intellectual impairment	Mild<br><br>Moderate<br><br>Severe<br><br>Probably impaired, severity unknown<br><br>Probably no impairment<br><br>No impairment<br><br>Unknown	SCPE classification:<br><br>Normal- IQ>=85, attendance of regular school without support<br><br>Borderline- IQ 70 to 84<br><br>Mild impairment- IQ 50 to 69, some basic literacy and numeracy achieved<br><br>Moderate impairment- IQ 20-49<br><br>Severe impairment- IQ < 20
Nutrition		
Body weight	kg / percentile	
Body height	cm / percentile	

For peer review only - http://bmjopen.bmj.com/site/about/guidelines.xhtml

Method of nutrition	Oral<br>Tube- nasogastric<br>PEG	PEG – percutaneous endoscopic gastrostomy
	Partially<br>Entirely	

Respiratory Health		COMMENTS
No of hospitalisations for chest infections in the past 6 months (since last visit)		
No of episodes of pneumonia		
Asthma	yes/ no	
No of episodes of asthmatic attacks in the past 6 months (since last visit)		
Others	Constipation<br>Diabetes<br>Cardiac conditions	
Continence	Urinary<br>Faecal	
Past surgical history		
Examination findings		Clinical signs and symptoms
MRI previous (yes/no)<br>date<br>location		

For peer review only

Appendix 4: Qld PREDICT CP Study Health Resource Use Form

Allied Health

During the last 6 months, have you received….

1. Physiotherapy Yes No

Does it emphasise on Motor learning Functional therapy NDT therapy Postural management

How often weekly fortnightly monthly others (________)

Duration of session 30 minutes 45 minutes 60 minutes others (________)

Format individual group others (________)

Compliance very poor poor average good very good

2. Occupational therapy Yes No

Does it emphasise on CIMT therapy Goal directed training Postural management

How often weekly fortnightly monthly others (________)

Duration of session 30 minutes 45 minutes 60 minutes others (________)

Format individual group others (________)

Compliance very poor poor average good very good

3. Speech therapy Yes No

How often weekly fortnightly monthly others (________)

Duration of session 30 minutes 45 minutes 60 minutes others (________)

Format individual group others (________)

Compliance very poor poor average good very good

4. Conductive education Yes No

How often weekly fortnightly monthly others (________)

Duration of session 30 minutes 45 minutes 60 minutes others (________)

For peer review only

Format individual group others (________)

Compliance very poor poor average good very good

5. Point percussion Yes No

How often weekly fortnightly monthly others (________)

Duration of session 30 minutes 45 minutes 60 minutes others (________)

Format individual group others (________)

Compliance very poor poor average good very good

6. Acupuncture Yes No

How often weekly fortnightly monthly others (________)

Duration of session 30 minutes 45 minutes 60 minutes others (________)

Format individual group others (________)

Compliance very poor poor average good very good

7. Other therapy (________________) Yes No

How often weekly fortnightly monthly others (________)

Duration of session 30 minutes 45 minutes 60 minutes others (________)

Format individual group others (________)

Compliance very poor poor average good very good

8. Other therapy (________________) Yes No

How often weekly fortnightly monthly others (________)

Duration of session 30 minutes 45 minutes 60 minutes others (________)

Format individual group others (________)

Compliance very poor poor average good very good

For peer review only

For peer review only - http://bmjopen.bmj.com/site/about/guidelines.xhtml

9. Other therapy (________________) Yes No

How often weekly fortnightly monthly others (________)

Duration of session 30 minutes 45 minutes 60 minutes others (________)

Format individual group others (________)

Compliance very poor poor average good very good

10. Therapy Packages Yes No

Post BTX-A therapy package Yes No (see attached table)

Post surgery package Yes No (see attached table)

Compliance very poor poor average good very good

Medical

During the last 6 months, has your child had….

1. Hospital admission Yes No Number

Visit 1 Reason: ____

Length of stay: ____

Investigations performed and treatment received: ____________________

____

Visit 2 Reason: ____

Length of stay: ____

Investigations performed and treatment received: ____________________

____

Visit 3 Reason: ____

Length of stay: ____

Investigations performed and treatment received: ____________________

For peer review only - http://bmjopen.bmj.com/site/about/guidelines.xhtml

____

Visit 4 Reason: ____

Length of stay: ____

Investigations performed and treatment received: _________________________

____

2. Specialist medical appointments Yes No

Reason: ____

Length of stay: ____

Investigations performed and treatment received: _________________________

____

3. Other out-patient appointments Yes No

Reason: ____

Length of stay: ____

Investigations performed and treatment received: _________________________

____

Medication

During the last 6 months, has your child had….

1. Medication Yes No

For asthma Yes No

Which medication: ____

Frequency (number of times per day): _____________________________

Dosage (per day): ____

Duration (length of treatment): __________________________________

For peer review only - http://bmjopen.bmj.com/site/about/guidelines.xhtml

For peer review only

Any adverse events Yes No

Required admissions to hospital (see 'Medical') Yes No

2. For epilepsy Yes No

Frequency (number of times per day): ______________________________________

Dosage (per day): ________________________

Duration: ____

Any adverse events Yes No

Required admissions to hospital (see 'Medical') Yes No

3. For saliva control Yes No

Frequency (number of times per day): ___________________

Dosage (per day): ________________________

Duration: ____

Any adverse events Yes No

Required admissions to hospital (see 'Medical') Yes No

4. Others: ____

Frequency (number of times per day): ___________________

Dosage (per day): _________________________

Duration: ____

Any adverse events Yes No

Required admissions to hospital (see 'Medical') Yes No

Spasticity management/ muscle contracture management

During the last 6 months, has your child received….

1.

For peer review only - http://bmjopen.bmj.com/site/about/guidelines.xhtml

BTX-A Yes No

No of limbs treated 1 2 3 4 others (____)

No. of muscles per limb calf hamstrings adductors UL others (____)

Total dose (units BOTOX or Dysport): ____

Body weight at Rx date: ____

Units/ kilogram/ body weight: ____

Any adverse events Yes No

Required admissions to hospital (see 'Medical') Yes No

2.

Phenol Yes No

No of limbs treated 1 2 3 4 others (____)

Obturator Nerve No

Yes direct indirect other site (_________)

Total dose: ____

Any adverse events Yes No

Required admissions to hospital (see 'Medical') Yes No

3.

Oral anti-spastic medication Yes No

What medications: ____

Dosage (per day): ____

Duration: ____

Any adverse events Yes No

Required admissions to hospital (see 'Medical') Yes No

For peer review only - http://bmjopen.bmj.com/site/about/guidelines.xhtml

For peer review only

4.

Intrathecal Balcofen	Yes	No			
Dosage (per day): ________________________________					
Pump refill	Yes	No			
Any adverse events	Yes	No			
Required admissions to hospital (see 'Medical')			Yes	No	

5.

Soft tissue release	Yes	No			
What level	Hip	knee	ankle	foot	spine
Which side	unilateral	bilateral			

6.

Bony reconstruction-derotational osteotomy	Yes	No			
What level	Hip	knee	ankle	foot	spine
Which side	unilateral	bilateral			

7.

Salvage procedure	Yes	No			
What level	Hip	knee	ankle	foot	spine
Which side	unilateral	bilateral			

8.

SEMLs	Yes	No			
What level	Hip	knee	ankle	foot	spine
Which side	unilateral	bilateral			

For peer review only

For peer review only - http://bmjopen.bmj.com/site/about/guidelines.xhtml

Any adverse events	Yes	No			
Required admissions to hospital (see 'Medical')			Yes	No	

9.

Rhizotomy	Yes	No			

Date and number of rootlets cut: ______________________________________

Which side	unilateral	bilateral			
Any adverse events	Yes	No			
Required admissions to hospital (see 'Medical')			Yes	No	

10.

Serial casting	Yes	No			
No of episodes	1	2	3	others (_______)	
No. of limbs treated	1	2			
Total no of weeks done	1	2	3	4	>4
Any adverse events	Yes	No			
Required admissions to hospital (see 'Medical')			Yes	No	

Equipment

During the last 6 months, have you received….

1.

Wheelchair	Yes	No			
Is it	New	Continuing			

Frequency of use hours per day: ______________________________

days per week: ______________________________

Compliance	very poor	poor	average	good	v. good

For peer review only - http://bmjopen.bmj.com/site/about/guidelines.xhtml

2.

Sticks	Yes	No			
Is it	New	Continuing			
Frequency of use	hours per day: ________________________________				
	days per week: ________________________________				
Compliance	very poor	poor	average	good	v. good

3.

Crutches	Yes	No			
Is it	New	Continuing			
Frequency of use	hours per day: ________________________________				
	days per week: ________________________________				
Compliance	very poor	poor	average	good	v. good

4.

Kaye Walker	Yes	No			
Is it	New	Continuing			
Frequency of use	hours per day: ________________________________				
	days per week: ________________________________				
Compliance	very poor	poor	average	good	v. good

5.

Pony Walker	Yes	No
Is it	New	Continuing
Frequency of use	hours per day: ________________________________	
	days per week: ________________________________	

For peer review only - http://bmjopen.bmj.com/site/about/guidelines.xhtml

Compliance	very poor	poor	average	good	v. good

6.

David Hart Walker	Yes	No
Is it	New	Continuing

Frequency of use hours per day: ________________________________

days per week: ________________________________

Compliance	very poor	poor	average	good	v. good

7.

Special seating	Yes	No

What brand: ____

Is it	New	Continuing

Frequency of use hours per day: ________________________________

days per week: ________________________________

Compliance	very poor	poor	average	good	v. good

8.

Standing frame	Yes	No

What brand: ____

Is it	New	Continuing

Frequency of use hours per day: ________________________________

days per week: ________________________________

Compliance	very poor	poor	average	good	v. good

9.

Orthoses	Yes	No

For peer review only - http://bmjopen.bmj.com/site/about/guidelines.xhtml

Is it	New	Continuing			
Is it	fixed AFO	hinged AFO		KAFO	HKFO
	unilateral		bilateral		
	hip abduction brace (static or SWASH)				
	Soft garment/ sleeves/ UPsuit				
	Night time positioning equipment (list brand_____________)				
Which side	unilateral	bilateral			
Frequency of use	hours per day: ________________________________				
	days per week: ________________________________				
Compliance	very poor	poor	avera ge	good	v. good

For peer review only - http://bmjopen.bmj.com/site/about/guidelines.xhtml

Appendix 5: Augmentative and Alternative Communication (AAC) Questionnaire

Communication System ○ Yes ○ No

Is it:

Makaton/ formal signs: number of signs: ______

Symbols or pictures for choices: number presented: ______

Communication board: number of words/ symbols: ______

Communication book: number of words/ symbols: ______

Single message device (eg Big Mack/ Step-by-Step)

Voice output communication device: name of device ____________________________

Access:

Eye pointing

Laser/ infra-red pointer

Visual scanning

Auditory scanning

Direct access (pointing)

Frequency of use hours per day: ☐☐

days per week: ☐

For peer review only

Appendix 6: Predict CP study - 7 day physical activity monitor log

Thank you for helping with our research! This study aims to identify when your child is active and when they are inactive, so that we can work out how much movement and physical activity children with Cerebral Palsy do as part of their daily life.

To do this we are using an activity monitor together with a logbook of wear time. To help make sure our measures are as accurate as possible, please read the instructions below.

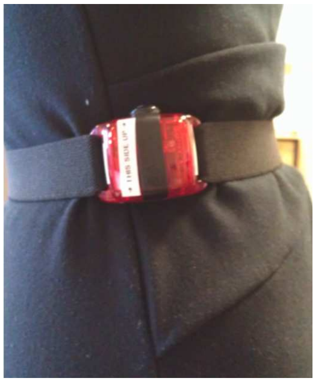

The activity monitor

Where do I wear it? The belt should be worn around your child's waist with the monitor on the side of the hip, on the dominant side. That is, wear the monitor on the (circle) RIGHT / LEFT side.

Position the Actigraph with the arrows pointing up and the open/close button on top. Remember that the monitor is already on and recording and don't worry if it flashes.

How many days should I wear this? Seven days. This should be made up of five weekdays and two weekend days.

How long do I wear it? The monitor should be worn as much as possible during waking hours. It can be hidden under clothing as long as it is on the belt and firm against the waist on the side of the hip, on the unaffected side.

Can I take the monitor off? Take the monitor off only:
When going to sleep; or for water activities such as having a shower, or swimming.

Important points:

Wear the monitor on the belt around the waist on the side of the hip, on the dominant side!
Wear the monitor all hours your child is awake, taking it off only to sleep or if having a swim or shower.

The log book

The log book allows us to compare the movement of the activity monitor to what you record doing at the time – this allows us to check it is accurate.

What do I need to do? At the top of the form are some questions about when the monitor was worn and when your child was asleep. Underneath this we then need you to we need you to record for each

For peer review only - http://bmjopen.bmj.com/site/about/guidelines.xhtml

half hour if the monitor was on or off. Under this, each block represents 15minutes of activity. Simply tick the box that corresponds to the activity – eg. Swimming, sleeping, sitting or walking.

How many days should I do this? The same seven days that your child is wearing the activity monitor.

Monitor on: _____am/pm ______________ Monitor off: _____am/pm ______________

Once you have completed the seven days, put both the activity monitor and the log book into the return mail bag provided, peel off the registered post number, and put it into a Yellow Express Post Box to return it to us.

Thank you for your help and participation in this study.

For peer review only

For peer review only

For peer review only - http://bmjopen.bmj.com/site/about/guidelines.xhtml

PREDICT CP 7- Day Sun Exposure Diary

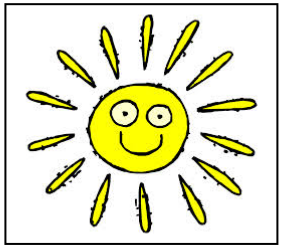

Name of Child: ……………………………………… **Date of Birth:** ….……………… **Dates of Diary:** ………………………………………

Instructions for completing diary
Please read the following instructions on how to complete your sun exposure diary.

• **Please answer all the questions on the first page**

• **For each day:** record the date

• **Time Outside in the Sun**: For each hour interval, put a cross in the box under the amount of time you spent in the sun during that interval. Please **do not leave** any hour interval(s) not ticked; i.e. tick '0 minutes' if you have not been exposed to the sun for that particular hour interval.

• **Sunscreen:** Put a cross in the 'YES" or 'NO' box on the top right of the page to indicate if you have applied sunscreen that day. If sunscreen was applied, shade in the area(s) on the diagram to reflect where you have applied sunscreen on your body that day. Please also put a cross in a box under the "**Did You Apply Sunscreen"** column, against the hour(s) of the day at which you applied sunscreen. If you did not apply sunscreen that day, do not put a cross in any of the boxes under this column

• **Clothing Worn**: Use the '**Sun Diary Clothing Guide**' to fill in the type of clothing you wore for each hour interval that day. Insert the relevant number that matches the picture for upper and lower body, headwear, and footwear. Please make sure you have specified all clothing worn at each hour interval, and that you have **written a number in every box in this section.** Please put a cross against the hour(s) that gloves have been worn that day. If no gloves have been worn, do not put a cross in any of the boxes in the gloves column.

To return the diary
A courier will collect the completed sun exposure diary from your home at a time that is convenient to you

Any questions please contact **Camilla:** 30697355, camilla.davenport@uq.edu.au

**Office Use Only**
RA signature:
RA name:
Date:
Study ID:

For peer review only

For peer review only - http://bmjopen.bmj.com/site/about/guidelines.xhtml

Questions about usual sun exposure

Please answer **all** the following questions. Please **circle** or write your answers in the space provided.

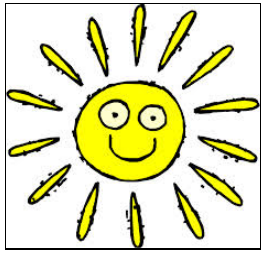

For peer review only

1. Have you been on a holiday in the sun in last month? YES / NO

2. Does your child usually wear sunscreen? YES / NO

   If **YES** what brand? ____________________

   What SPF factor? ______________________

3. What is the colour of your child's **untanned** skin?

   Very fair

   Fair

   Olive

   Light brown

   Dark Brown

For peer review only - http://bmjopen.bmj.com/site/about/guidelines.xhtml

Please refer to the following information as a guide to filling out the Sun Exposure Diary.

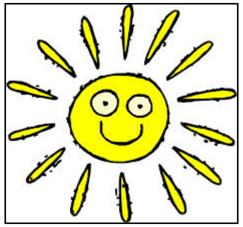

Day Example: Monday 01/07/2015

Name: Elsa Snow

Sunscreen: applied at 6.00am on face, neck, upper and lower limbs (excluding palms of hands and feet), again at 12 noon.

Time Outside in the Sun:

- 6-7 am: 15 minutes, playing **outside** at home
- 8-9 am: 20 minutes, driving to school in car
- 12-1 pm: 10 minutes, playing **outside** at school
- 1-2 pm: 10 minutes, playing **outside** at school
- 5-6 pm: 10 minutes, walking to swimming lessons **outside**
- 6-7 pm: 30 minutes, swimming lessons indoors

Type of Clothing Worn:

- 6 am to 6 pm: short-sleeved top with shorts, no headwear, enclosed shoes. These items worn until 6.00pm.
- 12-1pm & 1-2pm: “as above” with legionnaire’s cap

For peer review only - http://bmjopen.bmj.com/site/about/guidelines.xhtml
For peer review only

- 6-7 pm: swimsuit, no headwear, no foot or hand wear.

SUN DIARY CLOTHING GUIDE

**UPPER BODY**	**NO CLOTHING ON UPPER BODY**<br>**0**<br>**No upper clothing**	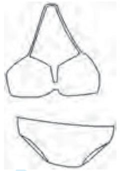<br>**1**<br>**Bikini**	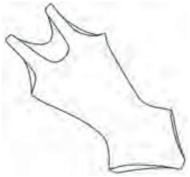<br>**2**<br>**Swimsuit**	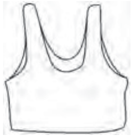<br>**3**<br>**Crop top**	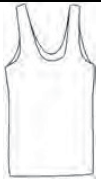<br>**4**<br>**Singlet top**	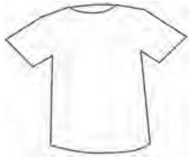<br>**5**<br>**Short-sleeved top**	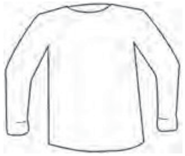<br>**6**<br>**Long-sleeved top**
**LOWER BODY**	**NO CLOTHING ON LOWER BODY**<br>**0**<br>**No lower clothing**	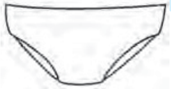<br>**1**<br>**Speedos/briefs**	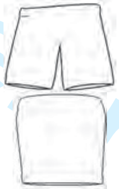<br>**2**<br>**Shorts or short skirt**	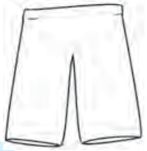<br>**3**<br>**Medium shorts or 3/4 pants**	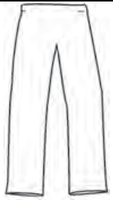<br>**4**<br>**Long trousers/ jeans**	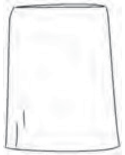<br>**5**<br>**Medium skirt**	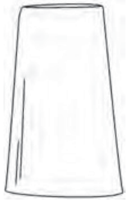<br>**6**<br>**Long skirt**
**HEADWEAR**	**NO HEADWEAR**<br>**0**<br>**No headwear**	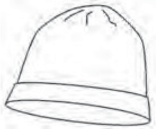<br>**1**<br>**Beanie**	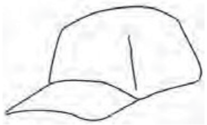<br>**2**<br>**Cap**	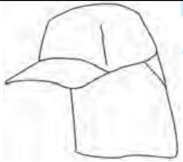<br>**3**<br>**Legionnaire's cap**	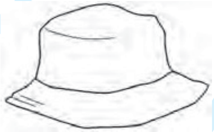<br>**4**<br>**Bucket hat**	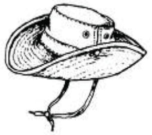<br>**5**<br>**Wide-brimmed hat**	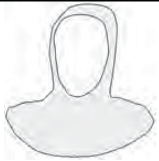<br>**6**<br>**Veil/burkha**
**FOOTWEAR**	**NO FOOTWEAR/ HANDWEAR**<br>**0**<br>**No footwear**	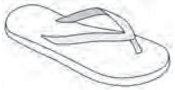<br>**1**<br>**Thong/open sandals**	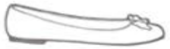<br>**2**<br>**Semi-enclosed shoes**	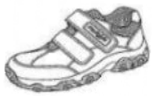<br>**3**<br>**Enclosed shoes**	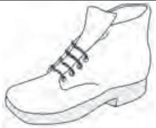<br>**4**<br>**Boots**	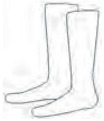<br>**5**<br>**Long socks**	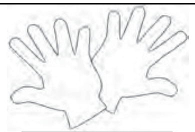<br>**HANDWEAR**<br>**Gloves**

For peer review only - http://bmjopen.bmj.com/site/about/guidelines.xhtml

DAY EXAMPLE

DATE 01 / 07 / 15

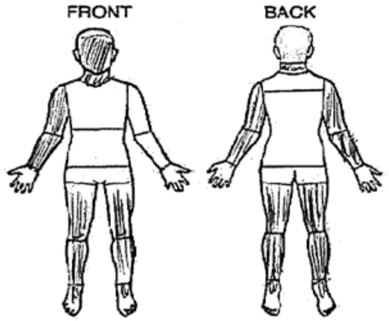

Did you apply sunscreen today?

☑ Yes ☐ No

If yes, shade the diagram on the left to show the parts of the body that you applied sunscreen to today

	TIME OUTDOORS (Cross the box which best represents the amount of time you spent outdoors during each one hour interval shown below)					DID YOU APPLY SUNSCREEN	INSERT NUMBER DENOTING TYPE OF CLOTHING WORN (You must write one number in each box)				
	0 minutes	Below 15 minutes	15-30 minutes	30-45 minutes	45-60 minutes	Cross as many as applicable	Upper body	Lower body	Head wear	Footwear	Gloves (cross all that apply)
**Morning**											
5 - 6 am	☑	☐	☐	☐	☐	☐	0	0	0	0	☐
6 - 7 am	☐	☐	☑	☐	☐	☑	5	2	0	3	☐
7 - 8 am	☑	☐	☐	☐	☐	☐	5	2	0	3	☐
8 - 9 am	☑	☐	☐	☐	☐	☐	5	2	0	3	☐
9 - 10 am	☑	☐	☐	☐	☐	☐	5	2	0	3	☐
10 - 11 am	☑	☐	☐	☐	☐	☐	5	2	0	3	☐
11 - 12 am	☑	☐	☐	☐	☐	☐	5	2	0	3	☐
**Afternoon**											
12 - 1 pm	☐	☑	☐	☐	☐	☑	5	2	3	3	☐
1 - 2 pm	☐	☑	☐	☐	☐	☐	5	2	3	3	☐
2 - 3 pm	☑	☐	☐	☐	☐	☐	5	2	0	3	☐
3 - 4 pm	☑	☐	☐	☐	☐	☐	5	2	0	3	☐
4 - 5 pm	☑	☐	☐	☐	☐	☐	5	2	0	3	☐
5 - 6 pm	☐	☑	☐	☐	☐	☐	5	2	0	3	☐
6 - 7 pm	☑	☐	☐	☐	☐	☐	2	0	0	0	☐

PREDICT_Sun Diary page 5

DAY 1
DATE _ _ / _ _ / _ _

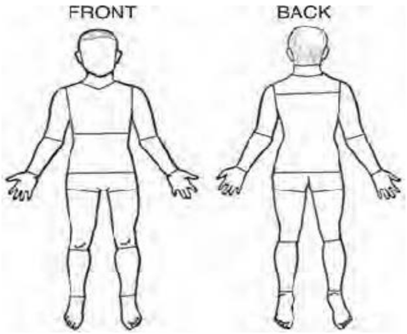

Did you apply sunscreen today?

☐ Yes ☐ No

If yes, shade the diagram on the left to show the parts of the body that you applied sunscreen to today

	**TIME OUTDOORS** (Cross the box which best represents the amount of time you spent outdoors during each one hour interval shown below)					**DID YOU APPLY SUNSCREEN**	**INSERT NUMBER DENOTING TYPE OF CLOTHING WORN** (You must write one number in each box)				
	0 minutes	Below 15 minutes	15-30 minutes	30-45 minutes	45-60 minutes	Cross as many as applicable	Upper body	Lower body	Head wear	Footwear	Gloves (cross all that apply)
**Morning**											
5 - 6 am	☐	☐	☐	☐	☐	☐					☐
6 - 7 am	☐	☐	☐	☐	☐	☐					☐
7 - 8 am	☐	☐	☐	☐	☐	☐					☐
8 - 9 am	☐	☐	☐	☐	☐	☐					☐
9 - 10 am	☐	☐	☐	☐	☐	☐					☐
10 - 11 am	☐	☐	☐	☐	☐	☐					☐
11 - 12 am	☐	☐	☐	☐	☐	☐					☐
**Afternoon**											
12 - 1 pm	☐	☐	☐	☐	☐	☐					☐
1 - 2 pm	☐	☐	☐	☐	☐	☐					☐
2 - 3 pm	☐	☐	☐	☐	☐	☐					☐
3 - 4 pm	☐	☐	☐	☐	☐	☐					☐
4 - 5 pm	☐	☐	☐	☐	☐	☐					☐
5 - 6 pm	☐	☐	☐	☐	☐	☐					☐
6 - 7 pm	☐	☐	☐	☐	☐	☐					☐

For peer review only - http://bmjopen.bmj.com/site/about/guidelines.xhtml

DAY 2

DATE _ _ / _ _ / _ _

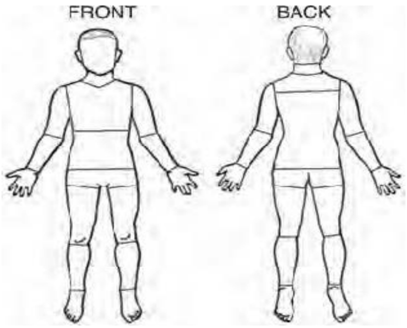

Did you apply sunscreen today?

☐ Yes ☐ No

If yes, shade the diagram on the left to show the parts of the body that you applied sunscreen to today

	TIME OUTDOORS (Cross the box which best represents the amount of time you spent outdoors during each one hour interval shown below)					DID YOU APPLY SUNSCREEN	INSERT NUMBER DENOTING TYPE OF CLOTHING WORN (You must write one number in each box)				
	0 minutes	Below 15 minutes	15-30 minutes	30-45 minutes	45-60 minutes	Cross as many as applicable	Upper body	Lower body	Head wear	Footwear	Gloves (cross all that apply)
**Morning**											
5 - 6 am	☐	☐	☐	☐	☐	☐					☐
6 - 7 am	☐	☐	☐	☐	☐	☐					☐
7 - 8 am	☐	☐	☐	☐	☐	☐					☐
8 - 9 am	☐	☐	☐	☐	☐	☐					☐
9 - 10 am	☐	☐	☐	☐	☐	☐					☐
10 - 11 am	☐	☐	☐	☐	☐	☐					☐
11 - 12 am	☐	☐	☐	☐	☐	☐					☐
**Afternoon**											
12 - 1 pm	☐	☐	☐	☐	☐	☐					☐
1 - 2 pm	☐	☐	☐	☐	☐	☐					☐
2 - 3 pm	☐	☐	☐	☐	☐	☐					☐
3 - 4 pm	☐	☐	☐	☐	☐	☐					☐
4 - 5 pm	☐	☐	☐	☐	☐	☐					☐
5 - 6 pm	☐	☐	☐	☐	☐	☐					☐
6 - 7 pm	☐	☐	☐	☐	☐	☐					☐

For peer review only - http://bmjopen.bmj.com/site/about/guidelines.xhtml

DAY 3

DATE _ _ / _ _ / _ _

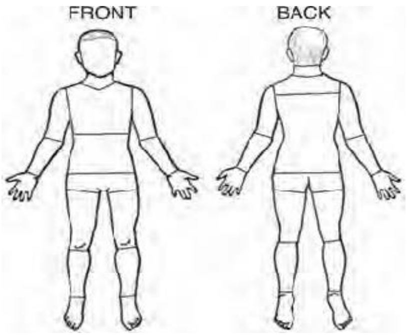

Did you apply sunscreen today?

☐ Yes ☐ No

If yes, shade the diagram on the left to show the parts of the body that you applied sunscreen to today

	TIME OUTDOORS (Cross the box which best represents the amount of time you spent outdoors during each one hour interval shown below)					DID YOU APPLY SUNSCREEN	INSERT NUMBER DENOTING TYPE OF CLOTHING WORN (You must write one number in each box)				
	0 minutes	Below 15 minutes	15-30 minutes	30-45 minutes	45-60 minutes	Cross as many as applicable	Upper body	Lower body	Head wear	Footwear	Gloves (cross all that apply)
**Morning**											
5 - 6 am	☐	☐	☐	☐	☐	☐					☐
6 - 7 am	☐	☐	☐	☐	☐	☐					☐
7 - 8 am	☐	☐	☐	☐	☐	☐					☐
8 - 9 am	☐	☐	☐	☐	☐	☐					☐
9 - 10 am	☐	☐	☐	☐	☐	☐					☐
10 - 11 am	☐	☐	☐	☐	☐	☐					☐
11 - 12 am	☐	☐	☐	☐	☐	☐					☐
**Afternoon**											
12 - 1 pm	☐	☐	☐	☐	☐	☐					☐
1 - 2 pm	☐	☐	☐	☐	☐	☐					☐
2 - 3 pm	☐	☐	☐	☐	☐	☐					☐
3 - 4 pm	☐	☐	☐	☐	☐	☐					☐
4 - 5 pm	☐	☐	☐	☐	☐	☐					☐
5 - 6 pm	☐	☐	☐	☐	☐	☐					☐
6 - 7 pm	☐	☐	☐	☐	☐	☐					☐

For peer review only

For peer review only - http://bmjopen.bmj.com/site/about/guidelines.xhtml

DAY 4

DATE _ _ / _ _ / _ _

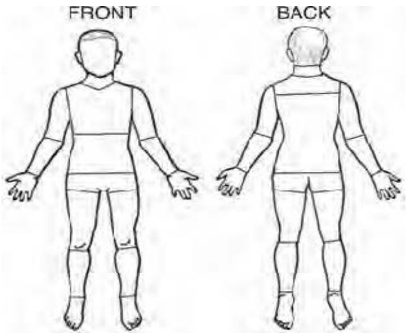

Did you apply sunscreen today?

☐ Yes ☐ No

If yes, shade the diagram on the left to show the parts of the body that you applied sunscreen to today

	**TIME OUTDOORS** (Cross the box which best represents the amount of time you spent outdoors during each one hour interval shown below)					**DID YOU APPLY SUNSCREEN**	**INSERT NUMBER DENOTING TYPE OF CLOTHING WORN** (You must write one number in each box)				
	0 minutes	Below 15 minutes	15-30 minutes	30-45 minutes	45-60 minutes	Cross as many as applicable	Upper body	Lower body	Head wear	Footwear	Gloves (cross all that apply)
**Morning**											
5 - 6 am	☐	☐	☐	☐	☐	☐					☐
6 - 7 am	☐	☐	☐	☐	☐	☐					☐
7 - 8 am	☐	☐	☐	☐	☐	☐					☐
8 - 9 am	☐	☐	☐	☐	☐	☐					☐
9 - 10 am	☐	☐	☐	☐	☐	☐					☐
10 - 11 am	☐	☐	☐	☐	☐	☐					☐
11 - 12 am	☐	☐	☐	☐	☐	☐					☐
**Afternoon**											
12 - 1 pm	☐	☐	☐	☐	☐	☐					☐
1 - 2 pm	☐	☐	☐	☐	☐	☐					☐
2 - 3 pm	☐	☐	☐	☐	☐	☐					☐
3 - 4 pm	☐	☐	☐	☐	☐	☐					☐
4 - 5 pm	☐	☐	☐	☐	☐	☐					☐
5 - 6 pm	☐	☐	☐	☐	☐	☐					☐
6 - 7 pm	☐	☐	☐	☐	☐	☐					☐

For peer review only - http://bmjopen.bmj.com/site/about/guidelines.xhtml

DAY 5

DATE _ _ / _ _ / _ _

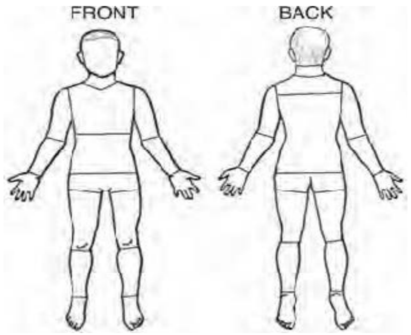

Did you apply sunscreen today?

☐ Yes ☐ No

If yes, shade the diagram on the left to show the parts of the body that you applied sunscreen to today

	TIME OUTDOORS (Cross the box which best represents the amount of time you spent outdoors during each one hour interval shown below)					DID YOU APPLY SUNSCREEN	INSERT NUMBER DENOTING TYPE OF CLOTHING WORN (You must write one number in each box)				
	0 minutes	Below 15 minutes	15-30 minutes	30-45 minutes	45-60 minutes	Cross as many as applicable	Upper body	Lower body	Head wear	Footwear	Gloves (cross all that apply)
**Morning**											
5 - 6 am	☐	☐	☐	☐	☐	☐					☐
6 - 7 am	☐	☐	☐	☐	☐	☐					☐
7 - 8 am	☐	☐	☐	☐	☐	☐					☐
8 - 9 am	☐	☐	☐	☐	☐	☐					☐
9 - 10 am	☐	☐	☐	☐	☐	☐					☐
10 - 11 am	☐	☐	☐	☐	☐	☐					☐
11 - 12 am	☐	☐	☐	☐	☐	☐					☐
**Afternoon**											
12 - 1 pm	☐	☐	☐	☐	☐	☐					☐
1 - 2 pm	☐	☐	☐	☐	☐	☐					☐
2 - 3 pm	☐	☐	☐	☐	☐	☐					☐
3 - 4 pm	☐	☐	☐	☐	☐	☐					☐
4 - 5 pm	☐	☐	☐	☐	☐	☐					☐
5 - 6 pm	☐	☐	☐	☐	☐	☐					☐
6 - 7 pm	☐	☐	☐	☐	☐	☐					☐

For peer review only

For peer review only - http://bmjopen.bmj.com/site/about/guidelines.xhtml

DAY 6

DATE _ _ / _ _ / _ _

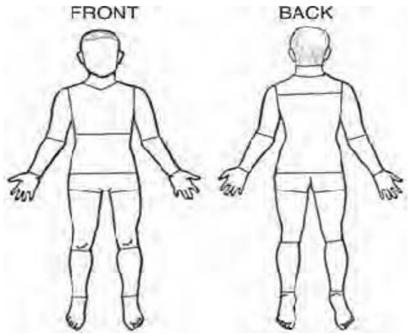

Did you apply sunscreen today?

☐ Yes ☐ No

If yes, shade the diagram on the left to show the parts of the body that you applied sunscreen to today

	TIME OUTDOORS (Cross the box which best represents the amount of time you spent outdoors during each one hour interval shown below)					DID YOU APPLY SUNSCREEN	INSERT NUMBER DENOTING TYPE OF CLOTHING WORN (You must write one number in each box)				
	0 minutes	Below 15 minutes	15-30 minutes	30-45 minutes	45-60 minutes	Cross as many as applicable	Upper body	Lower body	Head wear	Footwear	Gloves (cross all that apply)
**Morning**											
5 - 6 am	☐	☐	☐	☐	☐	☐					☐
6 - 7 am	☐	☐	☐	☐	☐	☐					☐
7 - 8 am	☐	☐	☐	☐	☐	☐					☐
8 - 9 am	☐	☐	☐	☐	☐	☐					☐
9 - 10 am	☐	☐	☐	☐	☐	☐					☐
10 - 11 am	☐	☐	☐	☐	☐	☐					☐
11 - 12 am	☐	☐	☐	☐	☐	☐					☐
**Afternoon**											
12 - 1 pm	☐	☐	☐	☐	☐	☐					☐
1 - 2 pm	☐	☐	☐	☐	☐	☐					☐
2 - 3 pm	☐	☐	☐	☐	☐	☐					☐
3 - 4 pm	☐	☐	☐	☐	☐	☐					☐
4 - 5 pm	☐	☐	☐	☐	☐	☐					☐
5 - 6 pm	☐	☐	☐	☐	☐	☐					☐
6 - 7 pm	☐	☐	☐	☐	☐	☐					☐

For peer review

For peer review only - http://bmjopen.bmj.com/site/about/guidelines.xhtml

DAY 7

DATE _ _ / _ _ / _ _

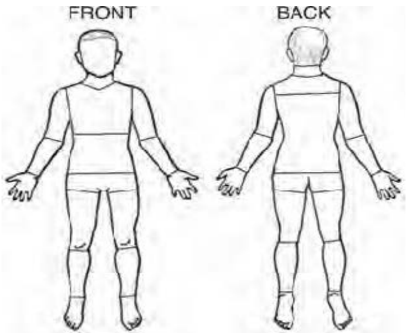

Did you apply sunscreen today?

☐ Yes ☐ No

If yes, shade the diagram on the left to show the parts of the body that you applied sunscreen to today

	TIME OUTDOORS (Cross the box which best represents the amount of time you spent outdoors during each one hour interval shown below)					DID YOU APPLY SUNSCREEN	INSERT NUMBER DENOTING TYPE OF CLOTHING WORN (You must write one number in each box)				
	0 minutes	Below 15 minutes	15-30 minutes	30-45 minutes	45-60 minutes	Cross as many as applicable	Upper body	Lower body	Head wear	Footwear	Gloves (cross all that apply)
**Morning**											
5 - 6 am	☐	☐	☐	☐	☐	☐					☐
6 - 7 am	☐	☐	☐	☐	☐	☐					☐
7 - 8 am	☐	☐	☐	☐	☐	☐					☐
8 - 9 am	☐	☐	☐	☐	☐	☐					☐
9 - 10 am	☐	☐	☐	☐	☐	☐					☐
10 - 11 am	☐	☐	☐	☐	☐	☐					☐
11 - 12 am	☐	☐	☐	☐	☐	☐					☐
**Afternoon**											
12 - 1 pm	☐	☐	☐	☐	☐	☐					☐
1 - 2 pm	☐	☐	☐	☐	☐	☐					☐
2 - 3 pm	☐	☐	☐	☐	☐	☐					☐
3 - 4 pm	☐	☐	☐	☐	☐	☐					☐
4 - 5 pm	☐	☐	☐	☐	☐	☐					☐
5 - 6 pm	☐	☐	☐	☐	☐	☐					☐
6 - 7 pm	☐	☐	☐	☐	☐	☐					☐

Thank you very much for answering the questions and completing the diary. Please be assured that this information will be kept confidential.

For peer review only - http://bmjopen.bmj.com/site/about/guidelines.xhtml

BMJ Open

PREDICT CP: Study Protocol of implementation of comprehensive surveillance to Predict outcomes for school aged children with Cerebral Palsy

Journal:	*BMJ Open*
Manuscript ID	bmjopen-2016-014950.R1
Article Type:	Protocol
Date Submitted by the Author:	11-Apr-2017
Complete List of Authors:	Boyd, Roslyn; The University of Queensland, Queensland Cerebral Palsy and Rehabilitation Research Centre; The University of Queensland, Queensland Children's Medical Research Institute<br>Davies, Peter; The University of Queensland, Children's Nutrition Research Centre<br>Ziviani, Jenny; Children's Health Queensland Hospital and Health Service, Allied Health Research; The University of Queensland, School of Health and Rehabilitation Science<br>Trost, Stewart; Queensland University of Technology, Institute of Health and Biomedical Innovation, Centre for Children's Health Research<br>Barber, Lee; The University of Queensland, Queensland Cerebral Palsy and Rehabilitation Research Centre, School of Medicine, Faculty of Medicine and Biomedical Sciences<br>Ware, Robert; The University of Queensland, School of Population Health<br>Rose, Stephen; CSIRO Australian e-Health Research Centre, Neuroimaging<br>Whittingham, Koa; The University of Queensland, Queensland Cerebral Palsy and Rehabilitation Research Centre<br>Sakzewski, Leanne; The University of Queensland, Queensland Cerebral Palsy and Rehabilitation Research Centre ; Children's Health Queensland, Children's Allied Health Research<br>Bell, Kristie; The University of Queensland, Queensland Cerebral Palsy and Rehabilitation Research Centre; The University of Queensland, Queensland Children's Medical Research Institute<br>Carty, Chris; Griffith Univ, Biomechanics<br>Obst, Steven ; The University of Queensland, Queensland Cerebral Palsy and Rehabilitation Research Centre<br>Benfer, Katherine; The University of Queensland, Queensland Cerebral Palsy and Rehabilitation Research Centre<br>Reedman, Sarah; The University of Queensland, School of Medicine, Queensland Cerebral Palsy and Rehabilitation Research Centre<br>Edwards, Priya; The University of Queensland, Queensland Cerebral Palsy and Rehabilitation Research Centre; Children's Health Queensland Hospital and Health Service, Queensland Paediatric Rehabilitation Service<br>Kentish, Megan; Children's Health Queensland Hospital and Health Service, Queensland Paediatric Rehabilitation Service<br>Copeland, Lisa; Children's Health Queensland Hospital and Health Service, Queensland Paediatric Rehabilitation Service<br>Weir, Kelly ; Gold Coast University Hospital, Speech Pathology<br>Davenport, Camilla; The University of Queensland, Queensland Cerebral Palsy and Rehabilitation Research Centre

	Brooks, Denise ; The University of Queensland, Queensland Cerebral Palsy and Rehabilitation Research Centre<br>Coulthard, Alan; The University of Queensland, University of Queensland, Faculty of Medicine<br>Pelekanos, Rebecca; The University of Queensland, Queensland Cerebral Palsy and Rehabilitation Research Centre<br>Guzzetta, Andrea; Stella Maris Institute, Neurology<br>Fiori, Simona; Stella Maris Institute, Neurology<br>Wynter, Meredith; Children's Health Queensland Hospital and Health Service, Queensland Paediatric Rehabilitation Service<br>Finn, Christine; Children's Health Queensland Hospital and Health Service, Queensland Paediatric Rehabilitation Service; The University of Queensland, Queensland Cerebral Palsy and Rehabilitation Research Centre<br>Burgess, Andrea; The University of Queensland, Queensland Cerebral Palsy and Rehabilitation Research Centre<br>Morris, Kym; The University of Queensland, Queensland Cerebral Palsy and Rehabilitation Research Centre<br>Walsh, John; Mater Medical Research Institute, Orthoapedics; Children's Health Queensland Hospital and Health Service, Department of Orthopaedics<br>Lloyd, Owen; Children's Health Queensland Hospital and Health Service, Queensland Paediatric Rehabilitation Service<br>Whitty, Jennifer; University of East Anglia Norwich Medical School, School of Pharmacy<br>Scuffham, Paul; Griffith University, Griffith Health Institute and School of Medicine

**Primary Subject Heading**:	Paediatrics
Secondary Subject Heading:	Neurology, Paediatrics, Rehabilitation medicine
Keywords:	Cerebral Palsy, Longitudinal cohort, brain structure and function, NUTRITION & DIETETICS, manual ability, communication

SCHOLARONE™
Manuscripts

Article type: Study Protocol

Title: PREDICT CP: Study Protocol of implementation of comprehensive surveillance to Predict outcomes for school aged children with Cerebral Palsy

Author List:

Roslyn Boyd,[1,4] Peter SW Davies,[2] Jenny Ziviani,[3, 4] Stewart Trost,[5] Lee Barber,[1] Robert Ware,[6] Stephen Rose,[14] Koa Whittingham,[1] Leanne Sakzewski, [1,4] Kristie Bell,[1,4], Christopher Carty,[9] Steven Obst,[1] Katherine Benfer,[1] Sarah Reedman,[1] Priya Edwards,[4] Megan Kentish,[4] Lisa Copeland,[4] Kelly Weir,[1, 10,16] Camilla Davenport,[1,2] Denise Brookes,[2] Alan Coulthard,[11] Rebecca Pelekanos,[12] Andrea Guzzetta,[15] Simona Fiori,[15]Meredith Wynter,[4] Christine Finn,[1] Andrea Burgess,[1] Kym Morris,[1] John Walsh,[9,13] Owen Lloyd,[4] Jennifer A. Whitty,[8] Paul A Scuffham. [16]

Author Affiliations:

[1]Queensland Cerebral Palsy and Rehabilitation Research Centre (QCPRRC), Child Health Research Centre, The University of Queensland, Centre for Children's Health Research, Faculty Medicine, The University of Queensland, Brisbane.

[2]Children's Nutrition Research Centre (CNRC), Child Health Research Centre, Centre for Children's Health Research, Faculty of Medicine, The University of Queensland, Brisbane.

[3]School of Health and Rehabilitation Sciences, The University of Queensland.

[4]Queensland Paediatric Rehabilitation Service, Children's Health Queensland, Lady Cilento Children's Hospital, Brisbane, QLD.

[5]Institute of Health and Biomedical Innovation, Queensland University of Technology

[6]UQ Child Health Research Centre, The University of Queensland

[7]CSIRO Mathematical and Information Sciences Biomedical Imaging Group, Australian e-Health Research Centre.

[8] Norwich Medical School, University of East Anglia, Norwich UK and School of Pharmacy, The University of Queensland

[9]Queensland Children's Motion Analysis Service, , Children's Health Queensland

[10]Clinical Governance, Education and Research, Gold Coast Health Service.

For peer review only - http://bmjopen.bmj.com/site/about/guidelines.xhtml

[11]Medical Imaging, Royal Brisbane and Women's Hospital, Faculty of Medicine, The University of Queensland

[12]University of Queensland Centre for Clinical Research

[13]Department of Paediatric Orthopaedics, the Mater Health Services, Brisbane.

[14]Medical Imaging, Diagnostic and Interventional Neuroradiology Department at the Royal Brisbane and Women's Hospital, Queensland Health, Brisbane.

[15] Department of Developmental Neuroscience, Instituto Di Ricovero E Cura A Carattere Scientifico (IRCCS), Stella Maris, University of Pisa, Italy;

[16] Menzies Health Institute Queensland, Griffith University;

[17] Queensland Centre for Intellectual and Developmental Disability, The University of Queensland;

Corresponding Author:

Professor Roslyn Boyd, PhD, Scientific Director,

Queensland Cerebral Palsy and Rehabilitation Research Centre,
Level 6, Children's Health Research Centre, The University of Queensland,
62 Graham Street, South Brisbane. QLD 4101, Australia.
E-Mail: r.boyd@uq.edu.au; Phone: +61 (07) 3069 7372 Fax: +61 (07) 3069 7109

Total Word Count: 11,480

Key words (5 max): Cerebral Palsy, Longitudinal cohort, Motor development, Brain Structure and Function, Communication, Gross Motor Function, Manual Ability,

For peer review only

ABSTRACT

Objectives

Cerebral Palsy (CP) remains the world's most common childhood physical disability with total annual costs of care and lost wellbeing of AU$3.87b. The Predict-CP study will investigate the influence of brain structure, body composition, dietary intake, oropharyngeal function, habitual physical activity, musculoskeletal development (hip status, bone health), and muscle performance on motor attainment, cognition, executive function, communication, participation, quality of life and related health resource use costs. The Predict-CP cohort provides further follow-up at 8-11 years of two overlapping preschool-age cohorts examined from 2-5 years (NHMRC465128 motor and brain development; NHMRC569605 growth, nutrition and physical activity).

Methods and Analyses

This population based cohort study undertakes state-wide surveillance of 245 children with CP born in Queensland (birth years 2006-2009). Children will be classified for Gross Motor Function Classification System (GMFCS); Manual Ability Classification System (MACS), Communication Function Classification System (CFCS) and Eating and Drinking Ability Classification System (EDACS). Outcomes include gross motor function, musculoskeletal development (hip displacement, spasticity, muscle contracture), upper limb function, communication difficulties, oropharyngeal dysphagia, dietary intake and body composition, participation, parent and child reported quality of life and medical and allied health resource use. These detailed phenotypical data will be compared to brain macro and micro structure using 3 Tesla Magnetic Resonance Imaging (3T MRI). Relationships between brain lesion severity and outcomes will be analysed using multilevel mixed-effects models.

Ethics and Dissemination

The PREDICT CP protocol is a prospectively registered and ethically accepted study protocol. The study combines data at 2-5 then 8-11 years of direct clinical assessment to enable prediction of outcomes and health care needs essential for tailoring interventions (e.g. rehabilitation, orthopaedic surgery and nutritional supplements) and the projected health care utilisation.

ANZTR Trial Registration Number: ACTRN 12616001488493

Strengths and limitations of this study:

- The Predict prospective cohort study provides comprehensive phenotypical data on a representative cohort of children with CP.
- The longitudinal follow-up of this cohort (at 2-5 years and now cross sectional at 8-11 years) will enable development of prediction models of outcome.

For peer review only - http://bmjopen.bmj.com/site/about/guidelines.xhtml

For peer review only

- Brain structure (macro and micro structure at 3.0 tesla) will be compared comprehensive motor, cognitive and communication outcomes at school age.
- A limitation is that only brain macrostructure at 1.5T has been captured from early clinical brain MRI scans as part of clinical practice.

For peer review only

For peer review only - http://bmjopen.bmj.com/site/about/guidelines.xhtml

BACKGROUND

Cerebral Palsy (CP) is a disorder of movement and posture secondary to an insult to the developing brain [1]. The insult is static and permanent and may be the consequence of different factors, including both genetic and environmental causes. Although the insult is static, the consequent symptoms are variable and may change over time [2]. The disability increases with age and ageing occurs earlier [3]. Children may have a range of comorbidities [4], which are likely to impact outcomes and costs of care [5]. Based on CP registers, a recent systematic review identified that in children diagnosed with CP at 5 years: 3 in 4 were in pain; 1 in 2 had an intellectual disability; 1 in 3 could not walk; 1 in 3 had hip displacement; 1 in 4 could not talk; 1 in 4 had epilepsy; 1 in 4 had a behaviour disorder; 1 in 4 had bladder control problems; 1 in 5 had a sleep disorder; 1 in 5 dribbled; 1 in 10 were blind; 1 in 15 were tube fed; and 1 in 25 were deaf [6]. It is known that peak motor attainment in CP is reached at 8-9 years and tends to plateau before a decline in adolescence [3]. Secondary musculoskeletal disorders involving muscle, tendons, bones and joints are common as a result of spasticity, muscle weakness and immobility. Cerebral Palsy has substantial lifelong effects on daily function, societal participation and quality of life (QOL) for children and their families. There is a paucity of data on the relationship between physical outcomes and school attainment [7]. Better prediction of outcomes is important for families and health care providers [8].

In Australia, CP remains the most common physical disability in children with ≈ 700 infants born each year that will be later diagnosed with CP [9]. The overall costs to society of persons with CP was AU$1.47b per year (0.14% of GDP), with an average annual cost of AU$43,431 per individual [10]. When taking into account the value of lost wellbeing (disability and premature death) the total costs were AU$3.87b per year or $115,000 per person. Cerebral Palsy has a lifetime impact at a total cost of over AU$2M per person [10]. More recently, in a preschool-aged cohort (CP-Child, National Health and Medical Research Council NHMRC 465128) we have determined a strong relationship between severity of Gross Motor Function Classification System (GMFCS) levels I-V and a stepwise increase in incremental costs of care [5].

The ability to better predict outcomes has the potential to guide intervention to reduce adverse outcomes (hip dislocation, poor growth, under or over nutrition, respiratory health complications from oropharyngeal dysphagia, pain, reduced participation in the community and under attainment at school). Development of prediction models based on early brain structure and function can inform health and social care provision (for example, via the National Disability Insurance Scheme, N.D.I.S.) and provide best practice comprehensive surveillance to allow implementation of timely and effective interventions to achieve optimal outcomes.

Understanding the relationship between specific brain Magnetic Resonance Imaging (MRI) appearance and outcome measures such as motor function is critically important [11]. Such data may prove invaluable in providing accurate prognostic counselling at the time of diagnosis, as well as potentially guiding the most appropriate treatments tailored to each individual's pattern of CP and type and severity of the brain lesion on imaging [12]. A focus of the majority of epidemiological research is the prevention of CP, which requires

For peer review only - http://bmjopen.bmj.com/site/about/guidelines.xhtml

clinical outcomes to be correlated with the presumed timing and aetiology of lesions in the developing brain [11]. Pathological insults in the developing brain cause abnormalities or lesions, which may be detected by brain MRI, and the patterns of these lesions depend on the stage and/or presumed timing of the injury during brain development [13]. Using this principle, a qualitative system of classification is established whereby lesions can be identified as brain maldevelopments (occurring in the 1st and 2nd trimesters) [11], periventricular white matter lesions (occurring early in the 3rd trimester and in preterm infants), or grey matter lesions (occurring late in the 3rd trimester and at term) [11]. A systematic review found studies with enough MRI data for subjects to be classified into these presumed lesion timing groups, and in the majority of studies this lesion timing classification was able to be linked to at least one measure of motor outcome [11.] There were however limited data on brain lesion severity, brain microstructure and quantitative comprehensive outcomes [11].

In the Australian CP-child study entire birth years of Victorian and Queensland born children with CP across the full spectrum of gross motor abilities were prospectively followed to determine the relationship between the rate and limit of motor development (gross and fine motor function) as related to the nature of the brain lesion [12, 14]. Representative population-based data has been reported on i) early development and prediction of hip outcomes [15], ii) the relationship between brain structure and motor development[12], and iii) social function [16] and communication [17] with cost and health resource use data across the spectrum of functional severity [5]. The cross sectional domains of school readiness (mobility, self-care, social function, communication) were reported at school entry [16, 18].

In CP there is a likely relationship between the severity of the early brain injury on structural MRI (nature, extent, presumed timing), early motor status at 3 years, and later outcomes at 8-11 years (motor attainment, musculoskeletal performance, hip displacement). In Sweden, Norway, and Scotland a population wide surveillance program (CP-UP) has been implemented for up to 10 years [19]. Since implementation in Southern Sweden no child with CP has had a dislocated hip [19], musculoskeletal contractures have been reduced [20] and nutrition and bone health are monitored [21, 22]. National hip surveillance best practice guidelines have been developed and implemented in Australia [23], and in Queensland population wide hip surveillance has been implemented [24].

The Predict CP study will undertake further comprehensive follow-up of four birth years of children with CP born in Queensland to capture longitudinal data on growth and physical outcomes (motor capacity, muscle and bone health, physical activity, feeding and oropharyngeal function, nutrition), cognition (executive function, educational attainment, communication), and participation, quality of life, pain and relate these to costs of health care utilisation. The quantitative evaluation of early brain structure on MRI and functional status at 2 years will be compared to these comprehensive outcomes at 8-11 years to build prediction models of CP. Development and implementation of prediction models of outcomes are essential for tailoring interventions (rehabilitation, medical management, orthopaedic surgery, nutritional supplements) and in understanding the likely costs of health care.

For peer review only - http://bmjopen.bmj.com/site/about/guidelines.xhtml

Growth, nutrition and physical activity are important determinants of health outcomes in children with CP. Knowledge of levels and patterns of Habitual Physical Activity (HPA) for children with CP are important as they have increased risk of inactivity (sedentary behaviour) related illness [9, 25]. In addition, poor nutrition and growth may have a secondary impact on body composition, bone health and brain maturation, as well as participation and Health Related Quality of Life (HRQOL) in later childhood. In our overlapping CP-child study of growth, nutrition and physical activity (NHMRC 569605) [26] we have determined at preschool-age the i) energy requirements, body composition, dietary intake [27-29], ii) validation of HPA cut-points [30], iii) validation of a modified 3-day weighed food record for the assessment of energy intake [29] and determined iv) oropharyngeal dysphagia (OPD) across the spectrum of functional severity [31, 32].

Our early data on nutritional status [29] used gold standard measures (doubly labelled water) to determine the energy requirements of preschool-aged children with CP compared to age matched children with typical development (TD) [28]. Children who were GMFCS III-V had energy requirements 18% lower than ambulant children and 31% lower than children with TD [28], with no differences between ambulant children with CP and children with TD. In addition, energy intake was related to fat free mass index in both children with CP and children with TD [29]. Associations were identified between OPD, energy intake and nutritional status after GMFCS level has been taken into account. At preschool age, OPD was originally reported in 85% of our cohort, with a significantly greater proportion of OPD with each increase in GMFCS level [31]. Following further testing of OPD psychometrics, with the inclusion of a typically developing reference sample, modified cut points were developed resulting in a revised estimate of 56% [33]. Children on full oral intakes that required modification (texture or additional energy and protein) were most at risk of poor growth and nutritional status [31].

Habitual Physical Activity accelerometer cut-points have been determined for sedentary and active behaviour in toddlers with CP [34], demonstrating that HPA levels are highly variable within GMFCS levels particularly GMFCS I-II [35].The musculoskeletal development of children with CP has focused on how spasticity interferes with normal muscle growth, and contributes to reduced joint range of motion, increased joint stiffness, and muscle weakness [36]. These factors lead to fixed contractures of the muscle-tendon unit and skeletal deformity that may require orthopaedic surgery [37]. These secondary alterations progress with age [38] and contribute to reduced gait speed, increased joint pain and falling, culminating in reduced HPA [39]. Muscle adaptations begin early[37] and compared to children with TD vary in the following ways: i) muscle volume is reduced [36, 40, 41]; ii) muscle fascicles are stiffer when passively stretched [40]; iii) muscle fascicles cannot stretch to lengths more favourable for force production [42]; and iv) the Achilles tendon is longer [42, 43]. This effectively means the ability of muscle to generate force is reduced in children with CP. In ambulant children (GMFCS I-III) the calf muscle (gastrocnemius/soleus) has a major role in forward propulsion during walking/running [44] and structural/functional adaptations are a cause of gait limitations [45]. Characteristics of muscle structural/functional adaptations also vary according to uni/bilateral motor distribution [46]. Lower limb treatments (casting, intramuscular Botulinum toxin A injections) aim to manage these adaptations in the preschool years however multi-level orthopaedic surgery is often required at functional attainment (8-11

For peer review only - http://bmjopen.bmj.com/site/about/guidelines.xhtml

years) according to a child's gait profile [47, 48]. A gait profile of ambulant children (GMFCS I-III) combined with muscle properties would provide important information for surgical decision making and prediction of functional outcome. Examination and surveillance of the relationship between muscle structure/function and gait profile to functional capacity/performance, physical activity, bone health, nutritional status and health care costs would provide vital information for structuring management plans into later childhood.

The broad aim of the CP-Child studies is to implement population based comprehensive surveillance of children with CP from early diagnosis (at 2-3 years) based on brain structure and function (early gross and fine motor, growth, nutrition, HPA, musculoskeletal development) to predict comprehensive outcomes at school age (8-11 years), a time of definitive motor maturation, walking ability, need for orthopaedic intervention and educational attainment. In this extended follow-up of two previous overlapping prospective population based CP cohorts (followed from 18-24 months corrected age (c.a.) to 5 years) across the full spectrum of functional severity (NHMRC 465128 [14]; NHMRC 569605 [26]) we will re-examine the relationship to severity of brain structure at 8-11 years on diffusion MRI (dMRI in a 3.0T MRI scanner). At 8-11 years health care utilisation is likely to be different to preschool-age so that associations between health resource use and a beneficial health/social outcome will be re-evaluated.

Aims and hypotheses

The Predict-CP study will undertake comprehensive state-wide surveillance (in Queensland) of four birth years of a representative population based cohort of children with CP. The relationship between brain structure on growth and physical outcomes (motor capacity, muscle and bone health, physical activity, oropharyngeal function, nutrition), cognition (executive function, educational attainment, communication), and participation (Habitual Physical Activity, Quality of Life, pain and sleep), will be related to educational attainment and health resource use costs.

Hypotheses

1. The location, extent of the brain lesion(s) on semi-quantitative MRI (at 2 years) and early motor capacity and performance (2-3 years) will predict severity of motor capacity Gross Motor Function Measure (GMFM-66) and performance (6 minute walk test, 6MWT), Pediatric Evaluation of Disability Inventory Computer Adaptive Test (PEDI-CAT) at 8-11 years.
2. The rate and limit of gross motor and fine motor development (GMFM-66, Assisting Hand Assessment (AHA), Both Hands Assessment (BoHA)), at 8-11 years will be influenced by the severity of musculoskeletal deformity (i.e. slower development will correlate with increased spasticity/contracture, poor muscle function, marked hip displacement, pain, reduced sleep, reduced manual ability).
3. Cognition, executive function, communication and educational attainment will be related to brain lesion severity (location, extent of the brain lesions) on semi-quantitative MRI but not gross and fine motor capacity (GMFCS, MACS) at 8-11 years.
4. Nutritional status (under/overweight), OPD, body composition (fat free mass and fat mass via Dual energy X-ray Absorptiometry (DXA)), Habitual Physical Activity (HPA), growth velocity and bone

For peer review only - http://bmjopen.bmj.com/site/about/guidelines.xhtml

health will be related to the level of GMFCS attainment and will predict: i) higher health care utilisation and direct medical costs; ii) lower levels of participation in school, leisure and community, and iii) poorer HRQOL.

Study Significance

For children with CP this unique project will:

1. Quantify the impact of functional severity on medical resource use to inform service provision planning at school age (a period of intensive medical and orthopaedic treatments). From earlier sampling of these cohorts (NHMRC 465128/569605) we have detailed information on the content, dose and compliance, adverse events, medical, surgical and allied health resource use (interventions, medications, equipment) and consequences of outcome (from the age of 1.5 till 5 years). By study completion we will have life-time data on all interventions from age 1.5 'till 8-11 years, with regular assessments of their functional status/ outcomes allowing predictive modelling of outcomes for children with CP.
2. This project will provide school-age follow-up of this comprehensively studied cohort enabling: i) prediction of outcome (brain structure and multiple outcomes); ii) prognostication on functional, cognitive, communication for school attainment; iii) risk factors for musculoskeletal problems (i.e. hip, spine deformity and need for surgery); and iv) health outcomes due to sedentary behaviour, body composition, dietary intake and OPD.
3. Highlight the contribution of poor dietary intake, low levels of HPA, and reduced bone health on growth, body composition and fracture risk, taking into account the severity of disability.
4. Define the relationship between HPA levels, motor capacity and muscle performance to predict eventual functional attainment and community performance.

As CP remains the most common childhood physical disability with high lifetime costs, models to predict outcomes and costs of care will inform health provision, social care and tailor data for national funding schemes such as the Australian National Disability Insurance Scheme (NDIS).

METHODS

All children diagnosed with CP, born between 1st January 2006 and 31st December, 2009 in Queensland will be invited to participate. These children have participated in two prospective longitudinal cohort studies between the ages of 1.5 to 5 years and will now be invited to return at 8-11 years. For inclusion criteria Cerebral Palsy is defined as a permanent (but not unchanging) disorder of movement and posture that results from an insult to the developing central nervous system. The characteristic signs are spasticity, movement disorders, muscle weakness, ataxia and rigidity[14].

Exclusion criteria

1. Children with a progressive or neurodegenerative lesion.
2. Children born outside Queensland in the relevant birth years.

Ethics Approvals

There are no known health or safety risks associated with participation in any aspect of the described study. All radiological tests (including AP pelvis, spine as required) and full body and lateral distal femur DXA for

For peer review only - http://bmjopen.bmj.com/site/about/guidelines.xhtml

body composition and bone health have been reviewed for radiation safety. All families will give written informed consent to participate, and they are able to withdraw their child from the study at any time without explanation, without any penalty from staff at Children's Health Queensland, or any effect on their child's care. Data collected in this study will be stored in a coded re-identifiable form (by ID number).

Ascertainment of the cohort

Prospective entry of birth years Queensland (born in 2006, 2007, 2008, 2009) whom were entered at 18 months and followed until school age (5 years) (n=245) in the Australian CP child study will be invited to participate in the Predict-CP follow-up study. State-wide recruitment was established in collaboration with the Queensland Cerebral Palsy Register with data collection at tertiary referral hospitals. In cases where the diagnosis of CP was unclear, or where there is a suggestion of a progressive or degenerative course, further investigations (such as metabolic screening) were requested before a diagnosis of CP was confirmed. Children detected after 18 months of age were entered into the study at the time of diagnosis, offered brain MRI at entry and were followed up with serial motor assessments and other outcomes until confirmation of the diagnosis of CP at 5 years.

The recruited sample born in Queensland (n=245) in the birth years of 2006, 2007, 2008 and 2009 are representative of a population based sample [49]. The sample is classified according to the Gross Motor Function Classification System for 2-18 years (GMFCS), a five level classification system of children's functional gross motor severity [39]. It is based on self-initiated movements, anti-gravity postures and motor skills expected in a typical five year old [50]. Children who are independently ambulant are classified as GMFCS I or II, those requiring an assistive mobility device to walk classified as GMFCS III and those in wheeled mobility as GMFCS IV and V. The recruited sample includes children who are functioning at 5 years of age at Gross Motor Function Classification, GMFCS level I=96 (39%), II=38 (15.6%), III=38 (15.6%), IV=35 (13.6%), V=38 (15.6%); of whom 146 are male (62%), of spastic motor type 208 (86.6%), and unilateral 78 (31.8%) or bilateral 165 (68%) motor distribution (Figure 1). Children will be assessed during their eighth to eleventh birth year at the Centre for Children's Health Research in Brisbane. Co-morbidities and need for medical management will be screened.

Procedures

Children and families who have participated in previous research projects (NHMRC 569605 and NHMRC 465128) and were born in Queensland will be approached to participate in the current study. After providing informed consent the child and their caregiver will be invited to attend the Children's Health Research Centre and the Lady Cilento Children's Hospital, a tertiary referral centre for a 1-2 day visit. All recent medical, surgical and neurological visits that have occurred since their last visit will be screened (from their medical records and by parent report) to confirm any changes in diagnosis of CP, differential diagnosis by neurological assessment (by a Paediatrician, Child Neurologist or Paediatric Rehabilitation Specialist). Experienced Allied Health researchers will perform all motor, upper limb, language and cognitive assessments at the visit. Physiotherapists will check range of motion, clinical measures of spasticity, then

For peer review only - http://bmjopen.bmj.com/site/about/guidelines.xhtml

rate GMFCS, gait pattern, MACS and measure pelvic and spine radiographs where indicated according to standardized protocols [23].

Classification Measures

All children with CP at all levels of ability (GMFCS I-V) at 8-11 years will be classified for:

Functional severity

The GMFCS has internationally established validity, reliability and stability for the classification and prediction of motor function of children with CP aged 2-12 years [50-52]. It has an acceptable inter-rater and intra-rater (test-retest) reliability (generalisability coefficients 0.93 and 0.68, respectively) [51]. Two physiotherapists, trained in the use of the GMFCS, will independently observe and classify children in one of five functional categories [50].
Classifications of gross motor abilities change with age and therefore separate descriptions are used for different age bands. In the current study, the 6-12 year descriptions from the extended and revised GMFCS (GMFCS-ER) will be used [53]. The GMFCS has been correlated with a number of motor scales, as well as CP motor type and distribution [54].

Motor type and distribution

Motor type will be classified as spastic, dystonic, ataxic, hypotonic, choreoathetosis, mixed CP or unclassifiable according to Surveillance of Cerebral Palsy in Europe (SPCE) guidelines [55]. Distribution will be classified by number of limbs impaired, uni- and bi-lateral distribution (hemiplegia, diplegia, triplegia, quadriplegia) by at least two independent raters. The Dyskinesia Impairment Scale [56] will be undertaken for those participants with a motor-type (primary or secondary) diagnosis of dystonia and/or choreoathotosis. This is an important assessment to measure the motor capacity and function of children with these particular motor-types [57].

Functional performance

The Functional Mobility Scale (FMS) [58] at 5m (home), 50m (school) and 500m (community) [58] will be used to evaluate functional performance. This is a valid and reliable measure of a child's usual walking ability at three distances (5m, 50m and 500m), representing their home, school and wider community [59].

Gait pattern

Gait patterns will be classified according to the Rodda & Graham's classifications [60], which has demonstrated validity and reliability[61]. Gait patterns for bilateral ambulant CP will be classified as either: i) True Equinus, ii) Jump Knee, iii) Apparent Equinus; or iv) Crouch Gait. For children with unilateral CP, gait patterns will be classified according to Winters, Gage and Hicks [48]. This classification considers the sagittal plane joint movements: i) type I - foot drop during swing phase (Apparent Equinus); ii) type II - persistent ankle plantarflexion (True Equinus); iii) type III - maintained plantar flexion through gait cycle plus limited

For peer review only - http://bmjopen.bmj.com/site/about/guidelines.xhtml

knee flexion-extension; and iv) type IV - similar to III, plus reduced hip flexion-extension. Winter's classification has good inter-rater reliability using written reports (weighted kappa, wκ=0.76) and videos (wκ=0.63) [61-63].

Upper Limb Function

Upper limb function is classified using the Manual Ability Classification System (MACS) [64]. The MACS is an international system to classify hand function based on the child's typical performance when handling objects in daily activities. The MACS is a five level classification of how well children with CP use their hands to handle objects in day-to-day activities [64]. This classification system was developed for children aged from 4-18 years, and has good reliability for use in children as young as two years[64]. The MACS has reported construct validity, and excellent inter-rater reliability (ICC=0.97 between therapists and 0.96 between therapists and parents) for children with CP [65]. Children will be classified on the MACS by an occupational therapist in discussion with the child's carer.

Communication Function

Communication function will be classified on three distinct but overlapping systems:

i. Communication Function Classification System (CFCS) classifies children's performance in sending and receiving communicative messages using their typical communication means (considering all communication methods including Augmentative and Alternative Communication). It has been validated in children with CP aged 2-18 years. Reliability between professionals was moderate (κ=0.66), professional-parent fair (κ=0.49), and test-retest strong (κ=0.82) [66].
ii. Functional Communication Classification System (FCCS) classifies children's performance only in sending communicative messages, and also considers their typical communication (including all communication methods including Augmentative and Alternative Communication). It has excellent inter-rater reliability between professionals (kappa=0.94) and parent-professional (κ=0.59) [67].
iii. The Viking Speech Scale (VSS) will be used to classify children's speech production [68]. The VSS is a four level classification system, which can be used to classify speech intelligibility for strangers and unfamiliar conversation partners of children with CP aged 4 years and above. It has strong content validity, and moderate-substantial inter-rater reliability between pairings of speech pathologists, health care professionals and parents (kappa=0.58-0.81) [68].

Eating and drinking function

The Eating and Drinking Ability Classification System (EDACS) classifies the eating and drinking abilities of children with CP aged 3 years and above. Classification is I-V and describes children's safety and efficiency predominately focusing on food and fluid textures [69]. The EDACS has strong inter-rater reliability between professionals (ICC=0.93), but fair reliability between parings of professionals and parents (ICC=0.45) [69].

For peer review only - http://bmjopen.bmj.com/site/about/guidelines.xhtml

PRIMARY OUTCOMES

This prospective longitudinal study follow-up has two primary outcomes (Hypothesis 1):

1. Gross motor function will be evaluated using the GMFM-66;
2. Brain lesion severity will be assessed using a structured scoring proforma (Fiori scale).

All additional measures are secondary outcome measures (Hypotheses 2-4).

Body Structure and Function Measures

Brain structure on magnetic resonance imaging (MRI)

The American Academy of Neurology practice parameter has concluded that brain MRI should be part of the diagnosis of CP [70]. Early MRI at 0-3 years will be classified according to the nature and presumed timing of the lesion [11] and analysed for brain lesion severity on the semi-quantitative scale of Fiori[8]. Aetiology of CP will be evaluated using MRI (location, nature and structure of the brain lesion)[11]. The brain lesion will be classified by 3 main criteria:

A. the *anatomical features* of the lesion:
   a. localisation by tissue (e.g. cortical, white matter, deep grey matter etc.)
   b. localisation by region (e.g. lobes involved, laterality etc.)
   c. extent of lesion (e.g. generalised, hemispheric, lobar etc.)
B. the presumed *aetiology* of the lesion: i) genetic; ii) ischemic; iii) infective and iv) other.
C. the presumed *timing* of the insult that caused the lesion:
   a. Prenatal by trimester or by stage of brain development;
   b. Perinatal;
   c. Postnatal.

All MRIs will be classified by a neurologist (SF) together with a neuroradiologist (AC) using a standardised method of image evaluation and classification. Following these evaluations, consensus will be reached regarding the above three criteria. Based on preliminary data it is estimated that >60 percent of children currently receiving a diagnosis of CP will have had early brain MRI as part of their clinical work-up. All children (n=245) will be offered a repeat brain MRI at 8-11 years at 3T. The majority will have their imaging performed and reported through the Herston Imaging Research Facility, on a Siemens 3.0T MR scanner. The current minimum imaging protocol for patients with suspected CP consists of axial fast spin echo and coronal fast spin echo sequences and 3D inversion prepared fast spoiled GRASS sequence. 3D acquisitions are reformatted in axial, coronal and sagittal planes, with additional oblique and curved reformatting. Age specific protocols are used to maximize the ability to detect cortical and white matter abnormalities at different stages of myelination. All neuroimaging will be reviewed by a neurologist (SF, AG) and a neuroradiologist (AC) familiar with the features of lesions that result in CP. This approach is consistent with a Clinical Practice guideline suggesting that all patients with the label of CP have high quality MR imaging

For peer review only - http://bmjopen.bmj.com/site/about/guidelines.xhtml

on at least one occasion[70]. MRI scans will be performed predominantly awake, without anaesthesia and after informed consent. Preparation for the MRI will be offered to families in the form of a training DVD explaining the scanner experience and practice in a "mock scanner" (0.0Tesla) will be offered, where required.

Brain lesion severity will be assessed using a structured scoring proforma [8] based on the CH2 template [71], a highly detailed single-subject T1 template in Montreal Neurological Institute MNI space, which is the international standard for brain mapping (International Consortium of Brain Mapping - ICBM). Lesions will be transcribed onto the proforma and the following measures obtained: number of i) anatomical lobes involved, ii) number of slices on the template that were affected and iii) size and distribution of the lesion measured by a global lesion score and lesion sub-scores. The score (maximum of 40) is based on: i) anatomical lobes involved; ii) number of affected slices; and iii) size and distribution of the lesion. The number of lobes and slices affected will be the average of summed right and left hemispheres. To calculate total lesion score, each frontal, parietal, temporal and occipital lobe will be first considered in three sections: periventricular, middle and subcortical matter. Each section will be scored as 0.5 if less than 50% of area was involved; or 1, for greater than 50% involvement, with a maximum lobar score of 3. Lobar scores for each hemisphere will be summed, with a maximum hemispherical score of 12 possible. The total lesion score will be the sum of right and left hemispherical scores (maximum score of 24). A 1-point score (involved/not involved) will also be attributed to 16 anatomical structures including the corpus callosum, the cerebellum and the main subcortical structures. The final maximum score of the scale will be, thus, a maximum of 40 (24+16) [72] . The Fiori scale method has strong inter rater and intra-rater reliability [72] and strong construct validity based on dMRI and functional severity in children with unilateral CP [73].

At 8-11 years structural MRI (sMRI) and functional MRI (fMRI) guided diffusion-weighted MRI (dMRI) scans suitable for connectivity analyses will be undertaken on the 3T scanner at Herston Imaging Research Facility (or Lady Cilento Children's Hospital at 3T for children requiring general anaesthesia ≈ 5%). Diffusion-weighted MRI (dMRI) for white matter fibre tracking and whole brain connectomes will be acquired using our published protocol [74]. Structural MRI (sMRI) images will be acquired using an MPRAGE sequence at an isotropic resolution of 1 mm. dMRI data will be pre-processed to reduce image artefacts [75], and the fibre orientation distribution estimated using constrained spherical deconvolution[76]. Probabilistic tractography will be conducted using MRtrix software and connectivity matrices generated using previously described methods [74]. Quantitative diffusivity indices Fractional Anisotrophy (FA) and Mean Diffusivity (MD) will be encoded within the connectome to assess reorganisation[74]. Network based statistics (NBS) [77] will be performed between FA and MD connectomes to identify significant cortical networks associated with neural reorganization. A second analysis will investigate brain maturation by comparing serial sMRI data acquired around 2 years with scans in the same children at 8-11 years to develop a predictive model of brain structure and functional outcome using spatiotemporal analysis of the longitudinal imaging data [77].

Clinical history and examination

For peer review only - http://bmjopen.bmj.com/site/about/guidelines.xhtml

Clinical history will be reviewed (Appendix 1: QLD CP Child Physicians Checklist) to determine:

a. Presence or absence of comorbidities including vision impairment, hearing difficulties, epilepsy;
b. Feeding issues including presence or absence of gastrostomy tube and failure to thrive;
c. Respiratory difficulties including episodes of pneumonia and aspiration.

A comprehensive musculoskeletal examination will be performed by a physiotherapist to record data relating to joint range of movement, leg length difference, bony anomalies, motor type and lower limb muscle spasticity and contracture [78-83].

Anthropometry

Anthropometric measures will be collected as described in detail in our published growth, nutrition and physical activity protocol [26], including the following:

a. Body mass to the nearest 100 grams using chair scales (Seca Ltd).
b. Height to the last completed millimetre with a stadiometer, or, length using a supine measuring board. Where a direct measure of height or length cannot be obtained, height will be estimated from knee height or upper arm length using published validated techniques and formulas [84].
c. Body mass index will be calculated as mass (kg) divided by height (m) squared.
d. Growth and growth velocity (*Z*-scores of measured or predicted height).

Weight and body mass index Z-scores will be calculated for age and sex according to Centres for Disease Control and Prevention (U.S. CDC) 2000 growth data [85] .

Gross motor function

Gross motor function will be evaluated using the GMFM-66 & GMFM-88 [86] by experienced research physiotherapists. The GMFM-88 assesses a child's motor abilities in lying to rolling, sitting, crawling to kneeling, standing, walking, running and jumping. The GMFM-66 is comprised of a subset of the 88 items identified (through Rasch analysis) as contributing to the measure of gross motor function in children with cerebral palsy. The GMFM-66 will be used to provide an overall measure of gross motor function and the GMFM-88 provides domain scores to explore specific motor skills [86].

Upper limb performance

Children with unilateral CP whom are manual ability MACS I-III will be assessed on the School kids Assisting Hand Assessment (AHA), a Rasch measure of effectiveness of impaired hand in bimanual activities. Test-retest reliability is high (ICC 0.98) and there is predictive validity of future assisting hand use[87]. The Both Hands Assessment (BoHA) will be utilised for children with bilateral CP whom are manual ability MACS I-IV. The BoHA test content was developed by researchers in Norway and Sweden through modification of the Assisting Hand Assessment (AHA) test items and by generation of new items [88]. Associations between BoHA measures and MACS levels show strong correlation (Spearman's rho: 0.74).

For peer review only - http://bmjopen.bmj.com/site/about/guidelines.xhtml

The person separation ratios (4.36 and 5.19) and the person reliability (0.95 and 0.96) for the subscales indicate that the children's hand function can be separated into 6 and 7 ability levels [88]. The BoHA is the first observation based assessment of effective use of the hands in bimanual activities for children with bilateral CP.

Hand dominance will be assessed using the Edinburgh Handedness Inventory laterality quotient [89]. The Edinburgh Handedness Inventory questionnaire consists of 10 items regarding hand preference (right or left) in performing a number of everyday tasks requiring one (writing, drawing, throwing and using scissors) or two hands (e.g. using a broom or opening a box). The laterality quotient is calculated using the following formula: laterality quotient = (right hand – left hand/ (right hand + left hand)*100). The Edinburgh Handedness Inventory has been included to objectively determine upper limb dominance. This classification system consists of a table that requires the participant to indicate which hand they use to perform a selection of everyday tasks.

Stereognosis relates to a participant's ability to perceive and recognise objects by using only tactile information [90, 91] and will be assessed on the impaired and unimpaired limbs, using the approach described by Sakzewski et al [91]. Participants will be required to identify objects placed in their hand, without any visual cues. A total of nine objects are placed in the hand one at a time. Three familiar objects (teaspoon, key, peg) and six similar matched objects (safety pin and paperclip; pen and pencil; coin and button) will be used. With vision occluded, participants will be presented with each item. If a participant is unable to grasp, manipulate or release an object the occupational therapist will assist the participant and move the object for them within their hand. A corresponding set of items will be used to allow participants to identify the object in order to minimise any errors due to incorrect naming of the object. Scores range on a scale from 0-9, where participants scoring below 9, will be considered to have impaired stereognosis [90, 91].

Radiological measures of hip displacement and spine

Hip surveillance, including anterior-posterior (AP) pelvis x-ray, is recommended for all Australian children with CP to facilitate early detection and treatment of severe or progressive hip displacement [19, 92, 93]. The migration percentage (MP) is widely accepted as the gold standard measure in hip surveillance [78, 94], measuring femoral head displacement [95]. Other measures include the acetabular index (AI), assessing acetabular dysplasia [96], the Hilgreneiner's Epiphysseal Angle (HEA) [96] and the femoral neck-shaft angle (NSA) [95, 97]. The HEA [96] is a radiographic measure describing the proximal femoral epiphysis and has been previously applied to assessment of coxa valga [98, 99], but may offer prognostic information for hips at risk in cerebral palsy. The HEA represents the acute angle between a line drawn parallel to and through the proximal femoral epiphysis and Hilgenreiner's line [81, 82]. Physiotherapists will perform a clinical examination of spinal alignment and mobility to screen for evidence of a potential scoliosis or kyphosis. Where indicated an AP spine radiograph (for scoliosis) or lateral (for kyphosis) will be performed. Spines where scoliosis is evident will be measured according to the Cobb angle [100].

For peer review only - http://bmjopen.bmj.com/site/about/guidelines.xhtml

Body composition and bone health:

Body composition measures and bone parameters will be acquired using a Lunar Prodigy DXA (GE Medical Systems, LUNAR, Madison, WI, USA). Body composition measures include: fat mass (FM, g) and fat free mass (FFM, g). Bone parameters include: areal bone mineral density (aBMD, g/cm^2) and bone mineral content (BMC, g) for all total body, bilateral proximal and lateral distal femur sites. The lateral distal femur, is a common site of fracture[101, 102], with the technique previously described [102], and measurements are reproducible in children with CP [103, 104]. The analysis involves creating three regions of interest, each containing different proportions of trabecular and cortical bone with results for each ROI, therefore, treated independently [101, 102]. Additionally, the proximal and distal femoral sites will be used to calculate bone mineral apparent density (BMAD, g/cm^3), derived from the projected bone area (cm^2) to provide an approximation of volumetric BMD [105]. All scans in this research are a 'one off" occurrence, with the total radiation dose for these five DXA scans being <15 μSv[106]. This is equivalent to approximately 1-2 days natural background radiation exposure, and only equivalent to 3% of the dose constraint limit for children as research volunteers, up to the age of 18 years[106]. The total estimated time for all DXA scans is 30 minutes, performed at UQ Children's Nutrition Research Centre.

Fracture rate

Fractures will be diagnosed radiologically. Parents will report by telephone within 24 hours of fracture occurrence and will bring X-ray films and details of management to their study visit. Vertebral fracture will be diagnosed on lateral X-rays of the thoracic and lumbar spine when indicated. Children whom are GMFCS III-V will undergo thoracic and lumbar spine (AP/lateral) at 8-11 years if there are clinical signs of fracture and/or scoliosis/kyphosis. Radiographs will be minimised to reduce the radiation exposure.

Sexual Maturation

Legal guardians of participants will be provided with standardised Tanner stage puberty diagrams, and parents will be asked to evaluate the child's current pubertal stage [107]. Parental pubertal assessment will be reviewed by a physician for precocious puberty. In cases of precocious puberty, a left hand/ wrist X-ray will be conducted to determine the bone age and relative skeletal maturity of children. The bone age will be used to determine if the CP condition is interfering with the proper growth and bone development of the child.

Pain

Children will complete the Pediatric Pain Questionnaire (PPQ) with adult help if required[108]. The PPQ asks children to report their pain now (severity, type, and location), as well as the severity of the worst pain they had in the previous week. The PPQ's visual analogue scale (VAS) for pain rating provides a valid and stable measure of pain intensity in children and adolescents with chronic musculosksletal pain [109].

For peer review only - http://bmjopen.bmj.com/site/about/guidelines.xhtml

Three-dimensional gait analysis and in vivo muscle mechanics

A full 3D gait analysis, including synchronised measurement of muscle activation using electromyography (EMG) and calf muscle mechanics using 2D ultrasound, will be performed for all children functioning at GMFCS I-III. Participants will walk unaided and barefoot at a self-selected speed over a level walkway (10m in length) with four force platforms embedded in the laboratory floor in the centre of the walkway. Reflective markers will be attached to the trunk, pelvis, and lower limbs according to the modified 'Plug in Gait' marker set, with additional clusters of three markers on each thigh and shank segment, and a marker on the 5th metatarsal head [110]. Marker trajectories will be recorded at 100 Hz using an 8-camera, 3D motion capture system (Vicon Motion Systems, Oxford, UK) and ground reaction force data will acquired at 1 kHz using four 510mm × 465mm force platforms (AMTI, Watertown, MA, USA) arranged in series. Lower limb muscle activations of the rectus femoris (RF), medial hamstrings (MH), medial gastrocnemius (MG), lateral gastrocnemius (LG), soleus (SOL) and tibialis anterior (TA) will be recorded for both legs at 1 KHz using a wireless surface EMG system (Aurion ZeroWire, Milan, Italy). Raw EMG signals will be high-pass filtered (Butterworth, zero-lag, 4th order, 30 Hz) to remove movement artefact, full wave rectified and low passed filtered (Butterworth, zero-lag, 4th order, 6Hz), and interpolated to 101 points per cycle. Non-negative matrix factorisation will be applied to extract muscle synergies [111], which represent neuromuscular control during gait. Whole body 3D gait kinematics, joint moments at ankle, knee and hip joints and musculotendinous lengths for MG, LG, SOL, RF and MH will be computed across at least five trials using OpenSim [112] and normalised to length in standing. The Gait Profile Score [113] will be calculated as an index of overall gait pathology. A digital output signal from the ultrasound system was used to synchronize acquisition of all 3D marker, force plate and EMG data.

Two-dimensional B-mode ultrasound will be used to examine MG and SOL muscle function during walking by attaching a flat ultrasound transducer (LV7.5/65/64D, Telemed Echo Blaster 64 EXT-1T, Vilnius, Lithuania) to the surface of the skin above the MG muscle and recording muscle fascicle length and pennation angle changes, as described previously [114]. Muscle fascicle behaviour during walking will be analysed using a semi-automatic process which has been shown to be highly repeatable (Coefficient of multiple correlation 0.88) [115]. The average of five complete strides will be used in the analysis for each participant to ensure the overall reliability of muscle fascicle length data [116]. Freehand three dimensional (3D) ultrasound will be used to measure muscle size and structure of the lower leg muscles: MG, LG, SOL and TA [117]. This method of 3D ultrasound is valid (within 1.3%) and reliable (ICC>0.99) for measuring gastrocnemius muscle volume and length in vivo [117] . Calf muscle physiological cross sectional area will be measured as the ratio of muscle volume muscle fascicle length, corrected for fascicle pennation angle.

Activity Limitations

The following measures of activity limitations for functional capacity will be performed for ambulant children (GMFCS I-III) at 8-11 years (≈ n=172).

For peer review only - http://bmjopen.bmj.com/site/about/guidelines.xhtml

Six Minute Walk Test (6MWT)

This simple, sub-maximal test measures the distance walked over six minutes, providing information about endurance during functional activities [118]. The 6MWT has excellent test-retest reliability (ICC=0.98) in CP [119]. Percentile curves have been created on 1,445 children with TD aged 7-16 years [120]. The test will be performed according to guidelines of the American Thoracic Society on a 10m course [121].

Muscle power sprint test (MPST)

The MPST provides an estimate of anaerobic power [122]. The MPST requires participants to complete six 15m runs as fast as possible with 10s rest between each lap. Power output is calculated as the product of body mass and distance, divided by time [122]. The MPST has been validated against the Wingate Anaerobic cycling test [123], and has excellent test-retest reliability (ICC 0.98) in children with CP [122].

10m fast walk test (10m FWT)

The 10mFWT is a test of maximal walking speed over a distance considered the minimum for functional ambulation. The 10mFWT has moderate test-retest reliability for in children with CP (ICC 0.81) [124].

Lower limb functional strength

Thirty second repetition maximum (rep_{max}) of functional strength exercises (including sit-to-stand, lateral step-ups and half-kneel to stand) will be tested according to published recommendations [125]. Functional strength tests demonstrate acceptable inter-tester reliability (ICC= >0.91; coefficient of variation (CV) 12.1-22.7%) in children with CP [125]. For each lower limb functional strength exercise participants will be given verbal and visual instructions as well as two practice repetitions prior to testing. The exercises were assessed in the following order: sit-to-stand, lateral step-up dominant leg, lateral step-up non-dominant leg, half-kneel to stand dominant and half-kneel to stand non-dominant. Participants will be given verbal encouragement throughout. Participants will be given 180 seconds rests between exercises. If a participant cannot complete an exercise whilst performing the practice attempts, they will be assigned a score of 0 and will not proceed to testing.

Habitual Physical Activity (HPA)

Triaxial accelerometers (ActiGraph GT3X+, Pensacola, FL, USA) will be used to evaluate the frequency, intensity, and duration of physical activity [126]. ActiGraph accelerometers have evidence of validity and inter-instrument reliability in children with TD compared to heart rate monitoring, direct observation, indirect calorimetry, whole-room calorimetry and doubly labelled water [126]. The ActiGraph has been validated for measurement of physical activity intensity in adolescents with CP using oxygen uptake as the criterion measure[127 128]. ActiGraphs will be fitted during assessment and worn during waking hours for 7 days[126]. Participants' caregivers will complete a 7 day physical activity monitor log to record wear and non-wear times (see Appendix 2). Stored data will be uploaded to an excel macro to determine daily wear time,

For peer review only - http://bmjopen.bmj.com/site/about/guidelines.xhtml

average counts per min, daily time spent in sedentary, light, moderate, and vigorous activity. Counts will be classified using established cut-points for children with CP[128].

Blood samples for Growth Hormone and Vitamin D:

Blood will be collected and tested for hormones and other markers of required for optimal growth, bone and metabolic health. Specifically, these tests are liver function, kidney function, full blood count (FBC), Insulin like Growth Factor-1 (IGF1), thyroid hormone, parathyroid hormone, vitamin D3, calcium, phosphate, and iron studies. As described above, these parameters of growth, bone health and body composition are often altered in children with CP and are related to gross motor function classification, body composition, growth velocity and nutritional status. Blood tests will be optional and consent will be obtained from the parent and assent from the child where possible. Blood samples will be collected by qualified phlebotomists, who are familiar with collecting blood from paediatric subjects using their standard procedures. Where preferred, samples will be collected under general anaesthesia, if a patient is undergoing an unrelated and non-emergency surgical procedure (e.g. orthopaedic surgery, Botulinum Toxin A injections, MRI under anaesthesia). Parents of participants will be advised if these results fall outside the relevant reference ranges in relation to age, gender and pubertal status. Parents will provide informed consent for information to be provided to their treating clinician who will take responsibility for ongoing care and follow up.

Dietary Intake

Dietary energy intake will be recorded using a 3-day weighed food record as validated[29] using our published methods[26]. Food records will be analysed using FoodWorks™. Mean energy intake will be expressed as megajoules per day and as a percentage of age and gender specific recommendations [129].

Vitamin D intake

A vitamin D in a food frequency questionnaire will be completed by parents to determine the habitual intake of Vitamin D containing foods of the participants. The questionnaire consists of a table that requires parent's to tick a frequency box and record the brand of a simple list of foods[130].

Sun Exposure

A Sun Exposure Diary will measure daily sun exposure in the participants to measure ultraviolet radiation (UV) exposure for vitamin D adequacy. Each day, participants will record the amount of time spent in the sun during each 1-hour interval (0, <15, 15–<30, 30–<45, or 45–60 minutes) between 5:00 AM and 7:00 PM. Clothing cover (based on a clothing cover guide provided with the dairy) and use of sunscreen (frequency and application site) using established methodology will also be recorded [131] (Appendix 3). It is proposed that the sun exposure diary will be done at the same time as the Physical activity monitor record to lessen the burden on the participants. Sun exposure diaries will be done within two weeks of serum Vitamin D levels being collected, to allow for meaningful interruption of sun exposure and Vitamin D levels.

For peer review only - http://bmjopen.bmj.com/site/about/guidelines.xhtml

Oropharyngeal dysphagia

Oropharyngeal dysphagia (feeding and swallowing difficulties) will be evaluated during a digital video recorded snack of 20 minutes. Children will be presented with three standardised boluses of five textures; puree, semi-solid, chewable, tough chewable and fluid. The following measures will be used to rate the mealtime:

1. The Dysphagia Disorders Survey (DDS) – Part 2 consists of a series of binary judgments on eight ingestion functions across the oral preparatory, oral, pharyngeal and gastro-oesophageal phases (maximum raw score of 22). The DDS has good reliability [132, 133] and convergent validity [132-136].

2. The Schedule for Oral Motor Assessment (SOMA) consists of seven oral motor challenge categories corresponding to four food textures and three fluid utensils. The SOMA has been validated on 127 young infants; 58 comparison children with typical oral skills, 56 with non-organic failure to thrive (aged 8-24 months), and 13 children with CP and overt feeding difficulties (aged up to 42 months) [137]. It has strong inter-rater reliability (κ=1.0 in 68% of fluid category items and 58% of food category items) and test-retest reliability between boluses (κ=1.0 in 84% of items) [137].

3. Observations of 16 clinical signs suggestive of pharyngeal phase impairment (e.g. cough, gurgly phonation, wet respiration) will be rated pre- and post-mealtime by a trained researcher, and rated according to each food/ fluid texture from video by a speech pathologist [138-140].

4. The Thomas-Stonell and Greenberg scale will be used pre and post mealtime to rate saliva loss [141]. This consists of two observational ordinal scales (1-5), based on severity and frequency of loss.

5. The Cerebral Palsy Child Feeding Questionnaire (CPFQ), used in the CP Child Study [142] will gather information on the child's typical mealtime performance based on parent report, which will supplement the data obtained from the participant's clinical feeding assessment.

6. The Feeding/Swallowing Impact Scale (FS-IS) will address questions of carer Quality of Life and how to incorporate it into economic evaluation. The Feeding/Swallowing Impact Scale (FS-IS) is a validated tool to measure the impact of caring for a child with dysphagia and concerns on caregiver Quality of Life[143]. It is an 18 item, parent questionnaire divided into three subsections: i) daily activities; ii) worry; and iii) feeding difficulties. The tool was validated on the caregivers of 164 children (median age 14 months, mean: 32 ± 44 months) with varying co-morbidities including prematurity (<37 weeks) in 66 (40%) children, 144 (88%) were medically complex with conditions in more than one diagnostic based category; and 77 (47%) of children had feeding tubes[143].

Communication

All children will have language assessed using the core language subtests of the Clinical Evaluation of Language Fundamentals screener (CELF-4) [144], in addition to the Peabody Picture Vocabulary Test (PPVT).

For peer review only

The CELF-4 is a criterion referenced assessment of language skills in children aged 5-21 years, with Australian norms available. Children who are non-verbal will only complete the receptive subtests of the CELF-4 and the PPVT. Children unable to participate in standardised assessment (e.g. due to significant cognitive, visual or motor limitations) will have language classified using the Triple C, a parent reported observational assessment. Communication performance will be indicated by parents on a communication questionnaire developed for this study (Appendix 4), which includes information on Augmentative and Alternative Communication system type, use, frequency and access [144].

Speech production will be assessed using the Verbal Motor Production assessment for Children (VMPAC). The VMPAC [145] is a diagnostic tool for the systematic assessment of neuromotor integrity of the motor speech system validated in 1,434 children aged 3-12 years[146]. The following subtests of the VMPAC will be administered:

1. Oromotor Production in Word Sequences and Sentences (6 items): Items consists of 3- and 4-word sequences and 5-word sentences. These items are designed to evaluate the child's ability to sequence oromotor movements across different plane within a linguistic context.

2. Oromotor Production in Connected Speech and Language (5 items): This subtest will assess the child's motor control (e.g. motor precision) as it varies in the context of higher level language formulation.

3. Oromotor Production in Automatic Verbal Sequences (1 item): This subtest allows evaluation of speech characteristics including pitch, resonance, vocal quality, loudness, prosody, intonation, and rate during production of an automatic speech task (e.g. counting to ten, saying the alphabet).

4. Oromotor Production in Word Sequences and Sentences (6 items): Items consists of 3- and 4-word sequences and 5-word sentences. These items are designed to evaluate the child's ability to sequence oromotor movements across different plane within a linguistic context. Oromotor Production in Connected Speech and Language (5 items): This subtest will assess the child's motor control (e.g. motor precision) as it varies in the context of higher level language formulation.

5. Oromotor Production in Automatic Verbal Sequences (1 item): This subtest allows evaluation of speech characteristics including pitch, resonance, vocal quality, loudness, prosody, intonation, and rate during production of an automatic speech task (e.g. counting to ten, saying the alphabet).

Pediatric Evaluation of Disability Inventory Computer Adapted Test (PEDI-CAT)

Performance of self-care will be evaluated using the parent-reported Pediatric Evaluation of Disability Inventory Computer Adapted Test (PEDI-CAT) for the domains of self-care, mobility and social functioning using scaled scores (Rasch), which have good validity and reliability[147]. The PEDI-CAT was developed on the basis of the original PEDI [148]. Scaled scores (possible range 20-80) for each domain provide an indication of the child's performance along a continuum of item difficulty and are most suitable for research [147]. Scaled

For peer review only - http://bmjopen.bmj.com/site/about/guidelines.xhtml

For peer review only

scores give more precise results in the extreme ranges than normative standard scores [147]. Scaled scores are recommended to track functional progress in children who are substantially delayed [147].

Participation and Environmental Measures

Participation and Environment Measure for Children and Youth (PEM-CY)

The PEM-CY is a parent-reported instrument that examines participation and environment across three settings: i) home; ii) school; and iii) community [149]. There are 10 items in the home section, five in the school section and 10 in the community setting. For each item, the parent is asked to identify how frequently (over the past four months) the child has participated (eight options: daily to never); how involved the child typically is while participating (five point scale: very involved to minimally involved); and whether the parent would like to see the child's participation in this type of activity change (no or yes, with five options for the type of change desired). For each setting, the parent is then asked to report on whether certain features of the environment make it easier or harder for the child to participate. The PEM-CY has reported moderate to good internal consistency (0.59 and above) and test-retest reliability (0.58 and above) in a population of children (aged 5 to 17 years) with and without disabilities residing in the United States of America and Canada (n=576) [149]. The PEM-CY will be collected using either a paper or online questionnaire format to gain an understanding of the participation of children and adolescents and the impact of environmental barriers and facilitators.

Strengths and Difficulties Questionnaire (SDQ)

The Strengths and Difficulties Questionnaire (SDQ) [150, 151] is a 33 item parent-rated questionnaire that is used to assess parents' perceptions of pro-social and difficult behaviours in their child or child adjustment. Parents respond to 25 questions about their child's behaviour in the last six-months using a three-point Likert scale (i.e., "0" = not true to "2" = certainly true). These 25 questions combine to create five sub-scales of: frequency of emotional symptoms; conduct problems; inattention/hyperactivity; peer problems; and prosocial behaviour (e.g. "considerate of other people's feelings"). A total score for each scale (0-10) and overall total difficulties score (0-40) will be calculated, with higher scores indicating more distress on all scales except prosocial behaviour. Scores of 17 or above for the total difficulties scale will be used a clinical cut-off point. Scores from the five sub-scales and the overall difficulties scale will be used as a measure of the child's psychological functioning. The overall total difficulties score has been demonstrated to have moderate to high internal consistency (Cronbachs α = 0.73-0.82) and test-retest reliability (r = 0.77 – 0.85) [152].

School Attainment

The Australian Early Development Index (AEDI) is a population measure of development at school entry, assessing school readiness [153]. The National Assessment Program - Literacy and Numeracy (NAPLAN), measures literacy/numeracy achievement and individual reports will be obtained from families as a measure

For peer review only - http://bmjopen.bmj.com/site/about/guidelines.xhtml

of early school achievement [154]. Where applicable, consent will be obtained from parents to the release of NAPLAN result by the Queensland Curriculum and Assessment Authority (QCAA).

Cognition

Raven's Coloured Progressive Matrices (RCPM)

The RCPM is an assessment of nonverbal intelligence for children aged 5-11 years with intellectual delay or physical disability. It consists of 36 items (15-30 minutes) of increasing difficulty in which the child has to complete a pattern. RCPM has validity with CP and Australian norms [155, 156]. In 618 Australian children (6-11years) the RCPM had good internal consistency (0.76-0.88) and split-half reliability (0.81-0.90) [156].

Behaviour Rating Inventory of Executive Function (BRIEF)

The BRIEF is a parent completed 86 item measure of executive function in their child's everyday life, yielding two scores: i) the behavioural regulation index; and ii) the metacognition index to form a global executive composite score. Both scores can be converted into *T* scores with ≥65 indicative of dysfunction [157]. It has good convergent and divergent validity with Child Behaviour Checklist and the Behaviour Assessment System for Children [158], high internal consistency, (Cronbach's α 0.80-0.98) for parent form [158, 159].

Attention and Executive Functioning

Connors 3 parent short form (Conners 3^{TM})

The Conners 3^{TM} [160] is a thorough assessment of ADHD and its most common co-morbid problems and disorders in children and adolescents ages 6-18 years old. The Conners 3^{TM} will be completed by the participant's parents or guardian and consists of 110 statements and takes approximately 20 minutes to complete. Parents or guardians must rate each statement using a four-point scale ranging from '0 – Not true at all (never, seldom)' to '3 – Very much true (very often, very frequently)'. The Conners 3^{TM} measures the seven key areas of inattention, learning problems, aggression, family relations, hyperactivity/impulsivity, executive functioning, and peer relations. Raw scores are converted into T scores based on a large representative normative sample based on United States of America consensus data. In addition, the Conners 3^{TM} calculates T scores for symptom scales including ADHD Hyperactive/Impulsive, ADHD Combined, Oppositional Defiant Disorder, ADHD Inattentive, and Conduct Disorder. Both internal consistency coefficients (α = 0.83-0.94) and test-retest reliability (r = 0.52-0.94) are good for the Conners 3^{TM} Parent version total sample age range [160].

The Autism Spectrum Quotient-Child (AQ10-Child)

The AQ10-child is a 10 item parent-report autism screening measure for children aged 4-11 years [161]. It was developed from the Quantitative Checklist for Autism in Toddlers (Q-CHAT). Parents respond to the 10 items on a 4 point Likert Scale from Definitely Agree to Definitely Disagree. Items are summed and the

For peer review only - http://bmjopen.bmj.com/site/about/guidelines.xhtml

scale has a cut off of 6. It has high internal consistency (Cronbach's α =0.90), sensitivity (0.95) and specificity (0.89) [161].

Sleep Disturbances Scale for Children (SDSC)

Sleep disorders are up to four times more common in children with cerebral palsy compared to the general population and are linked to both physical (total body involvement, severe visual impairment) and environmental factors (single-parent household, bed-sharing). The most commonly reported disorders include difficulty initiating and maintaining sleep, sleep-wake transitions and sleep breathing disorders [162]. The SDSC is a 27 item parent-report questionnaire that assesses sleep disturbance in children within the past six months [163]. Each item is responded to on a 5 item Likert scale with higher values representing greater clinical severity. Items are summed to produce a total score and six sub-scores representing different facets of sleep disturbance: disorders of initiating and maintaining sleep, sleep breathing disorders, disorders of arousal, sleep-wake transition disorders, disorders of excessive somnolence and sleep hyperhidrosis. It has good internal consistency (Cronbach's α = 0.71-0.79), test-retest reliability (r=0.71) and discriminative validity in distinguishing between clinical and community samples [163].

Quality of Life Measures

Condition-specific Quality of Life measures

The Cerebral Palsy Quality of Life-child (CPQOL-child) assesses wellbeing across multiple domains using parent-report (4-12 years) and child self-report from 9 years [164-166]. Psychometrics are excellent with Cronbach's α 0.74-0.92 parent-proxy report and 0.80-0.90 child self-report. Test re-test is adequate (ICC 0.76-0.89) and moderately correlated with health (r=0.30-0.51)[164-166].

Generic Quality of life

The Child Health Utility-9D (CHU-9D) is a generic instrument for children aged 7-11 years for which there is an algorithm to give a single preference-based utility index for health states (giving a single generic preference-based indicator of each individual's health state), making the data amenable for economic evaluations for interventions [167-170]. The EuroQOL five dimensional descriptive system (EQ-5D-5L) is a generic instrument designed to describe and value health based on 5 dimensions: mobility, self-care, usual activities, pain/discomfort and anxiety/depression [171]. The EQ-5D-5L will be completed by the parent/care-giver, about themselves and scored using the Australian algorithm [172, 173] . This is relevant (alongside the CES) to address questions such as carers QOL and how to incorporate it into economic evaluation.

Demographic Questionnaire

Parents will be required to complete questionnaires related to various aspects of their child's development/progress including participation, feeding, health related quality of life, sleep, fracture history, pain, executive functioning in everyday life, clinical co morbidities including epilepsy, psychological

For peer review only - http://bmjopen.bmj.com/site/about/guidelines.xhtml

functioning, treatments and interventions the child has received since the completion of the CP Child projects (Appendix 5). The socioeconomic status of Australian families will be classified into tertiles using scores on the Socioeconomic Indexes for Areas Index of Relative Disadvantage [174].

Parent Questionnaires

Carer Experience Scale (CES)

The CES will be completed by primary care givers at their study visit. This validated measure of care-related QoL has six domains (activities, support, assistance, fulfilment, control and relationship with the care recipient) and takes approximately 3 minutes to complete [175]. The CES is scored from an algorithm derived from preferences of the general population and can be used to value carer outcomes in economic evaluation using index values [176].

The McMaster Family Assessment Device General Functioning Scale (FAD)

Caring for a child with a disability can have an impact on the health and functioning of the care-giver and family unit [177]. The FAD is a 12-item measure of general family functioning [178]. Each item is rated on a four point Likert scale from strongly agree to strongly disagree and the items are summed to create the total score. It has good internal consistency (Cronbach's $\alpha = 0.92$), construct validity and reliability [178-180].

Monitoring of resource use and direct costs of treatments

To determine the relationship between motor prognosis and healthcare costs, resource use and direct costs of treatment will be monitored using the Health Resource use (HRU) questionnaire [14] (Appendix 6). Associations between costs (dependent variable) and all other outcome variables (independent variables) including those related to growth, body composition and HPA will be assessed, with adjustment for confounders such as brain lesion severity. Health-related resource use data collected includes therapy frequency and duration (traditional/alternate), hospital admissions, GP and medical specialist visits, medications (e.g. Botulinum Toxin-A), and equipment (e.g. orthoses). Data will be collected via questionnaire [14], supplemented by consented access to individual hospital, Medical Benefits Scheme (MBS) and Pharmaceutical Benefits Scheme (PBS) records. Standard cost sources (MBS, PBS) will be used to apply unit costs to resources. Statistical approaches, which consider the likely skewed distribution of cost data (such as generalised linear models, with extensions to allow for correlations in the data across observations from multiple time points from each individual as described below), and diagnostic related group costs for admissions to hospital)will be employed. As this cohort is embedded in a state-wide clinical service, we have consistency of interventions based on best practice guidelines for Botulinum toxin A (BoNT-A), bone health and hip surveillance generalisable across Australia. All children are offered best practice treatment across the state-wide service.

For peer review only - http://bmjopen.bmj.com/site/about/guidelines.xhtml

Data Analysis Plan

A comprehensive database has been established for all data collection, including clinical measures, MRI scoring and questionnaires so that it is entered prospectively at the time of each assessment. Summary reports are automatically generated from the database to report back to families and treating clinicians after each visit. Bio statistics methods proposed in this study include analysis of binary outcomes in longitudinal studies using weighted estimating equations (e.g. presence of comorbidities); multilevel mixed-effects models of longitudinal binary outcomes (e.g. GMFCS levels), and generalised estimating equations for ordinal data.

Sample size

We assume 95% of the total sample of 245 children will consent to Predict-CP program (we have 98% retention in NHMRC 465128, 569605). For H^1, assuming between-GMFCS group and within-child variability in GMFM-66 are similar to Rosenbaum[181], we will be able to detect significant between-group differences in GMFM-66 scores at 8-11 years according to the primary predictor variable of initial GMFCS group (2-5 years) with >80% power and alpha=0.05 for each pairwise comparison. For comparisons between MRI classification at 2 years and GMFM-66 at 8-11 years, then based on GMFCS and MRI data from NHMRC 465128 (where GMFCS I=30%, II-V=16% each and WMI=43%), assuming that GMFM-66 at 8-11 years has a SD of 7 units within each GMFCS group[181], we will be able to detect a difference between children with white-matter damage and other MRI pattern types of ≥ 4.6 GMFM-66 points in GMFCS I and ≥ 6.7 points within each GMFCS level II–V, with 80% power, alpha=0.05. For secondary outcomes we have differing power depending on sample characteristics e.g. two groups, evenly distributed, we will detect differences of ≥0.36 SD with α=0.05, 80% power.

Statistical considerations

Summary statistics will be described using either mean (standard deviation) or median (25th-75th percentile) for continuous variables, according to distribution, or as frequency (percentage) for categorical variables. Cross-sectional associations will be assessed using linear regression for interval outcome data, with effect estimates presented as mean difference and 95% confidence interval (95%CI), using logistic regression for binary outcome data, with effect estimates presented as odds ratios and 95%CI, and using Poisson regression for count outcome data, with effect estimates presented as incidence rate ratios and 95%CI. Longitudinal associations will be investigated using analyses that account for the multiple observations per participant. The particular analysis will be determined by data structure, for example hierarchical mixed-effects models or generalised estimating equations. If hierarchical mixed-effects models are used then ‘participant’ will be included in the model as a random effect, to account for the possible non-independence of observations from the same participant. When building multivariable models, first univariable models including all potential variables of interest will be constructed. Variables will be selected for potential inclusion in multivariable models based on univariable significance at the p<0.2 level. Multivariable models will be built in a step-wise manner with redundant variables eliminated using Akaike’s and Schwarz’s Bayesian criteria. In principal, multivariable models will first be constructed for the whole sample, then after stratification by GMFCS level.

For peer review only - http://bmjopen.bmj.com/site/about/guidelines.xhtml

For peer review only

Interactions will be investigated as appropriate. For H^1 we will first undertake univariable analyses of the association between quantitative MRI, motor capacity, performance, and potential confounding variables (e.g. gender) at 2-5 years with motor capacity at 8 years using a mixed-effects model with data grouped by individual participants to account for (up to 3) repeated measures between 2-5 years.

Missing data will be treated on a case-by-case basis depending on the observed pattern of missingness. For example if data is 'missing at random' we will use multiple imputation methods, and if data is 'not missing at random' we will use pattern-mixture models. There will be no global rule to account for multiple comparisons, instead adjustment for multiple comparisons will be made for each separate suite of analyses as appropriate, bearing in mind the type I & II error rates for each suite.   Cost data will be standardised to current values. Cost data are typically skewed and therefore will be tested for normality and transformed using a log, gamma or another appropriate link function for the multivariable analysis

Complex multivariable analyses accounting for attrition, if required, will be conducted by a biostatistician. The precise analyses will be determined by data structure. In principal, hierarchical mixed-effects models will be performed for the whole population then by GMFCS level. First, univariable then multivariable analyses will be undertaken. Variables included as fixed effects in the final multivariate model will be selected based on univariate results if $p<0.2$. Redundant variables will be eliminated from multivariable models using Akaike's and Schwarz's Bayesian criteria. All models will include a random intercept and slope (time effect) for each participant, accounting for the non-independence in repeated measures from the same participant and allowing for heterogeneity between participants. For H^1 we will first undertake univariable analyses of the association between quantitative MRI (primary), motor capacity (primary), performance, and potential confounding variables (e.g. gender) at 2-5 years with motor capacity at 8 years using a mixed-effects model with data grouped by individual participants to account for (up to 3) repeated measures between 2-5 years. Then all variables significant at $P<0.2$ will be included in the prediction model, before being investigated for elimination. Interactions will be investigated as appropriate. Missing data will be treated on a case-by-case basis using MAR (multiple imputation) or NMAR (using pattern-mixture models). Adjustment for multiple comparisons will be made for each separate analyses mindful of type I & II error rates, as is standard practice.

ETHICS AND DISSEMINATION

This study protocol describes the rationale, aims, hypotheses and methods for a large, prospective, longitudinal, population-based study of motor development and brain structure in a representative sample of preschool aged children with Cerebral Palsy, using direct clinical assessment.

Ethics committee approvals were obtained for the Australian CP child study through The Royal Children's Hospital Melbourne Ethics Committee, (HREC/25010 F), Southern Health Human Research Ethics Committee C (05077C), University of Queensland Medical Research Ethics Committee (2007001784), the Children's Health Services District Ethics Committee (HREC/07/QRCH/107), the Mater Health Services Human Research Ethics Committee (1186C), the Queensland Cerebral Palsy Register at the Cerebral Palsy

For peer review only - http://bmjopen.bmj.com/site/about/guidelines.xhtml

League of Queensland (CPLQ 2008/ 09-1010), Gold Coast Health Service District Human Research Ethics Committee (HREC/08/QGC/45), Central Queensland Health Services District Human Research Ethics Committee (HREC/08/QCQ/19), Cairns and Hinterland Health Service District Human research Ethics Committee (HREC/08/QCHHS/521) and the Townsville Health Service District Human Research Ethics Committee (HREC/08/QTHS/33). Subsequent ethics approvals have been obtained for the PREDICT study from the University of Queensland Medical Research Ethics Committee (2007001784) and the Children's Health Services District Ethics Committee (HREC/07/QRCH/107).

The results of this study will be published in peer reviewed journals and presented at relevant international conferences. Outcomes of this prospective cohort study will inform prediction of patient outcomes and likely needs, which will impact clinical practice, the management of children with CP, their carers and the health care system more widely.

AUTHORS' CONTRIBUTIONS

RB is the chief investigator and together with chief investigators PSWD, JZ, ST, LB, RW, SR, KW, JAW, KB and Associate Investigators KB, CC, JW, PE, MK, LC, LS, KW, AC, AG conceptualized, designed and established this research study and successfully obtained study funding for a National Health and Medical Research Council partnership grant. RP, KB, CD, SO, SR, DB, AB, SF and AG were responsible for the selection of particular assessments. JAW conceptualised the inclusion of QoL instruments for children and carers able to support economic evaluation. RB, SR, SF and AG were responsible for conceptualising the brain MRI protocol and analysis. RB is responsible for ethics applications and reporting. RB, KB, LB, CC, PE, LS, LC, KW, DB, KB, CD, SO, SER, CF, AB, KM, OL will be responsible for recruitment and data collection. RB drafted the manuscript with input from all the co-authors. All authors have agreed the final version of the manuscript and were involved in the decision to submit the manuscript. There is no financial support for the authors regarding this manuscript. The external funding agencies (NHMRC) have provided funds for the conduct of the study but will not be involved manuscript preparation, decisions to publish or the interpretation of results arising from the study.

COMPETING INTERESTS

The authors declare they have no competing interests.

FINANCIAL INTERESTS

The authors declare that they have no financial or non-financial competing interests.

FUNDING STATEMENT

This work was supported by the National Health and Medical Research Council of Australia (NHMRC Partnership Project 1077257) with in kind support from Children's Health Queensland, CSIRO and Griffith

For peer review only - http://bmjopen.bmj.com/site/about/guidelines.xhtml

University. The National Health and Medical Research Council has provided the following people support: Research Fellowship (R Boyd); Early Career Fellowship (L Barber, L Sakzewksi, K Whittingham).

List of abbreviations

ADL: Activities of daily living

AHA: Assisting hand assessment

BGBS: Basal ganglia and brainstem score

BM: Brain Malformation

BMC: bone mineral content

aBMD: areal bone mineral density

BMI: Body mass index

BRIEF: Brief rating inventory of executive functioning

CDGM: Cortical and deep grey matter

CFCS: Communication Function Classification System

CHU-9D: Child health utility 9D

CP: Cerebral palsy

CPFQ: Cerebral Palsy Feeding Questionnaire

CSD: Constrained spherical deconvolution

DDS: Dysphagia Disorders Survey

DXA: Dual-energy X-ray absorpitometry

EDACS: Eating and Drinking Ability Classification System

EPI: Echo-planar imaging

ET: Echo time

FC: Functional connectivity

FCCS: Functional Communication Classification System

FOV: Field of view

FSIQ: Full scale intelligence quotient

GEEs: Generalised estimating equations

GMFCS: Gross motor functional classification system

GMFM: Gross motor function measure

GRE: Gradient-recalled-echo

GS: Global score

HPA: Habitual physical activity

HS: Hemispheric score

ICC: Intraclass correlation coefficients

ICF: International classification of functioning, disability and health

IQ: Intelligence quotient

KM: Krägeloh-Mann

For peer review only

LI: Laterality index

MACS: Manual ability classification system

METS: Metabolic equivalents

MDC: Minimum detectable change

MRI: Magnetic resonance imaging

OPD: Oropharyngeal Dysphagia

PEDI: Pediatric evaluation of disability inventory

PEM-CY: Participation and environment measure for children and youth

PPVT: Peabody Picture Vocabulary Test

PWM: Periventricular white matter

QCPRRC: Queensland cerebral palsy and rehabilitation research centre

QOL: Quality of life

Rep_{max}: Repetition maximum

ROM: Range of motion

RT: Repetition time

SDQ: Strengths and difficulties questionnaire

SEM: Standard error of measurement

SOMA: Schedule for Oral Motor Assessment

sMRI: Structural magnetic resonance imaging

TD: Typical development

UL: Upper limb

VMPAC: Verbal Motor Performance Assessment for Children

WHO: World health organisation

3T: 3 tesla

6MWT: Six minute walk test

List of Figures:

Figure 1: Summary of surveillance and outcome measures for PREDICT study

Supplementary Documents:

Appendix 1: Qld Predict CP – Physician's checklist

Appendix 2: Predict CP study - 7 day physical activity monitor log

Appendix 3: PREDICT CP 7- Day Sun Exposure Diary

Appendix 4: Augmentative and Alternative Communication (AAC) Questionnaire

Appendix 5: PREDICT: Demographic questionnaire

Appendix 6: Qld PREDICT CP Study Health Resource Use Form

For peer review only - http://bmjopen.bmj.com/site/about/guidelines.xhtml

REFERENCES

1. Rosenbaum P, Paneth N, Leviton A, Goldstein M, Bax M, Damiano D, et al. A report: the definition and classification of cerebral palsy April 2006. Dev Med Child Neurol. 2007;109:8.
2. Graham HK. Absence of reference to progressive musculoskeletal pathology in definition of cerebral palsy. Dev Med Child Neurol. 2006;48(1):78-9.
3. Hanna SE, Rosenbaum PL, Bartlett DJ, Palisano RJ, Walter SD, Avery L, et al. Stability and decline in gross motor function among children and youth with cerebral palsy aged 2 to 21 years. Dev Med Child Neurol. 2009;51(4):295-302.
4. Australian Cerebral Palsy Register Group. Report of the Australian Cerebral Palsy Register, Birth Years 1993-2006. 2013.
5. Jordan R, David M, Pareezer L, Kentish M, McKinlay L, Ware RS, et al. Relationship between health resource cost and performance outcomes in preschool age children with cerebral palsy; economic analysis. Dev Med Child Neurol. 2013;55(s3):6.
6. Novak I, Hines M, Goldsmith S, Barclay R. Clinical prognostic messages from a systematic review on cerebral palsy. Pediatrics. 2012;130(5):e1285-312.
7. Bodimeade HL, Whittingham K, Lloyd O, Boyd RN. Executive function in children and adolescents with unilateral cerebral palsy. Dev Med Child Neurol. 2013;55(10):926-33.
8. Guzzetta A, Sinclair K, Clarke D, Boyd R. A novel semi-quantitative scale for classification of brain MRI for children with cerebral palsy. Dev Med Child Neurol. 2010;52:74.
9. Australian Bureau of Statistics. Sport and Recreation: A Statistical Overview, Australia. Canberra: Author; 2006.
10. Access Economics. The Economic Impact of Cerebral Palsy in Australia in 2007. 2008.
11. Krägeloh-Mann I, Horber V. The role of magnetic resonance imaging in elucidating the pathogenesis of cerebral palsy: a systematic review. Dev Med Child Neurol. 2007;49(2):144-51.
12. Arnfield E, Guzzetta A, Boyd R. Relationship between brain structure on magnetic resonance imaging and motor outcomes in children with cerebral palsy: a systematic review. Res Dev Disabil. 2013;34(7):2234-50.
13. Krägeloh-Mann I. Imaging of early brain injury and cortical plasticity. Exp Neurol. 2004;190(Suppl 1):84-90.
14. Boyd RN, Jordan R, Pareezer L, Moodie A, Finn C, Luther B, et al. Australian Cerebral Palsy Child Study: protocol of a prospective population based study of motor and brain development of preschool aged children with cerebral palsy. BMC Neurol. 2013;13(1):57.
15. Pym A, Ware R, Jordan R, Boyd R. Early predictors of hip displacement by school age in young children with cerebral palsy. Dev Med Child Neurol. 2012;54(s6):61.
16. Whittingham K, Fahey M, Rawicki B, Boyd R. The relationship between motor abilities and early social development in a preschool cohort of children with cerebral palsy. Res Dev Disabil. 2010;31(6):1346-51.
17. Coleman A, Weir KA, Ware RS, Boyd RN. Relationship Between Communication Skills and Gross Motor Function in Preschool-Aged Children With Cerebral Palsy. Arch Phys Med Rehabil. 2013;94(11):2210-7.
18. Stephenson J, Bauman A, Armstrong T, Smith B, Bellew B. The costs of illness attributable to physical inactivity in Australia. Canberra, Australia: Commonwealth Department of Health and Aged Care and the Australian Sports Commission; 2000.
19. Hagglund G, Andersson S, Duppe H, Lauge-Pedersen H, Nordmark E, Westbom L. Prevention of dislocation of the hip in children with cerebral palsy. The first ten years of a population-based prevention programme. J Bone Joint Surg. 2005;87(1):95-101.
20. Hagglund G, Wagner P. Spasticity of the gastrosoleus muscle is related to the development of reduced passive dorsiflexion of the ankle in children with cerebral palsy. Acta Orthop. 2011;82(6):744-8.
21. Uddenfeldt Wort U, Nordmark E, Wagner P, Duppe H, Westbom L. Fractures in children with cerebral palsy: a total population study. Dev Med Child Neurol. 2013;55(9):821-6.
22. Westbom L, Lundkvist Josenby A, Wagner P, Nordmark E. Growth in children with cerebral palsy during five years after selective dorsal rhizotomy: a practice-based study. BMC Neurol. 2010;10:57.

For peer review only - http://bmjopen.bmj.com/site/about/guidelines.xhtml

23. Wynter M, Gibson N, Kentish M, Love S, Thomason P, Kerr Graham H. The Consensus Statement on Hip Surveillance for Children with Cerebral Palsy: Australian Standards of Care. J Pediatr Rehabil Med. 2011;4(3):183-95.
24. Kentish M, Wynter M, Snape N, Boyd R. Five-year outcome of state-wide hip surveillance of children and adolescents with cerebral palsy. Journal of Pediatric Rehabilation Medicine. 2011;4(3):205-17.
25. Weil E, Wachterman M, McCarthy EP, Davis RB, O'Day B, Iezzoni LI, et al. Obesity among adults with disabling conditions. J Am Med Assoc. 2002;288(10):1265-8.
26. Bell KL, Boyd RN, Tweedy SM, Weir KA, Stevenson RD, Davies PSW. A prospective, longitudinal study of growth, nutrition and sedentary behaviour in young children with cerebral palsy. BMC Public Health. 2010;10:179.
27. Walker JL, Bell KL, Boyd RN, Davies PS. Energy requirements in preschool-age children with cerebral palsy. Am J Clin Nutr. 2012;96(6):1309-15.
28. Walker JL, Bell KL, Boyd RN, Davies PS. Validation of a modified three-day weighed food record for measuring energy intake in preschool-aged children with cerebral palsy. Clin Nutr. 2013;32(3):426-31.
29. Walker JL, Bell KL, Stevenson RD, Weir KA, Boyd RN, Davies PSW. Relationships between Dietary Intake and Body Composition according to Gross Motor Functional Ability in Preschool-Aged Children with Cerebral Palsy. Ann Nutr Metab. 2012;61(4):349-57.
30. Oftedal S, Bell K, Mitchell L, Davies P, Ware R, Boyd R. A Systematic Review of the Clinimetric Properties of Habitual Physical Activity Measures in Young Children with a Motor Disability. Int J Pediatr. 2012;2012:12.
31. Benfer KA, Weir KA, Bell KL, Ware RS, Davies PS, Boyd RN. Oropharyngeal dysphagia and gross motor skills in children with cerebral palsy. Pediatrics. 2013;131(5):e1553-62.
32. Weir K, Bell K, Ware R, Caristo F, Rawicki B, Fahey M, et al. Reported eating ability of young children with cerebral palsy: Is there an association with gross motor function? . Arch Phys Med Rehabil. 2012;94(3):495-502.
33. Benfer KA, Weir KA, Bell KL, Ware RS, Davies PS, Boyd RN. Oropharyngeal dysphagia in preschool children with cerebral palsy: oral phase impairments. Res Dev Disabil. 2014;35(12):3469-81.
34. Oftedal S, Bell KL, Davies PS, Ware RS, Boyd RN. Relationship between daily physical activity performance and functional mobility capacity and performance in toddlers with cerebral palsy. Dev Med Child Neurol. 2013;55(S3):5.
35. Keawutan P, Bell K, Davies PS, Boyd RN. Systematic review of the relationship between habitual physical activity and motor capacity in children with cerebral palsy. Res Dev Disabil. 2014;35(6):1301-9.
36. Barrett R, Lichtwark G. Gross muscle morphology and structure in spastic cerebral palsy: a systematic review. Dev Med Child Neurol. 2010;52(9):794-804.
37. Graham HK. Botulinum toxin type A management of spasticity in the context of orthopaedic surgery for children with spastic cerebral palsy. Eur J Neurol. 2001;8 (Suppl 5):30-9.
38. Bache CE, Selber P, Graham HK. (ii) The management of spastic diplegia. Curr Orthop. 2003;17(2):88-104.
39. Opheim A, Jahnsen R, Olsson E, Stanghelle JK. Balance in relation to walking deterioration in adults with spastic bilateral cerebral palsy. Phys Ther. 2012;92(2):279-88.
40. Barber L, Barrett R, Lichtwark G. Passive muscle mechanical properties of the medial gastrocnemius in young adults with spastic cerebral palsy. J Biomech. 2011;44(13):2496-500.
41. Barber L, Hastings-Ison T, Baker R, Barrett R, Lichtwark G. Medial gastrocnemius muscle volume and fascicle length in children aged 2 to 5 years with cerebral palsy. Dev Med Child Neurol. 2011;53(6):543-8.
42. Barber L, Barrett R, Lichtwark G. Medial gastrocnemius muscle fascicle active torque-length and Achilles tendon properties in young adults with spastic cerebral palsy. J Biomech. 2012;45(15):2526-30.
43. Wren TA, Cheatwood AP, Rethlefsen SA, Hara R, Perez FJ, Kay RM. Achilles tendon length and medial gastrocnemius architecture in children with cerebral palsy and equinus gait. J Pediatr Orthop. 2010;30(5):479-84.
44. Perry J, Hoffer MM, Giovan P, Antonelli D, Greenberg R. Gait analysis of the triceps surae in cerebral palsy. A preoperative and postoperative clinical and electromyographic study. J Bone Joint Surg. 1974;56(3):511-20.

For peer review only - http://bmjopen.bmj.com/site/about/guidelines.xhtml

45. Eek MN, Beckung E. Walking ability is related to muscle strength in children with cerebral palsy. Gait Posture. 2008;28(3):366-71.
46. Barber LA, Read F, Lovatt Stern J, Lichtwark G, Boyd RN. Medial gastrocnemius muscle volume in ambulant children with unilateral and bilateral cerebral palsy aged 2 to 9 years. Dev Med Child Neurol. 2016.
47. Gage JR, Schwartz MH, Koop SE, Novacheck TF, editors. The Identification and Treatment of Gait Problems in Cerebral Palsy. 2nd ed. London: MacKeith Press; 2009.
48. Winters TF, Gage JR, Hicks R. Gait patterns in spastic hemiplegia in children and young adults. Journal of Bone and Joint Surgery. 1987;69(3):437-41.
49. Howard J, Soo B, Graham HK, Boyd RN, Reid S, Lanigan A, et al. Cerebral palsy in Victoria: Motor types, topography and gross motor function. J Paediatr Child Health. 2005;41(9-10):479-83.
50. Palisano R, Rosenbaum P, Walter RS, Russell D, Wood E, Galuppi B. Development and reliability of a system to classify gross motor function in children with cerebral palsy. Dev Med Child Neurol. 1997;39(4):214-23.
51. Wood EC, Rosenbaum P. The Gross Motor Function Classification System for Cerebral Palsy: A study of reliability and stability over time. Dev Med Child Neurol. 2000;42:292-6.
52. Oeffinger D, Tylkowski C, Rayens M, Davies R, Gorton G, D'Astous J, et al. Gross Motor Function Classification System and outcome tools for assessing ambulatory cerebral palsy: A multicenter study. Dev Med Child Neurol. 2004;46:311-9.
53. Palisano RJ, Rosenbaum P, Bartlett D, Livingston MH. Content validity of the expanded and revised Gross Motor Function Classification System. Dev Med Child Neurol. 2008;50(10):744.
54. Gorter J, Rosenbaum P, Palisano R, Bartlett D, Russell D, Walter S, et al. Limb distribution, motor impairment and functional classification of cerebral palsy. Dev Med Child Neurol. 2004;46:461-7.
55. Surveillance of Cerebral Palsy in E. Surveillance of cerebral palsy in Europe: a collaboration of cerebral palsy surveys and registers. Surveillance of Cerebral Palsy in Europe (SCPE). Dev Med Child Neurol. 2000;42(12):816.
56. Monbaliu E, Ortibus E, De Cat J, Dan B, Heyrman L, Prinzie P, et al. The Dyskinesia Impairment Scale: a new instrument to measure dystonia and choreoathetosis in dyskinetic cerebral palsy. Dev Med Child Neurol. 2012;54(3):278-83.
57. Monbaliu E, de Cock P, Ortibus E, Heyrman L, Klingels K, Feys H. Clinical patterns of dystonia and choreoathetosis in participants with dyskinetic cerebral palsy. Dev Med Child Neurol. 2016;58(2):138-44.
58. Harvey A, Graham H, Morris M, Baker R, Wolfe R. The Functional Mobility Scale: Ability to detect change following single event multilevel surgery. Dev Med Child Neurol. 2007;49:603-7.
59. Harvey AR, Morris ME, Graham HK, Wolfe R, Baker R. Reliability of the functional mobility scale for children with cerebral palsy. Phys Occup Ther Pediatr. 2010;30(2):139-49.
60. Rodda J, Graham HK. Classification of gait patterns in spastic hemiplegia and spastic diplegia: a basis for a management algorithm. Eur J Neurol. 2001;8 (Suppl 5):98-108.
61. Dobson F, Morris ME, Baker R, Graham HK. Gait classification in children with cerebral palsy: A systematic review. Gait Posture. 2007;25(1):140-52.
62. Dobson F, Morris ME, Baker R, Wolfe R, Graham HK. Clinician agreement on gait pattern ratings in children with spastic hemiplegia. Dev Med Child Neurol. 2006;48(6):429-35.
63. Stott NS, Atherton WG, Mackey AH, Galley JJ, Nicol RO, Walsh SJ. The reliability and validity of assessment of sagittal plane deviations in children who have spastic diplegia. Arch Phys Med Rehabil. 2005;86(12):2337-41.
64. Eliasson AC, Krumlinde-Sundholm L, Rosblad B, Beckung E, Arner M, Ohrvall AM, et al. The Manual Ability Classification System (MACS) for children with cerebral palsy: scale development and evidence of validity and reliability. Dev Med Child Neurol. 2006;48(7):549-54.
65. Morris C, Kurinczuk JJ, Fitzpatrick R, Rosenbaum PL. Reliability of the manual ability classification system for children with cerebral palsy. Dev Med Child Neurol. 2006;48(12):950-3.
66. Hidecker MJC, Paneth N, Rosenbaum PL, Kent RD, Lillie J, Eulenberg JB, et al. Developing and validating the Communication Function Classification System for individuals with cerebral palsy. Dev Med Child Neurol. 2011;53(8):704-10.

For peer review only

67. Barty E, Caynes K, Johnston LM. Development and reliability of the Functional Communication Classification System for children with cerebral palsy. Dev Med Child Neurol. 2016;58(10):1036-41.
68. Pennington L, Virella D, Mjøen T, da Graça Andrada M, Murray J, Colver A, et al. Development of The Viking Speech Scale to classify the speech of children with cerebral palsy. Res Dev Disabil. 2013;34(10):3202-10.
69. Sellers D, Mandy A, Pennington L, Hankins M, Morris C. Development and reliability of a system to classify the eating and drinking ability of people with cerebral palsy. Dev Med Child Neurol. 2014;56(3):245-51.
70. Ashwal S, Russman BS, Blasco PA, Miller G, Sandler A, Shevell M, et al. Practice parameter: diagnostic assessment of the child with cerebral palsy. Neurology. 2004;62(6):851-63.
71. Holmes CJ, Hoge R, Collins L, Woods R, Toga AW, Evans AC. Enhancement of MR images using registration for signal averaging. J Comput Assist Tomogr. 1998;22(2):324-33.
72. Fiori S, Cioni G, Klingels K, Ortibus E, Van Gestel L, Rose S, et al. Reliability of a novel, semi-quantitative scale for classification of structural brain magnetic resonance imaging in children with cerebral palsy. Dev Med Child Neurol. 2014;56(9):839-45.
73. Fiori S, Guzzetta A, Pannek K, Ware RS, Rossi G, Klingels K, et al. Validity of semi-quantitative scale for brain MRI in unilateral cerebral palsy due to periventricular white matter lesions: Relationship with hand sensorimotor function and structural connectivity. NeuroImage Clinical. 2015;8:104-9.
74. Pannek K, Raffelt D, Bell C, Mathias JL, Rose SE. HOMOR: higher order model outlier rejection for high b-value MR diffusion data. Neuroimage. 2012;63(2):835-42.
75. Tournier JD, Calamante F, Connelly A. Robust determination of the fibre orientation distribution in diffusion MRI: non-negativity constrained super-resolved spherical deconvolution. Neuroimage. 2007;35(4):1459-72.
76. Pannek K, Mathias JL, Bigler ED, Brown G, Taylor JD, Rose S. An automated strategy for the delineation and parcellation of commissural pathways suitable for clinical populations utilising high angular resolution diffusion imaging tractography. Neuroimage. 2010;50(3):1044-53.
77. Rose S, Rowland T, Pannek K, Baumann F, Coulthard A, McCombe P, et al. Structural hemispheric asymmetries in the human precentral gyrus hand representation. Neuroscience. 2012;210:211-21.
78. Cornell MS, Hatrick NC, Boyd R, Baird G, Spencer JD. The hip in children with cerebral palsy. Predicting the outcome of soft tissue surgery. Clin Orthop. 1997;340(340):165-71.
79. Faraj S, Atherton WG, Stott NS. Inter- and intra-measurer error in the measurement of Reimers' hip migration percentage. J Bone Joint Surg Br. 2004;86(3):434-7.
80. Parrott J, Boyd RN, Dobson F, Lancaster A, Love S, Oates J, et al. Hip displacement in spastic cerebral palsy: repeatability of radiologic measurement. J Pediatr Orthop. 2002;22(5):660.
81. Scrutton D, Baird G. Surveillance measures of the hips of children with bilateral cerebral palsy. Arch Dis Child. 1997;76(4):381-4.
82. Scrutton D, Baird G, Smeeton N. Hip dysplasia in bilateral cerebral palsy: incidence and natural history in children aged 18 months to 5 years. Dev Med Child Neurol. 2001;43(9):586-600.
83. Boyd RN, Nattrass G, Graham HK, Dobson F, Parrott J, Love S, et al. The effect of botulinum toxin type A and a variable hip abduction orthosis on gross motor function: a randomized controlled trial. Eur J Neurol. 2001;8 (Suppl 5):109-19.
84. Stevenson RD. Use of Segmental Measures to Estimate Stature in Children with Cerebral Palsy. Arch Pediatr Adolesc Med. 1995;149:658-62.
85. Ogden CL, Kuczmarski RJ, Flegal KM, Mei Z, Guo S, Wei R, et al. Centers for Disease Control and Prevention 2000 growth charts for the United States: improvements to the 1977 National Center for Health Statistics version. Pediatrics. 2002;109(1):45-60.
86. Russell D, Rosenbaum P, Avery L, Lane M. Gross motor function measure (GMFM-66 and GMFM-88) user's manual: MacKeith press; 2002.
87. Holmefur M, Krumlinde-Sundholm L, Bergstrom J, Eliasson AC. Longitudinal development of hand function in children with unilateral cerebral palsy. Dev Med Child Neurol. 2010;52(4):352-7.
88. Elvrum A-KG. Assessment of hand function in children with bilateral cerebral palsy: Development and measurement properties of outcome measures and classifications [Degree of Philosophiae Doctor]. Trondheim, Norway: Norwegian University of Science and Technology; 2016.

For peer review only - http://bmjopen.bmj.com/site/about/guidelines.xhtml

89. Oldfield RC. The assessment and analysis of handedness: the Edinburgh inventory. Neuropsychologia. 1971;9(1):97-113.
90. Auld ML, Boyd RN, Moseley GL, Ware RS, Johnston LM. Impact of tactile dysfunction on upper-limb motor performance in children with unilateral cerebral palsy. Arch Phys Med Rehabil. 2012;93(4):696-702.
91. Sakzewski L, Ziviani J, Boyd R. The relationship between unimanual capacity and bimanual performance in children with congenital hemiplegia. Dev Med Child Neurol. 2010;52(9):811-6.
92. Hägglund G, Lauge-Pedersen H, Wagner P. Characteristics of children with hip displacement in cerebral palsy. BMC musculoskeletal disorders. 2007;8(1):101-.
93. Wynter M, Gibson N, Kentish M, Love S, Thomason P, Kerr Graham H. The development of Australian Standards of Care for Hip Surveillance in Children with Cerebral Palsy: how did we reach consensus? J Pediatr Rehabil Med. 2011;4(3):171-82.
94. Reimers J, Bialik V. Influence of femoral rotation on the radiological coverage of the femoral head in children. Pediatr Radiol. 1981;10(4):215-8.
95. Foroohar A, McCarthy JJ, Yucha D, Clarke S, Brey J. Head-shaft angle measurement in children with cerebral palsy. J Pediatr Orthop. 2009;29(3):248-50.
96. Hilgenreiner H. Early diagnosis and early treatment of congenital dislocation of the hip. Med Klin. 1925;21:1385-425.
97. Robin J, Graham HK, Selber P, Dobson F, Smith K, Baker R. Proximal femoral geometry in cerebral palsy: a population-based cross-sectional study. J Bone Joint Surg Br. 2008;90(10):1372-9.
98. Birkenmaier C, Jorysz G, Volkmar J, Heimkes B. Normal development of the hip: a geometric analysis based on planimetric radiography. J Pediatr Orthop B. 2010;19(1):1-8.
99. Haike H, Breuckmann G, Schulze H. [Surgical treatment of so-called congenital hip dislocation]. Arch Orthop Unfallchir. 1969;66(4):277-85.
100. Morrissy RT, Goldsmith G, Hall E, Kehl D, Cowie G. Measurement of the Cobb angle on radiographs of patients who have scoliosis. Evaluation of intrinsic error. J Bone Joint Surg. 1990;72(3):320-7.
101. Henderson RC, Henderson BA, Kecskemethy HH, Hidalgo ST, Nikolova BA, Sheridan K, et al. Adaptation of the lateral distal femur DXA scan technique to adults with disabilities. J Clin Densitom. 2015;18(1):102-8.
102. Henderson RC, Lark RK, Newman JE, Kecskemthy H, Fung EB, Renner JB, et al. Pediatric reference data for dual X-ray absorptiometric measures of normal bone density in the distal femur. AJR Am J Roentgenol. 2002;178(2):439-43.
103. Mueske NM, Chan LS, Wren TA. Reliability of lateral distal femur dual-energy X-ray absorptiometry measures. J Clin Densitom. 2014;17(4):522-7.
104. Zemel BS, Stallings VA, Leonard MB, Paulhamus DR, Kecskemethy HH, Harcke HT, et al. Revised pediatric reference data for the lateral distal femur measured by Hologic Discovery/Delphi dual-energy X-ray absorptiometry. J Clin Densitom. 2009;12(2):207-18.
105. Carter DR, Bouxsein ML, Marcus R. New approaches for interpreting projected bone densitometry data. J Bone Miner Res. 1992;7(2):137-45.
106. Australian Radiation Protection and Nuclear Safety Agency. Code of practice: Exposure of humans to ionizing radiation for research purposes. Canberra, Australia: 2005.
107. Duke PM, Litt IF, Gross RT. Adolescents' Self-Assessment of Sexual Maturation. Pediatrics. 1980;66(6):918.
108. Varni JW, Thompson KL, Hanson V. The Varni/Thompson Pediatrie Pain Questionnaire. I. Chronic musculoskeletal pain in juvenile rheumatoid arthritis. Pain. 1987;28(1):27-38.
109. Gragg RA, Rapoff MA, Danovsky MB, Lindsley CB, Varni JW, Waldron SA, et al. Assessing Chronic Musculoskeletal Pain Associated with Rheumatic Disease: Further Validation of the Pediatric Pain Questionnaire. J Pediatr Psychol. 1996;21(2):237-50.
110. Kainz H, Modenese L, Lloyd DG, Maine S, Walsh HP, Carty CP. Joint kinematic calculation based on clinical direct kinematic versus inverse kinematic gait models. J Biomech. 2016;49(9):1658-69.
111. Steele KM, Rozumalski A, Schwartz MH. Muscle synergies and complexity of neuromuscular control during gait in cerebral palsy. Dev Med Child Neurol. 2015;57(12):1176-82.
112. Delp SL, Anderson FC, Arnold AS, Loan P, Habib A, John CT, et al. OpenSim: open-source software to create and analyze dynamic simulations of movement. IEEE Trans Biomed Eng. 2007;54(11):1940-50.

For peer review only - http://bmjopen.bmj.com/site/about/guidelines.xhtml

113. Baker R, McGinley JL, Schwartz MH, Beynon S, Rozumalski A, Graham HK, et al. The gait profile score and movement analysis profile. Gait Posture. 2009;30(3):265-9.
114. Lichtwark GA, Wilson AM. Interactions between the human gastrocnemius muscle and the Achilles tendon during incline, level and decline locomotion. J Exp Biol. 2006;209(21):4379-88.
115. Cronin NJ, Carty CP, Barrett RS, Lichtwark G. Automatic tracking of medial gastrocnemius fascicle length during human locomotion. J Appl Physiol. 2011;111(5):1491-6.
116. Gillett JG, Barrett RS, Lichtwark GA. Reliability and accuracy of an automated tracking algorithm to measure controlled passive and active muscle fascicle length changes from ultrasound. Comput Methods Biomech Biomed Engin. 2013;16(6):678-87.
117. Barber L, Barrett R, Lichtwark G. Validation of a freehand 3D ultrasound system for morphological measures of the medial gastrocnemius muscle. J Biomech. 2009;42(9):1313-9.
118. Verschuren O, Ketelaar M, Keefer D, Wright V, Butler J, Ada L, et al. Identification of a core set of exercise tests for children and adolescents with cerebral palsy: a Delphi survey of researchers and clinicians. Dev Med Child Neurol. 2011;53(5):449-56.
119. Maher CA, Williams MT, Olds TS. The six-minute walk test for children with cerebral palsy. Int J Rehabil Res. 2008;31(2):185-8.
120. Li AM, Yin J, Au JT, So HK, Tsang T, Wong E, et al. Standard reference for the six-minute-walk test in healthy children aged 7 to 16 years. Am J Respir Crit Care Med. 2007;176(2):174-80.
121. ATS statement. Am J Respir Crit Care Med. 2002;166(1):111-7.
122. Verschuren O, Takken T, Ketelaar M, Gorter JW, Helders PJ. Reliability for running tests for measuring agility and anaerobic muscle power in children and adolescents with cerebral palsy. Pediatr Phys Ther. 2007;19(2):108-15.
123. Verschuren O, Bongers BC, Obeid J, Ruyten T, Takken T. Validity of the muscle power sprint test in ambulatory youth with cerebral palsy. Pediatr Phys Ther. 2013;25(1):25-8.
124. Thompson P, Beath T, Bell J, Jacobson G, Phair T, Salbach NM, et al. Test-retest reliability of the 10-metre fast walk test and 6-minute walk test in ambulatory school-aged children with cerebral palsy. Dev Med Child Neurol. 2008;50(5):370-6.
125. Verschuren O, Ketelaar M, Takken T, Van Brussel M, Helders PJ, Gorter JW. Reliability of hand-held dynamometry and functional strength tests for the lower extremity in children with Cerebral Palsy. Disabil Rehabil. 2008;30(18):1358-66.
126. Trost SG, McIver KL, Pate RR. Conducting accelerometer-based activity assessments in field-based research. Med Sci Sports Exerc. 2005;37(11 Suppl):S531-43.
127. Clanchy K, Tweedy S, Boyd R. Measurement of Habitual Physical Activity Performance in Adolescents with Cerebral Palsy. Dev Med Child Neurol. 2011;53(6):499-505.
128. Clanchy KM, Tweedy SM, Boyd RN, Trost SG. Validity of accelerometry in ambulatory children and adolescents with cerebral palsy. Eur J Appl Physiol. 2011;111(12):2951-9.
129. National Health and Medical Research Council. Nutrient Reference Values for Australia and New Zealand. Canberra, Australia: Department of Health and Ageing, 2005.
130. Taylor C, Lamparello B, Kruczek K, Anderson EJ, Hubbard J, Misra M. Validation of a food frequency questionnaire for determining calcium and vitamin D intake by adolescent girls with anorexia nervosa. J Am Diet Assoc. 2009;109(3):479-85, 85 e1-3.
131. Harrison SL, Buettner PG, Maclennan R. The North Queensland "Sun-Safe Clothing" study: design and baseline results of a randomized trial to determine the effectiveness of sun-protective clothing in preventing melanocytic nevi. Am J Epidemiol. 2005;161(6):536-45.
132. Sheppard J. Dysphagia Disorders Survey and Dysphagia Management Staging Scale (Adult and Pediatric Applications): User's Manual: Australian Edition. Ryde, NSW: The Centre for Developmental Disability 2003.
133. Sheppard JJ, Hochman R, Baer C. The Dysphagia Disorder Survey: Validation of an assessment for swallowing and feeding function in developmental disability. Res Dev Disabil. 2014;35(5):929-42.
134. Benfer K, Weir K, Bell K, Davies P, Ware R, Boyd R. Longitudinal cohort protocol study of oropharyngeal dysphagia: relationships to gross motor attainment, growth and nutritional status in preschool children with cerebral palsy. BMJ Open 2012;2(4):e001460.

For peer review only - http://bmjopen.bmj.com/site/about/guidelines.xhtml

135. Calis EA, Veugelers R, Sheppard JJ, Tibboel D, Evenhuis HM, Penning C. Dysphagia in children with severe generalized cerebral palsy and intellectual disability. Dev Med Child Neurol. 2008;50(8):625-30.
136. Sheppard J. Nutritional correlates of dysphagia in individuals institutionalized with mental retardation. Dysphagia. 1998;3:85-9.
137. Skuse D, Stevenson J, Reilly S, Mathisen B. Schedule for oral-motor assessment (SOMA): Methods of validation. Dysphagia. 1995;10:192-202.
138. Weir K, McMahon S, Barry L, Masters IB, Chang AB. Clinical signs and symptoms of oropharyngeal aspiration and dysphagia in children. Eur Respir J. 2009;33(3):604-11.
139. DeMatteo C, Matovich D, Hjartson A. Comparison of clinical and videofluoroscopic evaluation of children with feeding and swallowing difficulties. Dev Med Child Neurol. 2005;47(3):149-57.
140. Lefton-Greif M, McGrath-Morrow S. Deglutition and respiration: development, coordination, and practical implications. . Semin Speech Lang. 2007;28:166-79.
141. Thomas-Stonell N, Greenberg J. Three treatment approaches and clinical factors in the reduction of drooling. Dysphagia 1998;3:73-8.
142. Benfer KA, Weir KA, Bell KL, Ware RS, Davies PS, Boyd RN. Longitudinal Study of Oropharyngeal Dysphagia in Preschool Children With Cerebral Palsy. Arch Phys Med Rehabil. 2016;97(4):552-60 e9.
143. Lefton-Greif MA, Okelo SO, Wright JM, Collaco JM, McGrath-Morrow SA, Eakin MN. Impact of children's feeding/swallowing problems: validation of a new caregiver instrument. Dysphagia. 2014;29(6):671-7.
144. Semel E, Wiig E, Secord W. Clinical Evaluation of Language Fundamentals. 4th edition ed. Marrickville Australia.: Harcourt Assessment, Inc; 2006.
145. Fenson L, Marchmann V, Thal D, Dale P, JS. R, Bates E. McArthur Bates Communicative Development Inventory. 2nd edition ed. Baltimore, Maryland.: Paul H Brookes Publishing Co; 2007.
146. Hayden D, Square P. Verbal Motor Performance Assessment for Children: Examiner's Manual. USA.: Psychological Corporation; 1999.
147. Haley SM, Coster WJ, Dumas HM, Fragala-Pinkham MA, Moed R. Pediatric Evaluation of Disability Inventory Computer Adaptive Test, Development, Standardization and Administration Manual Version 1.3.6. Boston, MA: CRECare LLC; 2012.
148. Haley SM, Andrellos PJ, Coster W, Haltiwagner JT, Ludlow LH. Pediatric Evaluation of Disability Inventory: New England Medical Center Hospitals; 1992.
149. Coster W, Bedell G, Law M, Khetani MA, Teplicky R, Liljenquist K, et al. Psychometric evaluation of the Participation and Environment Measure for Children and Youth. Dev Med Child Neurol. 2011;53(11):1030-7.
150. Goodman R. The Strengths and Difficulties Questionnaire: A research note. Journal of Child Psychology and Psychiatry. 1997;38(5):581-6.
151. Goodman R. The extended version of the Strengths and Difficulties Questionnaire as a guide to psychiatric caseness and consequent burden. Journal of Child Psychology and Psychiatry. 1999;40(5):791-9.
152. Goodman R. Psychometric propeties of the Strengths and Difficulties Questionnaire. J Am Acad Child Adolesc Psychiatry. 2001;40:1337-45.
153. Australian Government. A Snapshot of Early Childhood Development in Australia 2012 - AEDI National Report. Canberra: The Australian Government, 2009.
154. Australian Curriculum Assessment and Reporting Authority. Measurement Framework for Schooling in Australia. Sydney, Australia: Education Council, 2013.
155. Cotton SM, Kiely PM, Crewther DP, Thomson B, Laycock R, Crewther SG. A normative and reliability study for the Raven's Coloured Progressive Matrices for primary school aged children from Victoria Australia. Pers Individ Dif. 2005;39(3):647-59.
156. Pueyo R, Junqué C, Vendrell P, Narberhaus A, Segarra D. Raven's coloured progressive matrices as a measure of cognitive functioning in cerebral palsy. J Intellect Disabil Res. 2008;52(5):437-45.
157. Gioia G, Isquith P, Guy S, Kenworthy L. Behavior Rating Inventory of Executive Function. Lutz , Florida: Parnic; 2000.
158. Reynolds C, Kamphaus R. Behavior Assessment System for children.: Circle Pines, MN: American Guidance Service; 1992.

For peer review only - http://bmjopen.bmj.com/site/about/guidelines.xhtml

159. Achenbach T. Integrative Guide to the 1991 CBCL/4-18, YSR, and TRF Profiles. Burlington, VT: University of Vermont,: Department of Psychology 1991.
160. Conners CK. Conner's Rating Scales-Revised Technical Manual. Toronto: Multi-Health Systems 1997.
161. Allison C, Auyeung B, Baron-Cohen S. Toward brief “red flags” for autism screening: the short autism spectrum quotient and the short quantitative checklist in 1,000 cases and 3,000 controls. J Am Acad Child Adolesc Psychiatry. 2012;51(2):202-12. e7.
162. Newman CJ, O'Regan M, Hensey O. Sleep disorders in children with cerebral palsy. Dev Med Child Neurol. 2006;48(07):564-8.
163. Bruni O, Ottaviano S, Guidetti V, Romoli M, Innocenzi M, Cortesi F, et al. The Sleep Disturbance Scale for Children (SDSC). Construction and validation of an instrument to evaluate sleep disturbances in childhood and adolescence. J Sleep Res. 1996;5(4):251-61.
164. Davis E, Shelly A, Waters E, Mackinnon A, Reddihough D, Boyd R, et al. Quality of life for adolescents with cerebral palsy: Perspectives of adolescents and parents. Dev Med Child Neurol. 2009;51:193.
165. Shelly A, Davis E, Waters E, Mackinnon A, Reddihough D, Boyd R, et al. The relationship between quality of life and functioning for children with cerebral palsy. Dev Med Child Neurol. 2008;50(3):199-203.
166. Waters E, Davis E, Mackinnon A, Boyd R, Graham H, Kai Lo S, et al. Psychometric properties of the quality of life questionnaire for children with CP. Dev Med Child Neurol. 2007;49(1):49-55.
167. Olree KS, Engsberg JR, Ross SA, Park TS. Changes in synergistic movement patterns after selective dorsal rhizotomy. Dev Med Child Neurol. 2000;42(5):297-303.
168. Stevens K. Developing a descriptive system for a new preference- based measure of health related quality of life forchildren. Qual Life Res. 2009;18:1105-13.
169. Stevens K. Working with children to develop dimensions for a preference-based generic paediatric health related quality of life measure. Qual Life Res. 2010;20:340-51.
170. Stevens K. Assessing the performance of a new generic measure of health-related quality of life for children and refining it for use in health state valuation Applied Health Economics and Health Policy. 2011;9(3):157-69.
171. Brooks R. EuroQol: the current state of play. Health Policy. 1996;37(1):53-72.
172. Herdman M, Gudex C, Lloyd A, Janssen M, Kind P, Parkin D, et al. Development and preliminary testing of the new five-level version of EQ-5D (EQ-5D-5L). Qual Life Res. 2011;20(10):1727-36.
173. Norman R, Cronin P, Viney R. A Pilot Discrete Choice Experiment to Explore Preferences for EQ-5D-5L Health States. Applied Health Economics and Health Policy. 2013;11(3):287-98.
174. Statistics ABo. Census of population and housing: socio-economic indexes for areas (SEIFA), Australia 2011. 2014.
175. Al-Janabi H, Coast J, Flynn TN. What do people value when they provide unpaid care for an older person? A meta-ethnography with interview follow-up. Soc Sci Med. 2008;67(1):111-21.
176. Al-Janabi H, Flynn TN, Coast J. Estimation of a preference-based carer experience scale. Med Decis Making. 2011;31(3):458-68.
177. Brehaut JC, Kohen DE, Raina P, Walter SD, Russell DJ, Swinton M, et al. The Health of Primary Caregivers of Children With Cerebral Palsy: How Does It Compare With That of Other Canadian Caregivers? Pediatrics. 2004;114(2):e182-e91.
178. Epstein NB, Baldwin LM, Bishop DS. The McMaster family assessment device. J Marital Fam Ther. 1983;9(2):171-80.
179. Miller IW, Epstein NB, Bishop DS, Keitner GI. The McMaster family assessment device: reliability and validity. J Marital Fam Ther. 1985;11(4):345-56.
180. Byles J, Byrne C, Boyle MH, Offord DR. Ontario Child Health Study: Reliability and Validity of the General Functioning Subscale of the McMaster Family Assessment Device. Fam Process. 1988;27(1):97-104.
181. Rosenbaum PL, Walter SD, Hanna SE, Palisano RJ, Russell DJ, Raina P, et al. Prognosis for gross motor function in cerebral palsy: creation of motor development curves. J Am Med Assoc. 2002;288(11):1357-63.

For peer review only - http://bmjopen.bmj.com/site/about/guidelines.xhtml

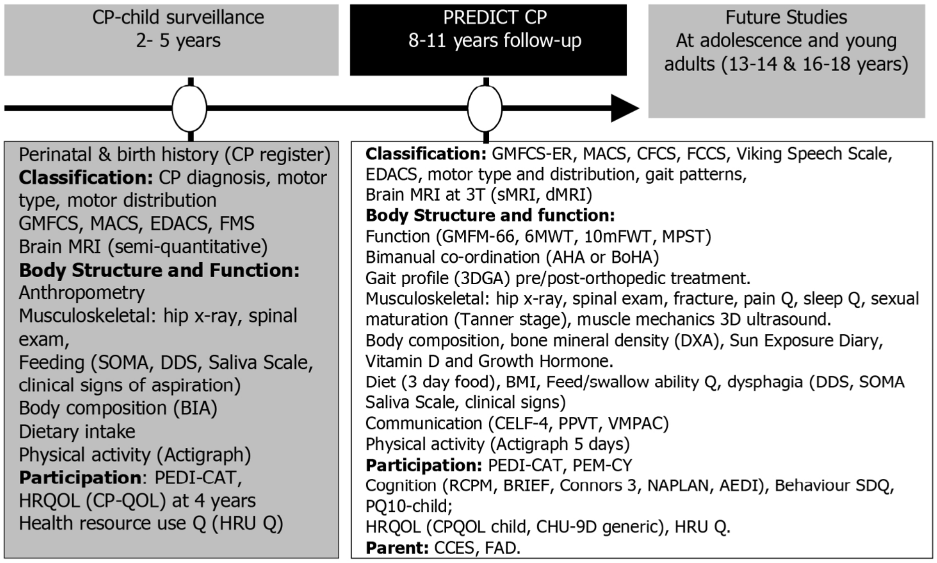

Key: AHA=Assisting hand Assessment; AEDI=Australian Early Development Index; BIA=Bioelectrical Impedance Analysis; BMI=Body Mass Index; BoHA=Both Hands Assessment; BRIEF=Behaviour Rating Inventory of Executive Function; CELF-4=Clinical Evaluation of Language Fundamentals-4; CFCS=Communication Function Classification System; CHU9D=Child Health Utility 9D; CCES=Carer Experience scale; CP-QOL=Cerebral Palsy Quality of Life Scale; 3DGA=3 dimensional gait analysis; DDS=Dysphagia Disorders Survey; dMRI=diffusion MRI; DXA=Dual-energy X-ray Absorptiometry; FAD=McMaster Family Assessment Device General Functioning Scale; FMS=Functional Mobility Scale; FCCS=Functional Communication Classification System; EDACS=Eating & Drinking Ability Classification System; GMFCS-ER=Gross Motor Function Classification System Expanded & Revised; GMFM-66=Gross Motor Ability Estimator; HRQOL=Health Related Quality of Life; MACS=Manual Ability Classification System; MPST=Muscle Power Sprint test; 6MWT=Six Minute Walk Test; 10mFWT=10 Metre Fast Walk test; NAPLAN=National Assessment Program (Literacy and Numeracy); PEDI-CAT=Paediatric Evaluation of Disability Inventory Computer Adapted Test; PEM-CY= Participation & Environment Measure for Children and Youth; PQ10-child=Autism Spectrum Quotient child; RCPM= Raven Coloured Progressive Matrices; SDQ=Strengths & Difficulties Q; PPVT= Peabody Picture Vocabulary Test; SOMA=Schedule for Oral Motor Assessment; VMPAC=Verbal Motor Performance Assessment for Children.

Figure 1: Summary of surveillance and outcome measures for PREDICT study

173x171mm (300 x 300 DPI)

Appendix 1: Qld Predict CP – Physician's checklist

**Diagnosis**		COMMENTS
Cerebral palsy	yes/ no/ unsure	Definition (Rosenbaum et al., 2005): "Cerebral Palsy (CP) describes a group of disorders of the development of movement and posture, causing activity limitation that are attributed to non-progressive disturbances that occurred in the developing foetal or infant brain. The motor disorders of cerebral palsy are often accompanied by disturbances of sensation, cognition, communication, perception, and/or behaviour, and/or by a seizure disorder."<br><br>What constitutes Cerebral Palsy? (Badawi et al., 1998): Cerebral palsy (CP) is a term of convenience applied to a group of motor disorders of central origin defined by clinical description. It is not a diagnosis in that its application infers nothing about pathology, aetiology or prognosis. It is an umbrella term covering a wide range of disorders which result in childhood motor impairment. There must be motor impairment, and this impairment must stem from a malfunction of the brain (rather than spinal cord or muscles). Furthermore, the brain malfunction must be non-progressive and it must manifest early in life.

Patterns of motor impairment

			COMMENTS	
Motor type	Spastic		See attachment for further information (SCPE definitions, 2000<br><br>For mixed motor type state 1 for dominant and 2 for secondary etc.	
	Ataxic			
	dystonic			
	choreoathetotic			
	Hypotonic			
	Hyperkinetic			
Distribution	Bilateral/ unilateral			
	No of limbs (based on activity or function not passive testing of muscle tone)		1/ 2/ 3/ 4	
Head circumference –				

For peer review only - http://bmjopen.bmj.com/site/about/guidelines.xhtml

For peer review only

current (cm)			

Functional level		COMMENTS
GMFCS level	I/ II/ III/ IV/ V	
Age at classification	years months	Age of child when this assessment is being performed (and GMFCS is classified)
Upper limb/ Handedness	Right predominant/ Left predominant/ Bilateral	

Co-morbidities		COMMENTS
Epilepsy	No<br><br>Yes (defined by 2 unprovoked seizures excluding febrile or neonatal seizures)<br><br>If yes, still on medication, Yes/ No	
Seizure type		Ask the family to describe exactly what they observed.<br><br>Generalised or partial<br><br>Generalised – sudden onset of seizures that compromises responsiveness and affects the whole body.<br><br>Partial – seizures have focality therefore symptoms reflect onset in 1 part of the brain<br><br>Date of commencement of seizures
Seizure syndrome		
Controlled/ not controlled		
Medications	Yes/ no/ not applicable	Medications for seizures or any other medications

For peer review only - http://bmjopen.bmj.com/site/about/guidelines.xhtml

Visual impairment<br><br>(after correction, on the better eye)	Normal<br><br>Impaired<br><br>Severely impaired (blind or no useful vision)	
Hearing impairment (before correction, on the better ear)	Normal<br><br>Impaired<br><br>Severely impaired (hearing loss > 70 dB	
Speech	Normal<br><br>Delayed	Expressive<br><br>Receptive<br><br>Both Unclassified
Communication	Oral<br><br>Signs<br><br>Device	
Intellectual impairment	Mild<br><br>Moderate<br><br>Severe<br><br>Probably impaired, severity unknown<br><br>Probably no impairment<br><br>No impairment<br><br>Unknown	SCPE classification:<br><br>Normal- IQ>=85, attendance of regular school without support<br><br>Borderline- IQ 70 to 84<br><br>Mild impairment- IQ 50 to 69, some basic literacy and numeracy achieved<br><br>Moderate impairment- IQ 20-49<br><br>Severe impairment- IQ < 20
Nutrition		
Body weight	kg / percentile	
Body height	cm / percentile	

For peer review only - http://bmjopen.bmj.com/site/about/guidelines.xhtml

Method of nutrition	Oral<br><br>Tube- nasogastric<br><br>PEG	PEG – percutaneous endoscopic gastrostomy
	Partially<br><br>Entirely	

Respiratory Health		COMMENTS
No of hospitalisations for chest infections in the past 6 months (since last visit)		
No of episodes of pneumonia		
Asthma	yes/ no	
No of episodes of asthmatic attacks in the past 6 months (since last visit)		
Others	Constipation<br><br>Diabetes<br><br>Cardiac conditions	
Continence	Urinary<br><br>Faecal	
Past surgical history		
Examination findings		Clinical signs and symptoms
MRI previous (yes/no)<br><br>date<br><br>location		

For peer review only - http://bmjopen.bmj.com/site/about/guidelines.xhtml

Appendix 2: Predict CP study - 7 day physical activity monitor log

Thank you for helping with our research! This study aims to identify when your child is active and when they are inactive, so that we can work out how much movement and physical activity children with Cerebral Palsy do as part of their daily life.

To do this we are using an activity monitor together with a logbook of wear time. To help make sure our measures are as accurate as possible, please read the instructions below.

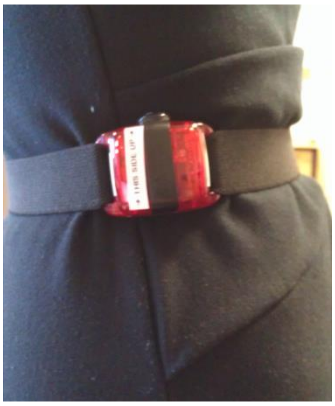

The activity monitor

Where do I wear it? The belt should be worn around your child's waist with the monitor on the side of the hip, on the dominant side. That is, wear the monitor on the (circle) RIGHT / LEFT side.

Position the Actigraph with the arrows pointing up and the open/close button on top. Remember that the monitor is already on and recording and don't worry if it flashes.

How many days should I wear this? Seven days. This should be made up of five weekdays and two weekend days.

How long do I wear it? The monitor should be worn as much as possible during waking hours. It can be hidden under clothing as long as it is on the belt and firm against the waist on the side of the hip, on the unaffected side.

Can I take the monitor off? Take the monitor off only:
When going to sleep; or for water activities such as having a shower, or swimming.

Important points:

Wear the monitor on the belt around the waist on the side of the hip, on the dominant side!
Wear the monitor all hours your child is awake, taking it off only to sleep or if having a swim or shower.

The log book

The log book allows us to compare the movement of the activity monitor to what you record doing at the time – this allows us to check it is accurate.

What do I need to do? At the top of the form are some questions about when the monitor was worn and when your child was asleep. Underneath this we then need you to we need you to record for each

For peer review only

For peer review only - http://bmjopen.bmj.com/site/about/guidelines.xhtml

half hour if the monitor was on or off. Under this, each block represents 15minutes of activity. Simply tick the box that corresponds to the activity – eg. Swimming, sleeping, sitting or walking.

How many days should I do this? The same seven days that your child is wearing the activity monitor.

Monitor on: _____am/pm ______________ Monitor off: _____am/pm ______________

Once you have completed the seven days, put both the activity monitor and the log book into the return mail bag provided, peel off the registered post number, and put it into a Yellow Express Post Box to return it to us.

Thank you for your help and participation in this study.

For peer review only

For peer review only - http://bmjopen.bmj.com/site/about/guidelines.xhtml

Appendix 3: PREDICT CP- 7 day sun Exposure Diary

For peer review only

For peer review only - http://bmjopen.bmj.com/site/about/guidelines.xhtml

PREDICT CP 7- Day Sun Exposure Diary

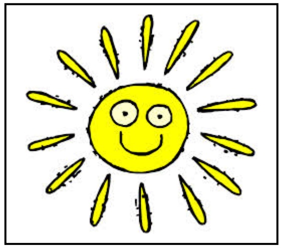

Name of Child: ………………………………………… **Date of Birth:** ………………… **Dates of Diary:** …………………………………………

For peer review only

Instructions for completing diary
Please read the following instructions on how to complete your sun exposure diary.

- **Please answer all the questions on the first page**

- **For each day:** record the date

- **Time Outside in the Sun**: For each hour interval, put a cross in the box under the amount of time you spent in the sun during that interval. Please **do not leave** any hour interval(s) not ticked; i.e. tick '0 minutes' if you have not been exposed to the sun for that particular hour interval.

- **Sunscreen:** Put a cross in the 'YES" or 'NO' box on the top right of the page to indicate if you have applied sunscreen that day. If sunscreen was applied, shade in the area(s) on the diagram to reflect where you have applied sunscreen on your body that day. Please also put a cross in a box under the "**Did You Apply Sunscreen"** column, against the hour(s) of the day at which you applied sunscreen. If you did not apply sunscreen that day, do not put a cross in any of the boxes under this column

- **Clothing Worn**: Use the '**Sun Diary Clothing Guide**' to fill in the type of clothing you wore for each hour interval that day. Insert the relevant number that matches the picture for upper and lower body, headwear, and footwear. Please make sure you have specified all clothing worn at each hour interval, and that you have **written a number in every box in this section.** Please put a cross against the hour(s) that gloves have been worn that day. If no gloves have been worn, do not put a cross in any of the boxes in the gloves column.

To return the diary
A courier will collect the completed sun exposure diary from your home at a time that is convenient to you

Any questions please contact **Camilla:** 30697355, camilla.davenport@uq.edu.au

**Office Use Only**
RA signature:
RA name:
Date:
Study ID:

For peer review only - http://bmjopen.bmj.com/site/about/guidelines.xhtml

Questions about usual sun exposure

Please answer **all** the following questions. Please **circle** or write your answers in the space provided.

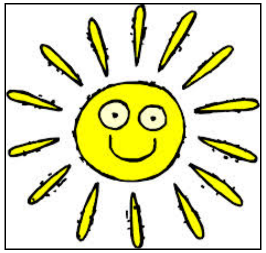

1. Have you been on a holiday in the sun in last month? YES / NO

2. Does your child usually wear sunscreen? YES / NO

   If **YES** what brand? ____________________

   What SPF factor? ______________________

3. What is the colour of your child's **untanned** skin?

   Very fair

   Fair

   Olive

   Light brown

   Dark Brown

For peer review only

Please refer to the following information as a guide to filling out the Sun Exposure Diary.

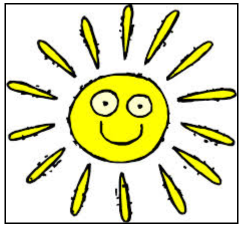

For peer review only

Day Example: Monday 01/07/2015

Name: Elsa Snow

Sunscreen: applied at 6.00am on face, neck, upper and lower limbs (excluding palms of hands and feet), again at 12 noon.

Time Outside in the Sun:

- 6-7 am: 15 minutes, playing **outside** at home
- 8-9 am: 20 minutes, driving to school in car
- 12-1 pm: 10 minutes, playing **outside** at school
- 1-2 pm: 10 minutes, playing **outside** at school
- 5-6 pm: 10 minutes, walking to swimming lessons **outside**
- 6-7 pm: 30 minutes, swimming lessons indoors

Type of Clothing Worn:

- 6 am to 6 pm: short-sleeved top with shorts, no headwear, enclosed shoes. These items worn until 6.00pm.
- 12-1pm & 1-2pm: "as above" with legionnaire's cap

For peer review only - http://bmjopen.bmj.com/site/about/guidelines.xhtml

- 6-7 pm: swimsuit, no headwear, no foot or hand wear.

SUN DIARY CLOTHING GUIDE

**UPPER BODY**	**NO CLOTHING ON UPPER BODY** **0** **No upper clothing**	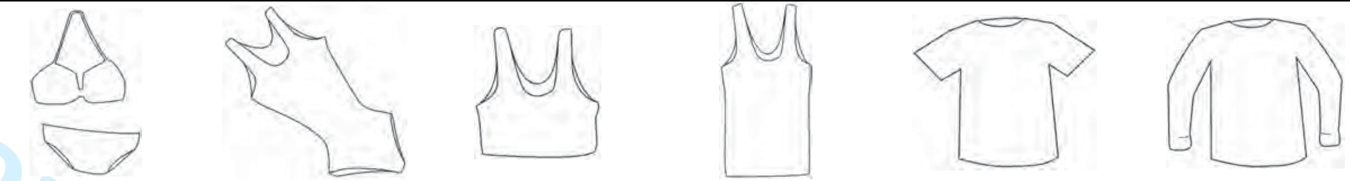 **1** **Bikini**	**2** **Swimsuit**	**3** **Crop top**	**4** **Singlet top**	**5** **Short-sleeved top**	**6** **Long-sleeved top**
**LOWER BODY**	**NO CLOTHING ON LOWER BODY** **0** **No lower clothing**	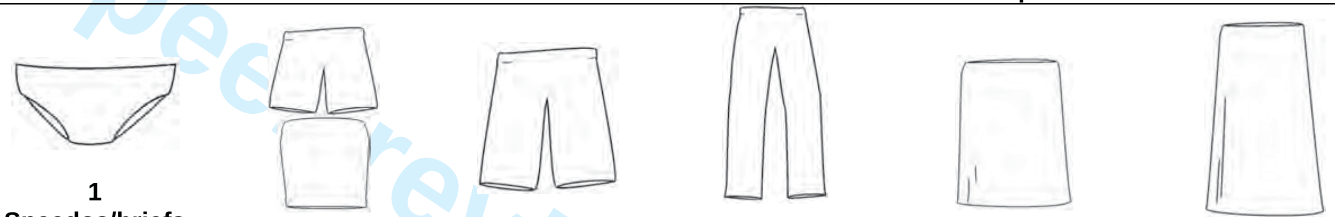 **1** **Speedos/briefs**	**2** **Shorts or short skirt**	**3** **Medium shorts or 3/4 pants**	**4** **Long trousers/ jeans**	**5** **Medium skirt**	**6** **Long skirt**
**HEADWEAR**	**NO HEADWEAR** **0** **No headwear**	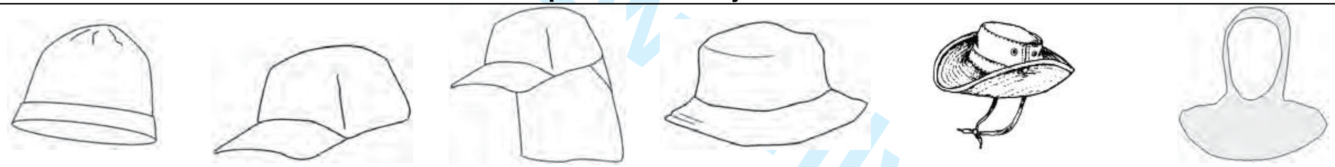 **1** **Beanie**	**2** **Cap**	**3** **Legionnaire's cap**	**4** **Bucket hat**	**5** **Wide-brimmed hat**	**6** **Veil/burkha**
**FOOTWEAR**	**NO FOOTWEAR/ HANDWEAR** **0** **No footwear**	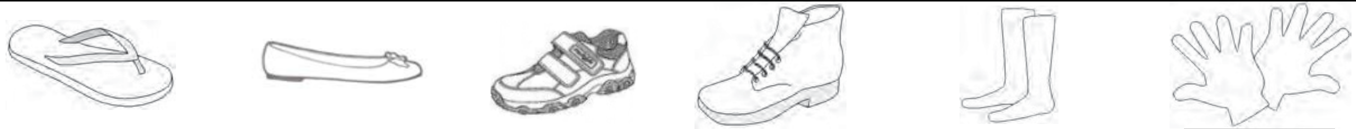 **1** **Thong/open sandals**	**2** **Semi-enclosed shoes**	**3** **Enclosed shoes**	**4** **Boots**	**5** **Long socks**	**HANDWEAR** **Gloves**

For peer review only - http://bmjopen.bmj.com/site/about/guidelines.xhtml

DAY EXAMPLE

DATE 01 / 07 / 15

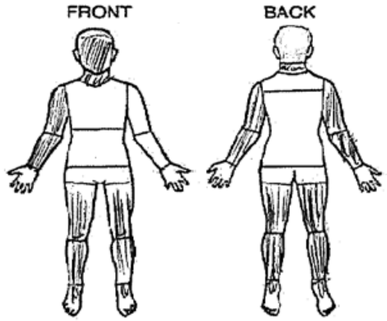

Did you apply sunscreen today?

☑ Yes ☐ No

If yes, shade the diagram on the left to show the parts of the body that you applied sunscreen to today

	TIME OUTDOORS (Cross the box which best represents the amount of time you spent outdoors during each one hour interval shown below)					DID YOU APPLY SUNSCREEN	INSERT NUMBER DENOTING TYPE OF CLOTHING WORN (You must write one number in each box)				
	0 minutes	Below 15 minutes	15-30 minutes	30-45 minutes	45-60 minutes	Cross as many as applicable	Upper body	Lower body	Head wear	Footwear	Gloves (cross all that apply)
**Morning**											
5 - 6 am	☑	☐	☐	☐	☐	☐	0	0	0	0	☐
6 - 7 am	☐	☐	☑	☐	☐	☑	5	2	0	3	☐
7 - 8 am	☑	☐	☐	☐	☐	☐	5	2	0	3	☐
8 - 9 am	☑	☐	☐	☐	☐	☐	5	2	0	3	☐
9 - 10 am	☑	☐	☐	☐	☐	☐	5	2	0	3	☐
10 - 11 am	☑	☐	☐	☐	☐	☐	5	2	0	3	☐
11 - 12 am	☑	☐	☐	☐	☐	☐	5	2	0	3	☐
**Afternoon**											
12 - 1 pm	☐	☑	☐	☐	☐	☑	5	2	3	3	☐
1 - 2 pm	☐	☑	☐	☐	☐	☐	5	2	3	3	☐
2 - 3 pm	☑	☐	☐	☐	☐	☐	5	2	0	3	☐
3 - 4 pm	☑	☐	☐	☐	☐	☐	5	2	0	3	☐
4 - 5 pm	☑	☐	☐	☐	☐	☐	5	2	0	3	☐
5 - 6 pm	☐	☑	☐	☐	☐	☐	5	2	0	3	☐
6 - 7 pm	☑	☐	☐	☐	☐	☐	2	0	0	0	☐

For peer review only - http://bmjopen.bmj.com/site/about/guidelines.xhtml

DAY 1

DATE _ _ / _ _ / _ _

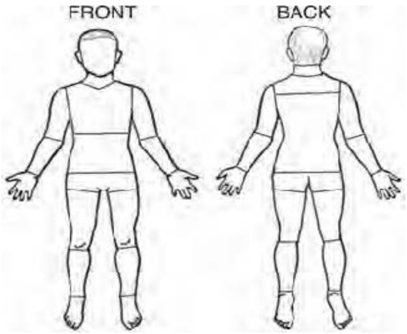

Did you apply sunscreen today?

☐ Yes ☐ No

If yes, shade the diagram on the left to show the parts of the body that you applied sunscreen to today

For peer review only

	**TIME OUTDOORS** (Cross the box which best represents the amount of time you spent outdoors during each one hour interval shown below)					**DID YOU APPLY SUNSCREEN**	**INSERT NUMBER DENOTING TYPE OF CLOTHING WORN** (You must write one number in each box)				
	0 minutes	Below 15 minutes	15-30 minutes	30-45 minutes	45-60 minutes	Cross as many as applicable	Upper body	Lower body	Head wear	Footwear	Gloves (cross all that apply)
**Morning**											
5 - 6 am	☐	☐	☐	☐	☐	☐					☐
6 - 7 am	☐	☐	☐	☐	☐	☐					☐
7 - 8 am	☐	☐	☐	☐	☐	☐					☐
8 - 9 am	☐	☐	☐	☐	☐	☐					☐
9 - 10 am	☐	☐	☐	☐	☐	☐					☐
10 - 11 am	☐	☐	☐	☐	☐	☐					☐
11 - 12 am	☐	☐	☐	☐	☐	☐					☐
**Afternoon**											
12 - 1 pm	☐	☐	☐	☐	☐	☐					☐
1 - 2 pm	☐	☐	☐	☐	☐	☐					☐
2 - 3 pm	☐	☐	☐	☐	☐	☐					☐
3 - 4 pm	☐	☐	☐	☐	☐	☐					☐
4 - 5 pm	☐	☐	☐	☐	☐	☐					☐
5 - 6 pm	☐	☐	☐	☐	☐	☐					☐
6 - 7 pm	☐	☐	☐	☐	☐	☐					☐

For peer review only - http://bmjopen.bmj.com/site/about/guidelines.xhtml

DAY 2

DATE _ _ / _ _ / _ _

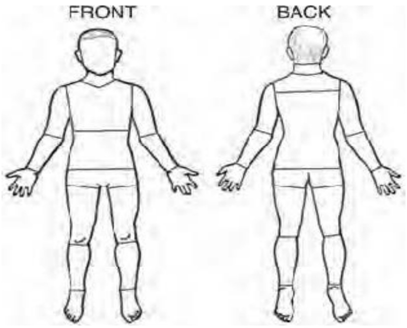

Did you apply sunscreen today?

☐ Yes ☐ No

If yes, shade the diagram on the left to show the parts of the body that you applied sunscreen to today

	TIME OUTDOORS (Cross the box which best represents the amount of time you spent outdoors during each one hour interval shown below)					DID YOU APPLY SUNSCREEN	INSERT NUMBER DENOTING TYPE OF CLOTHING WORN (You must write one number in each box)				
	0 minutes	Below 15 minutes	15-30 minutes	30-45 minutes	45-60 minutes	Cross as many as applicable	Upper body	Lower body	Head wear	Footwear	Gloves (cross all that apply)
**Morning**											
5 - 6 am	☐	☐	☐	☐	☐	☐					☐
6 - 7 am	☐	☐	☐	☐	☐	☐					☐
7 - 8 am	☐	☐	☐	☐	☐	☐					☐
8 - 9 am	☐	☐	☐	☐	☐	☐					☐
9 - 10 am	☐	☐	☐	☐	☐	☐					☐
10 - 11 am	☐	☐	☐	☐	☐	☐					☐
11 - 12 am	☐	☐	☐	☐	☐	☐					☐
**Afternoon**											
12 - 1 pm	☐	☐	☐	☐	☐	☐					☐
1 - 2 pm	☐	☐	☐	☐	☐	☐					☐
2 - 3 pm	☐	☐	☐	☐	☐	☐					☐
3 - 4 pm	☐	☐	☐	☐	☐	☐					☐
4 - 5 pm	☐	☐	☐	☐	☐	☐					☐
5 - 6 pm	☐	☐	☐	☐	☐	☐					☐
6 - 7 pm	☐	☐	☐	☐	☐	☐					☐

For peer review only - http://bmjopen.bmj.com/site/about/guidelines.xhtml

DAY 3

DATE _ _ / _ _ / _ _

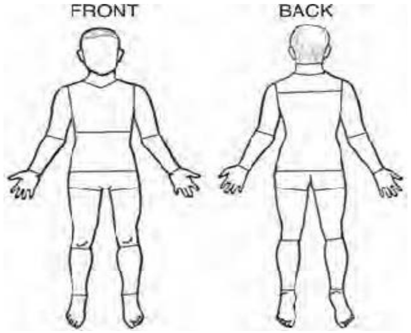

Did you apply sunscreen today?

☐ Yes ☐ No

If yes, shade the diagram on the left to show the parts of the body that you applied sunscreen to today

	**TIME OUTDOORS** (Cross the box which best represents the amount of time you spent outdoors during each one hour interval shown below)					**DID YOU APPLY SUNSCREEN**	**INSERT NUMBER DENOTING TYPE OF CLOTHING WORN** (You must write one number in each box)				
	0 minutes	Below 15 minutes	15-30 minutes	30-45 minutes	45-60 minutes	Cross as many as applicable	Upper body	Lower body	Head wear	Footwear	Gloves (cross all that apply)
**Morning**											
5 - 6 am	☐	☐	☐	☐	☐	☐					☐
6 - 7 am	☐	☐	☐	☐	☐	☐					☐
7 - 8 am	☐	☐	☐	☐	☐	☐					☐
8 - 9 am	☐	☐	☐	☐	☐	☐					☐
9 - 10 am	☐	☐	☐	☐	☐	☐					☐
10 - 11 am	☐	☐	☐	☐	☐	☐					☐
11 - 12 am	☐	☐	☐	☐	☐	☐					☐
**Afternoon**											
12 - 1 pm	☐	☐	☐	☐	☐	☐					☐
1 - 2 pm	☐	☐	☐	☐	☐	☐					☐
2 - 3 pm	☐	☐	☐	☐	☐	☐					☐
3 - 4 pm	☐	☐	☐	☐	☐	☐					☐
4 - 5 pm	☐	☐	☐	☐	☐	☐					☐
5 - 6 pm	☐	☐	☐	☐	☐	☐					☐
6 - 7 pm	☐	☐	☐	☐	☐	☐					☐

For peer review only - http://bmjopen.bmj.com/site/about/guidelines.xhtml

DAY 4

DATE _ _ / _ _ / _ _

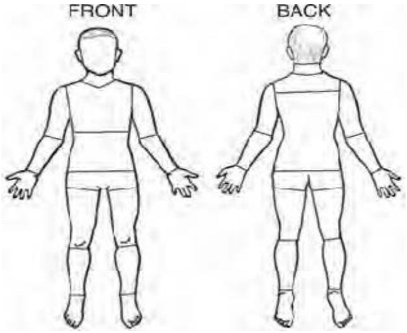

Did you apply sunscreen today?

☐ Yes ☐ No

If yes, shade the diagram on the left to show the parts of the body that you applied sunscreen to today

	**TIME OUTDOORS** (Cross the box which best represents the amount of time you spent outdoors during each one hour interval shown below)					**DID YOU APPLY SUNSCREEN**	**INSERT NUMBER DENOTING TYPE OF CLOTHING WORN** (You must write one number in each box)				
	0 minutes	Below 15 minutes	15-30 minutes	30-45 minutes	45-60 minutes	Cross as many as applicable	Upper body	Lower body	Head wear	Footwear	Gloves (cross all that apply)
**Morning**											
5 - 6 am	☐	☐	☐	☐	☐	☐					☐
6 - 7 am	☐	☐	☐	☐	☐	☐					☐
7 - 8 am	☐	☐	☐	☐	☐	☐					☐
8 - 9 am	☐	☐	☐	☐	☐	☐					☐
9 - 10 am	☐	☐	☐	☐	☐	☐					☐
10 - 11 am	☐	☐	☐	☐	☐	☐					☐
11 - 12 am	☐	☐	☐	☐	☐	☐					☐
**Afternoon**											
12 - 1 pm	☐	☐	☐	☐	☐	☐					☐
1 - 2 pm	☐	☐	☐	☐	☐	☐					☐
2 - 3 pm	☐	☐	☐	☐	☐	☐					☐
3 - 4 pm	☐	☐	☐	☐	☐	☐					☐
4 - 5 pm	☐	☐	☐	☐	☐	☐					☐
5 - 6 pm	☐	☐	☐	☐	☐	☐					☐
6 - 7 pm	☐	☐	☐	☐	☐	☐					☐

For peer review only - http://bmjopen.bmj.com/site/about/guidelines.xhtml

DAY 5

DATE _ _ / _ _ / _ _

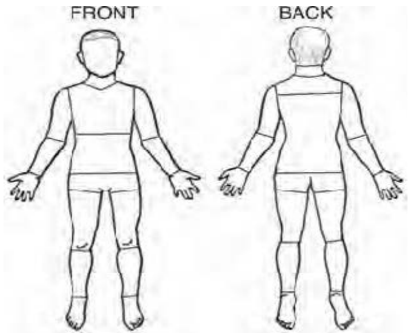

Did you apply sunscreen today?

☐ Yes ☐ No

If yes, shade the diagram on the left to show the parts of the body that you applied sunscreen to today

	TIME OUTDOORS (Cross the box which best represents the amount of time you spent outdoors during each one hour interval shown below)					DID YOU APPLY SUNSCREEN	INSERT NUMBER DENOTING TYPE OF CLOTHING WORN (You must write one number in each box)				
	0 minutes	Below 15 minutes	15-30 minutes	30-45 minutes	45-60 minutes	Cross as many as applicable	Upper body	Lower body	Head wear	Footwear	Gloves (cross all that apply)
**Morning**											
5 - 6 am	☐	☐	☐	☐	☐	☐					☐
6 - 7 am	☐	☐	☐	☐	☐	☐					☐
7 - 8 am	☐	☐	☐	☐	☐	☐					☐
8 - 9 am	☐	☐	☐	☐	☐	☐					☐
9 - 10 am	☐	☐	☐	☐	☐	☐					☐
10 - 11 am	☐	☐	☐	☐	☐	☐					☐
11 - 12 am	☐	☐	☐	☐	☐	☐					☐
**Afternoon**											
12 - 1 pm	☐	☐	☐	☐	☐	☐					☐
1 - 2 pm	☐	☐	☐	☐	☐	☐					☐
2 - 3 pm	☐	☐	☐	☐	☐	☐					☐
3 - 4 pm	☐	☐	☐	☐	☐	☐					☐
4 - 5 pm	☐	☐	☐	☐	☐	☐					☐
5 - 6 pm	☐	☐	☐	☐	☐	☐					☐
6 - 7 pm	☐	☐	☐	☐	☐	☐					☐

For peer review only

For peer review only - http://bmjopen.bmj.com/site/about/guidelines.xhtml

DAY 6

DATE _ _ / _ _ / _ _

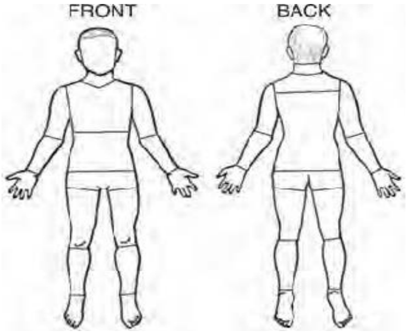

Did you apply sunscreen today?

☐ Yes ☐ No

If yes, shade the diagram on the left to show the parts of the body that you applied sunscreen to today

	**TIME OUTDOORS** (Cross the box which best represents the amount of time you spent outdoors during each one hour interval shown below)					**DID YOU APPLY SUNSCREEN**	**INSERT NUMBER DENOTING TYPE OF CLOTHING WORN** (You must write one number in each box)				
	0 minutes	Below 15 minutes	15-30 minutes	30-45 minutes	45-60 minutes	Cross as many as applicable	Upper body	Lower body	Head wear	Footwear	Gloves (cross all that apply)
**Morning**											
5 - 6 am	☐	☐	☐	☐	☐	☐	☐	☐	☐	☐	☐
6 - 7 am	☐	☐	☐	☐	☐	☐	☐	☐	☐	☐	☐
7 - 8 am	☐	☐	☐	☐	☐	☐	☐	☐	☐	☐	☐
8 - 9 am	☐	☐	☐	☐	☐	☐	☐	☐	☐	☐	☐
9 - 10 am	☐	☐	☐	☐	☐	☐	☐	☐	☐	☐	☐
10 - 11 am	☐	☐	☐	☐	☐	☐	☐	☐	☐	☐	☐
11 - 12 am	☐	☐	☐	☐	☐	☐	☐	☐	☐	☐	☐
**Afternoon**											
12 - 1 pm	☐	☐	☐	☐	☐	☐	☐	☐	☐	☐	☐
1 - 2 pm	☐	☐	☐	☐	☐	☐	☐	☐	☐	☐	☐
2 - 3 pm	☐	☐	☐	☐	☐	☐	☐	☐	☐	☐	☐
3 - 4 pm	☐	☐	☐	☐	☐	☐	☐	☐	☐	☐	☐
4 - 5 pm	☐	☐	☐	☐	☐	☐	☐	☐	☐	☐	☐
5 - 6 pm	☐	☐	☐	☐	☐	☐	☐	☐	☐	☐	☐
6 - 7 pm	☐	☐	☐	☐	☐	☐	☐	☐	☐	☐	☐

For peer review only - http://bmjopen.bmj.com/site/about/guidelines.xhtml

DAY 7

DATE _ _ / _ _ / _ _

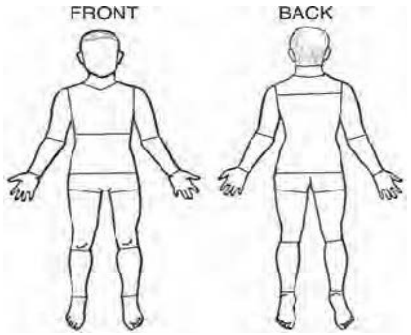

Did you apply sunscreen today?

☐ Yes ☐ No

If yes, shade the diagram on the left to show the parts of the body that you applied sunscreen to today

	TIME OUTDOORS (Cross the box which best represents the amount of time you spent outdoors during each one hour interval shown below)					DID YOU APPLY SUNSCREEN	INSERT NUMBER DENOTING TYPE OF CLOTHING WORN (You must write one number in each box)				
	0 minutes	Below 15 minutes	15-30 minutes	30-45 minutes	45-60 minutes	Cross as many as applicable	Upper body	Lower body	Head wear	Footwear	Gloves (cross all that apply)
**Morning**											
5 - 6 am	☐	☐	☐	☐	☐	☐					☐
6 - 7 am	☐	☐	☐	☐	☐	☐					☐
7 - 8 am	☐	☐	☐	☐	☐	☐					☐
8 - 9 am	☐	☐	☐	☐	☐	☐					☐
9 - 10 am	☐	☐	☐	☐	☐	☐					☐
10 - 11 am	☐	☐	☐	☐	☐	☐					☐
11 - 12 am	☐	☐	☐	☐	☐	☐					☐
**Afternoon**											
12 - 1 pm	☐	☐	☐	☐	☐	☐					☐
1 - 2 pm	☐	☐	☐	☐	☐	☐					☐
2 - 3 pm	☐	☐	☐	☐	☐	☐					☐
3 - 4 pm	☐	☐	☐	☐	☐	☐					☐
4 - 5 pm	☐	☐	☐	☐	☐	☐					☐
5 - 6 pm	☐	☐	☐	☐	☐	☐					☐
6 - 7 pm	☐	☐	☐	☐	☐	☐					☐

Thank you very much for answering the questions and completing the diary. Please be assured that this information will be kept confidential.

For peer review only - http://bmjopen.bmj.com/site/about/guidelines.xhtml

Appendix 4: Augmentative and Alternative Communication (AAC) Questionnaire

Communication System ○ Yes ○ No

Is it:

Makaton/ formal signs: number of signs: ______

Symbols or pictures for choices: number presented: ______

Communication board: number of words/ symbols: ______

Communication book: number of words/ symbols: ______

Single message device (eg Big Mack/ Step-by-Step)

Voice output communication device: name of device ____________________________

Access:

Eye pointing

Laser/ infra-red pointer

Visual scanning

Auditory scanning

Direct access (pointing)

Frequency of use hours per day: ☐☐

days per week: ☐

For peer review only - http://bmjopen.bmj.com/site/about/guidelines.xhtml

Appendix 5 PREDICT: Demographic questionnaire

What is your relationship to this child?

( ) Mother (biological or adoptive)

( ) Father (biological or adoptive)

( ) Step mother

( ) Step Father

( ) Legal Guardian (please explain)

What is your date of birth?

Does your child have any other difficulties, in addition to Cerebral Palsy, which would affect their participation in home, school or community activities (please tick any that apply)?

( ) Epilepsy

( ) Autism Spectrum Disorder (including Asperger Syndrome)

( ) ADHD

( ) Hearing Impairment

( ) Intellectual Impairment

( ) Learning Disability

( ) Visual Impairment

( ) Other (please explain)

Which best describes the household in which your child is currently living?

( ) Original, two parent family (both biological or adoptive parents are living in the same household as the child)

( ) Step, two parent family (two parents are living in the same household as the child and one is a step-parent)

( ) Shared custody (child lives in two separate households and divides his/her time between them)

( ) Sole parent family (Child lives with one parent only, may have contact with other parent)

For peer review only

For peer review only - http://bmjopen.bmj.com/site/about/guidelines.xhtml

( ) Other (please explain)

What is your current marital status?

( ) Married

( ) Defacto

( ) Separated

( ) Divorced

( ) Never married/defacto

( ) Widow/er

Does your child have any siblings living in the same household?

( ) Yes

( ) No

For each sibling living in the same household with your children please indicate the following:

	Sibling 1	Sibling 2	Sibling 3	Sibling 4
Age:				
Gender:				
Any difficulties that would affect their participation in home, school or community activities (please tick any that apply)?	( ) Nil<br><br>( ) Epilepsy<br><br>( ) Autism Spectrum Disorder (including Asperger Syndrome<br><br>( ) ADH<br><br>( ) Hearing Impairment<br><br>( ) Intellectual Impairment<br><br>( ) Learning	( ) Nil<br><br>( ) Epilepsy<br><br>( ) Autism Spectrum Disorder (including Asperger Syndrome<br><br>( ) ADH<br><br>( ) Hearing Impairment<br><br>( ) Intellectual Impairment<br><br>( ) Learning	( ) Nil<br><br>( ) Epilepsy<br><br>( ) Autism Spectrum Disorder (including Asperger Syndrome<br><br>( ) ADH<br><br>( ) Hearing Impairment<br><br>( ) Intellectual Impairment<br><br>( ) Learning	( ) Nil<br><br>( ) Epilepsy<br><br>( ) Autism Spectrum Disorder (including Asperger Syndrome<br><br>( ) ADH<br><br>( ) Hearing Impairment<br><br>( ) Intellectual Impairment<br><br>( ) Learning

For peer review only - http://bmjopen.bmj.com/site/about/guidelines.xhtml

	Disability<br><br>( ) Speech Language Difficulty<br><br>( ) Visual Impairment<br><br>( ) Other (please explain)	Disability<br><br>( ) Speech Language Difficulty<br><br>( ) Visual Impairment<br><br>( ) Other (please explain)	Disability<br><br>( ) Speech Language Difficulty<br><br>( ) Visual Impairment<br><br>( ) Other (please explain)	Disability<br><br>( ) Speech Language Difficulty<br><br>( ) Visual Impairment<br><br>( ) Other (please explain)

For peer review only

Age:

Gender:

Any difficulties that would affect their participation in home, school or community activities (please tick any that apply)?

( ) Epilepsy

( ) Autism Spectrum Disorder (including Asperger Syndrome)

( ) ADHD

( ) Hearing Impairment

( ) Intellectual Impairment

( ) Learning Disability

( ) Speech/Language Difficulty

( ) Visual Impairment

( ) Other (please explain)

Is English the main language spoken at home?

( ) Yes

( ) No, please specific the main language:

For peer review only - http://bmjopen.bmj.com/site/about/guidelines.xhtml

What is your postcode?

What is *your* highest level of education?

( ) Less than year 10

( ) Year 10/11

( ) Year 12

( ) Trade/apprenticeship

( ) TAFE/college certificate

( ) University degree

( ) University postgraduate degree

What is *your partner's* highest level of education?

( ) Less than year 10

( ) Year 10/11

( ) Year 12

( ) Trade/apprenticeship

( ) TAFE/college certificate

( ) University degree

( ) University postgraduate degree

Which best describes your current employment?

( ) Full time

( ) Part time

( ) Casual

( ) Full time parent/ home duties

( ) Unemployed (seeking work)

For peer review only

For peer review only - http://bmjopen.bmj.com/site/about/guidelines.xhtml

Which best describes your partner's current employment?

( ) Full time

( ) Part time

( ) Full time parent/ home duties

( ) Unemployed (seeking work)

Which best describes your family's combined annual income?

( ) <25,000

( ) 25,000-50,000

( ) 50,000-75,000

( ) 75,000-100,000

( ) 100,000-150,000

( ) 150,000+

What type of school does your child attend?

( ) State School

( ) Catholic School

( ) Independent (Private) School

( ) Special School

( ) Home schooled

( ) Other (please explain)

If your child does not attend a Special School, are they in a special class?

( ) Yes, my child is in a separate class for children with special needs

( ) No, my child is taught with the rest of his/her grade

Which grade is your child currently in?

For peer review only - http://bmjopen.bmj.com/site/about/guidelines.xhtml

( ) Prep

( ) 1

( ) 2

( ) 3

( ) 4

What support does your child receive at school? (Please tick all that apply)

( ) Nil

( ) Teacher Aide

( ) Learning support teacher/Specialist teacher

( ) School Nurse

( ) Occupational Therapy

( ) Physiotherapy

( ) Speech Pathology

( ) Academic/ Guidance Officer

( ) Other (please explain)

Does your child have an individual education plan (IEP – an individualised plan for your child's education)?

( ) Yes

( ) No

Does your child receive any other assistance with schooling (e.g. private tuition)?

( ) Yes, please describe

( ) No

Which of the following extra-curricular activities (outside of school) does your child participate in? (please tick all that apply). Please indicate for each activity, the number of times your child would participate in the activity (either formal lessons and/or practice:

For peer review only - http://bmjopen.bmj.com/site/about/guidelines.xhtml

For peer review only

Extra-curricular Activities	Frequency
( ) Nil	
( ) Music Classes (e.g. singing, instrument)	( ) Monthly ( ) Weekly ( ) 2-3 times/wk
( ) Creative Arts Classes (e.g. drama, drawing)	( ) Monthly ( ) Weekly ( ) 2-3 times/wk
( ) Team Sport (e.g. soccer, cricket)	( ) Monthly ( ) Weekly ( ) 2-3 times/wk
( ) Individual Sport (e.g. swimming, martial arts)	( ) Monthly ( ) Weekly ( ) 2-3 times/wk
( ) Dance (e.g. ballet, hip hop)	( ) Monthly ( ) Weekly ( ) 2-3 times/wk
( ) Social Clubs (e.g. scouts, girl guides)	( ) Monthly ( ) Weekly ( ) 2-3 times/wk
( ) Religious classes or clubs (e.g. Sunday school)	( ) Monthly ( ) Weekly ( ) 2-3 times/wk
( ) Other (please explain)	( ) Monthly ( ) Weekly ( ) 2-3 times/wk

For peer review only

For peer review only - http://bmjopen.bmj.com/site/about/guidelines.xhtml

Appendix 6: Qld PREDICT CP Study Health Resource Use Form

Allied Health

During the last 6 months, have you received….

1. Physiotherapy □ Yes □ No

Does it emphasise on □ Motor learning □ Functional therapy □ NDT therapy □ Postural management

How often □ weekly □ fortnightly □ monthly □ others (________)

Duration of session □ 30 minutes □ 45 minutes □ 60 minutes □ others (________)

Format □ individual □ group □ others (________)

Compliance □ very poor □ poor □ average □ good □ very good

2. Occupational therapy □ Yes □ No

Does it emphasise on □ CIMT therapy □ Goal directed training □ Postural management

How often □ weekly □ fortnightly □ monthly □ others (________)

Duration of session □ 30 minutes □ 45 minutes □ 60 minutes □ others (________)

Format □ individual □ group □ others (________)

Compliance □ very poor □ poor □ average □ good □ very good

3. Speech therapy □ Yes □ No

How often □ weekly □ fortnightly □ monthly □ others (________)

Duration of session □ 30 minutes □ 45 minutes □ 60 minutes □ others (________)

Format □ individual □ group □ others (________)

Compliance □ very poor □ poor □ average □ good □ very good

4. Conductive education □ Yes □ No

How often □ weekly □ fortnightly □ monthly □ others (________)

Duration of session □ 30 minutes □ 45 minutes □ 60 minutes □ others (________)

For peer review only

For peer review only - http://bmjopen.bmj.com/site/about/guidelines.xhtml

Format ☐ individual ☐ group ☐ others (________)

Compliance ☐ very poor ☐ poor ☐ average ☐ good ☐ very good

5. Point percussion ☐ Yes ☐ No

How often ☐ weekly ☐ fortnightly ☐ monthly ☐ others (________)

Duration of session ☐ 30 minutes ☐ 45 minutes ☐ 60 minutes ☐ others (________)

Format ☐ individual ☐ group ☐ others (________)

Compliance ☐ very poor ☐ poor ☐ average ☐ good ☐ very good

6. Acupuncture ☐ Yes ☐ No

How often ☐ weekly ☐ fortnightly ☐ monthly ☐ others (________)

Duration of session ☐ 30 minutes ☐ 45 minutes ☐ 60 minutes ☐ others (________)

Format ☐ individual ☐ group ☐ others (________)

Compliance ☐ very poor ☐ poor ☐ average ☐ good ☐ very good

7. Other therapy (________________) ☐ Yes ☐ No

How often ☐ weekly ☐ fortnightly ☐ monthly ☐ others (________)

Duration of session ☐ 30 minutes ☐ 45 minutes ☐ 60 minutes ☐ others (________)

Format ☐ individual ☐ group ☐ others (________)

Compliance ☐ very poor ☐ poor ☐ average ☐ good ☐ very good

8. Other therapy (________________) ☐ Yes ☐ No

How often ☐ weekly ☐ fortnightly ☐ monthly ☐ others (________)

Duration of session ☐ 30 minutes ☐ 45 minutes ☐ 60 minutes ☐ others (________)

Format ☐ individual ☐ group ☐ others (________)

Compliance ☐ very poor ☐ poor ☐ average ☐ good ☐ very good

For peer review only

9. Other therapy (________________) ☐ Yes ☐ No

How often ☐ weekly ☐ fortnightly ☐ monthly ☐ others (________)

Duration of session ☐ 30 minutes ☐ 45 minutes ☐ 60 minutes ☐ others (________)

Format ☐ individual ☐ group ☐ others (________)

Compliance ☐ very poor ☐ poor ☐ average ☐ good ☐ very good

10. Therapy Packages ☐ Yes ☐ No

Post BTX-A therapy package ☐ Yes ☐ No (see attached table)

Post surgery package ☐ Yes ☐ No (see attached table)

Compliance ☐ very poor ☐ poor ☐ average ☐ good ☐ very good

Medical

During the last 6 months, has your child had….

1. Hospital admission ☐ Yes ☐ No ☐ Number

Visit 1 Reason: ____

Length of stay: ____

Investigations performed and treatment received: ___________________________

____

Visit 2 Reason: ____

Length of stay: ____

Investigations performed and treatment received: ___________________________

____

Visit 3 Reason: ____

Length of stay: ____

Investigations performed and treatment received: ___________________________

For peer review only

____

Visit 4 Reason: ____

Length of stay: ____

Investigations performed and treatment received: ____________________________

____

2. Specialist medical appointments ☐ Yes ☐ No

Reason: ____

Length of stay: ____

Investigations performed and treatment received: ______________________________

____

3. Other out-patient appointments ☐ Yes ☐ No

Reason: ____

Length of stay: ____

Investigations performed and treatment received: ______________________________

____

Medication

During the last 6 months, has your child had….

1. Medication ☐ Yes ☐ No

For asthma ☐ Yes ☐ No

Which medication: ____

Frequency (number of times per day): ____

Dosage (per day): ____

Duration (length of treatment): ____

For peer review only - http://bmjopen.bmj.com/site/about/guidelines.xhtml

Any adverse events ☐ Yes ☐ No

Required admissions to hospital (see 'Medical') ☐ Yes ☐ No

2. For epilepsy ☐ Yes ☐ No

Frequency (number of times per day): ____

Dosage (per day): ____________________________

Duration: ____

Any adverse events ☐ Yes ☐ No

Required admissions to hospital (see 'Medical') ☐ Yes ☐ No

3. For saliva control ☐ Yes ☐ No

Frequency (number of times per day): ______________________

Dosage (per day): ____________________________

Duration: ____

Any adverse events ☐ Yes ☐ No

Required admissions to hospital (see 'Medical') ☐ Yes ☐ No

4. Others: ____

Frequency (number of times per day): ______________________

Dosage (per day): _____________________________

Duration: ____

Any adverse events ☐ Yes ☐ No

Required admissions to hospital (see 'Medical') ☐ Yes ☐ No

Spasticity management/ muscle contracture management

During the last 6 months, has your child received….

1.

For peer review only - http://bmjopen.bmj.com/site/about/guidelines.xhtml

BTX-A ☐ Yes ☐ No

No of limbs treated ☐ 1 ☐ 2 ☐ 3 ☐ 4 ☐ others (____)

No. of muscles per limb ☐ calf ☐ hamstrings ☐ adductors ☐ UL ☐ others (____)

Total dose (units BOTOX or Dysport): ____

Body weight at Rx date: ____

Units/ kilogram/ body weight: ____

Any adverse events ☐ Yes ☐ No

Required admissions to hospital (see 'Medical') ☐ Yes ☐ No

2.

Phenol ☐ Yes ☐ No

No of limbs treated ☐ 1 ☐ 2 ☐ 3 ☐ 4 ☐ others (____)

Obturator Nerve ☐ No

☐ Yes ☐ direct ☐ indirect ☐ other site (_________)

Total dose: ____

Any adverse events ☐ Yes ☐ No

Required admissions to hospital (see 'Medical') ☐ Yes ☐ No

3.

Oral anti-spastic medication ☐ Yes ☐ No

What medications: ____

Dosage (per day): ____

Duration: ____

Any adverse events ☐ Yes ☐ No

Required admissions to hospital (see 'Medical') ☐ Yes ☐ No

For peer review only - http://bmjopen.bmj.com/site/about/guidelines.xhtml

For peer review only

4.

Intrathecal Balcofen	☐ Yes	☐ No

Dosage (per day): ____

Pump refill	☐ Yes	☐ No
Any adverse events	☐ Yes	☐ No

Required admissions to hospital (see 'Medical') ☐ Yes ☐ No

5.

Soft tissue release	☐ Yes	☐ No			
What level	☐ Hip	☐ knee	☐ ankle	☐ foot	☐ spine
Which side	☐ unilateral	☐ bilateral			

6.

Bony reconstruction-derotational osteotomy	☐ Yes	☐ No			
What level	☐ Hip	☐ knee	☐ ankle	☐ foot	☐ spine
Which side	☐ unilateral	☐ bilateral			

7.

Salvage procedure	☐ Yes	☐ No			
What level	☐ Hip	☐ knee	☐ ankle	☐ foot	☐ spine
Which side	☐ unilateral	☐ bilateral			

8.

SEMLs	☐ Yes	☐ No			
What level	☐ Hip	☐ knee	☐ ankle	☐ foot	☐ spine
Which side	☐ unilateral	☐ bilateral			

For peer review only

For peer review only - http://bmjopen.bmj.com/site/about/guidelines.xhtml

Any adverse events ☐ Yes ☐ No

Required admissions to hospital (see 'Medical') ☐ Yes ☐ No

9.

Rhizotomy ☐ Yes ☐ No

Date and number of rootlets cut: ____

Which side ☐ unilateral ☐ bilateral

Any adverse events ☐ Yes ☐ No

Required admissions to hospital (see 'Medical') ☐ Yes ☐ No

10.

Serial casting ☐ Yes ☐ No

No of episodes ☐ 1 ☐ 2 ☐ 3 ☐ others (_______)

No. of limbs treated ☐ 1 ☐ 2

Total no of weeks done ☐ 1 ☐ 2 ☐ 3 ☐ 4 ☐ >4

Any adverse events ☐ Yes ☐ No

Required admissions to hospital (see 'Medical') ☐ Yes ☐ No

Equipment

During the last 6 months, have you received….

1.

Wheelchair ☐ Yes ☐ No

Is it ☐ New ☐ Continuing

Frequency of use hours per day: ________________________________

days per week: ________________________________

Compliance ☐ very poor ☐ poor ☐average ☐ good ☐ v. good

For peer review only

2.

Sticks	☐ Yes	☐ No			
Is it	☐ New	☐ Continuing			
Frequency of use	hours per day: ___				
	days per week: ___				
Compliance	☐ very poor	☐ poor	☐average	☐ good	☐ v. good

3.

Crutches	☐ Yes	☐ No			
Is it	☐ New	☐ Continuing			
Frequency of use	hours per day: ___				
	days per week: ___				
Compliance	☐ very poor	☐ poor	☐average	☐ good	☐ v. good

4.

Kaye Walker	☐ Yes	☐ No			
Is it	☐ New	☐ Continuing			
Frequency of use	hours per day: ___				
	days per week: ___				
Compliance	☐ very poor	☐ poor	☐average	☐ good	☐ v. good

5.

Pony Walker	☐ Yes	☐ No
Is it	☐ New	☐ Continuing
Frequency of use	hours per day: ___	
	days per week: ___	

For peer review only - http://bmjopen.bmj.com/site/about/guidelines.xhtml

Compliance	☐ very poor	☐ poor	☐average	☐ good	☐ v. good

6.

David Hart Walker	☐ Yes	☐ No
Is it	☐ New	☐ Continuing

Frequency of use hours per day: ______________________________

days per week: ______________________________

Compliance	☐ very poor	☐ poor	☐average	☐ good	☐ v. good

7.

Special seating	☐ Yes	☐ No

What brand: ____

Is it	☐ New	☐ Continuing

Frequency of use hours per day: ______________________________

days per week: ______________________________

Compliance	☐ very poor	☐ poor	☐average	☐ good	☐ v. good

8.

Standing frame	☐ Yes	☐ No

What brand: ____

Is it	☐ New	☐ Continuing

Frequency of use hours per day: ______________________________

days per week: ______________________________

Compliance	☐ very poor	☐ poor	☐average	☐ good	☐ v. good

9.

Orthoses	☐ Yes	☐ No

For peer review only

For peer review only - http://bmjopen.bmj.com/site/about/guidelines.xhtml

Is it	☐ New	☐ Continuing			
Is it	☐ fixed AFO	☐ hinged AFO		☐ KAFO	☐ HKFO
	☐ unilateral		☐ bilateral		
	☐ hip abduction brace (static or SWASH)				
	☐ Soft garment/ sleeves/ UPsuit				
	☐ Night time positioning equipment (list brand_____________)				
Which side	☐ unilateral	☐ bilateral			
Frequency of use	hours per day: ________________________________				
	days per week: ________________________________				
Compliance	☐ very poor	☐ poor	☐average	☐ good	☐ v. good

For peer review only - http://bmjopen.bmj.com/site/about/guidelines.xhtml

For peer review only
