# Supplementary material for: PREDICT-CP: study protocol of implementation of comprehensive surveillance to predict outcomes for school-aged children with cerebral palsy
Source: BMJ Open. 2017 Jul 12;7(7):e014950. doi: 10.1136/bmjopen-2016-014950 (PMC5734266; doi:10.1136/bmjopen-2016-014950)
Supplement: Reviewer comments [file bmjopen-2016-014950.reviewer_comments.pdf]

PEER REVIEW HISTORY

BMJ Open publishes all reviews undertaken for accepted manuscripts. Reviewers are asked to complete a checklist review form (**http://bmjopen.bmj.com/site/about/resources/checklist.pdf**) and are provided with free text boxes to elaborate on their assessment. These free text comments are reproduced below.

ARTICLE DETAILS

**TITLE (PROVISIONAL)**	PREDICT CP: Study Protocol of implementation of comprehensive surveillance to Predict outcomes for school aged children with Cerebral Palsy
**AUTHORS**	Boyd, Roslyn; Davies, Peter; Ziviani, Jenny; Trost, Stewart; Barber, Lee; Ware, Robert; Rose, Stephen; Whittingham, Koa; Sakzewski, Leanne; Bell, Kristie; Carty, Chris; Obst, Steven; Benfer, Katherine; Reedman, Sarah; Edwards, Priya; Kentish, Megan; Copeland, Lisa; Weir, Kelly; Davenport, Camilla; Brooks, Denise; Coulthard, Alan; Pelekanos, Rebecca; Guzzetta, Andrea; Fiori, Simona; Wynter, Meredith; Finn, Christine; Burgess, Andrea; Morris, Kym; Walsh, John; Lloyd, Owen; Whitty, Jennifer; Scuffham, Paul

VERSION 1 - REVIEW

**REVIEWER**	Gunnar Hägglund<br>Dept of Orthopaedics<br>Lund University<br>Sweden
**REVIEW RETURNED**	21-Mar-2017

**GENERAL COMMENTS**	This is a very comprehensive and well-designed study. The only uncertainty that I can see is that it requires that a high proportion of the 245 children and their parents agree to participate in the comprehensive study.

**REVIEWER**	Maryam Oskoui<br>McGill University<br>Canada
**REVIEW RETURNED**	01-Apr-2017

**GENERAL COMMENTS**	Please clearly identify a maximum of two primary outcomes, listing the remaining outcomes as secondary outcomes. You would then have a class II study in this population, which is much needed to have higher level of confidence in evidence. You capture great SES information from parents, and this could also be factored into your predictive models for various outcomes.

VERSION 1 – AUTHOR RESPONSE

Reviewer: 1: Reviewer Name: Gunnar Hägglund
Institution and Country: Dept of Orthopaedics, Lund University, Sweden Please state any competing interests or state 'None declared': None declared
This is a very comprehensive and well-designed study. The only uncertainty that I can see is that it

requires that a high proportion of the 245 children and their parents agree to participate in the comprehensive study.
Response: We acknowledge this uncertainty, however our experience has been very positive so far with this cohort who were followed from entry after confirmation of Diagnosis of CP on up to six occasions between 18 months C.A and 60 months (CP Child Study) with >98% retention. This cohort are now being invited to return for one outcome time (in 1-2 visits, for family convenience) and so far the response has been very positive. While some children may not undertake MRI at 8-10 years awake without sedation (though in all previous studies our ability for children to undertake brain MRI at this age has been >95%).

Reviewer: 2
Reviewer Name: Maryam Oskoui
Institution and Country: McGill University, Canada Please state any competing interests or state 'None declared': None declared
Question: Please leave your comments for the authors below Please clearly identify a maximum of two primary outcomes, listing the remaining outcomes as secondary outcomes. You would then have a class II study in this population, which is much needed to have higher level of confidence in evidence.
Response: We have amended the paper to have two primary outcomes Gross Motor Function (on GMFM-66) and Brain injury severity (on the Fiori Scale). Added on page 13:
This prospective longitudinal study follow-up has two primary outcomes (Hypothesis 1):
1. Gross motor function will be evaluated using the GMFM-66;
2. Brain lesion severity will be assessed using a structured scoring proforma (Fiori scale).
All additional measures are secondary outcome measures (Hypotheses 2-4).

Query: You capture great SES information from parents, and this could also be factored into your predictive models for various outcomes.
Response: As conducted in our previous studies we will factor SES into our predictive models by adjusting for tertiles on the SEIFA codes.
On page 26 we have added:
The socioeconomic status of Australian families will be classified into tertiles using scores on the Socioeconomic Indexes for Areas Index of Relative Disadvantage.181
